# Mechanistic Study of Photochemical Aminocarbonylation of Alkyl Iodides Catalyzed by a Palladium Catalyst Using Experimental and Computational Methods

**DOI:** 10.1021/acs.joc.4c02240

**Published:** 2025-02-25

**Authors:** Erik N. A. Sundén, Staffan Karlsson, Okky Dwichandra Putra, Måns Andreasson, Charles Elmore, Per-Ola Norrby, Malvika Sardana

**Affiliations:** † Early Chemical Development, Pharmaceutical Sciences, R&D, AstraZeneca, Gothenburg SE-43183, Sweden; ‡ Department of Chemistry, Ångström Laboratories, Uppsala University, P.O. Box 523, 751 20 Uppsala, Sweden; § Early Product Development and Manufacturing, Pharmaceutical Sciences, R&D, AstraZeneca, Gothenburg SE-43183, Sweden; ∥ Data Science and Modelling, Pharmaceutical Sciences, R&D, AstraZeneca, Gothenburg SE-43183, Sweden

## Abstract

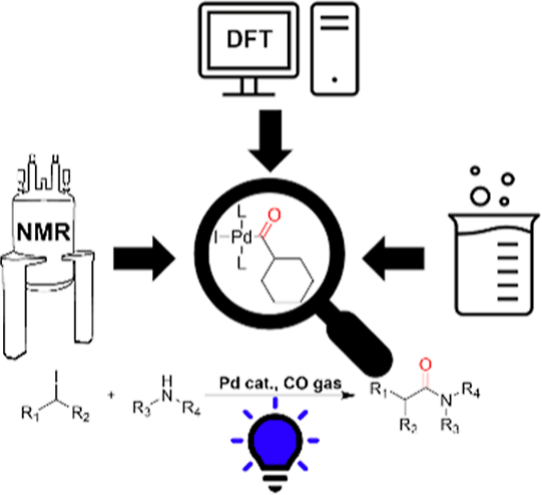

Aminocarbonylations are versatile reactions amenable to applications in convergent synthesis and isotope labeling. Herein, a mechanistic study of a previously reported visible-light-promoted aminocarbonylation of unactivated alkyl iodides is presented. This study combines in situ spectroscopy, computational chemistry, and organic chemistry techniques. A T1 excited-state promoted ligand dissociation in concert with an atom transfer radical addition was uncovered as a likely first step in the mechanism, instead of the usual three-center oxidative addition. Improvement in the reaction yield was achieved by optimizing the reaction based on mechanistic insights. This took the form of promoting a computationally uncovered cationic carbonylation pathway with the use of bidentate ligands.

## Introduction

Palladium-catalyzed aminocarbonylation reactions offer an enticing possibility for convergent synthesis and broad applicability with several protocols being available.^1−4^ Key among these is the protocol presented by Torres et al., which presents the opportunity of coupling aryl and alkyl halides with a broad range of nucleophiles at a high yield.^4^

In addition to their widespread applicability in organic synthesis, aminocarbonylations are inherently suited for isotopic labeling of compounds due to the broad functional group tolerance and ability to incorporate a one-carbon synthon at a late stage in the synthesis.^5^

**Figure 1:**
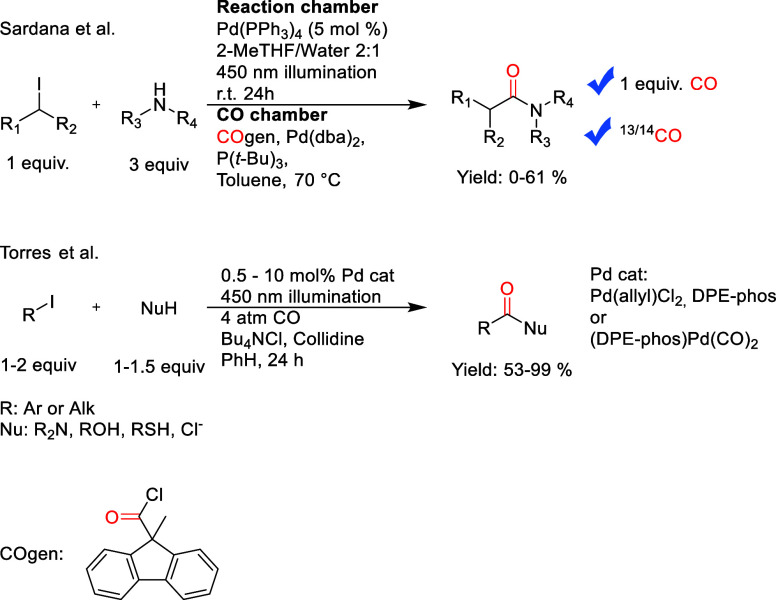
Reaction conditions employed by Sardana and co-workers and Torres and co-workers.

Most useful for the purpose of isotopic labeling are reactions employing a stoichiometric amount of carbon monoxide (CO), like the reactions described previously by Sardana and co-workers, shown in Figure 1, as well as Mühlfenzl and co-workers.^2,3^ The broad availability of carbon dioxide (CO_2_) and CO in ^13^C- and ^14^C-labeled form, or precursors to these compounds, also speaks to the synthetic utility of this reaction.^5−11^ Furthermore, the carbon monoxide used in this reaction is in the form of the surrogate COgen (9-methyl-9H-fluorene-9-carbonyl chloride), a solid, bench-stable reagent that liberates CO gas when exposed to a palladium catalyst. Thus, the handling of toxic gas is avoided, and the reaction setup is simplified significantly when a precise amount of carbon monoxide is desired as in isotope labeling.^5^

The reaction protocol presented by Sardana and Mühlfenzl has a broad applicability to both aryl and alkyl halides and requires light to achieve conversion. For the aryl iodides, it is expected that oxidative addition and subsequent carbonylation should be feasible in the ground state. The reductive elimination has, however, been proposed to be light driven.^4^ For the alkyl iodides, the oxidative addition has instead been shown to be infeasible in the ground state and thus is proposed to be the light-dependent step.^4^ No comprehensive investigation has been reported to support these claims.

To offer an avenue toward understanding and improving the reaction of Sardana and co-workers, a mechanistic study was deemed of interest. A previous work on similar reactions lends some insight and is outlined below.

Wang and co-workers performed a thorough study on the aminocarbonylation of aryl chlorides with ammonia catalyzed by Pd(dcpp) (1,3-Bis(dicyclohexylphosphino)propane).^12^ This reaction was thermally driven at 110 °C and extensively explored using mostly experimental methods. In addition, some density functional theory (DFT) calculations were used to assess reaction pathways that were difficult to measure experimentally. Notably, the authors found an off-cycle dimer that sequestered the catalyst and that either Pd(dcpp)CO or Pd(dcpp) was the active species for oxidative addition. The resting state of the catalyst was found to be Pd(dcpp)(CO)_2_. Kinetic studies also indicated that the formation of the amide bond proceeds by an inner sphere mechanism involving a ligand substitution of chloride with NH_3_, deprotonation, and finally a reductive elimination.^12^

A study by Kancherla and co-workers investigated a visible-light-induced palladium-catalyzed alkylation of α,β-unsaturated carboxylic acids by DFT and experimental methods.^13^ The model substrates used were cinnamic acid and bromocyclohexane. This study indicated that a ground-state oxidative addition has a prohibitively high energy barrier, while a T1 mechanism involving radicals has a reasonable activation energy for the oxidative addition.^13^ This single electron transfer mechanism was computationally predicted by Bickelhaupt and Ziegler in 1995.^14^

These literature examples can be used to generate a mechanistic hypothesis regarding the aminocarbonylation at hand; this work seeks to investigate the mechanism of this reaction in detail. For this purpose, computational methods were used to identify plausible reaction mechanisms. The mechanisms were then validated by conventional experimental methods, as well as in situ infrared (IR) and flow ^1^H nuclear magnetic resonance (NMR) spectroscopy.

## Computational Details

Calculations were done in Turbomole 7.6 using the RI-JK approximation and employing D3(BJ) with the three-body correction as the dispersion model.^15−21^ For scans, the RI-J approximation was used instead of RI-JK.^17,22−24^ Published free energy surfaces are corrected from 1 atm gas to 1 M solution standard state by adding 7.9 kJ/mol to each species Δ*G* value.^25^ Vibrational frequencies were scaled by 0.9547 in accordance with Kesharwani and co-workers to account for anharmonicity.^26^ To model THF in the COSMO framework, a dielectric constant of 7.43 and a refractive index of 1.4073 were chosen.^27,28^ THF was chosen as a substitute for 2-MeTHF as the latter is not available in the COSMO-RS solvation model.^29^ In all potential energy plots presented in this paper, dashed lines’ mean connectivity has not been validated, whereas solid lines’ mean IRC (intrinsic reaction coordinate), quick reaction coordinate (QRC), or a relaxed surface scan has been employed to validate connectivity. IRC means that for the two geometries which result when the TS is displaced along the negative eigenmode, a steepest descent optimization in mass-weighted coordinates was done using line searching to guarantee path following. This gives the reaction pathway without translation, rotation, or vibration, allowing transition states to be connected to their corresponding minima.^30^ QRC fulfills the same purpose but uses an initial displacement, followed by standard geometry optimization.^31^

Ground and first triplet (T1) state calculations were done by doing geometry optimizations and frequency calculations at the PBE0-D3(BJ)^15,16,32^/def2-SVP^33−35^/COSMO(THF)^36−38^ level, followed by a single point calculation at the PBE0-D3(BJ)^15,16,32^/def2-TZVPP^33−35^/DCOSMO-RS(THF)^29,36−38^ level. This methodology was chosen after extensive consultation of the benchmarking literature.^26,39−43^

For some compounds, the coupled perturbed Kohn–Sham equation iterations would not converge, likely due to limits on the accuracy of the COSMO cavity derivative.^44^ These are necessary for the calculation of the Hessian, which is then used for calculating entropy, zero point energy (ZPE) correction, and IR frequencies. Hence, relaxed convergence criteria ($force conversion 4) were employed for these geometries.

TDDFT calculations for S1 preequilibrium were done with PBE0-D3(BJ)/def2-TZVPP/DCOSMO-RS(THF) on T1 geometries.^45−47^ The scans for validating connectivity in the atom radical transfer addition were performed using ground-state T1 calculations with PBE0-D3(BJ)/def2-SVP/COSMO(THF).

## Results and Discussion

### Initial Mechanistic Proposal

**Figure 2:**
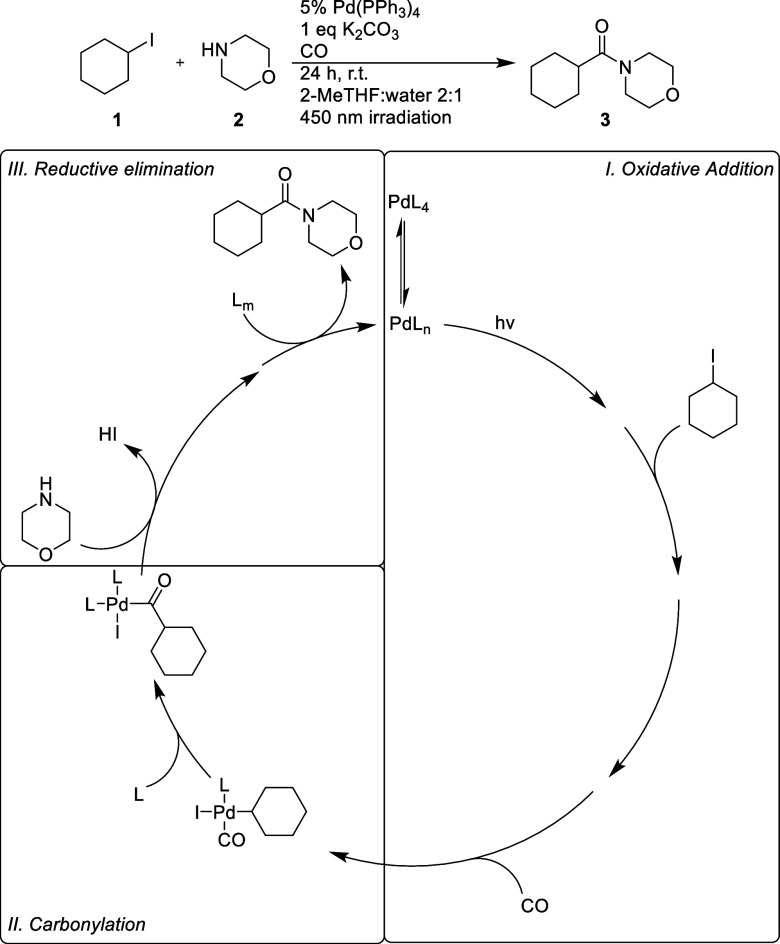
Initial postulated mechanism for the reaction.

The starting point of our study was based on the proposed mechanism presented in Figure 2.^5,48,49^ The mechanism was postulated to begin with an atom transfer radical addition (ATRA), as opposed to the typical oxidative addition, followed by carbonylation in the S0 state. Previous studies have assumed either an inner sphere reductive elimination or an outer sphere nucleophilic attack to complete the cycle.^1,12,50^

An inner sphere mechanism accounts for the observed kinetics of a palladium-catalyzed aryl aminocarbonylation, but the outer sphere mechanism does so as well if the step is solvent-assisted by a coordinating solvent like DMSO.^12^ This is quite interesting since another study, concerning a cobalt-catalyzed aminocarbonylation, the outer sphere mechanism seems favored.^51^

### Preequilibrium and Steady State

**Figure 3:**
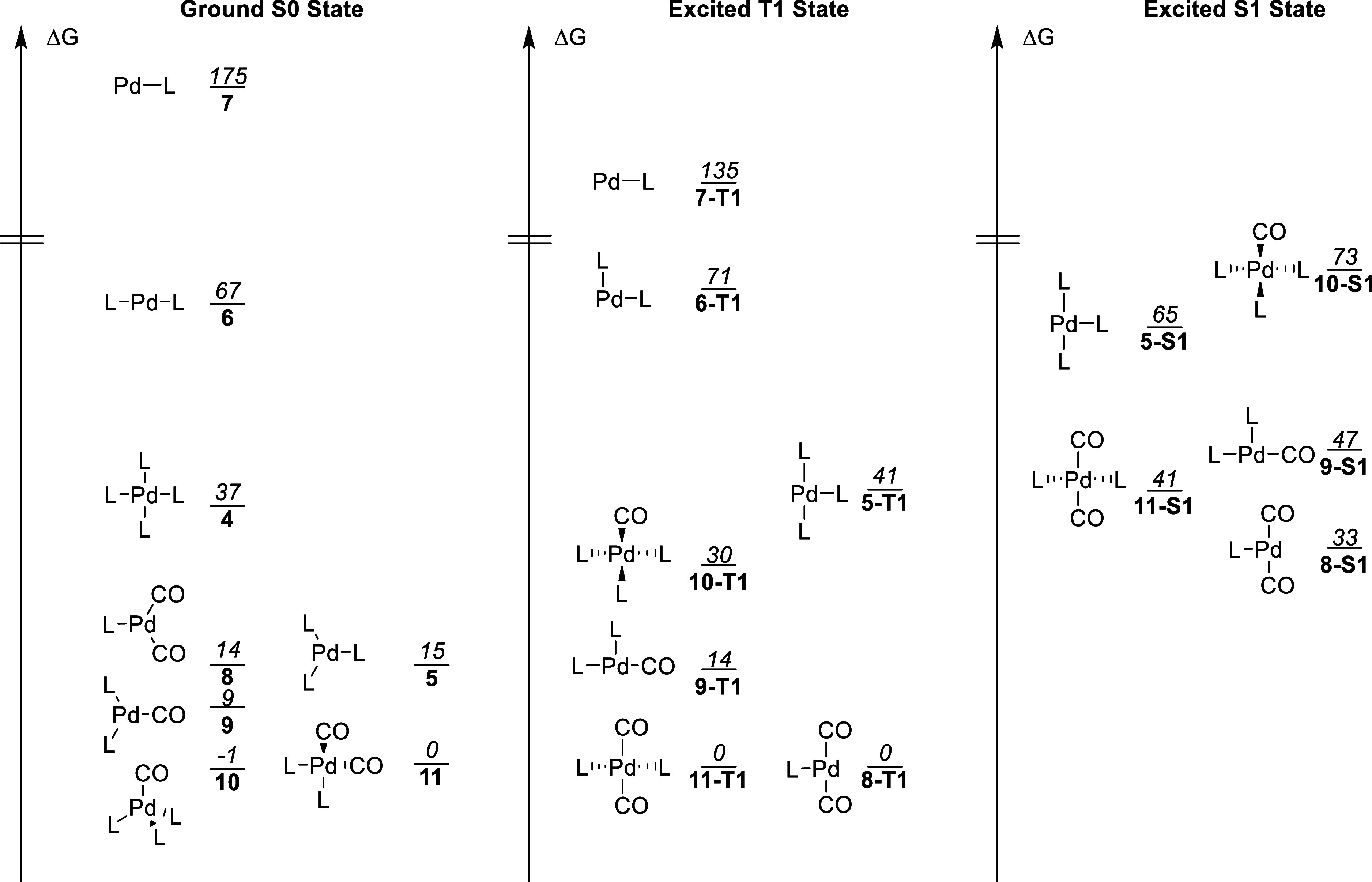
Free energies of all preequilibrium species in the S0 state as well as selected species in the T1 and S1 states. To allow for comparisons of the free energy landscapes, Bis-carbonyl **11** was chosen to have Δ*G* = 0 in S0 and T1, thus neglecting the excitation energies between these states. The S1 and T1 states are presented on a common energy scale with **11-T1** at Δ*G* = 0. The offset energy from **11** to **11-T1** is 193 kJ/mol. Free energies are reported in kJ/mol with dS and ZPE for S1 states taken from T1 calculations due to the lack of a TDDFT analytical Hessian in Turbomole. L = PPh_3_.

An investigation of the pre-equilibrium energies of potential palladium complexes was undertaken (Figure 3). Complexes **4**–**7** containing only PPh_3_ ligands are in good quantitative agreement with a previous study which used the B3LYP-D1 method.^52^ For the trivalent complexes in S0(**5**, **8**, and **9**) and the tetravalent species in S0 (**4**, **10**, and **11**), the monocarbonyl complex is preferred.

This ability of the Pd-complex to absorb and sequester CO from the headspace of the reaction chamber is likely the reason only one equiv of CO is required for good yields in the reaction. In the T1 state, Bis-carbonyl species are energetically preferred for both tri- and tetravalent species. The S0 to T1 excitation for both **10** and **11** corresponds to a palladium d-orbital electron being excited to the CO π* orbital, fully localized after geometry optimization. This would make the CO ligand less electron donating, and thus, more ligands could be accommodated on a metal center.

The geometries for all the complexes also change upon excitation to T1 due to the change in electron occupancy of Pd from s^0^d^10^ to s^1^d^9^; this causes a geometry change from tetrahedral coordination to a more directionally bonded square-planar geometry.^53^ However, the tetraligated complexes **11-T1** and **10-T1** exhibit a seesaw geometry and a skewed square-planar geometry, respectively, to ameliorate the steric interactions.

All complexes in S1 are higher in energy than the corresponding complex in T1, and the ligation preference for S1 seems to be very similar to T1 although tris ligation appears to be more favorable in S1 compared to T1 as can be seen on the relative energies of **5**, **8**, **11**, and **9** in these two states. Just like in T1, the S0 to S1 excitation corresponds to a palladium d-orbital electron being excited to the CO π* orbital. As was observed for T1 complexes more suited for a square-planar geometry are lower in energy.

The steady state of the pre-equilibrium of the reaction was probed by in situ IR spectroscopy, shown in Table 1.^12^ Signals corresponding to the complexes, which were predicted to be most stable in Figures 3, 10, and 11, were detected, as judged from DFT-predicted carbonyl stretching frequencies.

**Table 1:** Detected and Predicted IR Signals of Different Complexes in the Reaction Solution

complex	predicted IR (cm^–1^)	detected IR (cm^–1^)
10	1945	1965
11	1984, 2027	1965, 2020

To investigate if solvent participation was a possibility in carbonyls **10** and **11**, a solution of Pd(PPh_3_)_4_ in 1,2-dichloroethane was titrated with 2-MeTHF. No shift in the carbonyl peak wavenumber in the IR was observed, indicating a lack of solvent participation.

### Oxidative Addition

**Figure 4:**
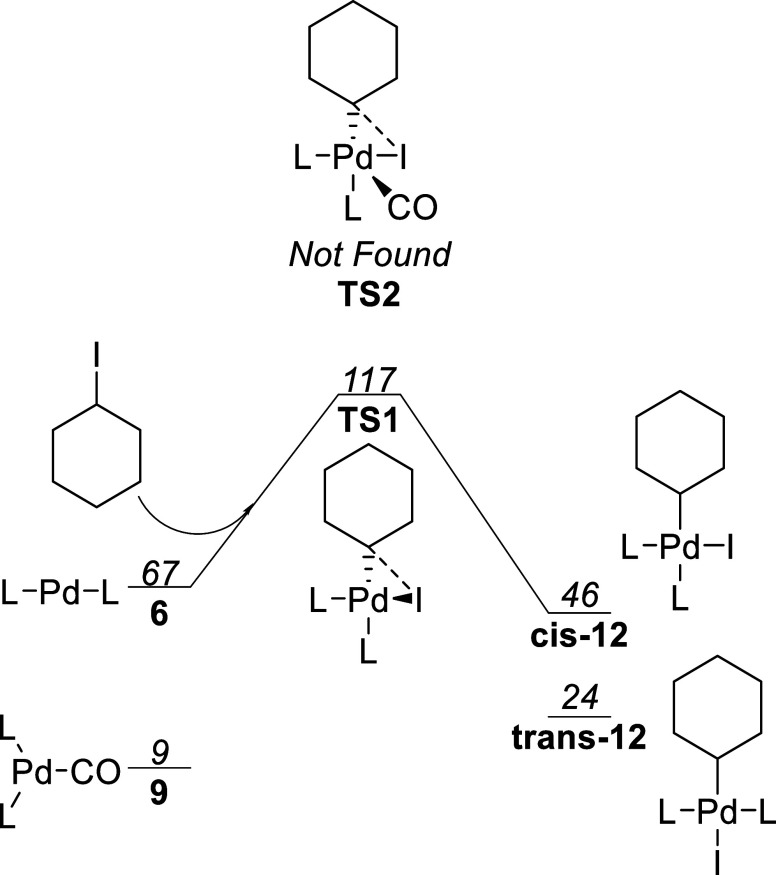
Two possible oxidative addition pathways in S0. Free energies reported in kJ/mol relative to complex **11-S0** with L = PPh_3_.

To test the hypothesis that this step was light driven, the activation energies for the normal three-center oxidative addition starting from bis-ligated complex **6**, leading to complex **cis**-**12-S0**, was first calculated, in addition to a pathway from monocarbonyl complex **9**, via **TS2**, as proposed by Wang and co-workers.^12^ For the latter pathway via **TS2**, no transition state geometry could be located. However, this is not unexpected since Wang and co-workers studied an aryl halide, rather than an alkyl halide.^12^ These calculations are shown in Figure 4. The activation energy relative to the proposed catalyst resting state **11** is in excess of 100 kJ/mol; hence, this is unlikely to be the reaction pathway.

**Figure 5:**
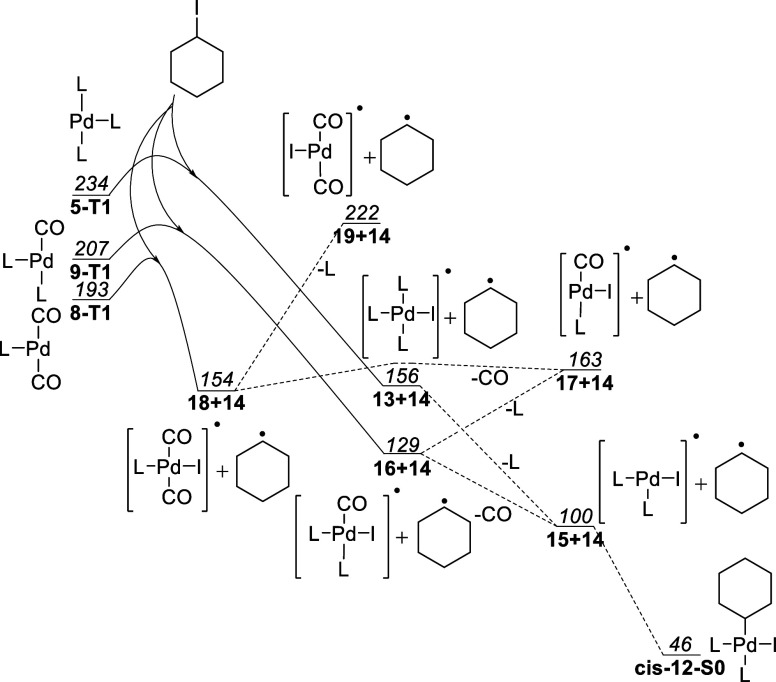
Pathways for atom radical transfer in T1. Free energies reported in kJ/mol relative to complex **11-S0** with L = PPh_3_.

Therefore, an investigation into T1 atom radical transfer (ATRA) was initiated to determine if the formation of an alkyl complex is viable through photocatalysis. The S1 state was not investigated since the expected lifetimes are so short that anything except intramolecular reactions is expected to be highly improbable. At first, the stationary points concerning such an oxidative addition were calculated with free energies (Figure 5). There are two different biradical pathways, both plausible and strongly exergonic, leading toward complex **cis-12-S0**.

Starting from phosphine complex **5-T1**, an iodine abstraction from alkyl iodide **1** is favorable, generating Pd(I) complex **13** and cyclohexyl radical **14**. Complex **13** can then undergo ligand dissociation to yield complex **15** with a free coordination site. This site can then be filled by radical **14**, with a spin flip at any point on the reaction path, to give alkyl complex **cis-12-S0**, which is the same product that a three-center oxidative addition would give.

Analogously, a pathway starting from monocarbonyl **9-T1** was calculated. An iodine abstraction gives cyclohexyl radical **14** and Pd(I) complex **16**, which can either dissociate a triphenylphosphine ligand to give complex **17** or dissociate the CO ligand yielding complex **15**. Here, it is interesting to note that DFT predicts that the formation of **15** is more favorable than that of **17**, despite CO generally being held as a stronger ligand in S0 and being less sterically demanding than triphenylphosphine. By formation of thermodynamically favorable Pd(I) complex **15**, recombination with the cyclohexyl radical **14** once again gives **cis-12**.

Excitation of Bis-carbonyl **11** to **11-T1** followed by dissociation of the ligand to Bis-carbonyl **8-T1** can also give yet another pathway. Iodine abstraction gives the Pd(I) complex **18** and the cyclohexyl radical **14**. Slightly endothermic dissociation of CO to **17** followed by phosphine ligand association brings this complex back to **16** on the common path.

We did not calculate transition states for the excited states in Figure 5. It can be postulated that exergonic processes in the excited state are allowed; moreover, at some point, the reaction path will cross over to S1 or S0 to allow the final radical coupling to give **cis-12-S0**. This is made possible by the high spin–orbit coupling of palladium and iodine which allows for fast intersystem crossing.^54,55^

The T1 iodine abstraction was scanned using the carbon iodine bond as the reaction coordinate using complexes **5**, **8**, and **9** as the starting material. This shows that in the T1 state, the abstraction is a monotonous downhill process with a minimum at infinite separation between Pd(I) and the radical. For none of these processes is there a transition state on the potential energy surface. As proposed by Harvey and co-workers, barrierless reactions can be assumed to be diffusion limited.^56^ The activation energy can be calculated from the viscosity of the solvent, for 2-MeTHF at room temperature via the Stokes–Einstein equation and Ficks first law as derived as shown in Atkins (eq 188.4), this corresponds to 15 kJ/mol.^57,58^

Binding of the alkyl radical onto Pd(I) species **15** and **17** was scanned in the same way. In the T1 state, the reaction has quite a small driving force but is not associated with a barrier.

Hence, if the reaction is solvent-caged, we expect the iodine abstraction to result in the two same spin radicals which have to recombine in a T1 state. Based on the strong spin–orbit coupling from palladium and iodine, it is likely that a pathway exists which would allow **cis-12** to relax to the S0 state upon radical recombination. However, it was not possible to study this with the computational techniques at hand.^59^

Another possibility is that the reaction is not solvent-caged and that the alkyl radical and Pd(I) species diffuse away from each other and find a reaction partner of opposite spin, thus leading to a singlet state immediately. It is also formally possible that the radicals separate and trigger catalytic cycles on the doublet surface, but this is unprecedented and was not investigated since closed shell pathways were found to rationalize all observations after the oxidative addition.

**Figure 6:**
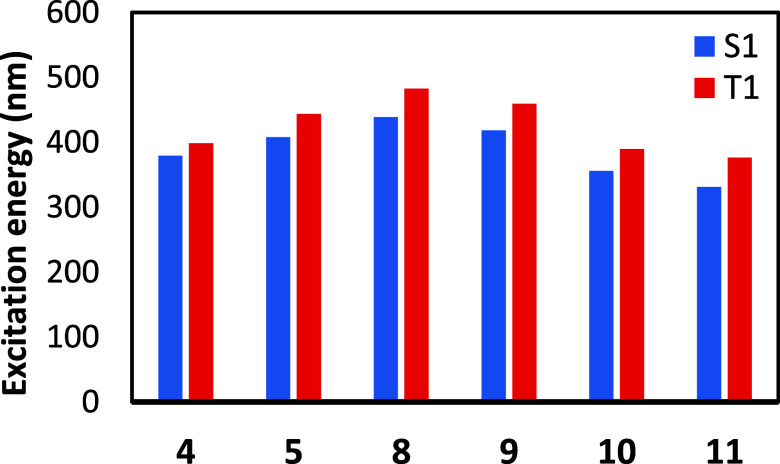
TDDFT excitation energies, in nanometers, for selected complexes are shown for both the T1 and S1 excitation.

To further investigate the most likely pathway for ATRA, the S1 and T1 vertical excitation energies were calculated by TDDFT for the complexes which are energetically reachable in the S0 state, as shown in Figure 6. We observe that most complexes are predicted to absorb close to the 450 nm illumination wavelength.

The complex with the lowest predicted excitation energy in the reactive T1 state is **8**, which would indicate it to be reactive. However, we are aware that the ATRA process for complex **8** involves endothermic reaction steps, which lowers the probability that an excited-state process could outcompete relaxation to the ground state. However, complexes **5** and **9** have predicted absorption maxima very close to 450 nm and have diffusion-limited and exothermic reaction pathways. Thus, both of these complexes could be the photoactive specie. One further differentiating factor does exist; monocarbonyl **9** is predicted to be more stable than complex **5** in the ground state (Figure 3); thus, more of the catalyst should be available in this state to absorb light.

Considering the facts presented above, we judge it most likely that complex **9** is the photoactive species which is excited to **9-T1** after which it undergoes ATRA, dissociating a CO ligand to give **cis-13**. However, several other pathways are also likely possible, especially since Kancherla and co-workers achieved an ATRA-type reaction without CO.^13^

To support radical involvement in the reaction, both diastereomers of alkyl iodide **20** were prepared and subjected to the aminocarbonylation reaction to yield compound **21**. If radicals are involved in the oxidative addition, as was predicted by DFT, we would expect the same diastereomeric ratio (dr) regardless of which the diastereomer of **20** was used.

**Figure 7:**
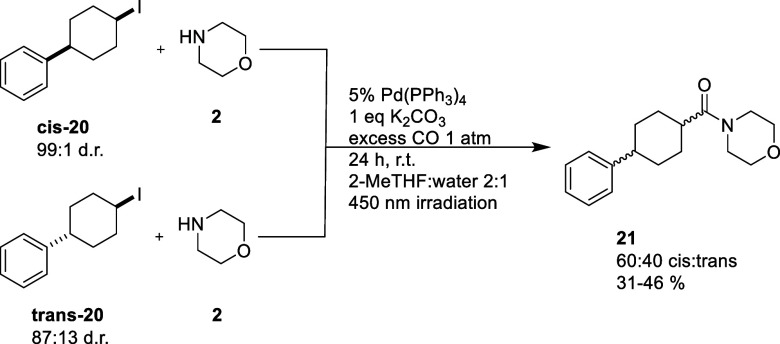
Visible-light-enabled aminocarbonylation of **cis-20** and **trans-20** to give a mixture of cis and trans **21**.

Analysis of the crude reaction mixtures from the reactions employing **cis-20** or **trans-20** showed that both reactions afforded compound **21** with an identical d.r. of 60:40 cis/trans (Figure 7). A cationic intermediate could also lead to diastereomeric scrambling, hence necessitating further investigation.

To further support the intermediacy of radicals, we choose not to use common radical detection methods like TEMPO ((2,2,6,6-tetramethylpiperidin-1-yl)oxyl) or thiols since these have been shown in the literature to be able to interact in unfavorable ways even with nonradical palladium complexes leading to false positives.^60^

DEPMPO (5-(diethoxyphosphoryl)-5-methyl-1-pyrroline-*N*-oxide) and DMPO (5,5-dimethyl-1-pyrroline *N*-oxide) are, however, potent radical traps, without known palladium interactions.^61^ After trapping a radical, the species affords the same type of aminoxyl radical as TEMPO.

**Figure 8:**
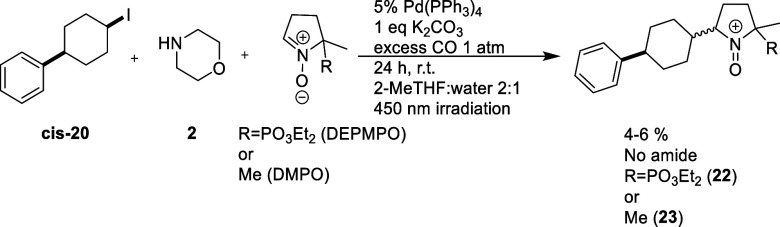
Visible-light-enabled aminocarbonylation of **cis-20** in the presence of DEPMPO and DMPO.

A competition reaction using 0.94 equiv of **cis-20** and 1 equiv of DEPMPO/DMPO inhibited the aminocarbonylation; product **21** was not detected by LC–MS. Instead, adducts **22** and **23** were detected by high-resolution mass spectrometry for DEPMPO and DMPO, respectively (Figure 8).

### Carbonylation

**Figure 9:**
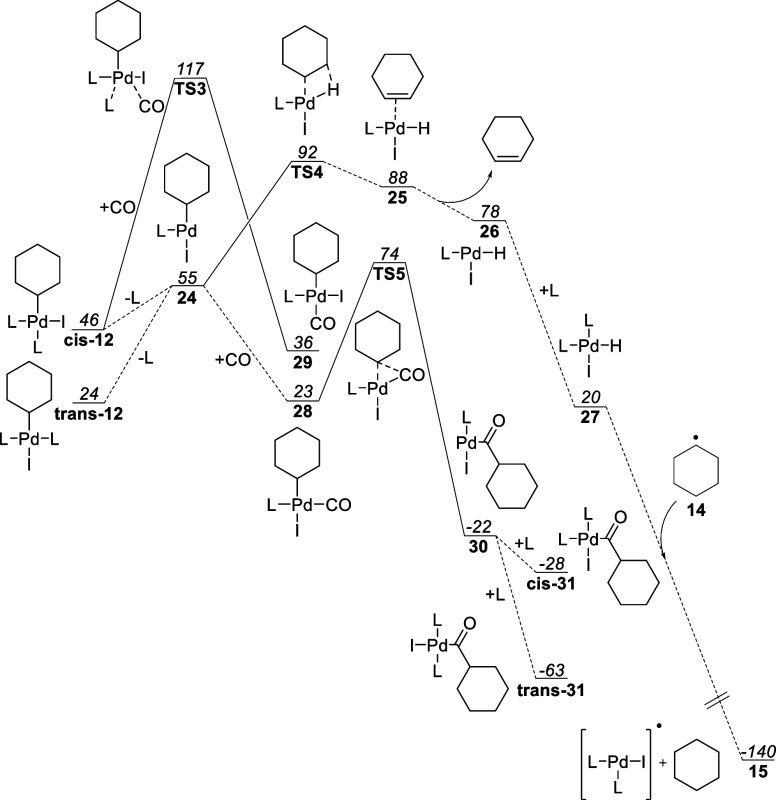
CO absorption into complex **13**, neutral carbonylation, as well as beta-hydride-elimination pathways. Free energies are reported in kJ/mol relative to complex **11-S0** with L = PPh_3_.

With the evidence supporting a photochemically catalyzed oxidative addition, the remainder of the mechanism was investigated in the ground state (Figure 9). Ligand dissociation from alkyl complex **12** affords complex **24** (Figure 9). In the absence of CO, complex **24** can undergo β-hydride elimination via **TS4** to form the alkene complex **25**. Exergonic dissociation of cyclohexene yields complex **26**, which can coordinate one more phosphine ligand to form hydride complex **27**. This complex can then be attacked by cyclohexyl radical **14** to form cyclohexane and stable Pd(I) species **15** (Figure 9). This entire process has a predicted overall activation energy of 68 kJ/mol.

This possible reaction was investigated in the absence of CO and amine using flow NMR. The reaction mixture was continuously monitored by sequential 1D-WET (water suppression enhanced through T1 effects) analysis.^62^ To allow for solvent suppression with this equipment, the solvent was switched to benzene, a solvent reported as giving similar yields to 2-MeTHF/water in the original paper.^2^

**Figure 10:**
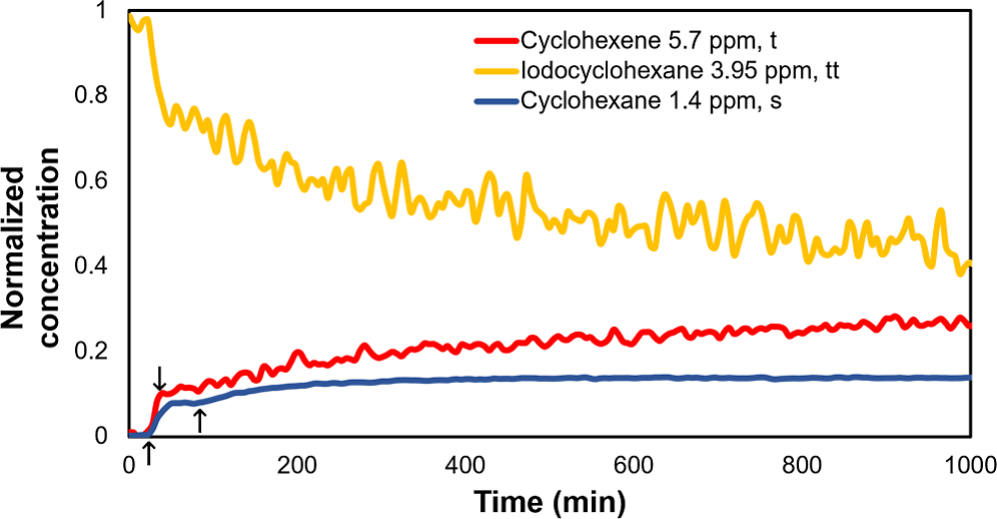
Integral curves obtained when first illuminating iodocyclohexane mixed with the catalyst overnight. ↑ = light turned on, ↓ = light turned off.

When the reaction mixture was irradiated in the absence of CO, substantial amounts of cyclohexane and cyclohexene were produced (Figure 10). This is consistent with the DFT predictions (Figure 9). As expected, the oxidative addition is observed even without CO, and the resulting complex undergoes beta-hydride elimination to give cyclohexene. Cyclohexane and cyclohexene have only been detected when the reaction was run in the absence of morpholine and CO, although we did not attempt to detect them under normal reaction conditions due to analytical limitations.

In the presence of CO, complex **24** can instead absorb a CO ligand to give complex **28**. An alternative mechanism via an associative pathway forming **29** via **TS3** was found to be unfavorable. Furthermore, complex **28** could alternatively stem from ATRA on monocarbonyl complex **9-T1** or **8-T1** via **17**.

Thermodynamics favor complex **28** over **29**. The cis orientation of the CO ligand and alkyl group in **28** is properly oriented to allow for carbonylative–insertion reaction via **TS5** to afford acyl complex **30**. Subsequent absorption of a ligand gives bis-ligated **cis-31** or **trans-31**, thus concluding the carbonylation step. An inspection of the free energy plot shows that the overall activation energy is 50 kJ/mol, making this reaction quite fast and is expected to easily outcompete the β-hydride elimination if CO is present in sufficient concentration.

**Figure 11:**
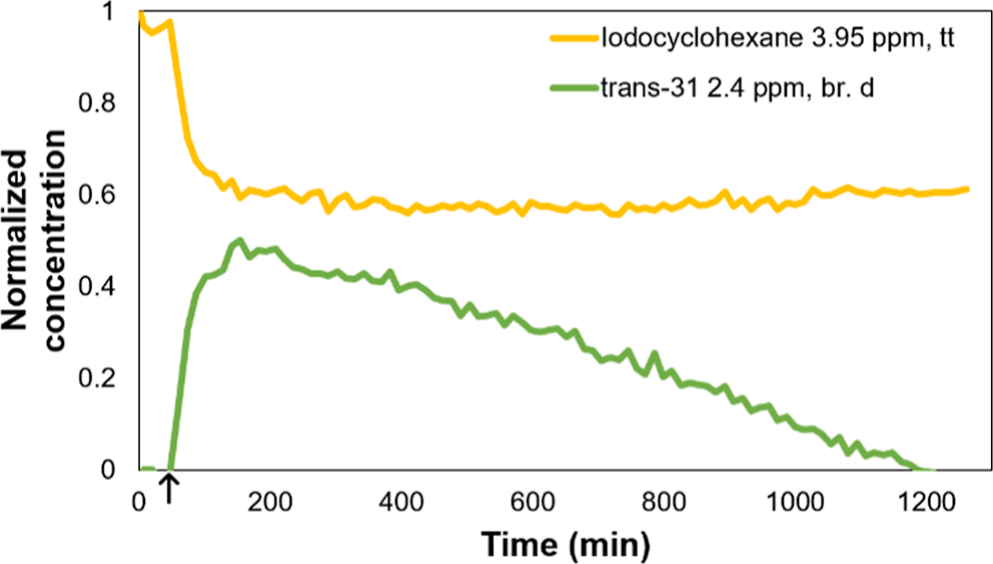
Integral curves obtained when illuminating iodocyclohexane and catalyst with CO gas added from the start. ↑ = light turned on, ↓ = light turned off.

To further support the computational investigation of the carbonylation, CO was added to Pd(PPh_3_)_4_ and cyclohexyl iodide **1** in benzene (Figure 11). When the light is switched on, a 2.4 ppm doublet forms and then rapidly decays by zero-order kinetics. We concluded that the signal at 2.4 ppm (Figure 11, green) likely belongs to **trans-31**.

**Figure 12:**
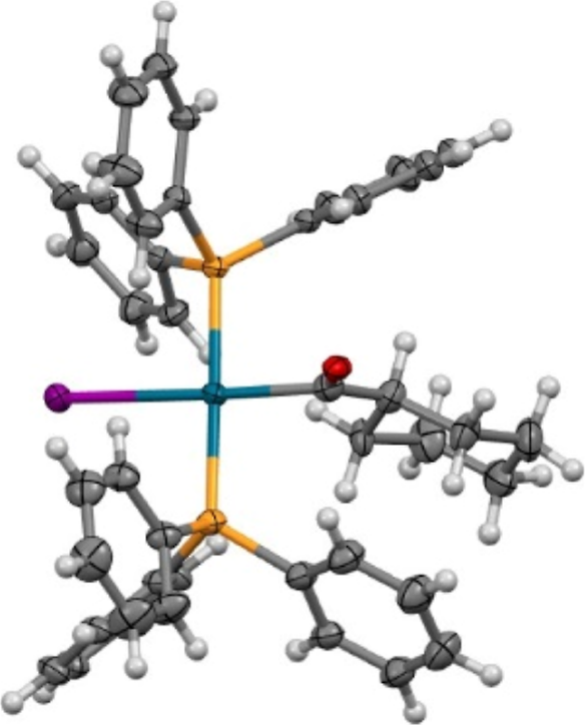
Thermal ellipsoid drawing of compound **trans-31** drawn at 50% probability level. The minor part of disorder, corresponding to the cyclohexane ring positional disorder, and cocrystallized benzene molecules are omitted for clarity.

To support this assumption, an aliquot was taken at 300 min when 2.5 ppm of species peaked. From this aliquot was grown a crystal for single-crystal X-ray diffraction (SC-XRD) under a CO atmosphere. SC-XRD showed the **trans-31** complex (Figure 12), while NMR was inconclusive due to exchange dynamics at room temperature.

To investigate if a cationic carbonylation was viable, experiments were conducted where the standard reaction was conducted in the presence of 1 equiv of dppe (1,2-Bis(diphenylphosphino)ethane) or dppp (1,2-Bis(diphenylphosphino)propane). Due to the entropic cooperativity of the two linked phosphines, it is expected that the bidentate ligand displaces two PPh_3_ from Pd(PPh_3_)_4_ forming the active catalyst in these experiments. This was chosen instead of a computational investigation due to the perceived inadequacy of implicit solvation models when applied to heterolytic bond cleavage.^63^

To our surprise, dppp boosted the NMR yield of the reaction from 51 to 80% yield compared to the standard conditions. This shows that bidentate ligands of interest for further investigation, vide infra, and additionally that the cationic carbonylation pathway must be possible, at least with a bidentate ligand. Another contributing factor could be that deactivation of the catalyst is likely slower with a more strongly bound bidentate ligand.

Motivated by the experimental result, the cationic carbonylation pathway was calculated for both L = PPh_3_ and L = dppp. However, we recognize that large errors can be expected, caused by inadequate solvation models for heterolytic bond cleavage.^63^

**Figure 13:**
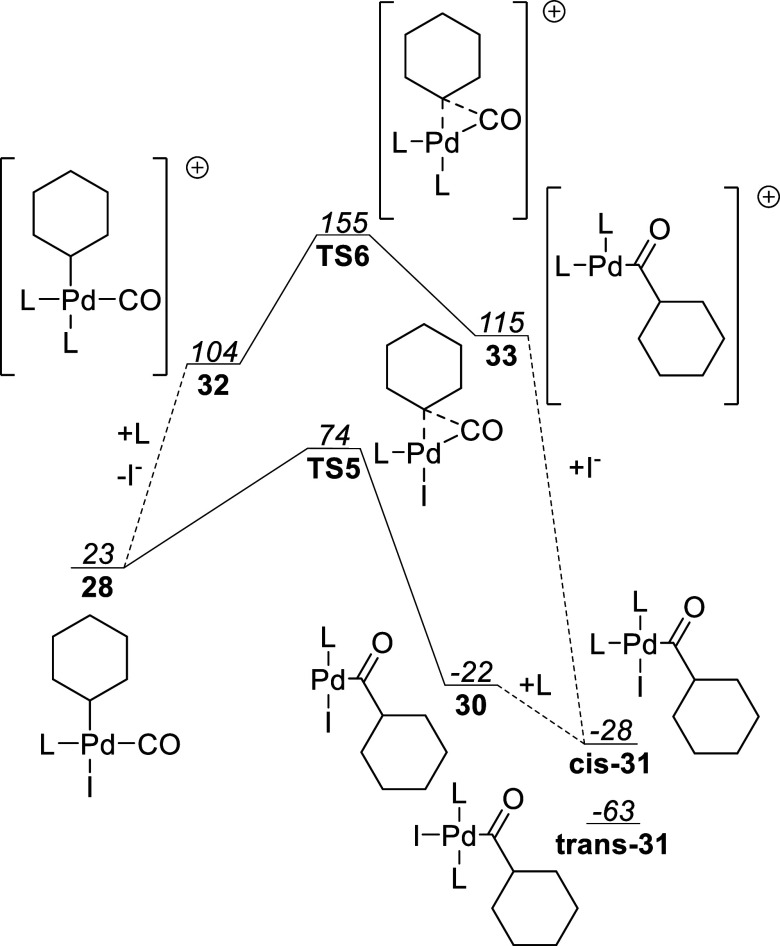
Comparison of the neutral and cationic carbonylation pathway for L = PPh_3_. Free energies reported in kJ/mol relative to complex **11-S0** with L = PPh_3_.

In Figure 13 the neutral carbonylation pathway is predicted to be favorable with a difference in overall activation energy of 81 kJ/mol compared with the cationic pathway. Most of this difference stems from the unfavorable conversion of **28** to **32** by replacement of iodide with a PPh_3_ ligand. The predicted activation energy for the carbonylation step is identical between the cationic and neutral pathway, indicating that the overall charge does not affect the carbonylative insertion in **TS6**. The cationic acyl complex **33** is also destabilized compared to the neutral acyl species **30** which would further disfavor the pathway.

**Figure 14:**
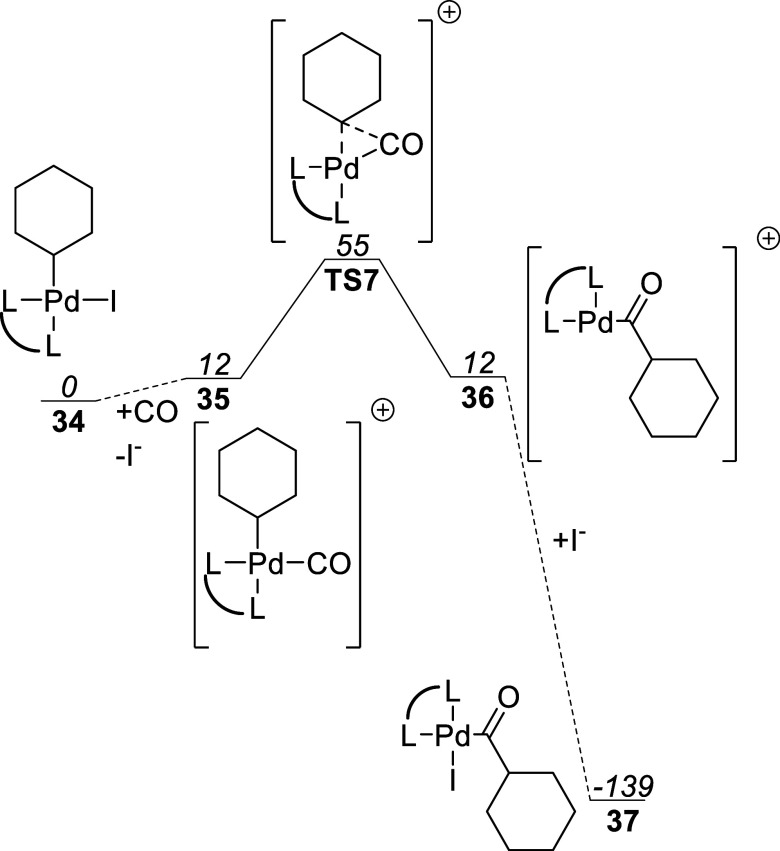
Free energy pathway for cationic carbonylation using dppp as the ligand. Free energies reported in kJ/mol relative to complex **34**.

This stands in stark contrast to the predicted carbonylation pathway when dppp is used as the ligand, for which the free energy plot is shown in Figure 14. The ligand change to dppp renders the replacement of iodide with CO from neutral complex **34** to cationic complex **35** only very slightly endergonic. Thus, the overall activation energy to reach **TS7** is much lower than that for reaching cationic **TS6** with L = PPh_3_, 55 kJ/mol compared to 132 kJ/mol. Further, the carbonylation of carbonyl complex **35** to acyl complex **36** does not change the Gibbs free energy allowing more of complex **36** to coexist in solution with **35** to absorb iodide, compared to **33**, increasing the overall rate.

### Reductive Elimination

This reaction step was only studied by DFT due to the excellent prior work by Wang and co-workers.^12^ A hydroxide ion is used as a model base to preserve the total charge over the reductive elimination to reduce the solvation model error.

**Figure 15:**
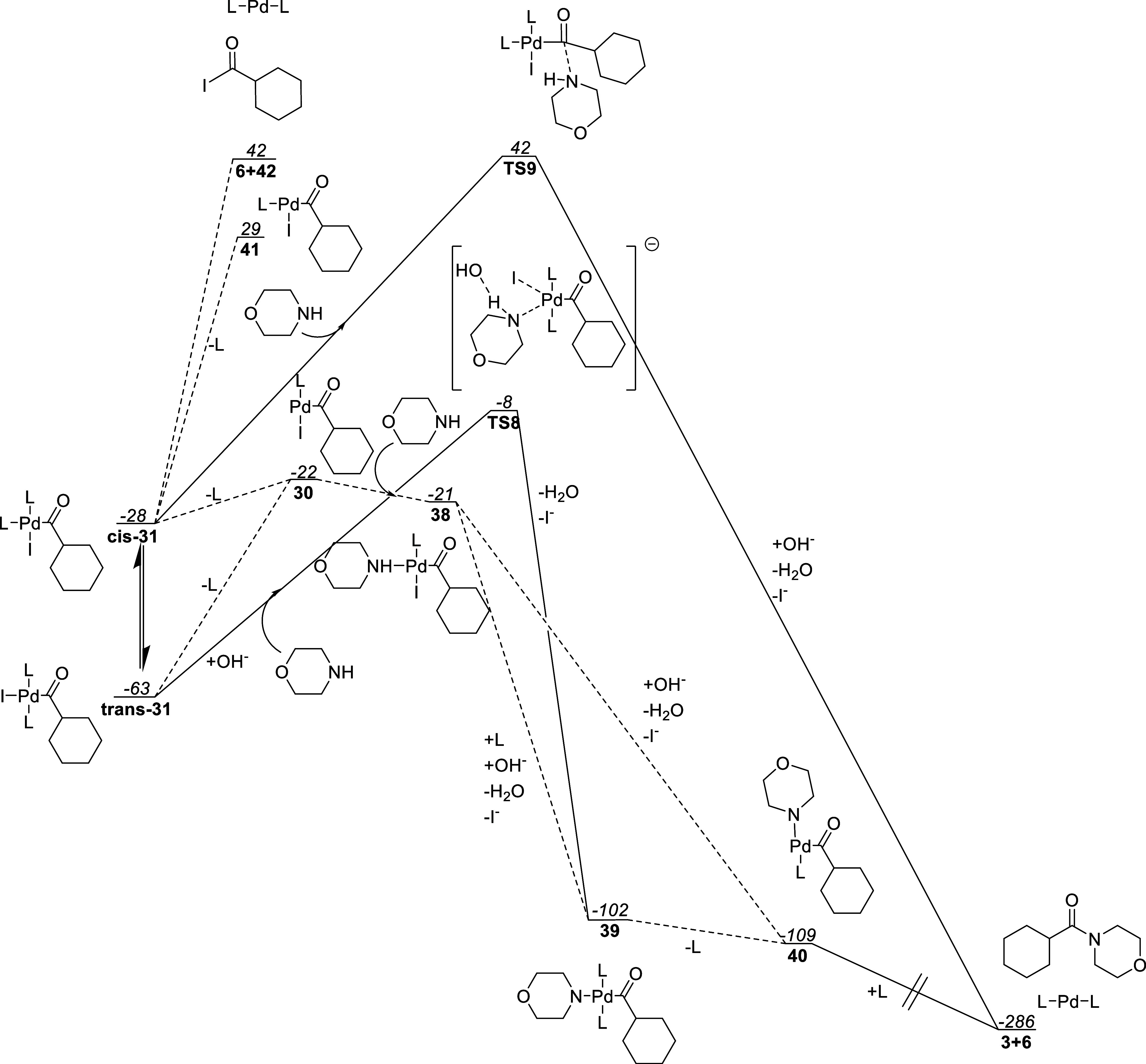
Morpholine binding pathway along with reductive elimination pathway of the amide. Also shown is the alternative reductive elimination acyl iodide **42**. Free energies reported in kJ/mol relative to complex **11-S0** with L = PPh_3_.

The free energy plot of this step is shown in Figure 15. From **cis-31** or **trans-31**, a ligand can dissociate to reform acyl complex **30**. The trans–cis isomerization of the iodide, relative to the acyl group, resulting from ligand dissociation from **trans-31** has been shown to be facile previously for a similar complex.^64^ Additionally, trying to optimize complex **30** with an iodide and acyl group in a trans orientation caused the geometry to collapse to the isomer of **30** shown in Figure 15.

Morpholine can then coordinate to complex **30** to produce morpholinium complex **38**. This complex is then deprotonated, and a ligand is switched for the iodide to yield morpholine complex **39**. This step was done to preserve charge neutrality to avoid solvation model errors. Morpholine complex **39** can then dissociate a ligand to give **40** where the morpholine ligand isomerizes from the trans-to the cis position relative to the acyl group. Alternatively, complex **38**could be deprotonated without accepting a ligand yielding deprotonated complex **40** directly. By binding in a phosphine ligand to complex **40**, a seemingly barrierless reductive elimination then takes place during geometry optimization to yield amide **3** and Bis-ligated complex **6**.

Alternative pathways from the acyl complex **31** were also investigated. The direct dissociation of the ligand cis to the acyl group to yield tris-ligated complex **41** was too endothermic to be viable as was the reductive elimination of acyl iodide **42**. The associative inner sphere morpholine binding via **TS8** and the outer sphere attack via **TS9** were found to possess too large of activation barriers.

### Impact of Irradiation Wavelength on Yield

An investigation of the wavelength of light used to conduct the reaction(Table 2) showed that when using Pd(PPh_3_)_4_ as the catalyst, using a green lamp (525 nm) gives only slightly lower yield compared to the blue lamp (450 nm). However, more of the starting material is recovered in the crude NMR. Hence, the conclusion is made that less alkyl iodide **1** decomposes to byproducts (possibly cyclohexane and cyclohexene) under 525 nm illumination compared to 450 nm.

**Table 2:**
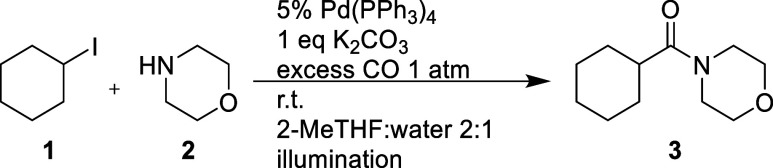
Reaction Dependence on Wavelength and Reaction Time

entry	wavelength (nm)	reaction time (h)	NMR yield (%)	NMR yield SM (%)
1	390	43	46	0
2	450	24	51	11
3	450	43	69	7
4	450	68	69	0
5	525	24	49	18
7	525	96	53	21
8	660	84	35	19

Despite this encouraging result, the yield does not improve with extended reaction time using green light, indicating that the catalyst deactivates faster under green light illumination compared to blue light. Red light (660 nm) illumination gave slow product formation and was therefore not investigated further. In contrast, usage of a 390 nm light gives complete consumption of cyclohexyl iodide **1** at 24 h. However, the product formed is a mixture of amide **3** and carbonyl dimorpholine (**43**) that is undesired in this investigation.

### Impact of Bidentate Ligands

The inclusion of bidentate ligands was explored further (Table 3) after the unexpected increase in reaction yield observed with the dppp ligand, vide supra. We opted to use PdCl_2_ as the catalyst instead since this palladium source does not carry any endogenous phosphine ligands. No significant improvement in yield over dppp (entries 2 and 4) was observed when using dppf (1,1′-Bis(diphenylphosphino)ferrocene), dppe, dppb (1,4-Bis(diphenylphosphino)butane), or Xantphos as the ligand (entries 3 and 5–7).

**Table 3:**
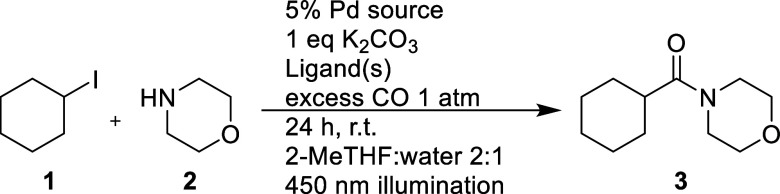
Optimization of the Reaction Conditions^a^

entry	Pd source	ligand(s)	yield (%)
1	Pd(PPh_3_)_4_	none	51, 67^b^, (59^b^)
2	Pd(PPh_3_)_4_	5.5% dppp	76
3	Pd(PPh_3_)_4_	5.5% dppe	56
4	PdCl_2_	5.5% dppp	80
5	PdCl_2_	5.5% dppf	27
6	PdCl_2_	5.5% Xantphos	58
7	PdCl_2_	5% dppb	35
8	PdCl_2_	10% dppp	84
9	PdCl_2_	5% dppp +10% PPh_3_	88
10	PdCl_2_	5% dppp +15% PPh_3_	79
11	PdCl_2_	5% dppp +20% PPh_3_	81, 75
12	Pd(OAc)_2_	5% dppp +15% PPh_3_	66
13	Pd(OAc)_2_	5% dppp +20% PPh_3_	78^c^, (62)^d^ 80^b^,^d^, (59)^b^,^d^
14^e^	PdCl_2_	10% dppp	34, 69^d^
15^e^	PdCl_2_	5% dppp +10% PPh_3_	53

a Isolated yield is shown in parentheses; otherwise, the yield is calculated from qNMR.

b 1 equiv of CO was used.

c 1 h preactivation without light or water phase.

d 30 min preactivation without light or water phase.

e 525 nm Evulochem lamp used.

We attempted to combine the stability of a bidentate palladium complex but retained the accessibility of free coordination sites associated with a monodentate complex. Hence, different mixtures of dppp and PPh_3_ were investigated (Table 3, entries 9–11). All of these conditions performed well regarding yield. However, these reactions did precipitate palladium black, which was judged to be undesirable due to the associated loss of activity.

To counter the precipitation of palladium black during the reaction, the catalyst was replaced with Pd(OAc)_2_ in the hope that the higher solubility of the palladium source would prevent precipitation (entry 12). This was certainly the case but only if the Pd(OAc)_2_ and ligands were dissolved in 2-MeTHF before the water layer was added and the light switched on (entry 13). Thus, it is likely that Pd(OAc)_2_(dppp) forms during the preactivation and then undergoes reduction without resulting in precipitation of unligated Pd^0^ (palladium black). This is not necessarily connected to a higher yield but indicates that a lower palladium loading could be used, if desired. The change is also a practical improvement since it avoids filtering palladium nanoparticles. Green light was also screened with dppp but did not give a competitive yield compared to blue light (entries 14–15).

The substantial increase in yield when switching to dppp (Table 3 entries 2 and 4) shows that a cationic carbonylation pathway is possible with this ligand since the palladium does not have an excess coordination site that conceivably could be exchanged for an iodide in carbonylation complex **36**. This increase in yield made the conditions in entry 13 interesting for further study, and these conditions will hereafter be referred to as the revised reaction conditions.

**Figure 16:**
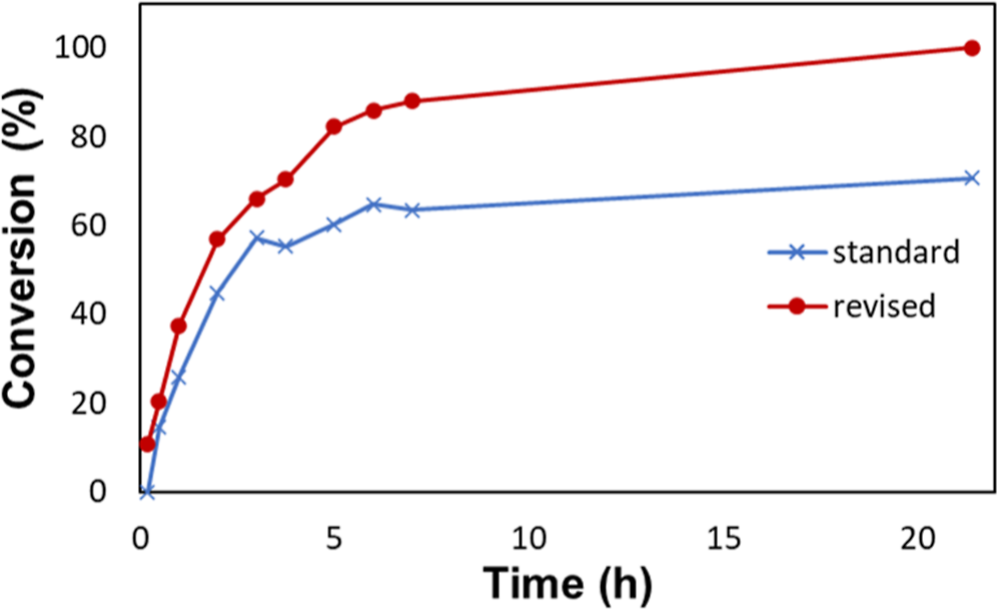
Conversion over time data for the standard and revised conditions as measured by GC–MS.

**Figure 17:**
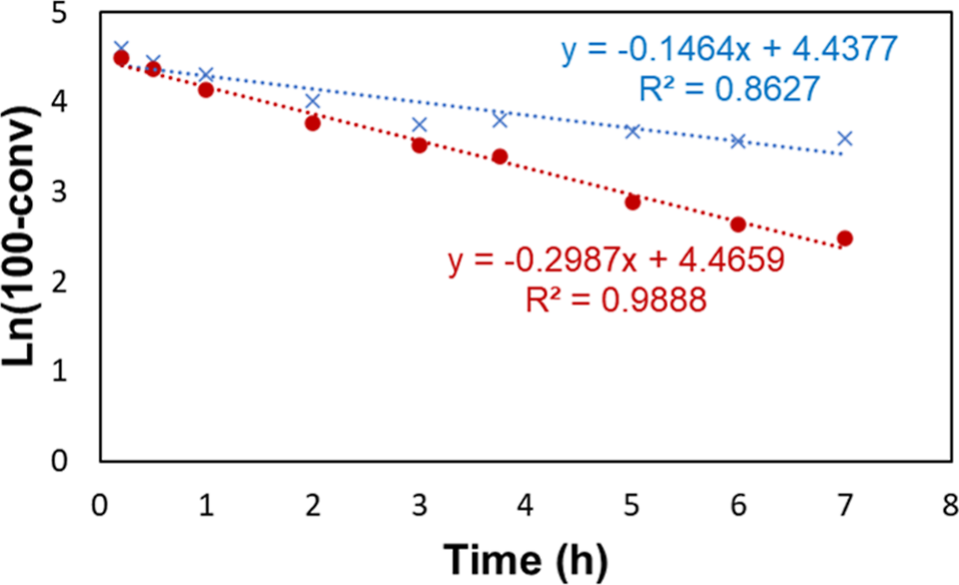
Data shown in Figure 16 is shown, linearized assuming first-order kinetics. The last data point was omitted to not skew the regression.

**Figure 18:**
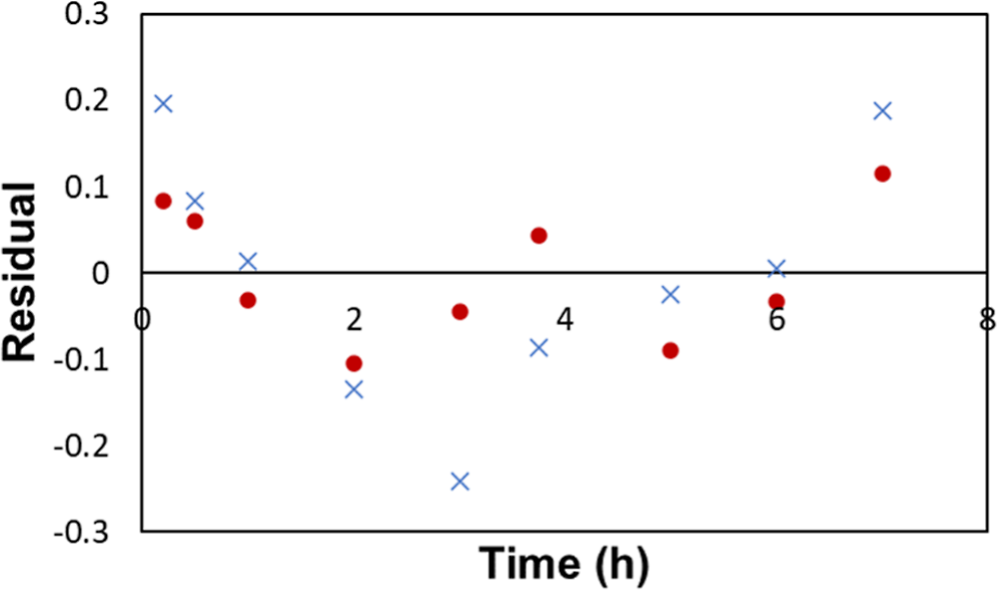
Residual plot of the regression shown in Figure 17.

To further elucidate the difference in kinetics between the standard and revised conditions, the conversion over time for both conditions was monitored by using gas chromatography–mass spectrometry (GC–MS). The data collected in these experiments is shown in Figure 16 with a linear regression assuming first-order kinetics being shown in Figure 17. Looking at the residual plot of the linearized data, shown in Figure 18, we observe that the standard conditions do not obey first-order kinetics well, with the observed rate constant decreasing rapidly around the 3 h mark. This is reinforced by the *R*^2^ of 0.8627 for the linear regression. The revised conditions are well represented by first-order kinetics, as judged by the *R*^2^ of 0.9888 and the residual plot in Figure 18.

Hence, we believe that the yield improvement of the reaction when switching to bidentate ligands is not due to an increase in the rate of the aminocarbonylation. Instead, we propose that bidentate complexes undergo deactivation to a lesser extent than Pd(PPh_3_)_4_, hence the observed first-order kinetics. The revised conditions provide a more synthetically attractive procedure, which was made possible by thorough investigation of the reaction mechanism.

### Summary of the Proposed Catalytic Cycle

**Figure 19:**
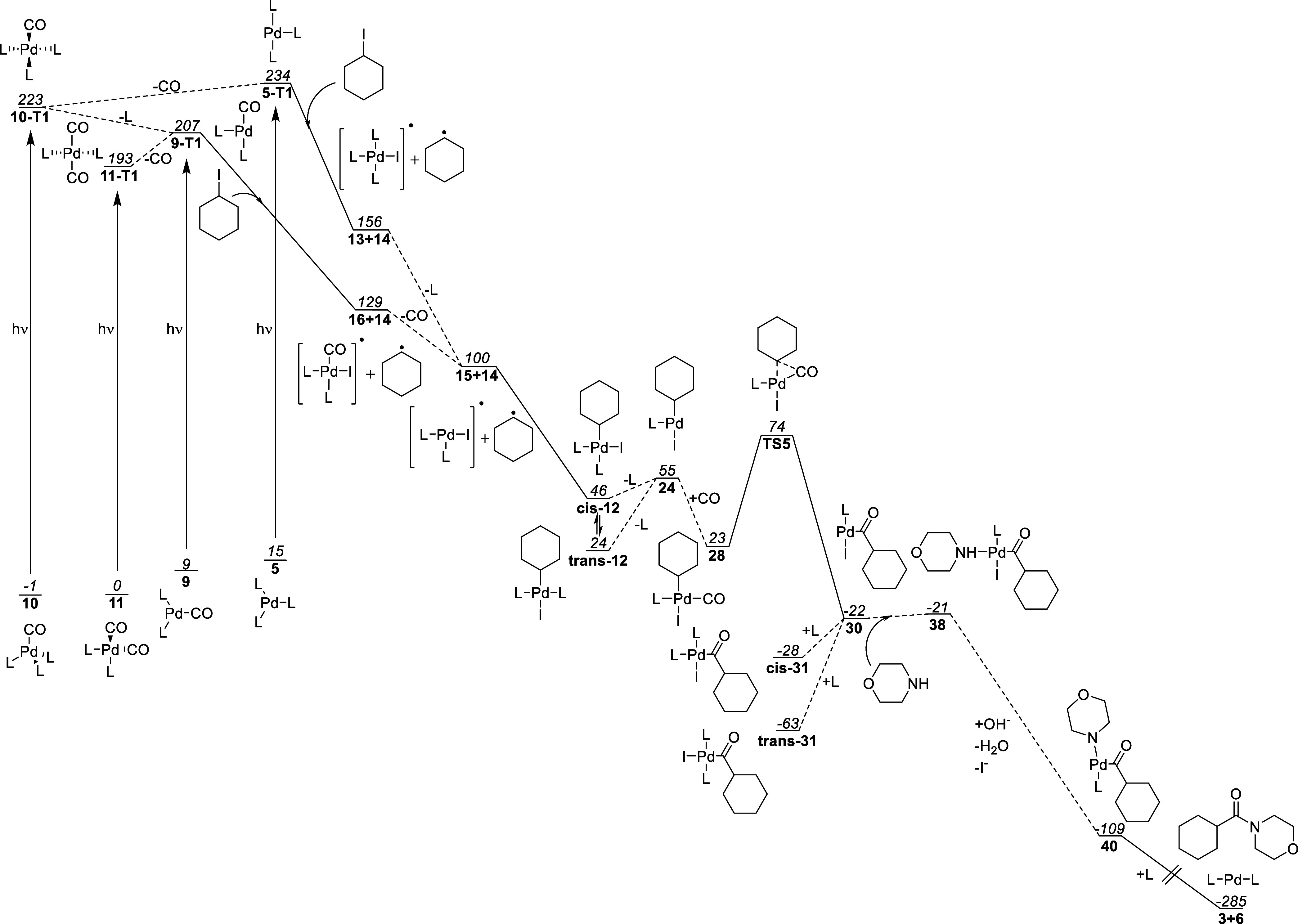
Final calculated free energy plot. Free energies reported in kJ/mol relative to complex **11-S0** with L = PPh_3_.

To summarize the mechanistic insights from our experimental and computational work, we provide a free–energy diagram (Figure 19) of the most likely reaction pathway for the aminocarbonylation reaction. Monocarbonyl **9** is excited to give compound **9-T1**. Alternatively, **5** is directly excited to form **5-T1**. One or both excited complexes can then undergo ATRA to give **cis-12**. This alkyl complex then proceeds through a dissociative ligand exchange via **24**, replacing a phosphine ligand with CO. The resulting carbonyl complex **28** then undergoes a carbonylative insertion via **TS5** to yield tris-ligated complex **30** which can bind to a phosphine ligand to yield acyl complex **trans-31**. This species can then dissociate a phosphine ligand to reform **30** causing the barrierless movement of the iodide into the coordination site cis to the acyl fragment yielding tris-ligated acyl complex **30**.

Morpholine coordinates into the empty coordination site to yield **38**. Deprotonation followed by barrierless movement of the morpholine group to the position cis relative to the acyl group results in **40**. Reassociation of a phosphine ligand results in the seemingly barrierless reductive elimination of the desired product amide **3** and Bis-ligated complex **6** which can associate with CO and phosphine ligands to restart the catalytic cycle.

## Conclusions

The aminocarbonylation reaction presented by Sardana and co-workers has been extensively investigated using computational and experimental methods. The pre-equilibria for palladium(0) in the presence of PPh_3_ and CO ligands in the S0, S1, and T1 states were investigated. This gave a far more complete picture of the ligation states, energies, and geometries available to this catalyst than previously known.

We show that the ATRA mechanism is the most likely reaction mechanism for oxidative addition. This was supported by diastereoselectivity and radical trapping experiments as well as by a computational study. The carbonylation was found to be viable with low barriers via a carbonylative insertion leading to acyl complex **trans-31**, which was also isolated and characterized by SC-XRD. Thus, the carbonylation likely happens on the palladium atom, not as a free radical process as previously suggested.^4,48,49,65,66^

The outer and inner sphere amide formation pathways were also compared, and it was concluded that the inner sphere pathway, proceeding via ligand dissociations, is likely responsible for amide formation. This process is expected to be diffusion limited, at least for the model substrate. This result is in agreement with the study by Wang and co-workers further lending support to this elementary step being responsible for palladium-catalyzed amide formations for both alkyl and aryl substrates.^12^

Taken together, this mechanism suggests that excitation of the catalyst by light is the rate-limiting step as the predicted rate-limiting step (**28** to **TS5**) with an activation energy of 51 kJ/mol yields a half-time of approximately 100 μs, assuming that all reactants are at 1 M concentration and a transmission coefficient of one.

An attempt was also made at improving the reaction based on mechanistic insight, which resulted in a more stable palladium catalyst as well as improved NMR yield of the model substrate.

In conclusion, we have provided a detailed reaction pathway of the palladium-catalyzed photochemical aminocarbonylation reaction between alkyl and aryl iodides. The reaction mechanism presents details of the catalyst complexes from catalyst activation to the reductive elimination of the amide product. We hope this knowledge will be useful to practitioners in the field and future studies of palladium-catalyzed photochemistry.

## Data Availability

The data underlying this study are available in the published article and its Supporting Information.

## Supporting Information Available

Detailed experimental procedures, spectral data, crystal data, as well as computational data (PDF)

## Notes

Safety statement Caution! Carbon monoxide is a toxic and flammable gas. When handled in large quantities, such as balloons, a CO monitor should be worn at all times, and the gas should only be handled inside a fume hood.
