# Supplementary material for: Mechanistic Study of Photochemical Aminocarbonylation of Alkyl Iodides Catalyzed by a Palladium Catalyst Using Experimental and Computational Methods
Source: J Org Chem. 2025 Feb 25;90(10):3544–56. doi: 10.1021/acs.joc.4c02240 (PMC11915365; doi:10.1021/acs.joc.4c02240)
Supplement: Supplementary file 1 — jo4c02240_si_001.pdf [file jo4c02240_si_001.pdf]

Mechanistic Study of a Photochemical Aminocarbonylation of Alkyl Iodides Catalyzed by Palladium – Supplementary Information

Erik N. A. Sundén[1,2,*], Staffan Karlsson[1], Okky Dwichandra Putra[3], Måns Andreasson[1], Charles Elmore[1], Per-Ola Norrby[4], Malvika Sardana[1,*]

[1]Early Chemical Development, Pharmaceutical Sciences, R&D, AstraZeneca, Gothenburg, SE-43183 (Sweden)

[2]Department of Chemistry, Ångström Laboratories, Uppsala University, Box 523, 751 20 Uppsala, Sweden

[3]Early Product Development and Manufacturing, Pharmaceutical Sciences, R&D, AstraZeneca, Gothenburg, SE-43183 (Sweden)

[4]Data Science and Modelling, Pharmaceutical Sciences, R&D, AstraZeneca, Gothenburg, SE-43183 (Sweden)

* Erik N.A. Sundén naeriks@gmail.com
* Malvika Sardana malvika.sardana@astrazeneca.com

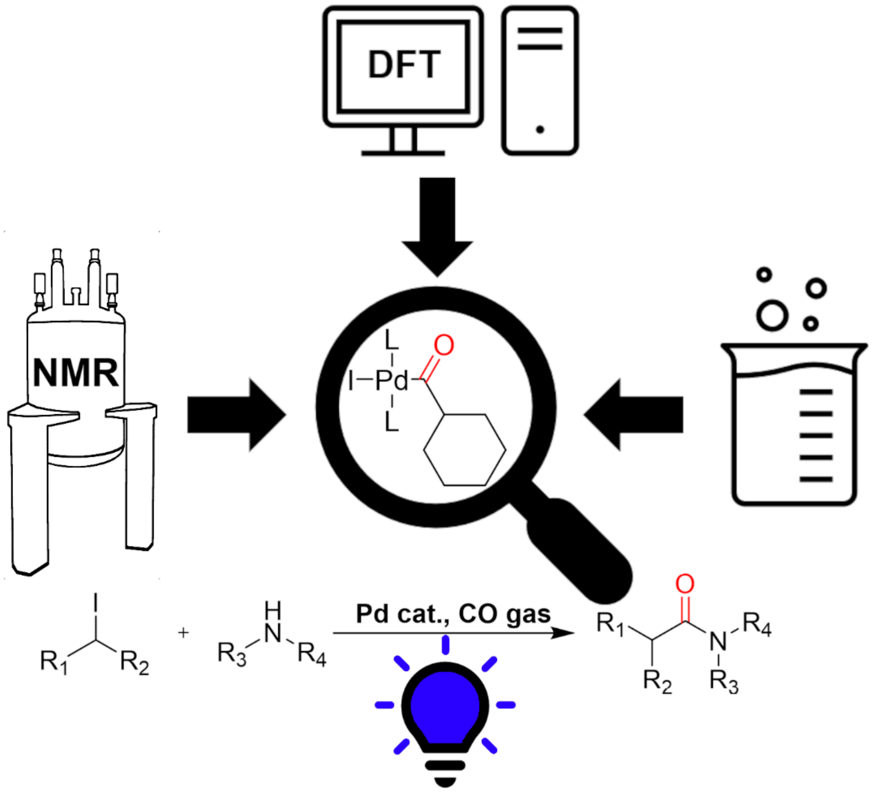

Table of Contents

General information S3

LED report S5

Experimental section S6

Procedure for in situ IR measurement of pre-equilibria S6

Procedure for in situ IR measurement of solvent participation S7

Procedure for flow-NMR measurement without CO at the beginning S7

Procedure for flow-NMR measurement with CO at the beginning. S8

General procedure 1 for synthesis of cyclohexyl(morpholino)methanone (**3**) using a CO balloon (CAS 29338-96-3)[1] S8

General procedure 2 for synthesis of cyclohexyl(morpholino)methanone (**3**) using COgen (CAS 29338-96-3)[1] S8

Synthesis of cis-(4-iodocyclohexyl)benzene (**cis-20**) (CAS 271767-51-2)[4,5] S9

Synthesis of trans-(4-iodocyclohexyl)benzene (**trans-20**) S9

(CAS 2095709-55-8)[4,5] S9

Synthesis of morpholino-(4-phenylcyclohexyl)methanone (**21**) from **cis-20** S10

Synthesis of morpholino-(4-phenylcyclohexyl)methanone (**21**) from **trans-20** S11

Synthesis of 2-diethoxyphosphoryl-2-methyl-5-(4-phenylcyclohexyl)pyrrolidin-1-ium 1-oxide (**22**) S11

Synthesis of 2-dimethyl-5-(4-phenylcyclohexyl)pyrrolidin-1-ium 1-oxide (**23**) S12

Synthesis of Carbonyl dimorpholine (**43**) (CAS 38952-62-4)[6,7] S13

Single crystal X-ray diffraction and structure refinments S13

Computational data S17

Scans S17

Input files for define and cosmoprep S19

Energies of geometries S21

NMR spectra S111

cyclohexyl(morpholino)methanone (**3**) S111

cis-(4-iodocyclohexyl)benzene (**cis-20**) S113

trans-(4-iodocyclohexyl)benzene (**trans-20**) S116

Morpholino-cis-(4-phenylcyclohexyl)methanone (**cis-21**) S118

Morpholino-trans-(4-phenylcyclohexyl)methanone (**trans-21**) S121

Carbonyl dimorpholine (**43**) S123

References S126

General information

All reagents were purchased from commercial vendors and used without further purification unless otherwise specified. Anhydrous solvents were purchased from Sigma-Aldrich in SureSeal bottles and stored under nitrogen. COgen and $Pd(PPh_3)_4$ was stored in nitrogen purged microwave vials between uses. CO gas of instrument grade was purchased from Linde. Solvent evaporation was done on a rotary evaporator with a water bath heated up to 40 °C.

Light sources used in this publication was either the same hovering photoreactor used in the original publication, commercially available Kessil lamps with the wavelength specified in the procedure or a 525 nm 18w EvoluChem LED spotlight. Kessil lamps and the Evulochem lamp were placed ca 5 cm from the reaction vessel. The hovering photoreactor consisted of Blue S6 LED strips (15 V, 15 W/meter, 4.67 m, λ = 465.2 nm) provided by LED Teknik Boras Sweden mounted on a cylindrical mesh.[1] The construction of the hovering photoreactor means that the distance from light to reaction vessel is approximatelly 10 cm. The LED report is provided below.

Experiments were repeated once unless otherwise stated.

Purification was either done by flash chromatography employing a Biotage isolera One running Sfär Duo cartridges or by reverse phase HPLC. The HPLC system was constructed from a Waters 2545 quaternary gradient module, prep degasser, 2707 autosampler, 2489 UV-Visible detector and a fraction collector III running a 19 x 250 mm Xbridge® prep C18 5 µm OBD™ column.

Offline NMR spectra were acquired in $CDCl_3$, C_6D_6, or d_6-DMSO. ^1H and ^{13}C NMR were acquired with Bruker AVANCE III systems at a proton frequency of either 500 or 600 MHz with the former either being equipped with a normal probe or a cryoprobe while the latter was equipped only with cryoprobes. ^{31}P NMR was acquired only on the Bruker AVANCE III equipped with normal probe and a 500 MHz proton frequency yielding a 202.39 MHz ^{31}P frequency. ^1H and ^{13}C NMR were referenced to the residual solvent peak as listed by Fulmer and coworkers.[2] ^{31}P NMR was indirectly referenced to 85% H_3PO_4 via the deuterium lock signal. Signals are listed in ppm, and multiplicity was identified as s = singlet, br = broad, d = doublet, dt = doublet of a triplet, t = triplet, tt = triplet of a triplet, q = quartet, quin= quintet, h = hextet, and m = multiplet; coupling constants in Hz; integration. qNMR was acquired with 1,3,5-trimethoxybenzene. All spectra were processed in MestReNova 14.3.3. Structural assignments were made with additional information from gCOSY, gHSQC, and gHMBC experiments

GC-MS (EI) analysis was performed with an Agilent 7890A GC system with a 5957 inert MSD system. The capillary column used was an Agilent 19091S-433L (30 m x 250 µm x 0.25 µm). The temperature program consisted of 40 °C for 1 min, followed by 40 °C/min for 6 min then 280 °C for 5 min. Helium gas at 2 mL/min was used as the carrier. The injector was configured as a 30:1 split injector at 250 °C using an injection volume of 1 µL with the sample dissolved or diluted in Et_2O. The detector was set to sweep 50-550 da at 3 Hz with 70 eV electron ionization. The quadrupole was held at 150 °C while the MS source was held at 230 °C.

LC-MS and LC-UV was measured on a Waters Aquity UPLC system. Detection was accomplished with a QDA detector running ESI +/- scanning 100-800 da at 5 Hz and a PDA detector running 220-350 nm with 1.2 nm resolution measuring 20 points/s. The gradient used was 0.2 min 10 % MeCN, 3.5 min 10-99 % MeCN, 0.3 min 99 % MeCN using one of the two eluent and column combinations shown below.

1. pH 10 method: MeCN-NH_4/NH_4CO_3:water employing a BEH C18 column (2.1 x 50 mm with 1.7 µm particles)

2. pH 4: MeCN-formic acid:water employing a HSS C18 column (2.1 x 50 mm with 1.8 μm particles).

Crude diastereomeric ratio (d.r.) was measured by LC-UV correcting for the difference in absorbance coefficient by single point calibration against NMR of purified material.
HR-MS was acquired with a Waters Xevo Q-TOF mass spectrometer using positive mode electrospray ionization. For compounds which did not ionize using ESI-MS, GC-MS (EI) was used as a substitute.
Melting points were measured on a Mettler-Toledo DSC3+ using a 5 K/min heating rate with the exception of **trans-21** which was measured at 10 K/min.
Thin layer chromatography (TLC) was done using Merck silica glass plates (60F-254). Visualization was conducted with either UV light (254 nm), iodine vapour or potassium permanganate.
Flow NMR was acquired with a Spinsolve 80 ultra carbon from Magritek with a proton frequency of 80 MHz using a RMK2 glass flow cell equipped with a 4mm ID measurement section. Before every reaction monitoring experiment, the instrument was "powershimmed". During the reaction monitoring experiment, the spectrometer was shimmed to the first order and calibrated on the proton solvent peak and spectra were then acquired with a WET-1D pulse sequence, this process was repeated each measurement during each flow experiment. Number of scans was varied from 4 to 16 depending on the desired time resolution. During processing, spectra were referenced to a residual toluene peak present in the $Pd(PPh_3)_4$ and apodized with a 0.6 Hz gaussian function. Processing was done in Spinsolve 2.3.5.
In situ IR was measured with a React-IR 702L fitted with a AgX fiber and SiComp probe. Sampling was conducted with an 8 cm^{-1} resolution with a measurement window from 2500 to 600 cm^{-1}. Spectra were apodized with the Norton-Beer medium scheme. Background spectra were acquired against air and the spectrometer was operated in the low gain mode for all measurements.

LED report

LED Integrated Testing System test report

test item:	LED spectrum analyse			
equipment:	Led test system			
Test Identifier	Product	Blue S6	Manufacturer:	Ledteknik
	Temperature	23 ° C	Humidity:	30 %
	Conner:	MOL	Date:	20120823
	Organ:	LEDteknik AB		
curve of spectrum power distributing				

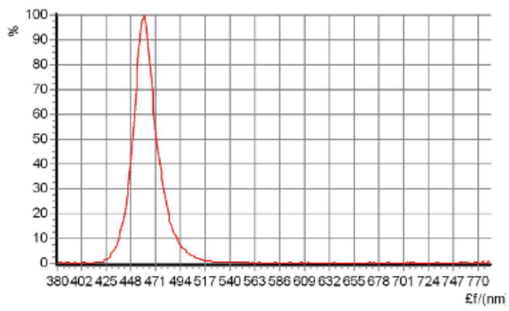

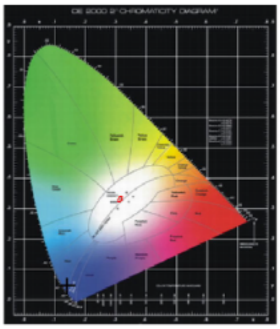

Spectrum		Electrical	
λ (Peak):	460. 4 nm	I(test):	120. 4 mA
λ (Main):	465. 2 nm	V(Positive):	12. 000 V
λ (Centroid):	450. 7 nm	Φv:	33497. 9 mlm
λ (Center):	451. 0 nm	Light Effi:	23. 185 lm/w
BandWidth:	23. 0 nm	Light Power:	554. 4217 mw
ColorTemp:	K		
(x, y):	0. 1418, 0. 0503		
(u, v):	0. 1708, 0. 0909		
Ra:	0. 0		
Color Purity:	0. 966		

Figure S1: Report of the LED:s used to build the hovering photoreactor.[1]

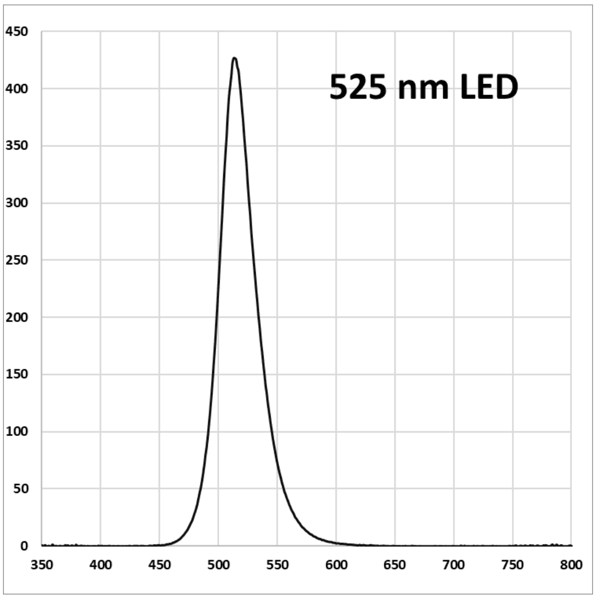

Figure S2: Report for the EvoluChem LED 525PF lamp.[3]

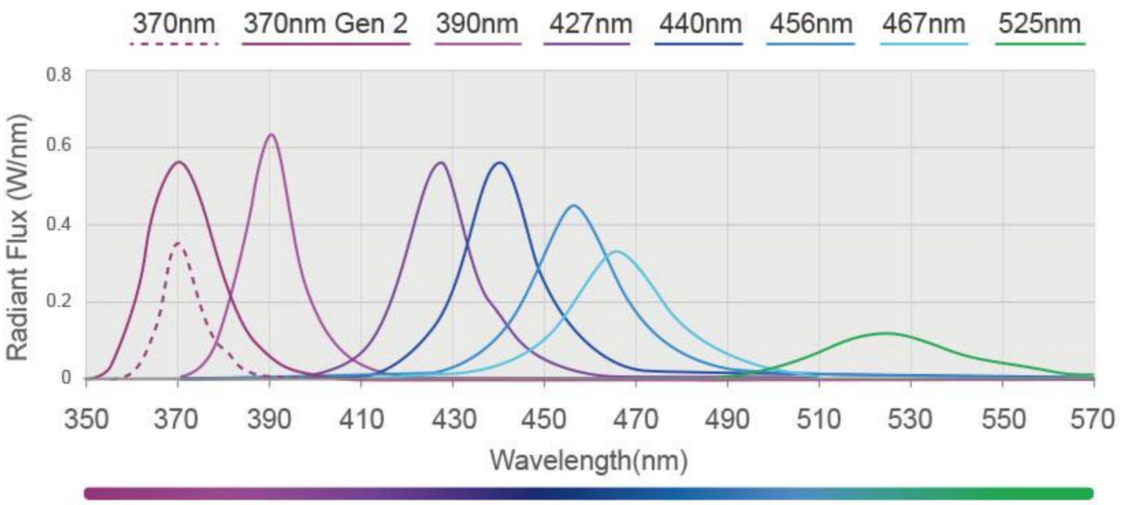

Figure S3: The wavelength specifications for the PR160L line of Kessil lamps used in this work with the exception of the 660 nm lamp.[4]

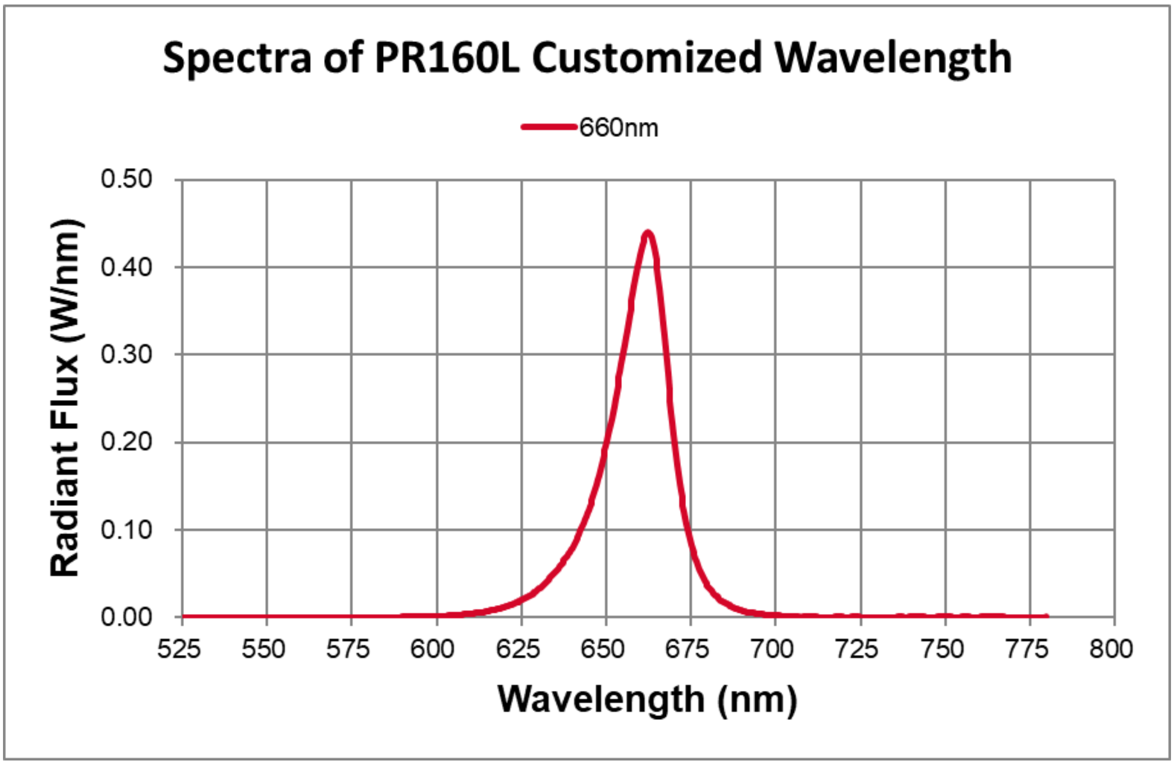

Figure S4: Emission spectra for the PR160-L 660 nm lamp.[5]

Experimental section

Procedure for in situ IR measurement of pre-equilibria

A 50 mL three necked round bottom flask was equipped with an IR probe and flushed with N_2. 2-MeTHF (25 mL) was added along with $Pd(PPh_3)_4$ (1 g, 0.87 mmol). The flask was then flushed CO gas from a balloon. At marker 8 in Figure S5 a CO balloon was connected to the flask causing first a peak at 1965 cm^{-1}, then a second peak at 2020 cm^{-1} to evolve. At marker 17, the flask was purged with N2 gas causing the aforementioned

peaks to disappear. At marker 18 CO was reconnected causing the reemergence of the peaks.

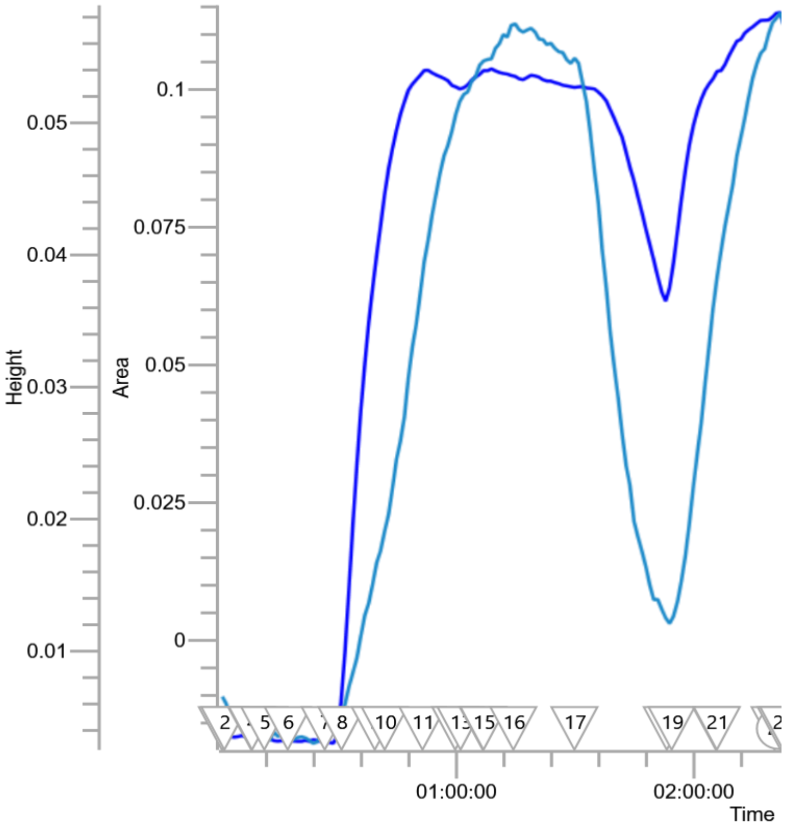

Trend	Color	Units
Peak at 1965 cm-1		Height
Peak at 2020 cm-1		Area

Figure S5: Here the raw data from the pre-equilibrium IR experiment is shown

Procedure for in situ IR measurement of solvent participation

A 100 mL three necked round bottom flask was equipped with an IR probe and flushed with N_2. 1,2-dichloroethane (5 mL) was added along with $Pd(PPh_3)_4$ (550 mg, 0.48 mmol). The flask was then flushed CO gas from a balloon. The development of signals in the carbonyl region was then monitored until they reached equilibria. 2-MeTHF (25 mL) was then titrated in portions starting at 50 μL, monitoring for signal shifts in the carbonyl region.

Procedure for flow-NMR measurement without CO at the beginning.

To a 100 mL round bottom flask equipped with a stir bar was added $Pd(PPh_3)_4$ (1.00 g, 0.87 mmol) and benzene (10 mL). The flask was flushed with N_2 and the measurements were started. Iodocylohexane (0.10 mL, 0.77 mmol) was added and after the signal reached equilibria, a 450 nm Kessil lamp was switched on at the highest intensity for 20 min. The lamp was then switched off for 50 min after which it was turned back on. Measurements in this way was continued overnight. The illumination was seized, and

the flask flushed with CO after which it was left to stir in the dark for 200 min. Illumination was then turned on for 100 min after which the experiment was aborted.

Procedure for flow-NMR measurement with CO at the beginning.

To a 50 mL round bottom flask equipped with a stir bar was added $Pd(PPh_3)_4$ (370 mg, 0.32 mmol) and benzene (10 mL). The flask was flushed with N_2 and the measurements were started. The flask was flushed with CO and left to equilibrate for 1.5 h. Iodocyclohexane (41 µL, 0.32 mmol) was then added and once again left to equilibrate for 40 min. A 450 nm Kessil lamp was then switched on and the reaction was illuminated overnight after which the measurements were aborted.

*General procedure 1 for synthesis of cyclohexyl(morpholino)methanone (**3**) using a CO balloon (CAS 29338-96-3)*[1]

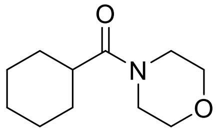

To a 5 mL microwave vial was added the Pd source (5.0 %, 0.030 mmol) and K_2CO_3 (1 equiv., 0.60 mmol) and ligand if used. 2-MeTHF (3.5 mL) and water (1.5 mL) was added followed by iodocyclohexane (1 equiv., 0.60 mmol) and morpholine (3 equiv., 1.80 mmol). The solution was bubbled with N_2 following which the vial was capped. A CO balloon was connected to the vial and used to flush the tube.
The flask was illuminated with the specified lamp for the specified time while cooling with a 120 mm fan. The reaction mixture was extracted over a phase separator with CH_2Cl_2 (3 x 10 mL). The organic layer was concentrated in vacuo yielding the crude as a yellow oil which was analysed by qNMR to determine NMR yield unless stated otherwise.
TLC (EtOAc) R_f = 0.44
^{1}H NMR (500 MHz, $CDCl_3$) δ 3.65 – 3.60 (m, 4H), 3.60 – 3.55 (m, 2H), 3.48 – 3.44 (m, 2H), 2.40 (tt, J = 11.6, 3.4 Hz, 1H), 1.77 (dt, J = 6.0, 3.2 Hz, 2H), 1.70 – 1.68 (m, 1H), 1.67 – 1.65 (m, 2H), 1.58 – 1.44 (m, 2H), 1.31 – 1.16 (m, 3H).
$^{13}C\{^1H\}$ NMR (126 MHz, $CDCl_3$) δ 174.8, 67.1, 67.0, 46.0, 42.0, 40.3, 29.4, 25.9, 25.9.
GC-MS (EI) m/z: $[M]^+$ Calcd for $C_{11}H_{19}NO_2$ 197.1; Found 197.1.

*General procedure 2 for synthesis of cyclohexyl(morpholino)methanone (**3**) using COgen (CAS 29338-96-3)*[1]

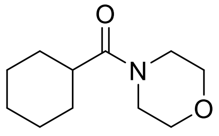

For this procedure a COware two chamber system was used.[6]

COgen chamber

To the Cogen chamber was added $Pd(dba)_2$ (5.0 %, 0.030 mmol), toluene (3.0 ml). The solution was bubbled with N_2 while $P(t\text{-}Bu)_3$ (5 %, 0.03 mmol) and DIPEA (1.5 equiv., 0.92 mmol) was added. The chamber was sealed with a PTFE septa and a stabilizing disc. Before illumination was started in the reaction chamber, COgen in toluene (2.00 mL, 0.3 M) was added and the chamber was heated to 70 °C.

Reaction chamber

To the reaction chamber was added the Pd source (5.0 %, 0.030 mmol) and K_2CO_3 (1 equiv., 0.6 mmol). 2-MeTHF (3.5 mL) was added and purging with N_2 was started. Iodocyclohexane (1 equiv., 0.60 mmol) and morpholine (3 equiv., 1.80 mmol) was added and the chamber is sealed with a PTFE septa and a stabilizing disc. The reaction was illuminated with 450 nm light from the hovering photoreactor for 24 h while cooling with a 120 mm fan. The reaction mixture was extracted with CH_2Cl_2 (3 x 10 mL) over a phase separator and solvent was removed under reduced pressure. Purification was performed using a flash chromatography yielding a white crystalline solid.
For analytical data, *vide supra*.

*Synthesis of cis-(4-iodocyclohexyl)benzene (**cis-20**) (CAS 271767-51-2)*[7,8]

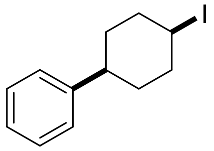

To a 20 mL microwave vial was added PPh_3 (624.7 mg, 2.38 mmol), imidazole (169.7 mg, 2.49 mmol) and CH_2Cl_2 (4.5 mL). The resulting mixture was allowed to stir for 5 min to dissolve before being cooled to 0 °C. Iodine (601.6 mg, 2.37 mmol) was then added in three portions over 10 min to the stirred solution after which trans-4-phenylcyclohexan-1-ol (352.1 mg, 2.00 mmol) was added. The vial was capped and flushed with N_2. The walls of the vial were washed down with CH_2Cl_2 (0.5 mL) with a syringe.
The reaction was left to stir for 22 h at r.t. Heptane (10 mL) was added, the reaction was filtered and concentrated under reduced pressure yielding the crude as an off-white flaky solid.
The crude was dry-loaded on silica and purified by flash column chromatography on a 10 g sfär duo cartridge with heptane. Pure fractions were pooled, and solvent was removed under reduced pressure yielding the product cis-4-iodocyclohexyl)benzene (261 mg, 45.7 %) as a white solid with a 99:1 d.r. cis:trans.
TLC (heptane): R_f = 0.28
^{1}H NMR (500 MHz, $CDCl_3$, 25°C) δ 7.38 – 7.32 (m, 2H), 7.33 – 7.27 (m, 2H), 7.27 – 7.21 (m, 1H), 4.96 (p, J = 3.3 Hz, 1H), 2.61 (tt, J = 12.1, 3.7 Hz, 1H), 2.27 – 2.17 (m, 2H), 2.07 (qd, J = 12.4, 3.2 Hz, 2H), 1.84 – 1.77 (m, 2H), 1.72 (ddt, J = 15.6, 12.3, 3.5 Hz, 2H).
^{13}C{^{1}H} NMR (126 MHz, $CDCl_3$) δ 146.8, 128.6, 127.0, 126.3, 43.9, 36.7, 36.6, 36.2, 30.3, 30.2, 30.1.
GC-MS (EI) m/z: $[M]^+$ Calcd for $C_{12}H_{15}I$ 286.2; found 286.0.
MP: 63-66 °C

*Synthesis of trans-(4-iodocyclohexyl)benzene (**trans-20**) (CAS 2095709-55-8)*[7,8]

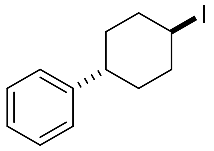

To a 20 mL microwave vial was added triphenylphosphine (638.3 mg, 2.43 mmol), imidazole (171.0 mg, 2.51 mmol) and CH_2Cl_2 (4.5 mL). The resulting mixture was allowed to stir for 5 min to dissolve before being cooled to 0 °C. Iodine (604.2 mg, 2.38

mmol) was then added in three portions over 10 min to the stirred solution after which cis-4-phenylcyclohexan-1-ol (355.4 mg, 2.02 mmol) was added, the vial capped, and flushed with N_2. The walls of the vial were washed with CH_2Cl_2 (0.5 mL).
The reaction was left to stir at r.t. for 22 h. Heptane (10 mL) was added, the reaction was filtered and concentrated under reduced pressure yielding the crude as a white oily solid.
The crude was dry-loaded on silica and purified by flash column chromatography on a 10g sfär duo cartridge with heptane. Product containing fractions were pooled and solvent was removed under reduced pressure yielding the purified product.
The product was again dry-loaded on silica and purified by flash column chromatography on a 10g sfär duo cartridge with heptane. Pure fractions were pooled, and solvent was removed under reduced pressure yielding the desired product (76.8 mg, 13 %) as a white solid with a d.r. 87:13 trans:cis.
TLC (heptane): R_f = 0.21
^{1}H NMR (500 MHz, $CDCl_3$, 25°C) 7.36 – 7.29 (m, 2H), 7.28 – 7.21 (m, 1H), 7.24 – 7.18 (m, 2H), 4.23 (tt, J = 12.3, 4.1 Hz, 1H), 2.65 (tt, J = 12.2, 3.5 Hz, 1H), 2.62 – 2.54 (m, 2H), 2.24 – 2.12 (m, 2H), 1.88 – 1.80 (m, 2H), 1.60 (qd, J = 13.3, 3.2 Hz, 2H).
^{13}C{^{1}H} NMR (126 MHz, $CDCl_3$) δ 146.3, 128.6, 126.8, 126.3, 42.9, 40.9, 36.7, 29.1.
GC-MS (EI) m/z: $[M]^+$ Calcd for $C_{12}H_{15}I$ 286.2; Found 286.1.
MP: 54-61 °C

*Synthesis of morpholino-(4-phenylcyclohexyl)methanone (**21**) from **cis-20***

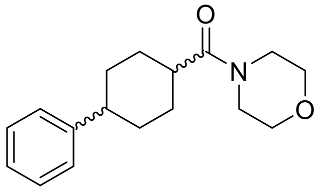

To a 5 mL microwave vial was added $Pd(PPh_3)_4$ (15.8 mg, 0.01 mmol) and potassium carbonate (40.4 mg, 0.29 mmol). 2-methyl tetrahydrofuran (1.75 mL) and water (0.75 mL) was added followed by cis-4-iodocyclohexyl)benzene (80.0 mg, 0.28 mmol). The solution was bubbled with N_2 and morpholine (72 µL, 0,83 mmol) was added following which the vial was capped. A CO balloon was connected to the vial and used to flush the tube causing the solution to turn red.
The vial was irradiated with a 450 nm Kessil lamp while stirring at r.t. cooled by a fan for 23 h. The reaction mixture was extracted over a phase separator using CH_2Cl_2 (3 x 2 mL). Solvent was removed under reduced pressure leaving the crude as a yellow solid with a d.r. of 40:60 trans:cis measured by LC-UV at 260 nm.
The crude was dry loaded on celite from CH_2Cl_2 and purified on a 10g Sfär Duo cartridge running heptane:EtOAc, 2 CV 25%, 2 CV 25-40%, 10 CV 40-60%. Product containing fractions were pooled and solvent was removed under reduced pressure.
Diastereomerically pure fractions were pooled separately. Solvent was removed yielding off white solids.
All material was then pooled yielding a colourless oil (35,5 mg, 0,130 mmol, 46,4 %) and the d.r. was determined to 39:61 trans:cis by NMR.
TLC (1:1 heptane:EtOAc) R_f = 0.18 (trans-product): 0.28 (cis-product)
Morpholino-cis-(4-phenylcyclohexyl)methanone
^{1}H NMR (500 MHz, $CDCl_3$, 25°C) δ 7.27 (d, J = 5.9 Hz, 4H), 7.20 – 7.13 (m, 1H), 3.71 –

3.65 (m, 4H), 3.61 (br s, 2H), 3.52 (br s, 2H), 2.81 (p, J = 4.6 Hz, 1H), 2.62 (tt, J = 10.7, 3.8 Hz, 1H), 2.09 (dtd, J = 13.2, 10.7, 3.6 Hz, 2H), 1.96 (dq, J = 13.7, 4.2 Hz, 2H), 1.75 (dq, J = 13.2, 4.2 Hz, 2H), 1.72 – 1.62 (m, 2H).
$^{13}C\{^{1}H\}$ NMR (126 MHz, $CDCl_3$) δ 174.6, 147.0, 128.4, 127.3, 126.0, 67.1, 67.0, 46.5, 42.8, 42.0, 35.2, 30.0, 27.7.
HR-MS (ESI-QTOF) m/z: $[M + H]^+$ Calcd for $C_{17}H_{24}NO_2$ 274.1802; Found 274.1812.
MP: 118-121 °C
Morpholino-trans-(4-phenylcyclohexyl)methanone
^{1}H NMR (500 MHz, $CDCl_3$, 25°C) δ 7.33 – 7.28 (m, 2H), 7.22 – 7.19 (m, 3H), 3.72 – 3.66 (m, 4H), 3.63 (br s, 2H), 3.56 (br s, 2H), 2.58 (tt, J = 12.1, 3.5 Hz, 1H), 2.53 (tt, J = 11.8, 3.6 Hz, 1H), 2.04 – 1.96 (m, 2H), 1.91 – 1.83 (m, 2H), 1.76 (qd, J = 13.5, 3.5 Hz, 2H), 1.50 (qd, J = 13.0, 3.5 Hz, 2H).
$^{13}C\{^{1}H\}$ NMR (126 MHz, $CDCl_3$) δ 174.6, 147.0, 128.5, 126.9, 126.2, 67.1, 46.1, 43.7, 42.3, 40.0, 33.6, 29.7.
HR-MS (ESI-QTOF) m/z: $[M + H]^+$ Calcd for $C_{17}H_{24}NO_2$ 274.1802; Found 274.1809.
MP: 147-149 °C

*Synthesis of morpholino-(4-phenylcyclohexyl)methanone (**21**) from **trans-20***

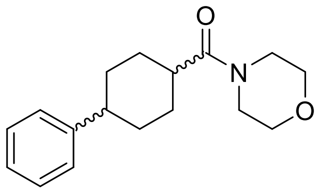

To a 5 mL microwave vial was added $Pd(PPh_3)_4$ (12.5 mg, 10.82 μmol) and potassium carbonate (28.98 mg, 0.21 mmol). 2-methyl tetrahydrofuran (1.75 mL) and water (0.75 mL) was added followed by trans-4-iodocyclohexyl)benzene (61.0 mg, 0.21 mmol). The solution was bubbled with N_2 and morpholine (54 μL, 0.63 mmol) was added following which the vial was capped. A CO balloon was connected to the vial and used to flush the tube causing the solution to turn red.
The vial was irradiated with a 450 nm Kessil lamp while stirring at r.t. cooled by a fan for 23 h. The reaction mixture was extracted over a phase separator using CH_2Cl_2 (3 x 2 mL). Solvent was removed under reduced pressure leaving the crude as a yellow solid with a d.r. of 40:60 trans:cis measured by LC-UV at 260 nm.
The crude was dry loaded on Celite from CH_2Cl_2 and purified on a 10g Sfär Duo cartridge running heptane:EtOAc, 1 CV 25%, 2 CV 25-40%, 5 CV 40-50%, 3 CV 50%, 5 CV 50-60% Product containing fractions were pooled and solvent was removed under reduced pressure yielding the product as a yellow solid (18.0 mg, 0.066 mmol, 30.9 %) with a d.r. of 36:64 by NMR.
TLC (1:1 heptane:EtOAc) R_f = 0,18 (trans-product): 0,28 (cis-product)
For analytical data, *vide supra.*

*Synthesis of 2-diethoxyphosphoryl-2-methyl-5-(4-phenylcyclohexyl)pyrrolidin-1-ium 1-oxide (**22**)*

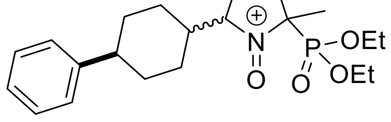

To a 5 mL microwave vial was added DEPMPO (20.6 mg, 0.09 mmol), cis-4-(iodocyclohexyl)benzene (23.5 mg, 0.08 mmol), $Pd(PPh_3)_4$ (4.8 mg, 4.15 µmol) and morpholine (21 µL, 0.24 mmol). This was dissolved in 2-methyltetrahydrofuran (1 mL).

The solution was bubbled for 2 min with N_2 before the vial was capped. A CO balloon was connected, and the vial flushed with the new gas. The vial was illuminated with a 450 nm Kessil lamp overnight, stirring at room temperature, while cooling with a fan. After 17 h 1 mL 2M HCl was added to the reaction mixture which was then extracted over a phase separator with CH_2Cl_2 (3 x 2 mL). Solvent was removed under reduced pressure yielding the crude product.

The product was purified by HPLC running a 25-95% gradient water+0.1% TFA:MeCN collecting at 238 nm. Product containing fractions were pooled, acetonitrile was removed under reduced pressure and the resulting suspension was freeze dried yielding the purified product contaminated with triphenylphosphine oxide (2 mg, 5 nmol, 6 %) as white needle like crystals.

HR-MS (ESI-QTOF) m/z: $[M]^+$ Calcd for $C_{21}H_{33}NO_4P^+$ 394.2142; Found 394.2161.

*Synthesis of 2-dimethyl-5-(4-phenylcyclohexyl)pyrrolidin-1-ium 1-oxide (**23**)*

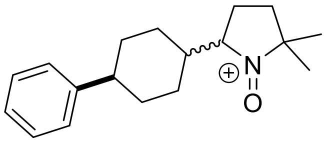

To a 5 MW vial was added DMPO (44,4 mg, 0,39 mmol), cis-4-(iodocyclohexyl)benzene (99 mg, 0,35 mmol), $Pd(PPh_3)_4$ (22,67 mg, 0,02 mmol), potassium carbonate (54,7 mg, 0,40 mmol) and morpholine (102 µL, 1,18 mmol). This was dissolved in 2-methyltetrahydrofuran (3 mL).

The solution was bubbled for 2 min with N2 before the vial was capped. A CO balloon was connected, and the vial flushed with the new gas. The vial was illuminated with a 450 nm Kessil lamp overnight, stirring at room temperature while cooling with a fan. After 23h the reaction was worked up by adding 2 mL 2M ascorbic acid solution to the reaction mixture, letting it stir for 10 min then extracting over a phase separator with CH_2Cl_2 (3x2 mL).

Solvent was removed under reduced pressure yielding the crude (160,3 mg) as a colourless oil with yellow solid in it. The product was dissolved in DMSO, filtered and purified by HPLC running a 5-95% gradient of H_2O+0,2% NH_4OH:MeCN collecting at 232 nm. Solvent was removed under reduced pressure and the resulting water phase was extracted with CH_2Cl_2 (3x5 mL) over a phase separator.

Solvent was removed under reduced pressure yielding the purified product contaminated with triphenylphosphine oxide (4 mg, 0.01 mmol, 4 %) as white needle like crystals.

HR-MS (ESI-QTOF) m/z: $[M]^+$ Calcd for $C_{18}H_{26}NO^+$ 272.2009; Found 272.2021.

*Synthesis of trans-cyclohexanecarbonylbis(triphenylphosphanyl)palladium(II) iodide (**trans-31**)*

Compound was prepared by following "Procedure for flow-NMR measurement with CO at the beginning" sampling 1 ml after 1 hour of illumination. The sample solution was added to a 5 mm NMR tube and frozen in liquid nitrogen under a CO atmosphere. Pentane was layered into the NMR tube which was allowed to stand at room temperature for four days. The resulting red crystals were analysed by SC-XRD, *vide infra*.

*Synthesis of Carbonyl dimorpholine (**43**) (CAS 38952-62-4)*[9,10]

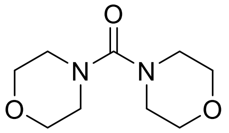

Compound was made according to general procedure 1 using a 390 nm Kessil lamp and $Pd(PPh_3)_4$. The crude was attained as an orange oil, NMR yield was taken as 0.12 mmol, 20%.
The crude was dry loaded on Celite and purified on a 5g Sfär cartridge using 10CV 20%, 10 CV 20-60%, 5 CV 60%, 5 CV 60-100%, 5CV 100% EtOAc in Heptane.
Fractions containing pure compound were isolated and subjected to NMR.
Yield was not noted since compound **43** was produced as an impurity.
TLC (EtOAc): R_f = 0.20
^{1}H NMR (500 MHz, $CDCl_3$, 25°C) δ 3.73 – 3.65 (m, 8H), 3.30 – 3.25 (m, 8H).
^{13}C{^{1}H} NMR (126 MHz, $CDCl_3$) δ 66.8, 47.4.
GC-MS (EI) m/z: $[M]^+$ Calcd for $C_9H_{16}N_2O_3$ 200.2; Found 200.1.

Single crystal X-ray diffraction and structure refinments

Single crystals of compound **trans-31** were grown from a mixture of benzene and pentane by solvent layering. Suitable single crystals were mounted on an XtaLab Synergy-S diffractometer (Rigaku, Japan) equipped with a HyPix-Arc 100 curve detector (Rigaku, Japan) and an Oxford Cryostream 800 (Oxford Cryosystem, UK). Data on two different single crystals were measured using Cu $K\alpha$ and Mo $K\alpha$ radiation, respectively, generated from a micro focus sealed tube (50 kV, 1 mA) at 100K. Measurement strategy was calculated using CrysAlisPro software 1.171.42.35a.[11] Data reduction and correction were performed using CrysAlisPro software[11] where numerical absorption correction based on gaussian integration over a multifaceted crystal model and empirical absorption correction using spherical harmonics, implemented in SCALE3 ABSPACK scaling algorithm were used. The structure was solved with ShelXT[12,13] structure solution program using the direct methods solution method within Olex2[14]. The model was refined on F_o^2 with ShelXL 2014.[12] All non-hydrogen atoms were refined anisotropically. All hydrogen atoms were determined geometrically and refined isotropically. CCDC 2362205 and 2362206 contains the supplementary crystallographic data for this paper. These data can be obtained free of charge via www.ccdc.cam.ac.uk/data_request/cif, or by emailing data_request@ccdc.cam.ac.uk, or by contacting The Cambridge Crystallographic Data Centre, 12 Union Road, Cambridge CB2 1EZ, UK; fax: +44 1223 336033.

Table S1 Crystallographic details of compound **trans-31**

Parameter		
Formula	$C_{49}H_{47}IOP_2Pd$	$C_{49}H_{47}IOP_2Pd$
$D_{calc.}$/ g cm^{-3}	1.491	1.488
m/mm^{-1}	10.280	1.281
Formula Weight	947.10	947.10
Colour	clear red	clear red
Shape	block-shaped	block-shaped
Size/mm^3	0.21×0.07×0.06	0.20×0.09×0.07
T/K	100(2)	100.00(2)
Crystal System	monoclinic	monoclinic
Space Group	$P2_1/c$	$P2_1/c$
a/Å	14.96250(10)	14.9843(2)
b/Å	11.52700(10)	11.52890(10)
c/Å	24.4605(2)	24.4734(2)
b/°	91.2130(10)	91.2670(10)
V/Å^3	4217.82(6)	4226.81(8)
Z; *Z'*	4; 1	4; 1
Wavelength/Å	1.54184	0.71073
θ_{min}/°	2.954	1.953
θ_{max}/°	74.139	29.997
Measured Reflections	55819	79540
Independent Reflections	8348	12311
Reflections I≥2σ(I)	8020	11027
R_{int}	0.0375	0.0287
Parameters	542	545
Restraints	182	188
Largest Peak	0.821	1.108
Deepest Hole	-1.093	-0.848
GooF	1.033	1.028
wR_2 (all data)	0.0766	0.0838
wR_2	0.0760	0.0816
R_1 (all data)	0.0295	0.0346
R_1	0.0285	0.0301
Deposit number	2362205	2362206

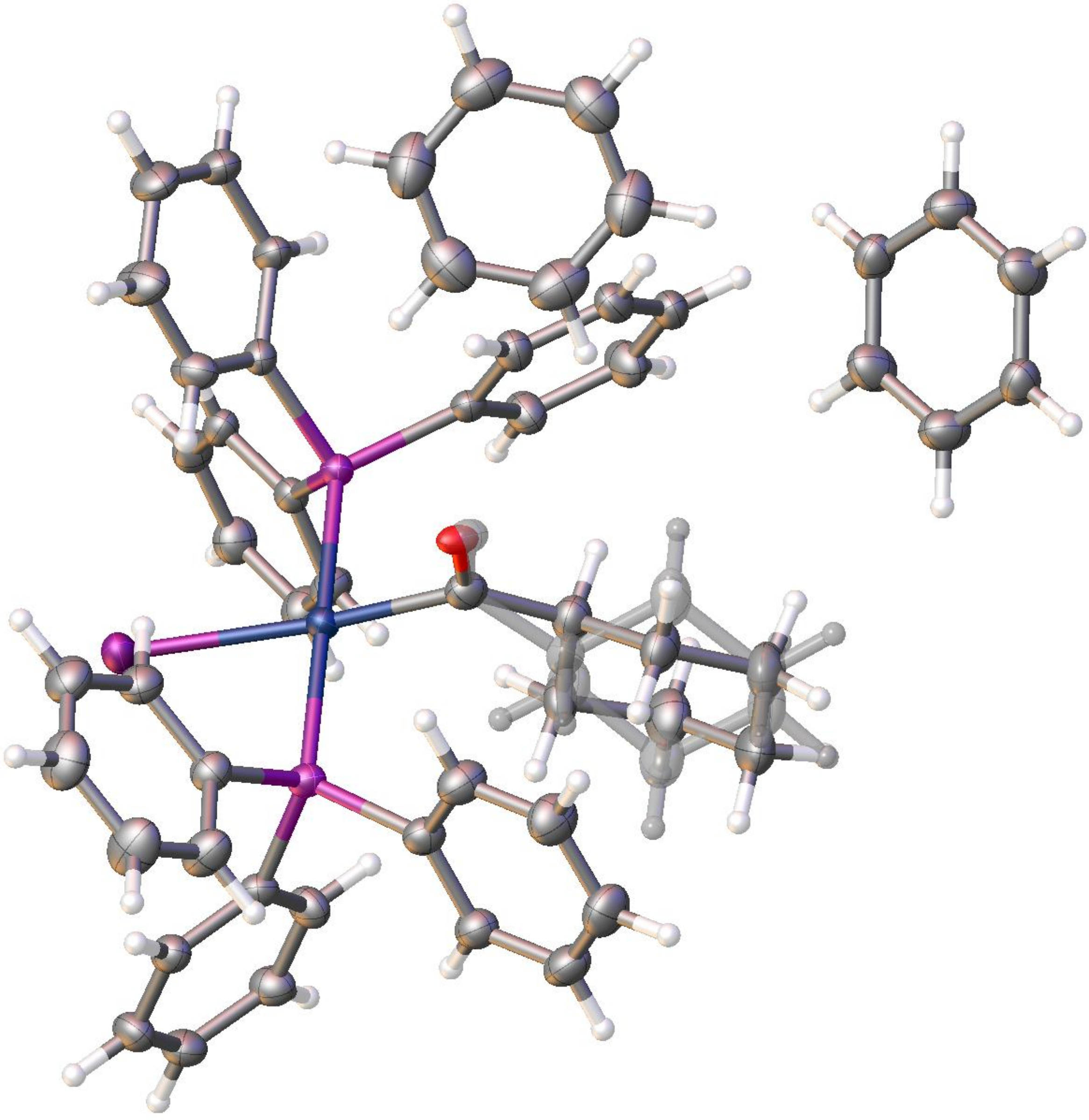

Figure S6: Thermal ellipsoid drawing of compound **trans-31** drawn at 50% probability level. This crystal structure was determined using Cu Kα radiation. The minor part of disorder is depicted as a semi-transparent layer over the major part of disorder. The ratio between major and minor part of disordered were refined at 0.522:0.478(12). Benzene molecules are drawn after grown by symmetry operations.

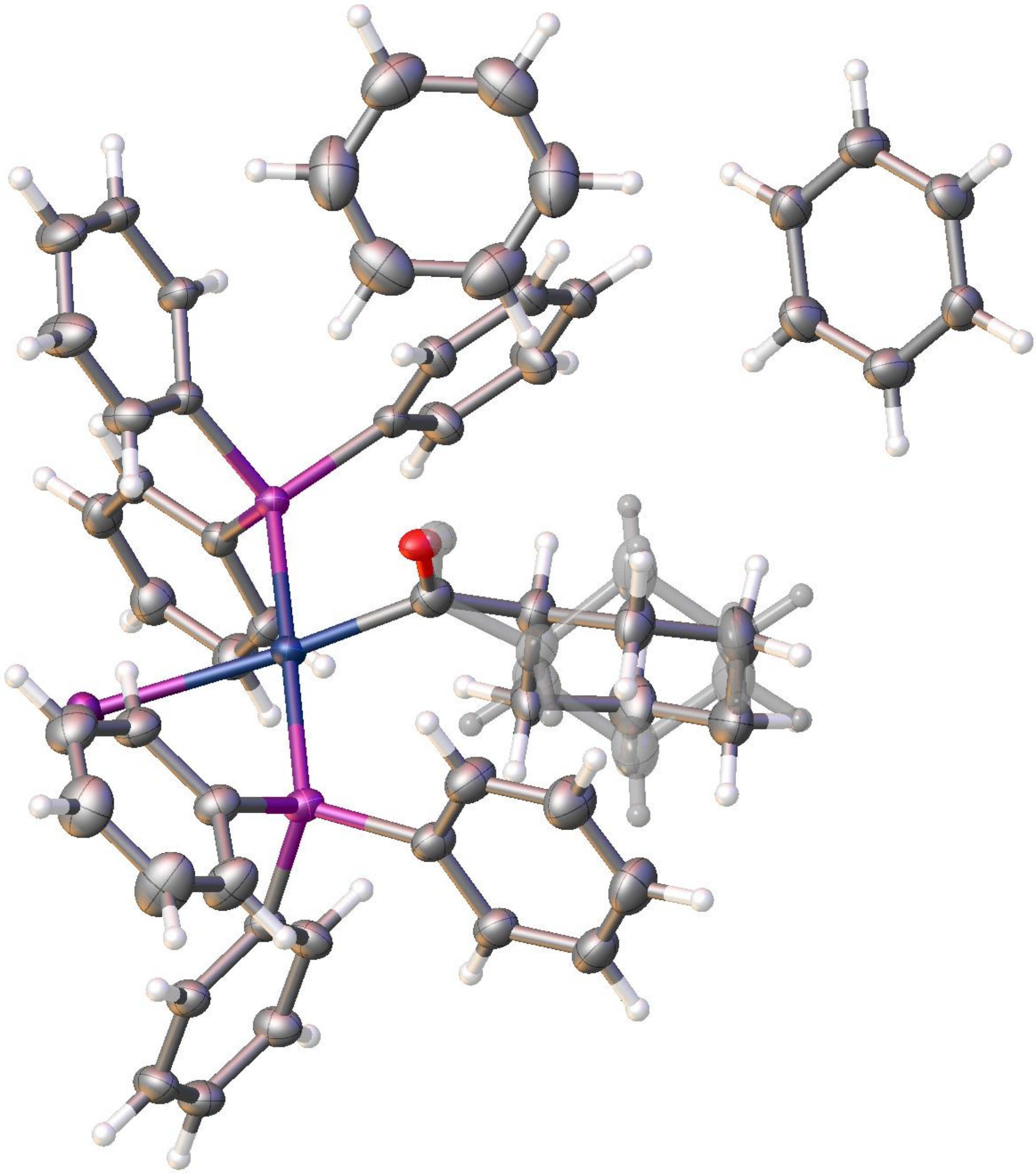

Figure S7: Thermal ellipsoid drawing of compound **trans-31** drawn at 50% probability level. This crystal structure was determined using Mo Kα radiation. The minor part of disorder is depicted as a semi-transparent layer over the major part of disorder. The ratio between major and minor part of disordered were refined at 0.541:0.459(10). Benzene molecules are drawn after grown by symmetry operations.

Computational data

Scans

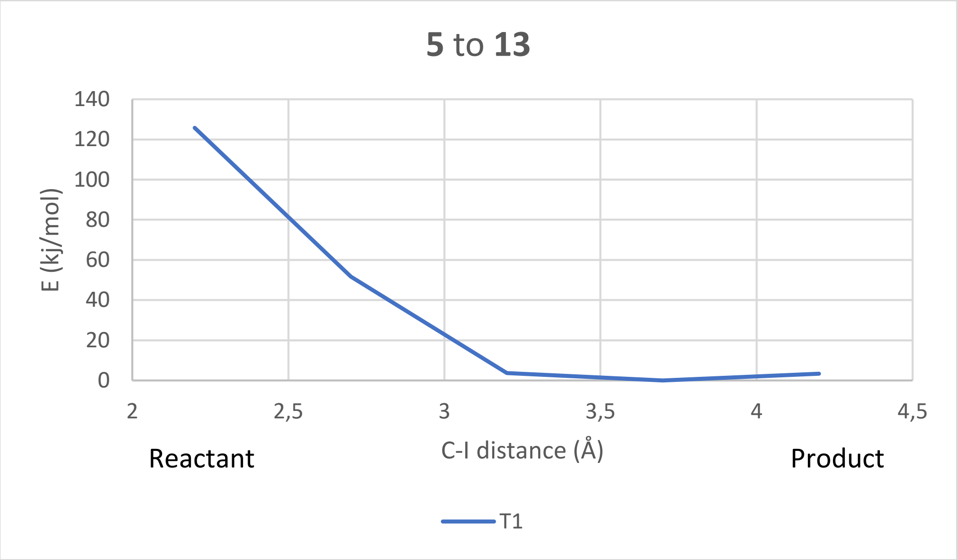

Figure S8: The relaxed surface scan from **5** to **13**

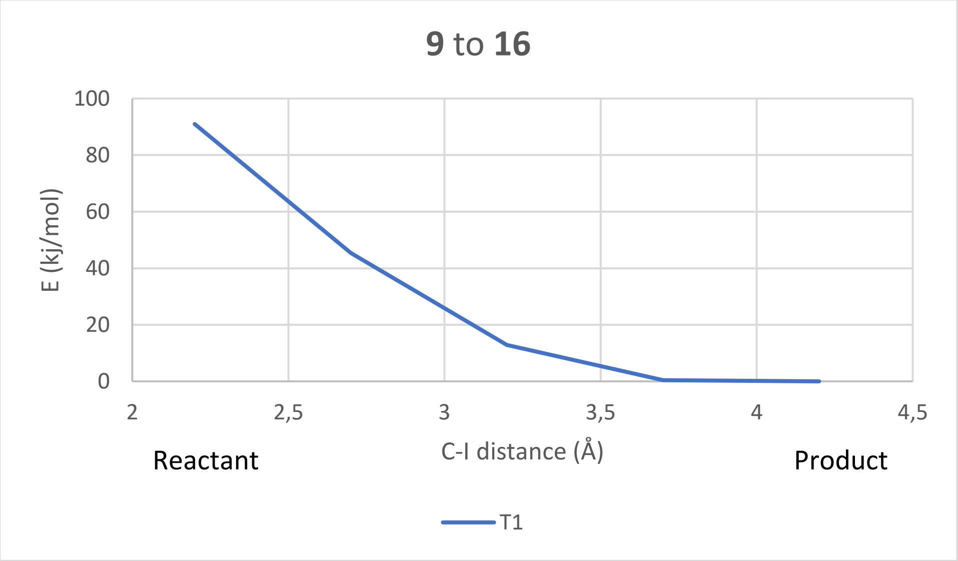

Figure S9: The relaxed surface scan from **9** to **16**

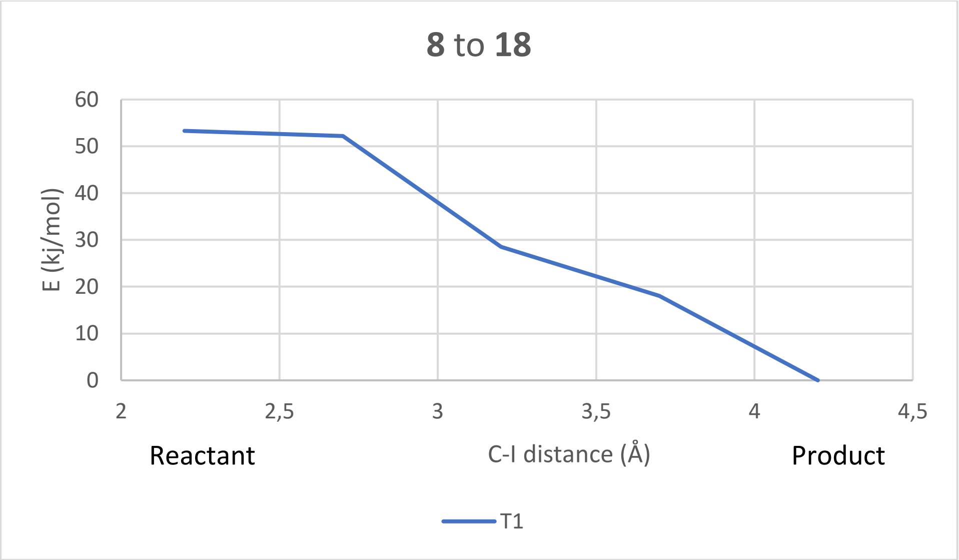

Figure S10: The relaxed surface scan from **8** to **18**

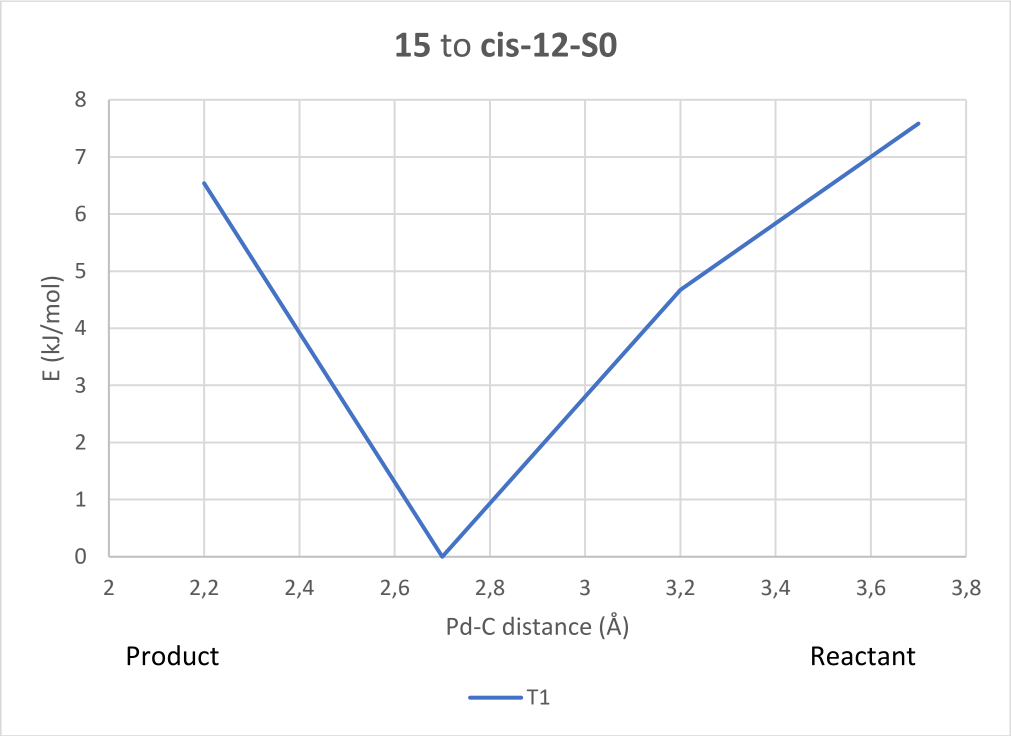

Figure S11: The relaxed surface scan from **15** to **cis-12-S0**

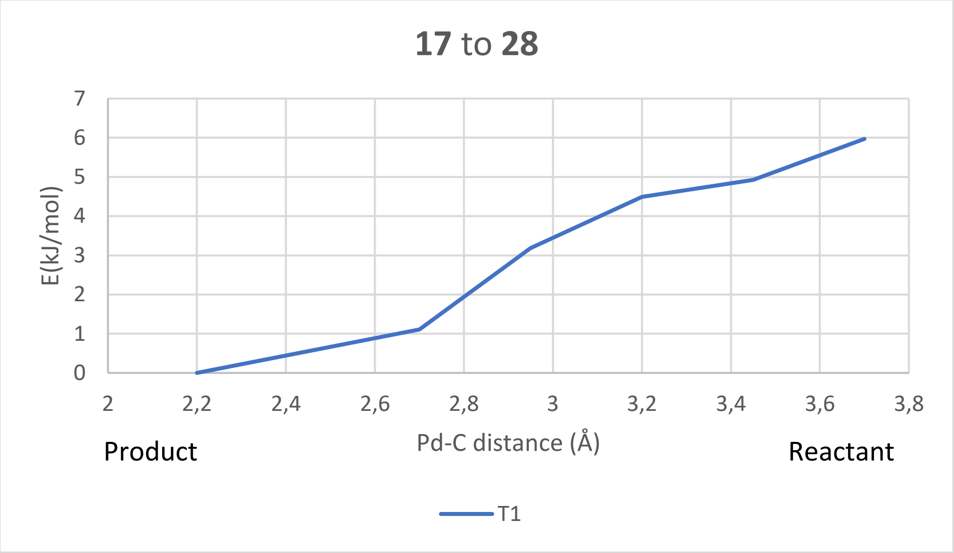

Figure S12: The relaxed surface scan from **17** to **28**

Input files for define and cosmoprep

To ease replication of the calculations presented herein, representative files used to set up TurboMole calculations are provided. The END indicates where each file ends and was not used as input to any program.

Define (def2-SVP)

```
a geom.coord
ired
ff
c 0

*
b all def2-SVP
*
eht
y
y
0
y


dft
func pbe0
grid m4
on
*
dsp
bj
abc
```

*
scf
iter
100

rex

on
hssint
*
*
*
*
*
*
rijk
on
jkbas
b all def2-SVP
*
m 1500
*
mp2
memory 1500
*
*
END

Define (def2-TZVPP)

a coord
syndi
ired
*
b all def2-TZVPP
*
eht
y
y
0
y

dft
func pbe0
grid m4
on

```
*
dsp
bj
abc
*
scf
iter
100

rijk
on
jkbas
b all def2-TZVPP
*
m 1500
*
mp2
memory 1500
*
*
END

Cosmoprep
7.43 #dielectric constant THF
1.4073 #refractive index THF









r all b
*

n

END
```

Energies of geometries

For the below reported compounds, the data reported was calculated thuslly:
E = PBE0-D3(BJ)-abc/def2-TZVPP/DCOSMO-RS(THF)
OC = outlying charge correction from DCOSMO-RS, PBE0-D3(BJ)-abc/def2-TZVPP/DCOSMO-RS(THF)
dU_{298} = PBE0-D3BJ-abc/def2-SVP/COSMO(THF)

dS_{298} = PBE0-D3BJ-abc/def2-SVP/COSMO(THF)

Iodocyclohexane **1**
E (E_h) = -532.8269236
OC (E_h) = -0.000185249
dU_{298} (kJ*mol^{-1}) = 421.42
dS_{298} (kJ*mol^{-1}*K^{-1}) = 0.36561

18

C	1.06180	0.09340	-1.01110
C	-0.40380	0.16600	-1.42390
C	-1.28260	0.61150	-0.26070
C	-1.09430	-0.28790	0.96280
C	0.37360	-0.32800	1.34590
C	1.25830	-0.80760	0.21000
H	1.42950	1.10830	-0.77660
H	1.68450	-0.27790	-1.84040
H	-0.73490	-0.82960	-1.77090
H	-0.52610	0.85030	-2.27860
H	-2.34530	0.61250	-0.55060
H	-1.03370	1.65260	0.01190
H	-1.70690	0.07100	1.80360
H	-1.43940	-1.30970	0.72690
I	0.66040	-1.59060	3.11420
H	0.98980	-1.84640	-0.05020
H	2.31520	-0.81720	0.51650
H	0.70740	0.65740	1.70800

Morpholine **2**
E (E_h) = -287.5979389
OC (E_h) = -0.000134924
dU_{298} (kJ*mol^{-1}) = 354.56
dS_{298} (kJ*mol^{-1}*K^{-1}) = 0.30786

15

C	0.9565438	0.2542586	-1.0908424
O	-0.4041258	0.2918511	-1.4587581
C	-1.2147512	0.7343696	-0.3932653
C	-1.0492587	-0.1522928	0.8269532
N	0.3504496	-0.1697623	1.2077773
C	1.1749618	-0.6439990	0.1124568
H	1.3120019	1.2766962	-0.8498251
H	1.5185308	-0.1131772	-1.9620853
H	-2.2552576	0.7213558	-0.7497432
H	-0.9494127	1.7767012	-0.1232115
H	-1.6586800	0.2448558	1.6542578
H	-1.4399176	-1.1641012	0.5778038
H	0.9353124	-1.6892054	-0.1850497

H	2.2372991	-0.6163088	0.4026872
H	0.4863043	-0.7512416	2.0308444

Cyclohexyl(morpholino)methanone **3**
E (E_h) = -635.378173
OC (E_h) = 0.00018058
dU_{298} (kJ*mol^{-1}) = 779.95
dS_{298} (kJ*mol^{-1}*K^{-1}) = 0.48063

33

C	1.8801929	2.5799238	-2.4257017
O	0.6125800	2.9773271	-2.8963439
C	-0.3937605	2.6835393	-1.9599663
C	-0.4495324	1.1966383	-1.6462037
N	0.8565077	0.7343866	-1.2199763
C	1.9208321	1.0906444	-2.1408161
H	2.1347881	3.1388224	-1.5029275
H	2.6136227	2.8410783	-3.2019902
H	-1.3502939	3.0139517	-2.3900088
H	-0.2192156	3.2466232	-1.0208468
H	-1.2031987	1.0160805	-0.8720514
H	-0.7584057	0.6444344	-2.5530748
H	1.7933431	0.5306387	-3.0854827
H	2.8765353	0.7977497	-1.6895705
C	1.1394418	-0.0256065	-0.1245855
O	2.2856929	-0.3889854	0.1170298
C	-0.6557072	-1.2983188	3.0561662
C	0.4848172	-0.8248580	2.1627834
C	-0.0153288	-0.4447970	0.7709776
C	-0.7789732	-1.6169806	0.1345147
C	-1.9187877	-2.0905731	1.0289412
C	-1.4217349	-2.4549783	2.4232389
H	-1.3513885	-0.4587419	3.2381272
H	-0.2615555	-1.5932114	4.0421972
H	1.2389600	-1.6215133	2.0547210
H	1.0063528	0.0319091	2.6182983
H	-0.7155691	0.4006803	0.8843704
H	-1.1636749	-1.3386717	-0.8594841
H	-0.0650011	-2.4438927	-0.0286040
H	-2.6718341	-1.2853650	1.1088756
H	-2.4288448	-2.9490687	0.5625892
H	-0.7548850	-3.3332064	2.3498744
H	-2.2659750	-2.7556590	3.0649295

$Pd(PPh_3)_4$ **4-S0**
E (E_h) = -4270.716938
OC (E_h) = 8.21077E-05
dU_{298} (kJ*mol^{-1}) = 2969.35
dS_{298} (kJ*mol^{-1}*K^{-1}) = 1.63499

137			
Pd	0.0000000	0.0000000	0.0000000
P	-1.3893223	1.3893223	1.3893223
P	1.3893223	-1.3893223	1.3893223
P	-1.3893223	-1.3893223	-1.3893223
P	1.3893223	1.3893223	-1.3893223
C	4.0405126	-4.5269526	-0.7995624
C	2.7041640	-4.3335055	-1.1499330
C	1.9405891	-3.3838813	-0.4779222
C	2.4880369	-2.6230568	0.5616425
C	3.8288091	-2.8271378	0.9110611
C	4.5994592	-3.7701852	0.2295517
H	4.6478530	-5.2631761	-1.3319467
H	2.2509771	-4.9058439	-1.9623861
H	0.9073423	-3.2170654	-0.7809282
H	4.2773351	-2.2446691	1.7183313
H	5.6457180	-3.9151261	0.5107903
C	-0.7995624	-4.0405126	4.5269526
C	0.2295517	-4.5994592	3.7701852
C	0.9110611	-3.8288091	2.8271378
C	0.5616425	-2.4880369	2.6230568
C	-0.4779222	-1.9405891	3.3838813
C	-1.1499330	-2.7041640	4.3335055
H	-1.3319467	-4.6478530	5.2631761
H	0.5107903	-5.6457180	3.9151261
H	1.7183313	-4.2773351	2.2446691
H	-0.7809282	-0.9073423	3.2170654
H	-1.9623861	-2.2509771	4.9058439
C	4.5269526	0.7995624	4.0405126
C	3.7701852	-0.2295517	4.5994592
C	2.8271378	-0.9110611	3.8288091
C	2.6230568	-0.5616425	2.4880369
C	3.3838813	0.4779222	1.9405891
C	4.3335055	1.1499330	2.7041640
H	5.2631761	1.3319467	4.6478530
H	3.9151261	-0.5107903	5.6457180
H	2.2446691	-1.7183313	4.2773351
H	3.2170654	0.7809282	0.9073423
H	4.9058439	1.9623861	2.2509771
C	-4.5269526	-0.7995624	4.0405126
C	-3.7701852	0.2295517	4.5994592
C	-2.8271378	0.9110611	3.8288091
C	-2.6230568	0.5616425	2.4880369
C	-3.3838813	-0.4779222	1.9405891
C	-4.3335055	-1.1499330	2.7041640
H	-5.2631761	-1.3319467	4.6478530
H	-3.9151261	0.5107903	5.6457180
H	-2.2446691	1.7183313	4.2773351

H	-3.2170654	-0.7809282	0.9073423
H	-4.9058439	-1.9623861	2.2509771
C	0.7995624	4.0405126	4.5269526
C	1.1499330	2.7041640	4.3335055
C	0.4779222	1.9405891	3.3838813
C	-0.5616425	2.4880369	2.6230568
C	-0.9110611	3.8288091	2.8271378
C	-0.2295517	4.5994592	3.7701852
H	1.3319467	4.6478530	5.2631761
H	1.9623861	2.2509771	4.9058439
H	0.7809282	0.9073423	3.2170654
H	-1.7183313	4.2773351	2.2446691
H	-0.5107903	5.6457180	3.9151261
C	-4.0405126	4.5269526	-0.7995624
C	-2.7041640	4.3335055	-1.1499330
C	-1.9405891	3.3838813	-0.4779222
C	-2.4880369	2.6230568	0.5616425
C	-3.8288091	2.8271378	0.9110611
C	-4.5994592	3.7701852	0.2295517
H	-4.6478530	5.2631761	-1.3319467
H	-2.2509771	4.9058439	-1.9623861
H	-0.9073423	3.2170654	-0.7809282
H	-4.2773351	2.2446691	1.7183313
H	-5.6457180	3.9151261	0.5107903
C	0.7995624	-4.0405126	-4.5269526
C	1.1499330	-2.7041640	-4.3335055
C	0.4779222	-1.9405891	-3.3838813
C	-0.5616425	-2.4880369	-2.6230568
C	-0.9110611	-3.8288091	-2.8271378
C	-0.2295517	-4.5994592	-3.7701852
H	1.3319467	-4.6478530	-5.2631761
H	1.9623861	-2.2509771	-4.9058439
H	0.7809282	-0.9073423	-3.2170654
H	-1.7183313	-4.2773351	-2.2446691
H	-0.5107903	-5.6457180	-3.9151261
C	-4.0405126	-4.5269526	0.7995624
C	-2.7041640	-4.3335055	1.1499330
C	-1.9405891	-3.3838813	0.4779222
C	-2.4880369	-2.6230568	-0.5616425
C	-3.8288091	-2.8271378	-0.9110611
C	-4.5994592	-3.7701852	-0.2295517
H	-4.6478530	-5.2631761	1.3319467
H	-2.2509771	-4.9058439	1.9623861
H	-0.9073423	-3.2170654	0.7809282
H	-4.2773351	-2.2446691	-1.7183313
H	-5.6457180	-3.9151261	-0.5107903
C	-4.5269526	0.7995624	-4.0405126
C	-4.3335055	1.1499330	-2.7041640
C	-3.3838813	0.4779222	-1.9405891

C	-2.6230568	-0.5616425	-2.4880369
C	-2.8271378	-0.9110611	-3.8288091
C	-3.7701852	-0.2295517	-4.5994592
H	-5.2631761	1.3319467	-4.6478530
H	-4.9058439	1.9623861	-2.2509771
H	-3.2170654	0.7809282	-0.9073423
H	-2.2446691	-1.7183313	-4.2773351
H	-3.9151261	-0.5107903	-5.6457180
C	4.0405126	4.5269526	0.7995624
C	2.7041640	4.3335055	1.1499330
C	1.9405891	3.3838813	0.4779222
C	2.4880369	2.6230568	-0.5616425
C	3.8288091	2.8271378	-0.9110611
C	4.5994592	3.7701852	-0.2295517
H	4.6478530	5.2631761	1.3319467
H	2.2509771	4.9058439	1.9623861
H	0.9073423	3.2170654	0.7809282
H	4.2773351	2.2446691	-1.7183313
H	5.6457180	3.9151261	-0.5107903
C	4.5269526	-0.7995624	-4.0405126
C	4.3335055	-1.1499330	-2.7041640
C	3.3838813	-0.4779222	-1.9405891
C	2.6230568	0.5616425	-2.4880369
C	2.8271378	0.9110611	-3.8288091
C	3.7701852	0.2295517	-4.5994592
H	5.2631761	-1.3319467	-4.6478530
H	4.9058439	-1.9623861	-2.2509771
H	3.2170654	-0.7809282	-0.9073423
H	2.2446691	1.7183313	-4.2773351
H	3.9151261	0.5107903	-5.6457180
C	-0.7995624	4.0405126	-4.5269526
C	0.2295517	4.5994592	-3.7701852
C	0.9110611	3.8288091	-2.8271378
C	0.5616425	2.4880369	-2.6230568
C	-0.4779222	1.9405891	-3.3838813
C	-1.1499330	2.7041640	-4.3335055
H	-1.3319467	4.6478530	-5.2631761
H	0.5107903	5.6457180	-3.9151261
H	1.7183313	4.2773351	-2.2446691
H	-0.7809282	0.9073423	-3.2170654
H	-1.9623861	2.2509771	-4.9058439

$Pd(PPh_3)_3$ -S0 **5-S0**
E (E_h) = -3235.028563
OC (E_h) = 4.70252E-05
dU_{298} ($kJ*mol^{-1}$) = 2222.87
dS_{298} ($kJ*mol^{-1}*K^{-1}$) = 1.36949

103

C	-0.0938464	2.3643798	5.5153238
C	1.2380871	2.5399322	5.1390210
C	1.6185382	2.3788297	3.8061892
C	0.6691969	2.0288205	2.8397080
C	-0.6632595	1.8385277	3.2303191
C	-1.0463731	2.0168100	4.5568031
H	-0.3875244	2.4855072	6.5611092
H	1.9887401	2.8034781	5.8888345
H	2.6642620	2.5179328	3.5209361
H	-1.3934372	1.5157497	2.4811871
H	-2.0867703	1.8560564	4.8491224
C	0.8985134	5.9384485	-0.8989566
C	0.5347522	5.8077203	0.4396346
C	0.6101549	4.5640249	1.0722280
C	1.0388333	3.4368647	0.3644151
C	1.3878129	3.5758839	-0.9878201
C	1.3316612	4.8168682	-1.6109266
H	0.8370068	6.9116759	-1.3924494
H	0.1927641	6.6803257	1.0026221
H	0.3313713	4.4724275	2.1249252
H	1.6895457	2.6943942	-1.5604674
H	1.6016730	4.9060704	-2.6658153
C	5.5624177	0.6169658	1.0090730
C	5.2011250	1.9638436	1.0726408
C	3.8553149	2.3294796	1.0973637
C	2.8548055	1.3494363	1.0631235
C	3.2273985	-0.0005359	0.9913239
C	4.5723556	-0.3656804	0.9677856
H	6.6178392	0.3339939	0.9839715
H	5.9734988	2.7368790	1.0994461
H	3.5799708	3.3867276	1.1333205
H	2.4461572	-0.7647775	0.9308582
H	4.8461668	-1.4220444	0.9055220
P	1.0587869	1.7398446	1.0685334
C	2.9540590	-1.0598836	-4.7437824
C	1.6710296	-1.1747286	-5.2816446
C	0.5592994	-0.7841013	-4.5353693
C	0.7199433	-0.2695202	-3.2422229
C	2.0120383	-0.1654713	-2.7074191
C	3.1229322	-0.5547477	-3.4538664
H	3.8228424	-1.3720597	-5.3289153
H	1.5339811	-1.5752364	-6.2894305
H	-0.4432918	-0.8897294	-4.9579671
H	2.1354708	0.2051843	-1.6848801
H	4.1230291	-0.4728686	-3.0204483
C	-1.7303156	4.5762269	-3.4763271
C	-1.0702614	3.7472194	-4.3838502
C	-0.7574040	2.4328061	-4.0352758
C	-1.0928285	1.9405073	-2.7691769

C	-1.7411582	2.7858929	-1.8586381
C	-2.0695765	4.0916590	-2.2125978
H	-1.9687313	5.6071377	-3.7501510
H	-0.7949031	4.1261362	-5.3716290
H	-0.2398966	1.7908531	-4.7524135
H	-1.9569366	2.4135057	-0.8520285
H	-2.5664638	4.7429242	-1.4896356
C	-4.1716909	-2.4173764	-3.6882604
C	-4.2496024	-1.0386728	-3.8733024
C	-3.1986088	-0.2124790	-3.4665009
C	-2.0649401	-0.7600790	-2.8581038
C	-2.0031844	-2.1483576	-2.6613599
C	-3.0399364	-2.9714859	-3.0845227
H	-4.9954395	-3.0617709	-4.0055556
H	-5.1329192	-0.5975095	-4.3428454
H	-3.2642399	0.8669590	-3.6239511
H	-1.1376531	-2.5837135	-2.1541168
H	-2.9777247	-4.0496286	-2.9189595
P	-0.6874293	0.2491335	-2.1799384
Pd	-0.1642171	0.0844836	0.0499857
C	-4.7882495	-3.6138640	-0.0209904
C	-3.7593230	-4.4850470	0.3379033
C	-2.5187851	-3.9822705	0.7321016
C	-2.2943436	-2.6014357	0.7591365
C	-3.3289321	-1.7348600	0.3812153
C	-4.5722685	-2.2356150	0.0051554
H	-5.7568736	-4.0109210	-0.3351440
H	-3.9221024	-5.5657148	0.3100027
H	-1.7185192	-4.6722356	1.0110492
H	-3.1343316	-0.6578357	0.3554033
H	-5.3670370	-1.5491416	-0.2962276
C	-0.7823583	-1.4773656	5.8349493
C	-1.9209131	-1.9293739	5.1711824
C	-1.9124988	-2.0835179	3.7823867
C	-0.7659653	-1.7731093	3.0446227
C	0.3704914	-1.3030846	3.7208234
C	0.3680033	-1.1692128	5.1041182
H	-0.7915051	-1.3558049	6.9210625
H	-2.8255325	-2.1702128	5.7360019
H	-2.8071760	-2.4472049	3.2709242
H	1.2603948	-1.0228700	3.1502532
H	1.2596743	-0.7968205	5.6139010
C	2.4894316	-5.0988132	0.3650469
C	1.7759080	-5.1415217	1.5640509
C	0.8103726	-4.1739832	1.8414611
C	0.5449890	-3.1539354	0.9186134
C	1.2724375	-3.1146220	-0.2795226
C	2.2363186	-4.0823818	-0.5570281
H	3.2494108	-5.8552170	0.1531083

H	1.9751097	-5.9327750	2.2914345
H	0.2651381	-4.2060916	2.7883578
H	1.0891271	-2.2988091	-0.9858803
H	2.7990210	-4.0343173	-1.4928866
P	-0.6919245	-1.8260839	1.2096093

Pd(PPh$_3$)$_3$ **5-T1/S1**
E-T1 (E_h) = -3234.945253
E-S1 (E_h) = -3234.936153
OC (E_h) = -1.80298E-05
dU_{298} (kJ*mol^{-1}) = 2216.99
dS_{298} (kJ*mol^{-1}*K^{-1}) = 1.3465

103

P	1.1505412	2.0671057	-0.3156806
C	1.0513960	4.5416013	-4.2207591
C	1.0076676	3.1408911	-4.2607264
C	1.0374027	2.4103871	-3.0777343
C	1.1502076	3.0618187	-1.8393727
C	1.1966945	4.4665409	-1.8046262
C	1.1403937	5.1969933	-2.9930548
H	1.0063612	5.1182294	-5.1479990
H	0.9319798	2.6201023	-5.2187216
H	0.9431146	1.3209801	-3.1053073
H	1.2714052	4.9857374	-0.8454396
H	1.1671056	6.2894368	-2.9577658
C	-1.1961672	4.2932247	2.9751224
C	-1.7519859	4.0743831	1.7147654
C	-0.9940457	3.4877395	0.7027086
C	0.3312437	3.0702406	0.9598918
C	0.8927475	3.3214306	2.2218350
C	0.1367749	3.9312652	3.2190619
H	-1.7937053	4.7520802	3.7666215
H	-2.7896867	4.3528318	1.5149117
H	-1.4328576	3.3200580	-0.2839936
H	1.9211406	3.0175564	2.4289558
H	0.5830185	4.1122271	4.2003029
C	5.5431879	1.7761197	1.1047670
C	4.5586506	0.9343689	1.6246094
C	3.2456882	1.0390687	1.1752612
C	2.9001259	2.0013769	0.2166961
C	3.8917077	2.8359238	-0.3092118
C	5.2085270	2.7228378	0.1358482
H	6.5775047	1.6845635	1.4459099
H	4.8199473	0.1771857	2.3676200
H	2.4727819	0.3595814	1.5500705
H	3.6363170	3.5737651	-1.0732504
H	5.9786379	3.3786599	-0.2784644
P	0.8657494	-1.4721421	-1.1273109

C	-0.3853370	-5.6114473	0.5253499
C	-0.1426053	-4.5375200	1.3819410
C	0.2434950	-3.3037728	0.8637314
C	0.4087136	-3.1361622	-0.5181912
C	0.1782383	-4.2214109	-1.3717948
C	-0.2232862	-5.4505031	-0.8507154
H	-0.7047982	-6.5746017	0.9308135
H	-0.2670115	-4.6573219	2.4609187
H	0.4017655	-2.4527140	1.5327245
H	0.2919642	-4.1036546	-2.4514237
H	-0.4158207	-6.2872638	-1.5268559
C	0.0253266	-1.5949031	-5.6895755
C	-0.8778791	-0.9930919	-4.8124803
C	-0.6064856	-0.9657352	-3.4454354
C	0.5597505	-1.5519907	-2.9413219
C	1.4605304	-2.1600341	-3.8264751
C	1.1936257	-2.1775904	-5.1948705
H	-0.1778393	-1.6053266	-6.7635045
H	-1.7920571	-0.5318842	-5.1947929
H	-1.2853653	-0.4594291	-2.7480938
H	2.3782818	-2.6149971	-3.4452524
H	1.9042755	-2.6468817	-5.8800012
C	5.4871329	-1.3020170	-0.8464009
C	4.7867649	-0.4085268	-1.6579827
C	3.4013549	-0.4853794	-1.7495648
C	2.6908110	-1.4674163	-1.0398127
C	3.4011409	-2.3613868	-0.2274918
C	4.7890612	-2.2766159	-0.1319754
H	6.5748123	-1.2353241	-0.7674995
H	5.3218321	0.3649583	-2.2134539
H	2.8665314	0.2265802	-2.3829205
H	2.8683066	-3.1344048	0.3305061
H	5.3291384	-2.9827012	0.5040568
Pd	-0.2653942	0.2683352	-0.1224317
P	-2.0986804	-0.4977300	1.1073799
C	-4.5543146	-4.2677456	0.0289637
C	-3.5662676	-3.7946649	-0.8352856
C	-2.8295934	-2.6614467	-0.4978734
C	-3.0579671	-2.0005361	0.7129630
C	-4.0667523	-2.4684036	1.5691077
C	-4.8066687	-3.5981918	1.2286539
H	-5.1353166	-5.1550805	-0.2348541
H	-3.3648370	-4.3104970	-1.7770861
H	-2.0647029	-2.2801104	-1.1763361
H	-4.2744563	-1.9467883	2.5071060
H	-5.5884255	-3.9575549	1.9027995
C	-0.4977369	-0.8013375	5.4425394
C	-0.5921707	0.4291440	4.7810580
C	-1.1322587	0.5001740	3.5036369

C	-1.6123722	-0.6589369	2.8616038
C	-1.5217660	-1.8883892	3.5342063
C	-0.9657913	-1.9556687	4.8109076
H	-0.0641236	-0.8579115	6.4439655
H	-0.2322939	1.3424655	5.2621427
H	-1.1730196	1.4647953	2.9904172
H	-1.8850195	-2.7995783	3.0547543
H	-0.9035490	-2.9214559	5.3199077
C	-5.3841741	2.7867169	1.0839114
C	-4.7080170	2.4325285	-0.0843863
C	-3.7177929	1.4522761	-0.0463278
C	-3.3999630	0.8093205	1.1562988
C	-4.0906406	1.1591082	2.3218374
C	-5.0736262	2.1477893	2.2857991
H	-6.1548882	3.5614180	1.0585523
H	-4.9472712	2.9275471	-1.0292230
H	-3.1641300	1.1869725	-0.9537617
H	-3.8516811	0.6663064	3.2671146
H	-5.5982789	2.4234301	3.2042427

$Pd(PPh_3)_2$ -S0 **6-S0**
E (E_h) = -2199.325232
OC (E_h) = -0.000195212
dU_{298} ($kJ*mol^{-1}$) = 1477.8
dS_{298} ($kJ*mol^{-1}*K^{-1}$) = 0.99012

69

P	0.7691523	2.1158856	0.3423087
C	-1.2268794	5.3228606	-2.3412251
C	-0.9523678	4.0424726	-2.8263638
C	-0.3669257	3.0924264	-1.9926593
C	-0.0341799	3.4176113	-0.6700916
C	-0.3144806	4.7015993	-0.1887464
C	-0.9111123	5.6485541	-1.0222780
H	-1.6948296	6.0660929	-2.9916230
H	-1.2056911	3.7799016	-3.8565241
H	-0.1704163	2.0800544	-2.3600078
H	-0.0688720	4.9646759	0.8430085
H	-1.1299760	6.6475506	-0.6368802
C	0.1547587	3.6887288	4.6572372
C	-0.7974027	2.8732682	4.0420883
C	-0.5794923	2.4006881	2.7499639
C	0.5850072	2.7521902	2.0528434
C	1.5369613	3.5674232	2.6758129
C	1.3218102	4.0308987	3.9743729
H	-0.0108768	4.0522814	5.6744805
H	-1.7095414	2.5956270	4.5762018
H	-1.3113428	1.7424272	2.2708558
H	2.4531657	3.8407914	2.1467713

H	2.0723279	4.6638484	4.4544888
C	5.3072513	2.6396560	-0.4329231
C	4.8005642	1.4675102	0.1321260
C	3.4298515	1.3298970	0.3388892
C	2.5529490	2.3693668	-0.0018147
C	3.0665051	3.5405322	-0.5706837
C	4.4388376	3.6721915	-0.7868193
H	6.3814726	2.7449361	-0.6042675
H	5.4760930	0.6523183	0.4031697
H	3.0238883	0.4046402	0.7602265
H	2.3926287	4.3541984	-0.8498271
H	4.8307547	4.5886591	-1.2350476
Pd	0.0002989	-0.0002632	0.0001441
P	-0.7693297	-2.1161422	-0.3420699
C	-4.4815859	-3.2259482	2.1991914
C	-3.4684400	-2.4147366	2.7144815
C	-2.3714250	-2.0827482	1.9227321
C	-2.2675066	-2.5721307	0.6130371
C	-3.2876654	-3.3822599	0.1011878
C	-4.3911162	-3.7047762	0.8922691
H	-5.3472387	-3.4792650	2.8163201
H	-3.5389325	-2.0302187	3.7350342
H	-1.5851928	-1.4285101	2.3128690
H	-3.2233166	-3.7622173	-0.9213497
H	-5.1848543	-4.3348167	0.4830675
C	-2.0658391	-2.9860484	-4.7045663
C	-2.2865120	-1.7373934	-4.1196731
C	-1.8713571	-1.4977285	-2.8117642
C	-1.2461516	-2.5092171	-2.0691256
C	-1.0249263	-3.7576509	-2.6618826
C	-1.4319474	-3.9923540	-3.9758803
H	-2.3827271	-3.1722487	-5.7337925
H	-2.7751788	-0.9431016	-4.6894509
H	-2.0206994	-0.5140411	-2.3552028
H	-0.5296518	-4.5514642	-2.0972804
H	-1.2517950	-4.9690013	-4.4321104
C	2.3126201	-5.4390093	0.6222922
C	2.7086457	-4.2300195	0.0464764
C	1.7632670	-3.2416611	-0.2177366
C	0.4094277	-3.4581042	0.0753457
C	0.0190950	-4.6707298	0.6550404
C	0.9692981	-5.6553299	0.9290752
H	3.0548439	-6.2114100	0.8388373
H	3.7612095	-4.0525067	-0.1879105
H	2.0705349	-2.2828687	-0.6475471
H	-1.0315692	-4.8481732	0.8973439
H	0.6561248	-6.5976689	1.3855345

$Pd(PPh_3)_2$ **6-T1/S1**

E-T1 (E_h) = -2199.249000
E-S1 (E_h) = -2199.226869
OC (E_h) = -0.002913329
dU_{298} (kJ*mol^{-1}) = 1482.73
dS_{298} (kJ*mol^{-1}*K^{-1}) = 0.99084

69

P	-0.9274672	1.1133751	1.2849263
C	-1.4372329	4.4605487	-1.8690347
C	-1.2814045	3.1311149	-2.2615271
C	-1.1471639	2.1316675	-1.2989264
C	-1.1565369	2.4546004	0.0638748
C	-1.3297386	3.7901228	0.4525534
C	-1.4653205	4.7874852	-0.5119860
H	-1.5473169	5.2446528	-2.6222203
H	-1.2746284	2.8649709	-3.3213232
H	-1.0504878	1.0894283	-1.6095520
H	-1.3641633	4.0515467	1.5132010
H	-1.5969032	5.8267968	-0.2011794
C	-3.3340171	2.6145092	4.9409949
C	-3.9719324	2.3013792	3.7380448
C	-3.2283123	1.8527712	2.6504034
C	-1.8333956	1.7320480	2.7489130
C	-1.1988499	2.0468510	3.9551007
C	-1.9501941	2.4833640	5.0478023
H	-3.9193122	2.9591269	5.7970887
H	-5.0566539	2.4000444	3.6498013
H	-3.7324377	1.5928501	1.7144120
H	-0.1140642	1.9544295	4.0432527
H	-1.4468258	2.7255586	5.9870667
C	3.4878033	1.2180903	2.6321945
C	2.7168632	0.0639418	2.7772670
C	1.3909585	0.0555443	2.3546889
C	0.8206993	1.2057843	1.7922113
C	1.5995512	2.3593389	1.6435805
C	2.9285380	2.3624538	2.0636284
H	4.5321556	1.2217615	2.9537437
H	3.1553720	-0.8397909	3.2062609
H	0.7904251	-0.8547687	2.4437974
H	1.1711232	3.2579558	1.1942096
H	3.5321433	3.2653143	1.9417680
Pd	-1.7728203	-0.9540335	0.6619312
P	-0.1645864	-1.7277608	-0.8406957
C	-0.1862123	-6.3515309	-0.7936456
C	-0.4304866	-5.6276268	0.3745163
C	-0.4461760	-4.2342403	0.3390856
C	-0.1975370	-3.5569190	-0.8610735
C	0.0502553	-4.2864054	-2.0295719
C	0.0506778	-5.6804845	-1.9943153

H	-0.1852630	-7.4441412	-0.7693201
H	-0.6198990	-6.1496195	1.3156091
H	-0.6629912	-3.6613074	1.2470334
H	0.2417919	-3.7646924	-2.9705989
H	0.2378514	-6.2458159	-2.9106156
C	-1.4048431	-0.4600486	-5.1158163
C	-2.3603173	-0.7819352	-4.1503235
C	-1.9603051	-1.1717046	-2.8747789
C	-0.5964745	-1.2427151	-2.5487942
C	0.3576083	-0.9303499	-3.5261075
C	-0.0476560	-0.5382963	-4.8014448
H	-1.7186052	-0.1501081	-6.1156915
H	-3.4249404	-0.7247190	-4.3897294
H	-2.7088368	-1.4105248	-2.1118037
H	1.4223091	-0.9884634	-3.2913429
H	0.7049762	-0.2941728	-5.5552224
C	4.3153032	-0.6803385	-0.3248759
C	3.3995031	0.2913075	-0.7343715
C	2.0597207	-0.0381126	-0.9023159
C	1.6169070	-1.3541493	-0.6923770
C	2.5402525	-2.3224607	-0.2776899
C	3.8805482	-1.9835066	-0.0920518
H	5.3658542	-0.4171757	-0.1802409
H	3.7280383	1.3184576	-0.9070292
H	1.3503869	0.7384581	-1.1992876
H	2.2149142	-3.3502803	-0.1037766
H	4.5897786	-2.7494515	0.2316946

$Pd(PPh_3)_1$ **7-S0**
E (E_h) = -1163.602457
OC (E_h) = -7.03534E-05
dU_{298} (kJ*mol^{-1}) = 741.36
dS_{298} (kJ*mol^{-1}*K^{-1}) = 0.62586

35

C	1.1241607	-0.5677824	4.0551123
C	0.8039108	0.5913676	3.3491478
C	0.1755755	0.5061160	2.1057779
C	-0.1446368	-0.7435247	1.5623020
C	0.1691122	-1.9040528	2.2833719
C	0.8075068	-1.8175579	3.5181310
H	1.6158774	-0.4983471	5.0286553
H	1.0453691	1.5720639	3.7668359
H	-0.0660253	1.4196026	1.5574046
H	-0.1000318	-2.8805309	1.8686555
H	1.0500328	-2.7293692	4.0696520
C	-2.4043742	3.3733893	-0.9326919
C	-1.1695987	2.9649930	-1.4356851
C	-0.7092216	1.6684180	-1.2012189

C	-1.4873878	0.7661894	-0.4663160
C	-2.7334196	1.1798109	0.0252667
C	-3.1854707	2.4780083	-0.1986182
H	-2.7626860	4.3891939	-1.1175228
H	-0.5562668	3.6600763	-2.0146658
H	0.2623518	1.3596140	-1.5938983
H	-3.3520381	0.4688443	0.5818044
H	-4.1569848	2.7903262	0.1925832
C	2.6299040	-1.4466512	-3.0036089
C	2.8780098	-1.0348943	-1.6946628
C	1.8229495	-0.8834314	-0.7935467
C	0.5091144	-1.1517143	-1.1950839
C	0.2686420	-1.5773874	-2.5089777
C	1.3211350	-1.7154545	-3.4105272
H	3.4576972	-1.5647405	-3.7073015
H	3.9006830	-0.8276504	-1.3695094
H	2.0266590	-0.5545360	0.2281608
H	-0.7557856	-1.8077448	-2.8177976
H	1.1210287	-2.0446920	-4.4332485
P	-0.9389023	-0.9454060	-0.0825692
Pd	-2.4668895	-2.4925459	-0.2154107

$Pd(PPh_3)_1$ **7-T1/S1**
E-T1 (E_h) = -1163.540177
E-S1 (E_h) = -1163.514647
OC (E_h) = -0.00271107
dU_{298} ($kJ*mol^{-1}$) = 741.34
dS_{298} ($kJ*mol^{-1}*K^{-1}$) = 0.63853

35

C	1.0880775	-0.6394995	4.1278534
C	0.7042328	0.5503443	3.5080713
C	0.1302858	0.5267783	2.2376303
C	-0.0635620	-0.6946089	1.5813151
C	0.3129992	-1.8903004	2.2124889
C	0.8929040	-1.8598095	3.4783411
H	1.5381497	-0.6165355	5.1233166
H	0.8549043	1.5062402	4.0155292
H	-0.1654903	1.4610528	1.7547309
H	0.1533402	-2.8488117	1.7089199
H	1.1874078	-2.7937482	3.9628077
C	-2.5106840	3.3766370	-0.9563191
C	-1.2972992	3.0015888	-1.5345921
C	-0.7598951	1.7401189	-1.2823456
C	-1.4399339	0.8453153	-0.4472757
C	-2.6655445	1.2215904	0.1234251
C	-3.1938323	2.4858814	-0.1263879
H	-2.9282451	4.3664691	-1.1561380
H	-0.7621283	3.6969535	-2.1857355

H	0.1919182	1.4516916	-1.7346389
H	-3.2069922	0.5218810	0.7676864
H	-4.1467521	2.7743649	0.3235216
C	2.6154074	-1.5352971	-3.0695470
C	2.9282426	-1.1749075	-1.7582112
C	1.9119027	-0.9503902	-0.8305748
C	0.5725546	-1.0880665	-1.2147274
C	0.2619000	-1.4612415	-2.5313938
C	1.2813202	-1.6772868	-3.4555506
H	3.4149429	-1.7100590	-3.7935848
H	3.9719158	-1.0646715	-1.4540143
H	2.1612835	-0.6654976	0.1942628
H	-0.7829937	-1.5812247	-2.8340703
H	1.0330168	-1.9645505	-4.4800051
P	-0.8122539	-0.8175244	-0.0697003
Pd	-2.4710991	-2.4928765	-0.2050877

$Pd(PPh_3)_1(CO)_2$ **8-S0**
E (E_h) = -1390.163148
OC (E_h) = 0.000159291
dU_{298} ($kJ*mol^{-1}$) = 791.68
dS_{298} ($kJ*mol^{-1}*K^{-1}$) = 0.73618

39

C	1.8643047	3.8173487	-0.2904045
Pd	2.4557602	2.0204202	0.1246167
P	0.6249679	0.4985755	0.0874159
C	-3.4706523	2.3955308	1.1074484
C	-2.3096642	2.7897494	1.7749196
C	-1.0850135	2.2162054	1.4398053
C	-1.0129053	1.2319458	0.4452848
C	-2.1800992	0.8402462	-0.2210424
C	-3.4033259	1.4241447	0.1083795
H	-4.4301836	2.8518552	1.3629755
H	-2.3563939	3.5551287	2.5533779
H	-0.1722449	2.5386249	1.9501933
H	-2.1343993	0.0781012	-1.0027700
H	-4.3094270	1.1170665	-0.4197399
C	0.0514941	-1.2797554	-4.1465232
C	0.3547139	0.0714021	-3.9673573
C	0.5350636	0.5809882	-2.6838297
C	0.4004908	-0.2541185	-1.5651501
C	0.0975788	-1.6078691	-1.7503568
C	-0.0738546	-2.1168012	-3.0381153
H	-0.0826517	-1.6814824	-5.1538809
H	0.4593453	0.7305209	-4.8325619
H	0.7840068	1.6374405	-2.5456679
H	-0.0051702	-2.2689212	-0.8866651
H	-0.3085141	-3.1754122	-3.1740683

C 0.8966465 -3.2010371 2.8608379
C 2.0064659 -2.7618620 2.1359363
C 1.9066121 -1.6360983 1.3225778
C 0.6903760 -0.9483847 1.2088886
C -0.4188651 -1.3933418 1.9374500
C -0.3122922 -2.5134083 2.7629170
H 0.9772907 -4.0783610 3.5073586
H 2.9581448 -3.2933335 2.2125658
H 2.7816414 -1.2850633 0.7688536
H -1.3718549 -0.8648888 1.8621539
H -1.1827454 -2.8502413 3.3312902
C 4.1738660 1.2381422 0.5479823
O 5.1661350 0.7289237 0.8007798
O 1.4353529 4.8480196 -0.5358757

$Pd(PPh_3)_1(CO)_2$ **8-T1/S1**
E-T1 (E_h) = -1390.095688
E-S1 (E_h) = -1390.083344
OC (E_h) = -4.64026E-05
dU_{298} ($kJ*mol^{-1}$) = 793.59
dS_{298} ($kJ*mol^{-1}*K^{-1}$) = 0.73378

39
C 1.3328030 3.5412711 -0.4737507
Pd 2.3539323 2.0947783 0.2856751
P 0.6283470 0.5299851 0.0651087
C -3.4909804 2.3601804 1.0703325
C -2.3569975 2.7196292 1.7998192
C -1.1172187 2.1744447 1.4749182
C -1.0073453 1.2520819 0.4252691
C -2.1469022 0.8952969 -0.3060585
C -3.3834529 1.4517552 0.0171251
H -4.4618846 2.7951614 1.3197714
H -2.4351597 3.4360163 2.6209391
H -0.2240482 2.4711150 2.0323038
H -2.0716152 0.1860725 -1.1329573
H -4.2680194 1.1736878 -0.5607665
C 0.1636905 -1.1898132 -4.1959542
C 0.6822871 0.0910465 -4.0028823
C 0.8439273 0.5910753 -2.7124788
C 0.4755533 -0.1843425 -1.6067983
C -0.0460318 -1.4707752 -1.8039425
C -0.1984819 -1.9692269 -3.0964518
H 0.0455158 -1.5849124 -5.2078893
H 0.9717589 0.7023746 -4.8608194
H 1.2681134 1.5875431 -2.5591117
H -0.3345193 -2.0851519 -0.9479432
H -0.6034397 -2.9731740 -3.2443240
C 1.1257437 -3.0956435 2.8758037

C	1.9784341	-2.9191707	1.7841981
C	1.8092347	-1.8304801	0.9344965
C	0.7774219	-0.9086923	1.1681830
C	-0.0774845	-1.0897318	2.2620209
C	0.0996845	-2.1812251	3.1114789
H	1.2616264	-3.9500379	3.5432079
H	2.7828449	-3.6334821	1.5938264
H	2.4810019	-1.7006487	0.0817908
H	-0.8875951	-0.3820525	2.4505819
H	-0.5731437	-2.3173672	3.9614732
C	3.6402728	0.9023237	1.0913672
O	4.3424933	0.1551026	1.6250766
O	0.6196335	4.3549862	-0.8826386

$Pd(PPh_3)_2(CO)_1$ **9-S0**
E (E_h) = -2312.597328
OC (E_h) = 0.000204071
dU_{298} (kJ*mol^{-1}) = 1509.26
dS_{298} (kJ*mol^{-1}*K^{-1}) = 1.06676

71

P	1.1430867	1.6693526	-0.1804303
C	-3.1441860	3.2286167	-0.9654945
C	-2.4882967	2.4127445	-1.8879610
C	-1.2035339	1.9504646	-1.6170120
C	-0.5558420	2.3091939	-0.4274252
C	-1.2218009	3.1211718	0.4971249
C	-2.5115961	3.5782739	0.2272052
H	-4.1577051	3.5818755	-1.1711873
H	-2.9875628	2.1192475	-2.8142614
H	-0.6974818	1.2856013	-2.3240734
H	-0.7351407	3.3949643	1.4363737
H	-3.0265167	4.2086280	0.9566788
C	1.6747422	1.7537388	4.4278770
C	1.3030090	0.5819846	3.7679676
C	1.1636997	0.5782474	2.3811566
C	1.3808300	1.7469156	1.6389219
C	1.7668017	2.9162313	2.3074250
C	1.9104942	2.9179327	3.6947358
H	1.7912882	1.7582498	5.5144851
H	1.1313182	-0.3391297	4.3306077
H	0.9006791	-0.3480541	1.8652103
H	1.9617717	3.8305210	1.7413032
H	2.2119134	3.8354846	4.2062727
C	3.8972364	5.1238031	-1.6012603
C	4.4088025	3.8528229	-1.3288338
C	3.5555713	2.8305235	-0.9217481
C	2.1840833	3.0702695	-0.7596174
C	1.6772329	4.3450041	-1.0369176

C	2.5314569	5.3651716	-1.4598375
H	4.5643244	5.9238677	-1.9318178
H	5.4771017	3.6544033	-1.4465655
H	3.9495761	1.8265009	-0.7368387
H	0.6092127	4.5452376	-0.9246454
H	2.1239148	6.3553891	-1.6789120
Pd	1.5642051	-0.4436744	-1.1314758
P	-0.1956276	-1.9221128	-0.5996338
C	-4.4497944	-0.1126050	-0.2412832
C	-3.4599997	0.1963654	0.6937203
C	-2.1936912	-0.3664777	0.5817142
C	-1.9038257	-1.2730177	-0.4494854
C	-2.8994304	-1.5750205	-1.3847538
C	-4.1639976	-0.9921646	-1.2828021
H	-5.4409608	0.3403646	-0.1605852
H	-3.6697227	0.8962301	1.5055200
H	-1.4215021	-0.0970378	1.3063932
H	-2.6911425	-2.2726617	-2.1990589
H	-4.9310635	-1.2354741	-2.0224796
C	-0.7787024	-5.4626984	-3.5344035
C	-0.1217381	-4.3012437	-3.9395504
C	0.0607926	-3.2530646	-3.0367753
C	-0.4239689	-3.3526516	-1.7285545
C	-1.0848575	-4.5215984	-1.3284093
C	-1.2571972	-5.5725152	-2.2267467
H	-0.9147280	-6.2885215	-4.2372709
H	0.2588644	-4.2125090	-4.9601059
H	0.5937733	-2.3464922	-3.3393143
H	-1.4640519	-4.6140409	-0.3074893
H	-1.7674315	-6.4838404	-1.9048402
C	0.7174973	-3.9158376	3.4871995
C	1.7372686	-3.6004451	2.5876302
C	1.4295156	-3.0052313	1.3663575
C	0.0978017	-2.7259481	1.0251343
C	-0.9199858	-3.0513354	1.9300063
C	-0.6092938	-3.6410248	3.1555440
H	0.9581048	-4.3737058	4.4498926
H	2.7791522	-3.8091687	2.8424849
H	2.2284453	-2.7360427	0.6681524
H	-1.9617198	-2.8343883	1.6832489
H	-1.4115717	-3.8867220	3.8558703
C	3.0931475	-0.9204718	-2.1513167
O	4.0289519	-1.2184663	-2.7510402

$Pd(PPh_3)_2(CO)_1$ **9-T1/S1**

E-T1 (E_h) = -2312.524277

E-S1 (E_h) = -2312.511963

OC (E_h) = -0.000140679

dU_{298} ($kJ*mol^{-1}$) = 1508.06

dS$_{298}$ (kJ*mol^{-1}*K^{-1}) = 1.03625

71

P	0.9948707	1.6131864	-0.1037930
C	-3.1748343	3.4027024	-1.0014789
C	-2.5701082	2.5080252	-1.8845872
C	-1.3211225	1.9709540	-1.5849423
C	-0.6634883	2.3282497	-0.4015775
C	-1.2767767	3.2207172	0.4844577
C	-2.5280577	3.7558455	0.1827592
H	-4.1588818	3.8185172	-1.2321215
H	-3.0796617	2.2149498	-2.8053813
H	-0.8479273	1.2514278	-2.2607762
H	-0.7856974	3.4938922	1.4204983
H	-3.0022586	4.4499001	0.8811454
C	1.6150408	1.8342570	4.4853114
C	1.3148023	0.6210303	3.8653276
C	1.1392642	0.5700341	2.4833813
C	1.2418154	1.7324403	1.7105101
C	1.5566281	2.9464332	2.3379455
C	1.7413654	2.9944041	3.7188568
H	1.7633768	1.8750774	5.5672311
H	1.2305152	-0.2949804	4.4553110
H	0.9456998	-0.3881233	1.9964012
H	1.6696176	3.8573484	1.7443640
H	1.9898133	3.9440499	4.1992034
C	4.0002694	4.7094153	-1.7835969
C	4.4344056	3.6470795	-0.9872735
C	3.5142000	2.7386143	-0.4716912
C	2.1446821	2.8855679	-0.7461611
C	1.7143675	3.9535384	-1.5420471
C	2.6409392	4.8601819	-2.0576327
H	4.7235314	5.4210309	-2.1892437
H	5.4977532	3.5249422	-0.7669234
H	3.8595027	1.9081636	0.1511234
H	0.6513359	4.0794459	-1.7595622
H	2.2952219	5.6914636	-2.6770894
Pd	1.5434289	-0.3446443	-1.1979052
P	-0.1312765	-1.8307385	-0.5712383
C	-4.3340239	0.0491813	-0.1102379
C	-3.3419931	0.2917969	0.8399479
C	-2.0878767	-0.2950021	0.7056877
C	-1.8120520	-1.1458793	-0.3755216
C	-2.8134376	-1.3866032	-1.3245845
C	-4.0662224	-0.7900744	-1.1910292
H	-5.3155587	0.5184558	-0.0095858
H	-3.5395820	0.9545838	1.6851845
H	-1.3193394	-0.0887611	1.4541018
H	-2.6176804	-2.0466185	-2.1721738

H	-4.8388268	-0.9866408	-1.9384471
C	-0.7538285	-5.2868472	-3.5875265
C	-0.4805215	-3.9937946	-4.0346576
C	-0.2755817	-2.9661838	-3.1166460
C	-0.3526746	-3.2209182	-1.7409694
C	-0.6268930	-4.5207708	-1.2973076
C	-0.8236108	-5.5482066	-2.2187052
H	-0.9080314	-6.0944079	-4.3075270
H	-0.4177104	-3.7839901	-5.1052040
H	-0.0417476	-1.9554508	-3.4636775
H	-0.6830870	-4.7352290	-0.2279247
H	-1.0314365	-6.5604021	-1.8630624
C	0.8193651	-3.9123505	3.4574308
C	1.8388088	-3.5616772	2.5702809
C	1.5291226	-2.9341748	1.3662669
C	0.1961062	-2.6638379	1.0305311
C	-0.8246946	-3.0323447	1.9165845
C	-0.5103366	-3.6488179	3.1275808
H	1.0622041	-4.3909374	4.4094515
H	2.8820877	-3.7667064	2.8225599
H	2.3246162	-2.6262389	0.6790615
H	-1.8689293	-2.8355051	1.6645123
H	-1.3117826	-3.9261969	3.8168359
C	2.5668256	-1.6409518	-2.2216119
O	3.0959662	-2.4328974	-2.8984236

$Pd(PPh_3)_3(CO)_1$ **10-S0**
E (E_h) = -3348.295377
OC (E_h) = 8.60413E-05
dU_{298} (kJ*mol^{-1}) = 2252.41
dS_{298} (kJ*mol^{-1}*K^{-1}) = 1.34213

105

P	0.8953457	2.0178729	0.0684542
C	2.9861549	5.3541120	-2.4086020
C	1.9339925	4.6201514	-2.9530110
C	1.3262819	3.6115584	-2.2035428
C	1.7606189	3.3259455	-0.9068845
C	2.8111095	4.0781870	-0.3622731
C	3.4218007	5.0823398	-1.1092146
H	3.4676855	6.1414086	-2.9941540
H	1.5840012	4.8289218	-3.9670892
H	0.5021686	3.0313940	-2.6257552
H	3.1607958	3.8750497	0.6530122
H	4.2425550	5.6587516	-0.6746714
C	-1.2455261	4.6849419	3.2146128
C	-1.8870230	4.2687945	2.0477425
C	-1.2101990	3.4650521	1.1325116
C	0.1129210	3.0716768	1.3635947

C	0.7581790	3.5146532	2.5244188
C	0.0787903	4.3091881	3.4470528
H	-1.7762083	5.3021810	3.9439398
H	-2.9248228	4.5527022	1.8552120
H	-1.7138092	3.1094962	0.2307240
H	1.7923762	3.2265359	2.7248810
H	0.5885563	4.6361458	4.3570083
C	4.3454276	-0.2302497	2.2082701
C	3.1171322	-0.0506182	2.8451661
C	2.0934943	0.6530548	2.2146465
C	2.2894738	1.2109443	0.9434858
C	3.5227559	1.0159273	0.3040800
C	4.5401096	0.2980143	0.9312114
H	5.1458428	-0.7876244	2.7012997
H	2.9428809	-0.4721430	3.8379938
H	1.1334069	0.7665888	2.7195650
H	3.6915158	1.4183407	-0.6963899
H	5.4908670	0.1508351	0.4130637
P	0.6054993	-1.2875369	-1.6901365
C	1.2533443	-4.4007227	1.6900872
C	2.1029445	-3.3005860	1.5700620
C	1.9269815	-2.3850702	0.5351151
C	0.9112569	-2.5677460	-0.4138524
C	0.0532311	-3.6693570	-0.2798758
C	0.2220650	-4.5754619	0.7661762
H	1.3883414	-5.1152612	2.5059453
H	2.9045994	-3.1400747	2.2949000
H	2.5880052	-1.5199597	0.4687546
H	-0.7611868	-3.8199876	-0.9907990
H	-0.4615285	-5.4231344	0.8572835
C	-0.8898516	-3.7596996	-5.3354713
C	-1.4743265	-2.5340913	-5.0218632
C	-1.0256205	-1.8102267	-3.9159913
C	0.0079845	-2.3003850	-3.1141768
C	0.5991436	-3.5280757	-3.4447677
C	0.1508550	-4.2540619	-4.5452305
H	-1.2417713	-4.3315720	-6.1978576
H	-2.2860837	-2.1379629	-5.6371486
H	-1.4812623	-0.8495292	-3.6640674
H	1.4140376	-3.9252134	-2.8343657
H	0.6173276	-5.2117851	-4.7899143
C	4.7810246	-0.0495729	-3.3112223
C	3.6643341	0.7739637	-3.4567301
C	2.4310193	0.3667094	-2.9519118
C	2.2926963	-0.8655036	-2.3027690
C	3.4123516	-1.6975240	-2.1836174
C	4.6497555	-1.2867518	-2.6778607
H	5.7543790	0.2713219	-3.6908939
H	3.7541173	1.7466836	-3.9470867

H 1.5577253 1.0177425 -3.0345442
H 3.3291340 -2.6665622 -1.6867394
H 5.5197417 -1.9388070 -2.5652510
Pd -0.6799942 0.6401086 -1.0918706
P -2.1018426 -0.1648156 0.6559451
C -3.8594334 -4.3280751 -0.4276200
C -3.5315493 -3.3924565 -1.4091531
C -3.0022076 -2.1570727 -1.0412019
C -2.7989774 -1.8356301 0.3056913
C -3.1546750 -2.7698519 1.2858593
C -3.6735739 -4.0109854 0.9192038
H -4.2599690 -5.3048278 -0.7105696
H -3.6684207 -3.6293036 -2.4673798
H -2.7146092 -1.4298574 -1.8039137
H -3.0119135 -2.5405520 2.3440817
H -3.9334033 -4.7377379 1.6932216
C -0.1483746 -0.4878338 4.8529537
C -0.8958823 0.6409149 4.5140895
C -1.5252358 0.7216769 3.2726478
C -1.4097935 -0.3249117 2.3458445
C -0.6336953 -1.4423840 2.6841315
C -0.0192847 -1.5273724 3.9313917
H 0.3392440 -0.5523840 5.8288926
H -0.9917110 1.4703006 5.2190972
H -2.1029424 1.6136410 3.0236298
H -0.5047272 -2.2553217 1.9683314
H 0.5765307 -2.4103506 4.1744563
C -6.0809696 2.1422694 1.3509684
C -5.2748391 2.4333459 0.2520546
C -4.0757127 1.7437399 0.0648359
C -3.6699137 0.7593971 0.9693298
C -4.4935574 0.4640266 2.0650685
C -5.6884556 1.1531175 2.2563725
H -7.0182986 2.6833318 1.5034054
H -5.5762771 3.2025617 -0.4633900
H -3.4386844 1.9687515 -0.7939752
H -4.1973878 -0.3076139 2.7799842
H -6.3195476 0.9162253 3.1167558
C -1.5809065 1.4882954 -2.5379680
O -2.1279245 2.0033051 -3.4157404

$Pd(PPh_3)_3(CO)_1$ **10-T1/S1**
E-T1 (E_h) = -3348.207998
E-S1 (E_h) = -3348.191658
OC (E_h) = 5.55644E-05
dU_{298} (kJ*mol^{-1}) = 2249.59
dS_{298} (kJ*mol^{-1}*K^{-1}) = 1.34799

105

P	0.7718242	2.2082933	0.1561341
C	2.9586468	5.5847642	-2.1602776
C	2.1376421	4.6895157	-2.8421045
C	1.4727051	3.6778453	-2.1453121
C	1.6273304	3.5543525	-0.7625801
C	2.4527611	4.4613629	-0.0810021
C	3.1137390	5.4698263	-0.7759200
H	3.4793261	6.3766039	-2.7047715
H	2.0094034	4.7747253	-3.9241334
H	0.8287101	2.9714485	-2.6799242
H	2.5821834	4.3791973	1.0010803
H	3.7541070	6.1714984	-0.2355338
C	-1.4857044	4.5750444	3.4339703
C	-2.0773722	4.2714562	2.2069076
C	-1.3592563	3.5611243	1.2479774
C	-0.0465796	3.1435109	1.5015174
C	0.5488370	3.4716872	2.7255167
C	-0.1708402	4.1807393	3.6863481
H	-2.0490908	5.1209401	4.1948099
H	-3.1066655	4.5731095	1.9981609
H	-1.8252603	3.3058715	0.2940400
H	1.5728484	3.1613675	2.9429265
H	0.2990775	4.4221030	4.6430450
C	4.3250486	-0.1583839	1.9675863
C	3.1393812	-0.0269075	2.6915593
C	2.0732711	0.7052721	2.1732413
C	2.1899559	1.3465709	0.9314075
C	3.3833683	1.2044218	0.2046320
C	4.4376455	0.4513597	0.7163265
H	5.1562369	-0.7410561	2.3723212
H	3.0305440	-0.5090737	3.6659360
H	1.1470672	0.7747326	2.7438663
H	3.4883197	1.6787275	-0.7730478
H	5.3541102	0.3436963	0.1311362
P	0.5131167	-1.4366136	-1.7391560
C	1.3189072	-4.4290446	1.7042626
C	2.1189110	-3.2964782	1.5383723
C	1.8756521	-2.4132736	0.4908443
C	0.8456641	-2.6651636	-0.4290932
C	0.0339818	-3.7940852	-0.2468383
C	0.2693019	-4.6658159	0.8159360
H	1.5058815	-5.1190088	2.5308133
H	2.9310013	-3.0863856	2.2382377
H	2.4941929	-1.5198165	0.3873781
H	-0.7918690	-3.9940707	-0.9320072
H	-0.3761113	-5.5377714	0.9465575
C	-1.1891645	-3.8409960	-5.3197168
C	-1.6813978	-2.5724027	-5.0161643
C	-1.1708925	-1.8691950	-3.9248105

C	-0.1637461	-2.4263496	-3.1309945
C	0.3395269	-3.6941791	-3.4525063
C	-0.1756084	-4.3993614	-4.5376271
H	-1.5922530	-4.3964505	-6.1702839
H	-2.4690693	-2.1265697	-5.6284755
H	-1.5575440	-0.8759301	-3.6827553
H	1.1361104	-4.1377896	-2.8500953
H	0.2182670	-5.3904179	-4.7765752
C	4.6999855	-0.2884316	-3.3945181
C	3.6087715	0.5684120	-3.5340903
C	2.3623818	0.2034671	-3.0269865
C	2.1913319	-1.0259092	-2.3783173
C	3.2853263	-1.8945649	-2.2659182
C	4.5324549	-1.5230942	-2.7647255
H	5.6810101	0.0025442	-3.7785315
H	3.7273834	1.5356891	-4.0292814
H	1.5053104	0.8762430	-3.1310769
H	3.1733528	-2.8630134	-1.7738741
H	5.3803026	-2.2048488	-2.6601345
Pd	-0.5308555	0.5956587	-1.0404150
P	-1.8564938	-0.2291679	0.7527923
C	-3.7946269	-4.2723721	-0.4316598
C	-3.4847224	-3.3003114	-1.3837456
C	-2.9018028	-2.0999019	-0.9854809
C	-2.6117033	-1.8570814	0.3628763
C	-2.9553908	-2.8218558	1.3167491
C	-3.5365977	-4.0248731	0.9178469
H	-4.2429733	-5.2202591	-0.7395139
H	-3.6835497	-3.4789826	-2.4434161
H	-2.6470817	-1.3370016	-1.7254215
H	-2.7620964	-2.6443285	2.3764302
H	-3.7887886	-4.7767451	1.6699719
C	-0.0453433	-0.5190582	5.0007150
C	-0.7909231	0.6049050	4.6409688
C	-1.3972110	0.6798350	3.3883106
C	-1.2678434	-0.3767107	2.4718284
C	-0.4981310	-1.4942429	2.8346156
C	0.0980573	-1.5677394	4.0895551
H	0.4290947	-0.5735221	5.9835443
H	-0.8988576	1.4394961	5.3379403
H	-1.9606561	1.5754937	3.1205042
H	-0.3539804	-2.3105013	2.1258684
H	0.6906855	-2.4482711	4.3498187
C	-5.7453429	2.2987251	0.9104179
C	-4.8994456	2.3590776	-0.1965532
C	-3.7232057	1.6096971	-0.2187698
C	-3.3806001	0.7979240	0.8684529
C	-4.2498561	0.7169747	1.9639642
C	-5.4209958	1.4709837	1.9876323

H	-6.6645182	2.8896570	0.9316486
H	-5.1533525	2.9948561	-1.0484420
H	-3.0513275	1.6547840	-1.0835986
H	-4.0120489	0.0633179	2.8067771
H	-6.0878094	1.4089476	2.8514174
C	-0.8488674	1.4663902	-2.8661000
O	-1.7646301	1.8511316	-3.5212177

$Pd(PPh_3)_2(CO)_2$ **11-S0**
E (E_h) = -2425.855594
OC (E_h) = 0.000234172
dU_{298} (kJ*mol^{-1}) = 1533.39
dS_{298} (kJ*mol^{-1}*K^{-1}) = 1.07182

73

P	1.1773448	1.6637689	0.2223465
C	-3.1022881	2.8868078	-1.0625574
C	-2.3970254	1.8984236	-1.7506617
C	-1.1142659	1.5487814	-1.3414916
C	-0.5130215	2.1906154	-0.2515709
C	-1.2240014	3.1832186	0.4316453
C	-2.5144529	3.5263398	0.0276061
H	-4.1164423	3.1513659	-1.3718584
H	-2.8567109	1.3820023	-2.5963222
H	-0.5733782	0.7541120	-1.8607720
H	-0.7718366	3.6898616	1.2870132
H	-3.0640206	4.2983245	0.5722088
C	1.6387252	3.7293629	4.3520443
C	1.0592335	2.4641511	4.2417620
C	0.9512458	1.8537203	2.9944263
C	1.4035244	2.5051725	1.8390882
C	1.9821046	3.7748405	1.9577207
C	2.1023633	4.3802735	3.2090511
H	1.7336429	4.2055483	5.3310679
H	0.6993176	1.9444969	5.1331146
H	0.5167348	0.8537201	2.9170087
H	2.3414675	4.2961853	1.0675902
H	2.5604959	5.3692896	3.2890003
C	3.9561901	4.1588766	-2.5267460
C	4.4687495	3.1585176	-1.6979580
C	3.6121656	2.4142613	-0.8912731
C	2.2340545	2.6728794	-0.8885276
C	1.7270931	3.6796220	-1.7177403
C	2.5856680	4.4146225	-2.5369024
H	4.6266979	4.7365796	-3.1678815
H	5.5415500	2.9504020	-1.6874266
H	4.0115228	1.6182852	-0.2559353
H	0.6557314	3.8926009	-1.7254800
H	2.1781198	5.1947825	-3.1848745

Pd 1.7415140 -0.6681784 0.1904232
P -0.2007534 -2.0393279 -0.1178128
C -4.3413841 -0.0855161 0.5806640
C -3.2553748 0.2090301 1.4062658
C -2.0226465 -0.3917821 1.1723176
C -1.8617550 -1.3034395 0.1214143
C -2.9548887 -1.5964678 -0.7011052
C -4.1886196 -0.9871911 -0.4713982
H -5.3063745 0.3976025 0.7525874
H -3.3637880 0.9264095 2.2228969
H -1.1630306 -0.1403973 1.7985516
H -2.8438019 -2.2994467 -1.5295776
H -5.0350714 -1.2175159 -1.1233792
C -0.6312192 -3.9372879 -4.3300263
C -0.1923481 -2.6211851 -4.1769293
C -0.0489051 -2.0787306 -2.9018563
C -0.3583237 -2.8363476 -1.7646421
C -0.7975121 -4.1564184 -1.9265690
C -0.9291511 -4.7035025 -3.2031370
H -0.7348741 -4.3679798 -5.3290350
H 0.0509415 -2.0168434 -5.0542339
H 0.3192906 -1.0560526 -2.7859656
H -1.0386075 -4.7625700 -1.0502542
H -1.2683950 -5.7362330 -3.3163212
C -0.1665788 -5.7233210 2.6975665
C 0.9877211 -5.3174589 2.0242843
C 0.9548479 -4.1995807 1.1950464
C -0.2372521 -3.4839992 1.0134571
C -1.3911983 -3.8970302 1.6887112
C -1.3525689 -5.0095281 2.5309397
H -0.1390334 -6.5949499 3.3562631
H 1.9217764 -5.8698078 2.1539435
H 1.8636792 -3.8692321 0.6834300
H -2.3265056 -3.3481272 1.5573228
H -2.2584623 -5.3204989 3.0574770
C 2.8007771 -0.8802435 -1.4247250
O 3.3567007 -0.9687406 -2.4230466
C 2.3226455 -1.1133684 1.9912987
O 2.5722303 -1.3765541 3.0784384

$Pd(PPh_3)_2(CO)_2$ **11-T1/S1**
E-T1 (E_h) = -2425.777181
E-S1 (E_h) = -2425.761574
OC (E_h) = 0.000183709
dU_{298} ($kJ*mol^{-1}$) = 1533.28
dS_{298} ($kJ*mol^{-1}*K^{-1}$) = 1.11293

73
P 1.2909712 1.7890359 -0.1453972

C	-2.1363195	2.1462658	-3.2345835
C	-0.8193322	1.9335765	-3.6490819
C	0.2097151	1.8778480	-2.7129950
C	-0.0623591	2.0547640	-1.3464234
C	-1.3833902	2.2766100	-0.9382465
C	-2.4129186	2.3171259	-1.8789083
H	-2.9445593	2.1791008	-3.9690827
H	-0.5914017	1.8014291	-4.7096304
H	1.2331905	1.6919244	-3.0483218
H	-1.6170543	2.4133632	0.1192340
H	-3.4397158	2.4866548	-1.5453337
C	-0.0926249	3.9313012	3.7125339
C	-0.5912800	2.6744523	3.3686554
C	-0.1554653	2.0428041	2.2058133
C	0.7667539	2.6779440	1.3593614
C	1.2683586	3.9367451	1.7121468
C	0.8409816	4.5572279	2.8858156
H	-0.4266735	4.4213309	4.6304365
H	-1.3166171	2.1721739	4.0131323
H	-0.5296410	1.0463442	1.9594451
H	1.9991872	4.4365063	1.0727190
H	1.2414584	5.5378820	3.1543308
C	4.9021990	4.1617855	-1.7691433
C	5.0835080	2.9467929	-1.1089603
C	3.9781476	2.2377082	-0.6396462
C	2.6865495	2.7450108	-0.8218019
C	2.5091701	3.9649632	-1.4900431
C	3.6149574	4.6688811	-1.9612032
H	5.7674440	4.7154852	-2.1421338
H	6.0888366	2.5439797	-0.9647079
H	4.1073740	1.2750605	-0.1349898
H	1.5031288	4.3626531	-1.6464609
H	3.4720033	5.6185698	-2.4824687
Pd	1.5592872	-0.6043803	0.1186664
P	-0.2734387	-2.1790159	0.2242053
C	-3.4327326	0.0025002	2.8083799
C	-2.3800751	-0.6963153	3.4042901
C	-1.4645768	-1.3894733	2.6171532
C	-1.6013196	-1.4101461	1.2193902
C	-2.6639705	-0.7153229	0.6287327
C	-3.5702620	-0.0102139	1.4211577
H	-4.1443777	0.5551070	3.4264258
H	-2.2652155	-0.6954631	4.4910287
H	-0.6343948	-1.9163494	3.0944522
H	-2.7868001	-0.7160593	-0.4558843
H	-4.3922519	0.5310727	0.9460865
C	-2.2004067	-3.0431489	-3.8900671
C	-1.7771557	-1.7581318	-3.5491410
C	-1.1924437	-1.5203984	-2.3071599

C -1.0501290 -2.5655403 -1.3809853
C -1.4714528 -3.8543329 -1.7302781
C -2.0411594 -4.0899187 -2.9813707
H -2.6488752 -3.2311017 -4.8687289
H -1.8898767 -0.9329508 -4.2562675
H -0.8375377 -0.5170426 -2.0602786
H -1.3520269 -4.6813230 -1.0271417
H -2.3629231 -5.1001679 -3.2460127
C 0.5668492 -6.2698351 2.1900832
C 1.5997850 -5.5232965 1.6238349
C 1.3274778 -4.2825071 1.0484394
C 0.0189161 -3.7864534 1.0300641
C -1.0155924 -4.5388636 1.6044325
C -0.7397352 -5.7760632 2.1819016
H 0.7800753 -7.2398245 2.6461523
H 2.6239751 -5.9035723 1.6362402
H 2.1327616 -3.6803322 0.6159159
H -2.0384563 -4.1535870 1.6042104
H -1.5486842 -6.3586418 2.6294240
C 2.0499693 -0.8895433 -1.7743527
O 2.7358919 -1.2259132 -2.6508877
C 1.8658935 -0.6358345 2.0709090
O 2.4704056 -0.8409161 3.0429197

cis-PdL2chexI **cis-12**
E (E_h) = -2732.186175
OC (E_h) = -0.000790599
dU_{298} (kJ*mol^{-1}) = 1913.36
dS_{298} (kJ*mol^{-1}*K^{-1}) = 1.14443

87
P 1.6881278 -1.3213003 -0.6631283
P -0.8906995 1.0706952 0.1538799
C -5.2155926 -1.4329753 -0.4964913
C -3.8265624 -0.9531163 -0.9329871
C -2.7589341 -1.4747783 0.0072709
C -3.0265544 -1.1525182 1.4675880
C -4.4224927 -1.6326130 1.8812272
C -5.4959797 -1.0687520 0.9568643
H -5.2745509 -2.5305248 -0.6130498
H -5.9866744 -1.0046901 -1.1590530
H -3.8290514 0.1468235 -0.9528084
H -3.6122638 -1.2931835 -1.9586879
H -2.7343484 -2.5722606 -0.0864229
H -2.2640045 -1.6199506 2.1124623
H -2.9689161 -0.0693674 1.6528600
H -4.4531127 -2.7366246 1.8419867
H -4.6208248 -1.3498322 2.9290538
H -5.5169308 0.0320923 1.0545678

H	-6.4925772	-1.4303397	1.2609161
C	2.6760293	-5.6513959	0.6521082
C	1.5943702	-4.9631305	1.2040818
C	1.3002623	-3.6715908	0.7749649
C	2.0961091	-3.0459684	-0.1937540
C	3.1790469	-3.7390791	-0.7411720
C	3.4633645	-5.0392242	-0.3226240
H	2.9012072	-6.6698320	0.9783275
H	0.9674233	-5.4394537	1.9616606
H	0.4318357	-3.1475722	1.1860248
H	3.8012315	-3.2688002	-1.5057255
H	4.3067345	-5.5765319	-0.7634973
C	3.8783987	-0.6411015	-4.6926323
C	2.5306966	-1.0007646	-4.6635633
C	1.8864204	-1.2023662	-3.4447556
C	2.5841023	-1.0515454	-2.2397120
C	3.9409490	-0.7032466	-2.2765921
C	4.5821821	-0.4961986	-3.4970358
H	4.3817282	-0.4748791	-5.6483982
H	1.9730464	-1.1208821	-5.5956548
H	0.8315533	-1.4881184	-3.4242532
H	4.5026621	-0.5852339	-1.3475375
H	5.6395748	-0.2206235	-3.5119114
C	4.0178137	1.1734967	2.4866696
C	3.6742148	1.7309122	1.2552196
C	3.0217963	0.9631497	0.2941480
C	2.6921543	-0.3745597	0.5531031
C	3.0609306	-0.9327477	1.7840892
C	3.7186296	-0.1634249	2.7423476
H	4.5257067	1.7781969	3.2419114
H	3.9153683	2.7739908	1.0356627
H	2.7813556	1.4076530	-0.6718505
H	2.8322465	-1.9773984	2.0036460
H	3.9955149	-0.6154726	3.6979616
C	-4.8750377	3.3366610	-0.5776833
C	-3.9924546	3.0766607	-1.6270418
C	-2.7851690	2.4281600	-1.3818418
C	-2.4418413	2.0269366	-0.0824349
C	-3.3291853	2.2997741	0.9658587
C	-4.5375214	2.9506029	0.7182030
H	-5.8233546	3.8440377	-0.7703423
H	-4.2453421	3.3790361	-2.6460189
H	-2.1082592	2.2270896	-2.2151667
H	-3.0836390	2.0085900	1.9885687
H	-5.2180652	3.1554662	1.5480982
C	0.3944836	1.7892107	4.5369811
C	0.5110113	0.5306951	3.9510864
C	0.1036850	0.3359180	2.6331060
C	-0.4159517	1.3970836	1.8849806

C -0.5458098 2.6585110 2.4873441
C -0.1385548 2.8516665 3.8047536
H 0.7121973 1.9435336 5.5711111
H 0.9215070 -0.3062286 4.5200990
H 0.1849713 -0.6508874 2.1716159
H -0.9826960 3.4934242 1.9353511
H -0.2431835 3.8375688 4.2639770
C 1.9649207 3.4657346 -2.6044763
C 1.6933568 3.9513930 -1.3232646
C 0.8381546 3.2536930 -0.4738661
C 0.2461309 2.0581138 -0.8984890
C 0.5055080 1.5887493 -2.1909697
C 1.3646466 2.2857907 -3.0398649
H 2.6485120 4.0080393 -3.2621534
H 2.1626003 4.8752953 -0.9765649
H 0.6646585 3.6315328 0.5339636
H 0.0473545 0.6540534 -2.5252846
H 1.5770679 1.8904901 -4.0356566
Pd -0.7755661 -1.1248105 -0.5246968
I -0.9818524 -3.3246263 -2.1265868

trans-PdL2chexI **trans-12**
E (E_h) = -2732.191107
OC (E_h) = -0.000697558
dU_{298} ($kJ*mol^{-1}$) = 1913.34
dS_{298} ($kJ*mol^{-1}*K^{-1}$) = 1.1747

87
P 1.1477503 2.1544428 -0.3824900
C -0.6792466 4.4611308 -3.9567090
C -0.7775887 3.0717910 -3.8952281
C -0.2145373 2.3758662 -2.8259919
C 0.4501371 3.0662869 -1.8064278
C 0.5309282 4.4661994 -1.8648673
C -0.0245106 5.1576030 -2.9390469
H -1.1192797 5.0055425 -4.7959710
H -1.2951189 2.5209278 -4.6845792
H -0.2804575 1.2869547 -2.7848349
H 1.0171715 5.0228216 -1.0599655
H 0.0484328 6.2471322 -2.9775667
C -0.2284074 4.7046767 3.2325633
C -1.1132461 4.3967150 2.1972984
C -0.6732116 3.6691298 1.0952281
C 0.6598264 3.2405593 1.0136464
C 1.5430041 3.5562193 2.0519353
C 1.0988476 4.2859854 3.1553333
H -0.5745703 5.2749168 4.0980399
H -2.1541584 4.7251263 2.2484004
H -1.3727252 3.4347141 0.2887777

H	2.5852981	3.2335352	2.0028175
H	1.7990113	4.5294970	3.9581758
C	5.7556377	2.5124486	-0.3815057
C	5.1016170	1.5513522	0.3911648
C	3.7132071	1.4560190	0.3526814
C	2.9645932	2.3355539	-0.4410472
C	3.6234323	3.2935580	-1.2158548
C	5.0156226	3.3769065	-1.1876579
H	6.8461208	2.5812002	-0.3619736
H	5.6762453	0.8627502	1.0152432
H	3.2009135	0.6804212	0.9300246
H	3.0566099	3.9696610	-1.8585854
H	5.5242758	4.1222445	-1.8039659
P	-0.4554604	-2.2086243	-0.6319762
C	-3.9594572	-3.8161159	1.9699063
C	-2.6390178	-4.1389111	2.2821142
C	-1.5971052	-3.6855655	1.4759697
C	-1.8583135	-2.8969905	0.3458381
C	-3.1888969	-2.5903127	0.0326537
C	-4.2303200	-3.0462432	0.8394479
H	-4.7766736	-4.1716249	2.6021708
H	-2.4148928	-4.7518436	3.1585170
H	-0.5706006	-3.9608826	1.7277032
H	-3.4234750	-1.9931072	-0.8501816
H	-5.2617882	-2.7979751	0.5777760
C	-2.2346399	-1.7326271	-4.8797334
C	-2.4758461	-0.7897874	-3.8793483
C	-1.9340870	-0.9701347	-2.6093133
C	-1.1587800	-2.1028137	-2.3200946
C	-0.9160779	-3.0400867	-3.3269815
C	-1.4527306	-2.8528195	-4.6008427
H	-2.6493057	-1.5885981	-5.8804354
H	-3.0778865	0.0973806	-4.0910438
H	-2.0939176	-0.2101222	-1.8382546
H	-0.2854440	-3.9088815	-3.1320223
H	-1.2486318	-3.5870063	-5.3840917
C	2.5295306	-5.7306192	-0.4133747
C	2.9449052	-4.4319039	-0.1227259
C	2.0414776	-3.3731719	-0.2124416
C	0.7147329	-3.6083043	-0.5884134
C	0.2973098	-4.9201221	-0.8581382
C	1.2041037	-5.9741765	-0.7798760
H	3.2385511	-6.5596884	-0.3472915
H	3.9785957	-4.2382122	0.1733169
H	2.3644590	-2.3517510	0.0022651
H	-0.7451708	-5.1234825	-1.1156351
H	0.8728244	-6.9921812	-0.9985626
Pd	0.5062489	-0.0785883	-0.2925929
I	2.0578892	-0.5170474	-2.5641382

C	-0.2969524	-0.5843701	3.9634589
C	0.2105215	-0.8500323	2.5418254
C	-0.2937760	0.2198946	1.5863257
C	-1.8042245	0.3715469	1.6119793
C	-2.2989191	0.6285808	3.0381162
C	-1.8167915	-0.4597223	3.9909274
H	0.1515220	0.3535459	4.3389405
H	0.0398216	-1.3878414	4.6403785
H	-0.1379494	-1.8460590	2.2305641
H	1.3130236	-0.8843477	2.5345404
H	0.1358350	1.1732280	1.9273095
H	-2.1153227	1.1971975	0.9514448
H	-2.2910521	-0.5390029	1.2308484
H	-1.9190301	1.6091755	3.3786099
H	-3.4002473	0.6936110	3.0508745
H	-2.2634311	-1.4249859	3.6894742
H	-2.1667613	-0.2573655	5.0171520

PdL3I* **13**
E (E_h) = -3532.786844
OC (E_h) = -0.000896015
dU_{298} (kJ*mol^{-1}) = 2231.26
dS_{298} (kJ*mol^{-1}*K^{-1}) = 1.36873

104

P	1.3426713	2.2874817	0.1823131
C	1.1185310	4.0063421	-4.1209324
C	0.3564244	2.8972631	-3.7591240
C	0.4462126	2.3867371	-2.4642281
C	1.2973886	2.9672003	-1.5222858
C	2.0377499	4.1029404	-1.8851407
C	1.9549019	4.6114024	-3.1791089
H	1.0525876	4.4115808	-5.1337732
H	-0.3152326	2.4265876	-4.4817027
H	-0.1582730	1.5277100	-2.1759590
H	2.6730302	4.6006397	-1.1481479
H	2.5403342	5.4926448	-3.4530165
C	0.8520243	6.2602472	2.5115866
C	-0.1731489	5.7585384	1.7055327
C	0.0053699	4.5639731	1.0165528
C	1.2124094	3.8597010	1.1172782
C	2.2366048	4.3665034	1.9196193
C	2.0542407	5.5630862	2.6149845
H	0.7108970	7.1956065	3.0589533
H	-1.1191556	6.2993997	1.6211127
H	-0.8034890	4.1690648	0.3944268
H	3.1799701	3.8238937	2.0104898
H	2.8605237	5.9509271	3.2428934
C	5.6894977	0.9607621	1.0858179

C	4.6434633	0.6781073	1.9648808
C	3.3441664	1.0725062	1.6554752
C	3.0776094	1.7684181	0.4658105
C	4.1271577	2.0311498	-0.4194728
C	5.4257408	1.6297844	-0.1077645
H	6.7089547	0.6484293	1.3260907
H	4.8380484	0.1442719	2.8985659
H	2.5230649	0.8556247	2.3457064
H	3.9362730	2.5378251	-1.3663135
H	6.2354858	1.8353286	-0.8119234
P	0.6914974	-1.0821602	-0.8492109
C	1.0021682	-5.2157659	1.2130941
C	1.2929355	-4.0344282	1.8954710
C	1.1584783	-2.8074985	1.2487055
C	0.7455842	-2.7495003	-0.0905009
C	0.4411486	-3.9365408	-0.7628776
C	0.5704807	-5.1620313	-0.1118512
H	1.0997767	-6.1793935	1.7191670
H	1.6193989	-4.0660433	2.9379152
H	1.3709367	-1.8756845	1.7842635
H	0.0798852	-3.9123473	-1.7916935
H	0.3198559	-6.0829167	-0.6437004
C	-1.9411306	-1.2761805	-4.6580446
C	-2.2973458	-0.4621592	-3.5842737
C	-1.5015851	-0.4361923	-2.4397613
C	-0.3389773	-1.2100022	-2.3573368
C	0.0237449	-2.0069792	-3.4529862
C	-0.7796489	-2.0484889	-4.5900351
H	-2.5634465	-1.3024661	-5.5559626
H	-3.1993555	0.1527237	-3.6332315
H	-1.7810740	0.1963900	-1.5914626
H	0.9508300	-2.5843616	-3.4282464
H	-0.4904948	-2.6787867	-5.4345067
C	4.9688426	-0.6501517	-2.6093394
C	3.8793218	-0.0792459	-3.2645619
C	2.5943260	-0.2201469	-2.7444988
C	2.3793764	-0.9391697	-1.5599850
C	3.4835554	-1.4916168	-0.8962220
C	4.7653745	-1.3513859	-1.4207450
H	5.9761768	-0.5425675	-3.0188929
H	4.0252383	0.4797558	-4.1923015
H	1.7538680	0.2248193	-3.2784739
H	3.3443159	-2.0465737	0.0334238
H	5.6133239	-1.7923975	-0.8916080
Pd	-0.0900987	0.4364670	0.7973869
P	-2.1365358	-0.8176982	1.3492841
C	-3.1051841	-4.9354886	-0.5981603
C	-3.3564553	-3.7511569	-1.2895697
C	-3.0993850	-2.5211736	-0.6889642

C -2.5732229 -2.4485254 0.6084156
C -2.3284221 -3.6453234 1.2952521
C -2.5963177 -4.8748685 0.6982225
H -3.3097520 -5.9016735 -1.0660239
H -3.7634685 -3.7784466 -2.3035503
H -3.3318948 -1.6098834 -1.2372175
H -1.9279314 -3.6264499 2.3093137
H -2.3971656 -5.7941966 1.2538769
C -2.7576985 -1.7795500 5.8404699
C -3.7787218 -1.1462821 5.1336835
C -3.6153122 -0.8423906 3.7814003
C -2.4229108 -1.1621374 3.1274822
C -1.3903795 -1.7742629 3.8507410
C -1.5619767 -2.0959032 5.1937086
H -2.8893100 -2.0188407 6.8987038
H -4.7134507 -0.8845396 5.6359879
H -4.4223885 -0.3424001 3.2425641
H -0.4386783 -1.9864079 3.3593999
H -0.7500465 -2.5776945 5.7440535
C -5.6152691 1.9792446 0.0997499
C -4.3184415 2.4691762 0.2510255
C -3.2873208 1.6080703 0.6238118
C -3.5417955 0.2491561 0.8513167
C -4.8496093 -0.2342203 0.7015727
C -5.8788588 0.6272041 0.3268029
H -6.4240705 2.6516956 -0.1969629
H -4.1065139 3.5267945 0.0754686
H -2.2661526 1.9844748 0.7473265
H -5.0654427 -1.2914089 0.8728658
H -6.8941457 0.2396862 0.2115925
I -0.1650891 1.5467691 3.4100426

Cyclohexane radical **14**
E (E_h) = -235.0272478
OC (E_h) = 4.1451E-05
dU_{298} (kJ*mol^{-1}) = 406.39
dS_{298} (kJ*mol^{-1}*K^{-1}) = 0.32079

17
C 1.1195020 0.2266972 -0.8829386
C -0.3471781 0.2651608 -1.2996673
C -1.2339888 0.7339696 -0.1507422
C -1.0705656 -0.1590753 1.0825676
C 0.3611076 -0.3871075 1.4289418
C 1.3314923 -0.6767917 0.3352837
H 1.4470198 1.2515012 -0.6326246
H 1.7526641 -0.1117431 -1.7191652
H -0.6639610 -0.7458452 -1.6173558
H -0.4780053 0.9219738 -2.1752644

H	-2.2912087	0.7597983	-0.4610649
H	-0.9568751	1.7696650	0.1151914
H	-1.6211663	0.2571353	1.9420151
H	-1.5557001	-1.1378438	0.8660327
H	0.6298864	-0.6311368	2.4618856
H	1.2081546	-1.7335195	0.0061948
H	2.3688223	-0.6028383	0.7007104

PdL2I* **15**
E (E_h) = -2497.108869
OC (E_h) = -0.000967289
dU_{298} (kJ*mol^{-1}) = 1491.84
dS_{298} (kJ*mol^{-1}*K^{-1}) = 1.05915

70

P	-0.6413466	1.1974679	1.3878073
C	-0.9780458	4.7707124	-1.5327925
C	-1.0372453	3.4597929	-2.0057102
C	-0.9466262	2.3923889	-1.1136915
C	-0.7833553	2.6276780	0.2577194
C	-0.7414125	3.9472809	0.7287191
C	-0.8352747	5.0124254	-0.1652232
H	-1.0553867	5.6083095	-2.2302960
H	-1.1669313	3.2637177	-3.0727906
H	-1.0252005	1.3675307	-1.4845558
H	-0.6454878	4.1440974	1.7996431
H	-0.8019487	6.0382680	0.2096336
C	-2.6669255	2.7234888	5.2593091
C	-3.4173308	2.4430555	4.1146155
C	-2.7857646	1.9857051	2.9620704
C	-1.3926218	1.8180387	2.9379195
C	-0.6452386	2.0975968	4.0871887
C	-1.2841468	2.5477345	5.2437252
H	-3.1641145	3.0751552	6.1666106
H	-4.5021707	2.5729776	4.1231171
H	-3.3793333	1.7503576	2.0731174
H	0.4389545	1.9653719	4.0815393
H	-0.6935904	2.7629463	6.1376540
C	3.8139018	0.7156629	2.4909234
C	2.9282507	-0.3582233	2.5863210
C	1.5919102	-0.1902567	2.2349516
C	1.1304397	1.0556203	1.7918543
C	2.0223729	2.1289745	1.6942705
C	3.3605107	1.9567398	2.0444480
H	4.8654753	0.5813676	2.7560316
H	3.2829535	-1.3356133	2.9207676
H	0.8984356	-1.0350181	2.2855488
H	1.6780253	3.1011224	1.3347678
H	4.0541463	2.7970390	1.9617268

Pd	-1.7612262	-0.6844884	0.6724688
P	-0.3873196	-1.6473930	-0.9435406
C	-0.7937056	-6.2485239	-1.0135933
C	-0.8346424	-5.5479073	0.1926410
C	-0.7335081	-4.1585381	0.1948615
C	-0.5719546	-3.4623767	-1.0093654
C	-0.5310796	-4.1680069	-2.2172283
C	-0.6454858	-5.5575698	-2.2169055
H	-0.8847093	-7.3375571	-1.0164889
H	-0.9602897	-6.0842462	1.1361990
H	-0.7941257	-3.6061616	1.1365270
H	-0.4137760	-3.6321784	-3.1621875
H	-0.6194545	-6.1031855	-3.1633200
C	-1.7022934	-0.1783155	-5.1290311
C	-2.6352531	-0.3714683	-4.1089052
C	-2.2184092	-0.8116515	-2.8549377
C	-0.8610873	-1.0693914	-2.6124685
C	0.0700388	-0.8832118	-3.6423035
C	-0.3517596	-0.4365219	-4.8942066
H	-2.0290909	0.1750111	-6.1101591
H	-3.6945830	-0.1720445	-4.2872322
H	-2.9476534	-0.9537387	-2.0512545
H	1.1291770	-1.0838005	-3.4677352
H	0.3817444	-0.2911914	-5.6910476
C	4.1792639	-0.9516085	-0.7016509
C	3.3129313	0.1063054	-0.9820782
C	1.9429268	-0.1168173	-1.0623167
C	1.4208408	-1.4064965	-0.8825346
C	2.2948091	-2.4631701	-0.6026278
C	3.6674851	-2.2331669	-0.5099243
H	5.2547302	-0.7733489	-0.6280141
H	3.7050217	1.1155347	-1.1248335
H	1.2754856	0.7230480	-1.2701944
H	1.9053052	-3.4732510	-0.4599385
H	4.3397830	-3.0660244	-0.2898832
I	-3.9840144	-2.1360603	0.7062688

cis-PdL2COI **16**
E (E_h) = -2610.360486
OC (E_h) = -0.000861316
dU_{298} (kJ*mol^{-1}) = 1518.61
dS_{298} (kJ*mol^{-1}*K^{-1}) = 1.09713

72

P	0.7477934	-1.7328917	-0.4345674
P	-1.6193108	1.0248587	0.2707930
C	-3.2101566	-1.6719610	0.9574240
C	1.7453893	-6.1845754	0.3496049
C	0.5932883	-5.6105478	0.8869007

C	0.2890357	-4.2769451	0.6192764
C	1.1437323	-3.5028532	-0.1739764
C	2.2984181	-4.0835957	-0.7114000
C	2.5950276	-5.4204854	-0.4517973
H	1.9793171	-7.2330824	0.5502949
H	-0.0801698	-6.2060960	1.5078573
H	-0.6264934	-3.8320179	1.0188701
H	2.9684492	-3.4938277	-1.3412166
H	3.4952809	-5.8680883	-0.8799962
C	2.6613392	-0.7868421	-4.5418795
C	1.3385860	-1.2218477	-4.4464952
C	0.7796656	-1.4930772	-3.1997809
C	1.5414968	-1.3365318	-2.0342103
C	2.8734832	-0.9161053	-2.1367501
C	3.4270328	-0.6376843	-3.3858629
H	3.0967957	-0.5657015	-5.5195286
H	0.7340129	-1.3462807	-5.3482036
H	-0.2583163	-1.8313056	-3.1249800
H	3.4851608	-0.7982536	-1.2399767
H	4.4652525	-0.3037684	-3.4539667
C	3.1069562	0.4805182	2.8774182
C	2.7950743	1.1311952	1.6838550
C	2.1379081	0.4488179	0.6633656
C	1.7844034	-0.8986635	0.8229703
C	2.1095752	-1.5500009	2.0206064
C	2.7664536	-0.8622841	3.0401433
H	3.6168568	1.0189252	3.6798252
H	3.0595547	2.1821651	1.5437830
H	1.9024604	0.9708756	-0.2647159
H	1.8462582	-2.6007089	2.1619597
H	3.0134926	-1.3828853	3.9686533
C	-5.8309193	2.6425483	-0.7636270
C	-4.7825075	2.8213015	-1.6654940
C	-3.5005458	2.3700863	-1.3528952
C	-3.2566548	1.7324828	-0.1302393
C	-4.3156619	1.5540134	0.7730633
C	-5.5926854	2.0101607	0.4580853
H	-6.8342235	2.9977128	-1.0106833
H	-4.9595450	3.3199735	-2.6215117
H	-2.6873277	2.5230593	-2.0656253
H	-4.1422583	1.0666939	1.7357202
H	-6.4074741	1.8699401	1.1724773
C	-0.7642759	2.3023132	4.6227595
C	-0.3476523	1.0579147	4.1525050
C	-0.6309380	0.6774773	2.8417344
C	-1.3238134	1.5452497	1.9908783
C	-1.7486886	2.7931607	2.4703721
C	-1.4676697	3.1680160	3.7819713
H	-0.5463986	2.5992921	5.6516699

H 0.1998195 0.3757642 4.8066845
H -0.3115468 -0.2996400 2.4724072
H -2.3071989 3.4706017 1.8194299
H -1.8017363 4.1406408 4.1511714
C 1.4219196 3.2729599 -2.3786959
C 1.1684901 3.7477730 -1.0901306
C 0.2336138 3.1098593 -0.2769325
C -0.4531303 1.9856842 -0.7514597
C -0.2032675 1.5206115 -2.0487766
C 0.7292397 2.1625031 -2.8607054
H 2.1662745 3.7681847 -3.0068778
H 1.7107624 4.6170424 -0.7102286
H 0.0620945 3.4725760 0.7382658
H -0.7245751 0.6327149 -2.4156952
H 0.9297204 1.7761181 -3.8624568
Pd -1.5975355 -1.3229460 -0.0944705
I -2.4472608 -3.2829288 -1.8962488
O -4.2395474 -1.8073629 1.4292622

PdLCOI **17**
E (E_h) = -1574.660648
OC (E_h) = -0.00056328
dU_{298} (kJ*mol^{-1}) = 776.37
dS_{298} (kJ*mol^{-1}*K^{-1}) = 0.76195

38
P -0.7757597 0.1755618 -0.0257898
C -4.8314123 1.5443653 0.0960658
C 0.1384706 -4.2372777 0.9402147
C -0.9314791 -3.5812158 1.5518868
C -1.2098611 -2.2551682 1.2320944
C -0.4082811 -1.5740675 0.3072441
C 0.6622981 -2.2332663 -0.3058729
C 0.9317538 -3.5645059 0.0109963
H 0.3506685 -5.2808932 1.1851915
H -1.5598183 -4.1088207 2.2732271
H -2.0606307 -1.7475197 1.6949088
H 1.2858731 -1.7095431 -1.0342915
H 1.7662581 -4.0777141 -0.4726776
C 1.4531222 1.0845473 -3.9659611
C 0.1927586 0.4843293 -3.9830636
C -0.4773786 0.2305098 -2.7888016
C 0.1180677 0.5701077 -1.5658288
C 1.3820539 1.1721062 -1.5509074
C 2.0449658 1.4284517 -2.7507613
H 1.9747147 1.2876536 -4.9044870
H -0.2754082 0.2162191 -4.9331307
H -1.4668239 -0.2362889 -2.8022979
H 1.8517624 1.4418497 -0.6022713

H 3.0308227 1.8992701 -2.7335804
C 1.4373183 2.6108669 3.2164869
C 0.5221496 3.2247724 2.3587092
C -0.1406558 2.4708896 1.3941284
C 0.1172714 1.0974624 1.2721107
C 1.0337877 0.4856155 2.1342177
C 1.6896301 1.2439015 3.1042948
H 1.9525461 3.2010607 3.9781811
H 0.3188186 4.2946576 2.4458159
H -0.8627744 2.9535038 0.7277181
H 1.2369262 -0.5842836 2.0493071
H 2.4033997 0.7609288 3.7759092
Pd -3.0485541 0.7876937 -0.0422609
I -3.9400602 -1.3812139 -1.4171462
O -5.9065406 1.9054540 0.1304210

PdLI(CO)2* **18**
E (E_h) = -1687.916887
OC (E_h) = -0.000755382
dU_{298} (kJ*mol^{-1}) = 802.56
dS_{298} (kJ*mol^{-1}*K^{-1}) = 0.81161

40
C 1.1634267 3.4799728 -0.6240664
Pd 2.1891008 2.0708791 0.2943012
P 0.5057385 0.4136470 0.0117501
C -3.5793068 2.2894258 1.0595321
C -2.4308089 2.6310747 1.7749544
C -1.2050590 2.0635755 1.4358108
C -1.1247969 1.1356365 0.3870356
C -2.2781552 0.7977160 -0.3303022
C -3.5004072 1.3775839 0.0067167
H -4.5391779 2.7423623 1.3191368
H -2.4855166 3.3510885 2.5946292
H -0.3021812 2.3503520 1.9849104
H -2.2253808 0.0872906 -1.1579584
H -4.3967376 1.1149144 -0.5600674
C 0.1327913 -1.3336706 -4.2390157
C 0.7610696 -0.1054470 -4.0348394
C 0.8888886 0.4061423 -2.7444205
C 0.3803205 -0.3071938 -1.6525287
C -0.2436047 -1.5458920 -1.8610473
C -0.3665135 -2.0538307 -3.1520861
H 0.0372525 -1.7366029 -5.2501926
H 1.1618741 0.4554257 -4.8821863
H 1.3978198 1.3601634 -2.5838529
H -0.6303019 -2.1163814 -1.0129364
H -0.8537533 -3.0188052 -3.3100681
C 1.0733313 -3.1774619 2.8554606

C 1.8844428 -3.0253508 1.7288663
C 1.6905560 -1.9486221 0.8690699
C 0.6734102 -1.0165812 1.1257365
C -0.1389082 -1.1724852 2.2549102
C 0.0642160 -2.2512646 3.1152470
H 1.2288970 -4.0218445 3.5311375
H 2.6749301 -3.7495950 1.5186957
H 2.3276303 -1.8418620 -0.0133284
H -0.9366470 -0.4563074 2.4633477
H -0.5760785 -2.3680629 3.9927539
C 3.6564422 0.8340880 0.7414632
O 4.3882795 0.0879341 1.1879346
O 0.4144446 4.2680634 -0.9567891
I 3.1184726 3.9299251 2.1122856

PdI(CO)2* **19**
E (E_h) = -652.2121162
OC (E_h) = -0.000579246
dU_{298} (kJ*mol^{-1}) = 61.07
dS_{298} (kJ*mol^{-1}*K^{-1}) = 0.40772

6
C -2.84800 1.85360 -0.82300
Pd -1.78190 0.60480 0.19680
C -0.53230 -0.79250 0.74450
O 0.17550 -1.54340 1.26560
O -3.54970 2.66780 -1.24830
I -0.58580 2.27330 0.96740

PdL1Ichex **24**
E (E_h) = -1696.494677
OC (E_h) = -0.00096748
dU_{298} (kJ*mol^{-1}) = 1166.68
dS_{298} (kJ*mol^{-1}*K^{-1}) = 0.80197

53
P 0.5678394 -0.5843814 0.1114913
C -4.5300021 0.2472254 1.0968523
C -3.3144119 -0.1489179 1.9252423
C -2.0819812 0.6344053 1.6065347
C -2.2920682 1.7904072 0.6632688
C -3.0486134 1.4195781 -0.6131023
C -4.1504619 0.3690840 -0.3742969
H -4.9095538 1.2117251 1.4762683
H -5.3390097 -0.4862112 1.2325715
H -3.1393177 -1.2877663 1.6940320
H -3.5119050 -0.2196231 3.0058954
H -1.4512886 0.8842326 2.4709565
H -2.8729476 2.5485725 1.2247335

H -1.3371409 2.2767533 0.4225323
H -3.4793844 2.3360106 -1.0460501
H -2.3316901 1.0388161 -1.3587505
H -3.7997752 -0.6238695 -0.7116864
H -5.0410266 0.5972415 -0.9792295
C 1.1010399 -1.5449736 -4.3763664
C 2.2286133 -1.3906048 -3.5676992
C 2.0832054 -1.1226148 -2.2082613
C 0.8043067 -1.0097689 -1.6488163
C -0.3228752 -1.1774720 -2.4609144
C -0.1742644 -1.4382221 -3.8223435
H 1.2183177 -1.7562795 -5.4421174
H 3.2290190 -1.4816278 -3.9974672
H 2.9715805 -1.0065114 -1.5832166
H -1.3203028 -1.1197760 -2.0174387
H -1.0596414 -1.5685892 -4.4489374
C 1.3685270 3.9554222 0.4092530
C 1.3617879 3.1564128 1.5555031
C 1.1597239 1.7839850 1.4480727
C 0.9711508 1.1942250 0.1892913
C 0.9851138 1.9961801 -0.9561333
C 1.1816457 3.3736085 -0.8432627
H 1.5213504 5.0339115 0.4952891
H 1.5101922 3.6071096 2.5397210
H 1.1471495 1.1659079 2.3501504
H 0.8362448 1.5485273 -1.9411421
H 1.1884838 3.9933616 -1.7430796
C 4.0948426 -2.6522568 2.2793004
C 4.2807096 -1.3952209 1.7028445
C 3.2208392 -0.7546873 1.0635023
C 1.9631774 -1.3715857 0.9946566
C 1.7809779 -2.6309897 1.5790622
C 2.8446887 -3.2677709 2.2162295
H 4.9265820 -3.1513146 2.7826908
H 5.2568692 -0.9067645 1.7519423
H 3.3753072 0.2332758 0.6236281
H 0.8017185 -3.1146610 1.5266726
H 2.6920263 -4.2503374 2.6690964
I -1.6892625 -3.7706229 0.1628722
Pd -1.4761054 -1.1625577 0.8701545

PdLHIcyclohexene **25**
E (E_h) = -1696.477682
OC (E_h) = -0.000672456
dU_{298} (kJ*mol^{-1}) = 1157.33
dS_{298} (kJ*mol^{-1}*K^{-1}) = 0.81314

53
I 1.9587198 1.2059605 2.6902295

C	-1.9626135	3.7639473	0.0682324
C	-1.8195142	2.9939181	1.3522264
C	-0.7276061	3.2640224	2.1600054
C	0.2379879	4.3277746	1.7191148
C	0.5643824	4.2690240	0.2241007
C	-0.6375421	3.9143835	-0.6797000
H	-2.3517718	4.7621753	0.3467109
H	-2.7211760	3.3082405	-0.5851844
H	-1.6335292	1.1399120	0.4247318
H	-2.7156294	2.5394007	1.7858489
H	-0.7610678	3.0014395	3.2219672
H	-0.2346189	5.2998783	1.9598758
H	1.1632680	4.2745644	2.3103967
H	1.0048769	5.2309219	-0.0796372
H	1.3540081	3.5127210	0.0862636
H	-0.4373768	2.9559808	-1.1874212
H	-0.7585396	4.6642781	-1.4762671
Pd	-0.3366813	1.2597270	1.2336339
P	0.0544429	-0.7302297	0.1786546
C	-0.8098364	-4.4562456	2.7740639
C	-0.3812185	-3.2665269	3.3638879
C	-0.1394996	-2.1423101	2.5766615
C	-0.3194300	-2.2056224	1.1887763
C	-0.7533271	-3.4006425	0.6000996
C	-0.9964204	-4.5212057	1.3928997
H	-1.0033255	-5.3354855	3.3935494
H	-0.2370210	-3.2098492	4.4454602
H	0.2006178	-1.2098613	3.0356953
H	-0.9046724	-3.4585244	-0.4802529
H	-1.3355637	-5.4497071	0.9273534
C	-2.3753135	-1.5900067	-3.6730596
C	-3.0023352	-1.0716129	-2.5378733
C	-2.2565536	-0.7906070	-1.3967689
C	-0.8777503	-1.0365619	-1.3711563
C	-0.2556560	-1.5634571	-2.5087267
C	-1.0032610	-1.8327982	-3.6563578
H	-2.9588090	-1.8012357	-4.5724882
H	-4.0776194	-0.8778877	-2.5439107
H	-2.7528025	-0.3736755	-0.5156381
H	0.8174855	-1.7651500	-2.5037543
H	-0.5057569	-2.2365945	-4.5413983
C	4.4428424	-1.1179886	-1.1957249
C	3.7133580	0.0471124	-1.4445052
C	2.3958239	0.1487012	-1.0101677
C	1.7924586	-0.9219621	-0.3371375
C	2.5232632	-2.0861492	-0.0888738
C	3.8485551	-2.1800637	-0.5164741
H	5.4807423	-1.1941844	-1.5290845
H	4.1780757	0.8847233	-1.9700571

H	1.8325460	1.0695478	-1.1863405
H	2.0623676	-2.9216088	0.4426865
H	4.4180170	-3.0906002	-0.3151662

PdLIH **26**
E (E_h) = -1461.996049
OC (E_h) = -0.001216749
dU_{298} (kJ*mol^{-1}) = 769.1
dS_{298} (kJ*mol^{-1}*K^{-1}) = 0.71257

37

Pd	-3.1643096	-0.2133818	-0.2435330
H	-3.0876217	1.2760756	-0.3575689
I	-5.7507972	-0.4382385	-0.4614772
P	-0.9349829	0.0047342	-0.0610052
C	0.7380338	-4.2921308	0.0717359
C	-0.2213494	-3.8682607	0.9939068
C	-0.7078584	-2.5651736	0.9427968
C	-0.2275675	-1.6736259	-0.0294609
C	0.7299087	-2.1023177	-0.9550296
C	1.2104810	-3.4106487	-0.9000866
H	1.1164763	-5.3164034	0.1102463
H	-0.5953482	-4.5573033	1.7546284
H	-1.4590426	-2.2370805	1.6681300
H	1.1033803	-1.4155361	-1.7178409
H	1.9600765	-3.7412031	-1.6228627
C	1.1562208	2.0602383	-3.6170265
C	-0.0715504	1.4198302	-3.7902089
C	-0.7058351	0.8238523	-2.7022072
C	-0.1064492	0.8585168	-1.4368930
C	1.1242025	1.5036578	-1.2647955
C	1.7510901	2.1038698	-2.3554589
H	1.6495175	2.5338674	-4.4693013
H	-0.5427081	1.3907115	-4.7753715
H	-1.6752016	0.3333610	-2.8315114
H	1.5931247	1.5431966	-0.2787126
H	2.7090338	2.6106604	-2.2174355
C	0.6173052	2.1383107	3.7219936
C	-0.5320713	2.5943644	3.0747254
C	-1.0082516	1.9296948	1.9471365
C	-0.3282390	0.8083369	1.4549129
C	0.8235942	0.3509040	2.1080529
C	1.2920663	1.0167250	3.2396837
H	0.9851086	2.6563893	4.6109678
H	-1.0672619	3.4675482	3.4548776
H	-1.9176313	2.2794859	1.4514141
H	1.3550643	-0.5280355	1.7353784
H	2.1893926	0.6550085	3.7472003

cis-PdL2HI **27**
E (E_h) = -2497.707019
OC (E_h) = -0.001092108
dU_{298} (kJ*mol^{-1}) = 1513.14
dS_{298} (kJ*mol^{-1}*K^{-1}) = 1.03994

71

P	0.7398861	-1.7435422	-0.4044978
P	-1.7972942	0.8870045	0.1497869
H	-3.1834653	-1.0988113	-0.4927406
C	1.7618766	-6.0921901	0.7920969
C	0.7083514	-5.4110403	1.4031579
C	0.3961718	-4.1125188	1.0072997
C	1.1503896	-3.4761513	0.0131523
C	2.2043351	-4.1630263	-0.5972274
C	2.5049060	-5.4689217	-0.2106687
H	1.9999740	-7.1152556	1.0937471
H	0.1161932	-5.8979782	2.1816112
H	-0.4483332	-3.5914853	1.4665112
H	2.7917025	-3.6817957	-1.3825093
H	3.3255958	-6.0017702	-0.6971740
C	2.5696983	-1.0382840	-4.5958497
C	1.2362949	-1.4227236	-4.4491303
C	0.7057890	-1.6315129	-3.1778728
C	1.5063871	-1.4612034	-2.0403948
C	2.8482858	-1.0910046	-2.1947335
C	3.3744793	-0.8769308	-3.4678153
H	2.9835262	-0.8657371	-5.5924940
H	0.6017917	-1.5557221	-5.3286501
H	-0.3408566	-1.9284761	-3.0623517
H	3.4865315	-0.9601947	-1.3182090
H	4.4209868	-0.5822771	-3.5776733
C	3.1849608	0.7431570	2.6513193
C	2.8109000	1.2980296	1.4276669
C	2.1242699	0.5316220	0.4894306
C	1.8012380	-0.8046194	0.7625621
C	2.1975362	-1.3616320	1.9856897
C	2.8823300	-0.5902269	2.9237410
H	3.7178636	1.3478201	3.3891104
H	3.0507430	2.3390366	1.1979799
H	1.8464637	0.9774748	-0.4661841
H	1.9687664	-2.4045053	2.2146809
H	3.1817135	-1.0387846	3.8741631
C	-5.8620873	2.9901103	-0.5566409
C	-4.7120595	3.5138454	-1.1421839
C	-3.4773418	2.8902668	-0.9473888
C	-3.3928406	1.7305376	-0.1718271
C	-4.5544509	1.2022439	0.4082551
C	-5.7802633	1.8329846	0.2228506

H -6.8269598 3.4797451 -0.7093025
H -4.7696445 4.4158446 -1.7562081
H -2.5804231 3.3127115 -1.4051377
H -4.4999497 0.2878668 1.0061043
H -6.6794227 1.4155252 0.6822620
C -0.9345943 1.8152471 4.5923459
C -0.4923008 0.6228641 4.0205396
C -0.7823069 0.3404322 2.6874296
C -1.5036239 1.2548990 1.9133680
C -1.9613506 2.4450263 2.4962427
C -1.6738415 2.7226229 3.8309967
H -0.7113477 2.0359457 5.6390926
H 0.0785842 -0.0953239 4.6132498
H -0.4481263 -0.5972448 2.2407712
H -2.5497315 3.1542294 1.9085311
H -2.0327693 3.6517229 4.2802315
C 1.3179588 3.3046954 -2.2571563
C 0.9958804 3.7173454 -0.9629819
C 0.0457407 3.0191856 -0.2191627
C -0.5911653 1.9028463 -0.7725220
C -0.2810182 1.5060388 -2.0794085
C 0.6740023 2.2007758 -2.8179226
H 2.0764934 3.8441148 -2.8295328
H 1.4987531 4.5815592 -0.5223785
H -0.1760710 3.3298864 0.8036938
H -0.7723689 0.6302450 -2.5105526
H 0.9261951 1.8659533 -3.8267763
Pd -1.6542490 -1.3006368 -0.4317824
I -2.3432880 -3.6799349 -1.3946296

cis-PdLICOchex **28**
E (E_h) = -1809.757430
OC (E_h) = -0.000639458
dU_{298} (kJ*mol^{-1}) = 1197.69
dS_{298} (kJ*mol^{-1}*K^{-1}) = 0.87102

55
P 0.3739609 0.7017453 -0.8026323
C -0.0910460 -3.2483140 2.2869844
C 0.4382552 -2.8502581 0.9050159
C -0.6478329 -2.1444205 0.1100892
C -1.8938688 -3.0056929 -0.0351358
C -2.4294453 -3.3773993 1.3510313
C -1.3619851 -4.0844455 2.1801831
H -0.3056734 -2.3289909 2.8606803
H 0.6869528 -3.7948821 2.8456724
H 0.7735651 -3.7566904 0.3690639
H 1.3264198 -2.2068986 1.0140226
H -0.9428968 -1.2362275 0.6543305

H	-2.6695515	-2.4795910	-0.6152493
H	-1.6656471	-3.9373330	-0.5831284
H	-2.7497248	-2.4572640	1.8726725
H	-3.3263398	-4.0111312	1.2511957
H	-1.1198732	-5.0522238	1.7039739
H	-1.7500668	-4.3209041	3.1847403
C	-2.0636930	3.7076705	-3.3210698
C	-0.7871330	4.0679432	-2.8911300
C	-0.0247004	3.1817408	-2.1304665
C	-0.5385814	1.9243916	-1.8018154
C	-1.8139827	1.5575347	-2.2533380
C	-2.5774575	2.4501791	-3.0004937
H	-2.6571928	4.4050311	-3.9172738
H	-0.3758869	5.0459832	-3.1520355
H	0.9775586	3.4716951	-1.8101908
H	-2.2054233	0.5599272	-2.0321679
H	-3.5713517	2.1566745	-3.3461700
C	-1.1538697	1.1485023	3.5416020
C	0.1215270	0.6425562	3.2852916
C	0.5889348	0.5549764	1.9766799
C	-0.2163597	0.9764003	0.9085294
C	-1.4925215	1.4869404	1.1708435
C	-1.9566541	1.5724045	2.4835327
H	-1.5200675	1.2159181	4.5688261
H	0.7582299	0.3131565	4.1097863
H	1.5894529	0.1586367	1.7872700
H	-2.1302694	1.8244601	0.3510669
H	-2.9528634	1.9769016	2.6776301
C	4.7872415	2.0362051	-0.5204984
C	3.7862910	2.8021796	0.0805905
C	2.4556809	2.4013802	-0.0038969
C	2.1142048	1.2344632	-0.7054174
C	3.1224960	0.4658778	-1.2970087
C	4.4549329	0.8672389	-1.2031060
H	5.8315527	2.3500180	-0.4490596
H	4.0435249	3.7143188	0.6241124
H	1.6816068	2.9936779	0.4902675
H	2.8621501	-0.4458465	-1.8388140
H	5.2355255	0.2597096	-1.6667741
C	-0.0042858	-3.2254207	-2.4456930
Pd	0.0838935	-1.4499000	-1.7107220
I	0.8933007	-0.5609624	-4.1836796
O	0.0089876	-4.2516424	-2.9287178

trans-PdLICOchex **29**
E (E_h) = -1809.751871
OC (E_h) = -0.000833994
dU_{298} (kJ*mol^{-1}) = 1197.86
dS_{298} (kJ*mol^{-1}*K^{-1}) = 0.87094

55

P	0.8835553	-0.0202854	0.0703729
C	-3.2553852	-0.5228358	-2.3464236
C	-1.7436105	-0.4829671	-2.1009110
C	-1.2563955	-1.8042134	-1.5288703
C	-2.0098924	-2.2332136	-0.2809948
C	-3.5192046	-2.2622344	-0.5466389
C	-4.0106129	-0.9225805	-1.0832596
H	-3.4724543	-1.2538344	-3.1461683
H	-3.6024958	0.4574545	-2.7143842
H	-1.5279397	0.3475422	-1.4107598
H	-1.2147998	-0.2509790	-3.0400612
H	-1.4157107	-2.5762728	-2.2973486
H	-1.6658055	-3.2284209	0.0454382
H	-1.8184183	-1.5408899	0.5533068
H	-3.7437301	-3.0537837	-1.2843054
H	-4.0579281	-2.5307457	0.3776541
H	-3.8595566	-0.1478798	-0.3094475
H	-5.0947919	-0.9612583	-1.2811251
C	4.4651116	-0.7777743	2.9107507
C	3.5490344	-1.7897296	2.6219124
C	2.4872470	-1.5405752	1.7558714
C	2.3193215	-0.2697285	1.1845927
C	3.2447576	0.7386196	1.4732346
C	4.3140681	0.4812789	2.3310655
H	5.3049124	-0.9746506	3.5814042
H	3.6667858	-2.7825904	3.0619898
H	1.7910655	-2.3484920	1.5097569
H	3.1477084	1.7266755	1.0203870
H	5.0361783	1.2732624	2.5432488
C	1.8403708	3.7697662	-2.3865895
C	1.8195975	2.5157915	-2.9953673
C	1.5132604	1.3818899	-2.2445361
C	1.2305091	1.4945052	-0.8777088
C	1.2460514	2.7606853	-0.2722577
C	1.5517500	3.8910461	-1.0258924
H	2.0769251	4.6593113	-2.9754154
H	2.0363441	2.4169259	-4.0613630
H	1.4830525	0.3991263	-2.7244224
H	1.0123801	2.8683326	0.7895685
H	1.5626730	4.8731644	-0.5476050
C	-2.5961611	0.9879495	2.9571639
C	-2.4784402	1.6741100	1.7494723
C	-1.4156297	1.4041594	0.8892549
C	-0.4548368	0.4427498	1.2305600
C	-0.5739487	-0.2354188	2.4519230
C	-1.6384696	0.0359619	3.3075997
H	-3.4317521	1.1986778	3.6287770

H -3.2184176 2.4276006 1.4699147
H -1.3389095 1.9551625 -0.0496087
H 0.1651732 -0.9838293 2.7436541
H -1.7180650 -0.5014940 4.2553367
Pd 0.8081320 -1.8841328 -1.3219119
I 0.8480209 -4.1196911 -2.8356539
C 2.8029219 -2.0421249 -1.3557666
O 3.9264541 -2.1391234 -1.4694141

PdLIchexCO **30**
E (E_h) = -1809.775718
OC (E_h) = -0.000830842
dU_{298} (kJ*mol^{-1}) = 1198.22
dS_{298} (kJ*mol^{-1}*K^{-1}) = 0.83205

55
I 1.9896751 -4.5196203 -0.6818910
P 1.1443863 0.1150749 0.7178659
C -3.0306420 1.9960747 0.0918939
C -2.0025754 2.2885533 -0.8034633
C -0.7269951 1.7608734 -0.6044546
C -0.4756980 0.9274697 0.4915534
C -1.5114170 0.6371666 1.3914725
C -2.7805597 1.1745387 1.1923738
H -4.0288608 2.4114342 -0.0653187
H -2.1899755 2.9361496 -1.6631577
H 0.0728403 2.0004198 -1.3088376
H -1.3295839 -0.0143600 2.2492990
H -3.5807722 0.9440032 1.8995265
C 2.2925843 0.7956806 5.1340592
C 2.6921986 -0.3621888 4.4668294
C 2.3201512 -0.5643588 3.1388814
C 1.5512808 0.3957518 2.4699811
C 1.1459934 1.5549642 3.1463230
C 1.5184508 1.7518290 4.4742245
H 2.5797246 0.9517001 6.1767745
H 3.2898672 -1.1167361 4.9832784
H 2.6196524 -1.4788333 2.6174860
H 0.5324079 2.3029621 2.6382360
H 1.1998339 2.6564051 4.9975786
C 4.0443001 2.6549289 -1.8293208
C 3.8424207 2.9913527 -0.4899832
C 2.9803772 2.2349599 0.3018881
C 2.3158521 1.1310211 -0.2458700
C 2.5327242 0.7878757 -1.5881183
C 3.3883069 1.5530472 -2.3784335
H 4.7220041 3.2504072 -2.4458074
H 4.3614727 3.8490889 -0.0557653
H 2.8323447 2.5037700 1.3499240

H 2.0295603 -0.0847203 -2.0156828
H 3.5488840 1.2805344 -3.4240231
Pd 1.3607633 -2.0886004 0.0689516
C -0.5344825 -2.4280855 0.3316716
O -0.9447433 -2.6213103 1.4315443
C -1.6936409 -1.8876945 -3.3606266
C -0.8211346 -1.6966020 -2.1246198
C -1.3492019 -2.5150566 -0.9489740
C -2.8229875 -2.2156068 -0.6590125
C -3.6759625 -2.4036535 -1.9075963
C -3.1556067 -1.5681205 -3.0713639
H -1.6095762 -2.9344274 -3.7041863
H -1.3127238 -1.2581840 -4.1804715
H -0.8134875 -0.6304150 -1.8434026
H 0.2197329 -1.9838534 -2.3446134
H -1.2426350 -3.5888214 -1.2067925
H -3.1736499 -2.8577595 0.1622873
H -2.9055524 -1.1730340 -0.3080137
H -3.6726655 -3.4706335 -2.1943213
H -4.7226246 -2.1440411 -1.6806075
H -3.2496526 -0.4960880 -2.8187238
H -3.7703835 -1.7352315 -3.9704507

cis-PdL2chexCOI **cis-31**
E (E_h) = -2845.471178
OC (E_h) = -0.000772275
dU_{298} (kJ*mol^{-1}) = 1941.23
dS_{298} (kJ*mol^{-1}*K^{-1}) = 1.14807

89
P 1.9568904 -1.2897921 -0.0316253
P -0.7904452 0.8926577 0.8249512
C 3.9468296 -4.9482639 2.0375976
C 3.7833726 -3.7572883 2.7443893
C 3.2186613 -2.6436598 2.1243711
C 2.8121178 -2.7010233 0.7849571
C 2.9718850 -3.9059285 0.0850625
C 3.5393840 -5.0168484 0.7062027
H 4.3910580 -5.8203553 2.5236411
H 4.0998446 -3.6884215 3.7880555
H 3.1117693 -1.7245112 2.7003961
H 2.6521214 -3.9804794 -0.9548773
H 3.6592055 -5.9446133 0.1413608
C 3.0813994 -1.7948372 -4.4929098
C 1.7567552 -1.5781321 -4.1180288
C 1.4298173 -1.4388247 -2.7694751
C 2.4215212 -1.5044822 -1.7866087
C 3.7505331 -1.7374346 -2.1712409
C 4.0774327 -1.8793106 -3.5170934

H	3.3401349	-1.9080252	-5.5486447
H	0.9712841	-1.5267866	-4.8757397
H	0.3893978	-1.3009098	-2.4687215
H	4.5340707	-1.8141747	-1.4131844
H	5.1153536	-2.0602163	-3.8067554
C	4.3867695	2.4004677	1.4306173
C	4.4584773	2.0160809	0.0931150
C	3.7371960	0.9154979	-0.3678250
C	2.9344449	0.1742733	0.5079658
C	2.8390943	0.5947342	1.8429756
C	3.5650132	1.6897616	2.3040262
H	4.9555113	3.2626039	1.7873406
H	5.0770119	2.5819891	-0.6077010
H	3.7970291	0.6446794	-1.4222886
H	2.1735650	0.0819258	2.5364510
H	3.4745885	1.9928037	3.3499719
C	-4.9794940	2.8557660	0.4782971
C	-4.0453720	3.0399233	-0.5403170
C	-2.7745009	2.4751458	-0.4380199
C	-2.4300309	1.7097353	0.6826034
C	-3.3734348	1.5278224	1.7037969
C	-4.6378347	2.1022986	1.6020940
H	-5.9737725	3.3016416	0.3982839
H	-4.3014894	3.6339041	-1.4208814
H	-2.0483176	2.6427939	-1.2361389
H	-3.1226023	0.9290010	2.5814509
H	-5.3635181	1.9533308	2.4051479
C	0.0788067	1.3463043	5.3399371
C	0.1337090	0.0948161	4.7274796
C	-0.1254038	-0.0232790	3.3625899
C	-0.4362170	1.1095393	2.6007169
C	-0.5325442	2.3579531	3.2313074
C	-0.2616286	2.4755339	4.5923265
H	0.2854711	1.4416029	6.4087175
H	0.3752101	-0.7953241	5.3129008
H	-0.0905761	-1.0036031	2.8760738
H	-0.8384570	3.2407790	2.6652844
H	-0.3275505	3.4534374	5.0750484
C	1.8217065	3.5915340	-1.8698060
C	1.7601218	3.8725466	-0.5052166
C	0.9967504	3.0767420	0.3466766
C	0.2890821	1.9852453	-0.1650731
C	0.3683030	1.6969965	-1.5343145
C	1.1264022	2.4982548	-2.3843385
H	2.4256107	4.2177568	-2.5309041
H	2.3216426	4.7141538	-0.0934517
H	0.9791815	3.2994038	1.4136509
H	-0.1668495	0.8337460	-1.9362673
H	1.1806330	2.2581032	-3.4486944

Pd -0.5036241 -1.3143135 0.2289786
C -2.4905293 -1.5212839 0.4617625
I -0.5922137 -3.9154521 -0.4560476
O -2.9353380 -1.6870241 1.5675919
C -3.6873421 -0.9894504 -3.2194907
C -2.7698313 -0.8660761 -2.0079977
C -3.3736958 -1.5398408 -0.7815542
C -4.7833919 -1.0273893 -0.4856607
C -5.6873177 -1.1583160 -1.7052730
C -5.0854647 -0.4595605 -2.9197113
H -3.7566293 -2.0530325 -3.5114465
H -3.2473737 -0.4573589 -4.0786370
H -2.5999143 0.2007529 -1.7867310
H -1.7868966 -1.3142796 -2.2245303
H -3.4316450 -2.6258297 -0.9967955
H -5.1953389 -1.5711597 0.3774150
H -4.7205395 0.0326957 -0.1901036
H -5.8360717 -2.2283656 -1.9385197
H -6.6843452 -0.7461743 -1.4782003
H -5.0256364 0.6256752 -2.7176972
H -5.7389925 -0.5809788 -3.7990414

trans-PdL2chexCOI **trans-31**
E (E_h) = -2845.477606
OC (E_h) = -0.000739498
dU_{298} (kJ*mol^{-1}) = 1943.22
dS_{298} (kJ*mol^{-1}*K^{-1}) = 1.21443

89
P 1.3470680 -1.9926515 -0.2439621
P 0.2053865 2.5230328 0.1017463
C -1.6159588 -5.1006027 1.5219173
C -0.7388760 -4.4235585 2.3687589
C 0.1779725 -3.5103887 1.8503127
C 0.2284349 -3.2637336 0.4732907
C -0.6544564 -3.9505739 -0.3731786
C -1.5692683 -4.8627785 0.1482588
H -2.3334929 -5.8155286 1.9316551
H -0.7643617 -4.6067748 3.4456151
H 0.8570946 -2.9932781 2.5294845
H -0.6248117 -3.7816032 -1.4519358
H -2.2486316 -5.3905354 -0.5254627
C 2.2294398 -3.9147764 -4.3458607
C 1.7315865 -2.6147644 -4.2727560
C 1.4934430 -2.0262653 -3.0307972
C 1.7477551 -2.7397576 -1.8553551
C 2.2261459 -4.0562494 -1.9342319
C 2.4751659 -4.6360631 -3.1753646
H 2.4205389 -4.3734205 -5.3192413

H	1.5289942	-2.0510685	-5.1864012
H	1.1143960	-1.0039463	-2.9680137
H	2.3899051	-4.6384557	-1.0238702
H	2.8548519	-5.6591138	-3.2296631
C	5.0799636	-1.8417558	2.4775083
C	5.1719131	-2.4912470	1.2473868
C	4.0577413	-2.5824852	0.4126965
C	2.8402433	-2.0237026	0.8096280
C	2.7552535	-1.3517929	2.0397277
C	3.8686142	-1.2710694	2.8720749
H	5.9565409	-1.7701393	3.1260356
H	6.1218766	-2.9252999	0.9261502
H	4.1521557	-3.0710739	-0.5583292
H	1.8172084	-0.8715598	2.3359526
H	3.7917578	-0.7474881	3.8282171
C	-4.2240296	3.1201121	1.2882517
C	-3.8268945	3.3197474	-0.0337007
C	-2.4815393	3.2149123	-0.3859680
C	-1.5209353	2.9023981	0.5836472
C	-1.9236623	2.7171501	1.9143861
C	-3.2675315	2.8272994	2.2622266
H	-5.2789834	3.1977257	1.5617954
H	-4.5681456	3.5590125	-0.7999625
H	-2.1838451	3.3691207	-1.4253957
H	-1.1871227	2.4653976	2.6804233
H	-3.5701998	2.6755522	3.3010256
C	2.7368880	4.2822014	3.5441947
C	3.0861824	3.0658459	2.9586941
C	2.3188434	2.5444307	1.9181997
C	1.1945507	3.2393441	1.4560017
C	0.8405791	4.4568593	2.0561298
C	1.6130019	4.9764798	3.0923987
H	3.3381888	4.6894659	4.3607977
H	3.9606426	2.5148668	3.3133781
H	2.5932055	1.5935864	1.4542482
H	-0.0502992	4.9961391	1.7246397
H	1.3319703	5.9262447	3.5536281
C	0.7358901	5.0582125	-3.7237294
C	1.0626571	5.6182975	-2.4890007
C	0.9229706	4.8710251	-1.3196158
C	0.4630219	3.5527056	-1.3832570
C	0.1557942	2.9873323	-2.6276046
C	0.2799146	3.7410011	-3.7916333
H	0.8459521	5.6469515	-4.6377761
H	1.4320196	6.6450977	-2.4324980
H	1.1890445	5.3185656	-0.3600881
H	-0.1632843	1.9430363	-2.6847112
H	0.0357232	3.2919794	-4.7572207
Pd	0.7311672	0.2682232	-0.2157376

C	-0.8075636	-0.2215532	0.9486855
I	3.0765784	0.9367410	-1.5321925
O	-0.6244687	-0.3390586	2.1357076
C	-4.4374651	-1.5002388	0.5278532
C	-3.0276259	-1.4095443	1.0985929
C	-2.1846038	-0.3947171	0.3257865
C	-2.1751930	-0.6655550	-1.1737482
C	-3.5938531	-0.7586718	-1.7236653
C	-4.4174321	-1.7964800	-0.9681171
H	-4.9617516	-0.5422556	0.6999002
H	-5.0113605	-2.2725626	1.0660448
H	-2.5399242	-2.3967485	1.0434259
H	-3.0471940	-1.1266823	2.1613318
H	-2.6441120	0.6009816	0.4749903
H	-1.6096410	0.1275405	-1.6912227
H	-1.6398679	-1.6091571	-1.3727268
H	-4.0804612	0.2294977	-1.6296234
H	-3.5664620	-0.9940353	-2.7998999
H	-3.9767716	-2.7969129	-1.1321088
H	-5.4441518	-1.8364378	-1.3671748

Cis-PdL2COchex **32**
E (E_h) = -2547.523373
OC (E_h) = 0.003132245
dU_{298} ($kJ*mol^{-1}$) = 1933.78
dS_{298} ($kJ*mol^{-1}*K^{-1}$) = 1.15811

88

P	1.8386574	-0.9444883	-0.3026948
P	-0.8884080	1.0696518	0.5616027
C	3.5252687	-3.9686747	2.7565911
C	4.3568730	-2.9745798	2.2388235
C	3.8581186	-2.0490920	1.3250292
C	2.5173572	-2.1139327	0.9189472
C	1.6889723	-3.1138993	1.4443060
C	2.1906649	-4.0384001	2.3582515
H	3.9189118	-4.6897267	3.4768839
H	5.4025911	-2.9171413	2.5495337
H	4.5152777	-1.2705669	0.9309962
H	0.6369224	-3.1678691	1.1518024
H	1.5334273	-4.8106531	2.7639595
C	3.2223275	-2.3735660	-4.4776264
C	2.1580706	-1.4812242	-4.3424540
C	1.7543887	-1.0692814	-3.0746459
C	2.4225077	-1.5342291	-1.9318632
C	3.4884452	-2.4288613	-2.0733515
C	3.8821636	-2.8475760	-3.3439826
H	3.5345301	-2.7052705	-5.4706557
H	1.6345352	-1.1100111	-5.2264165

H	0.9123199	-0.3785287	-2.9726875
H	4.0098327	-2.8108776	-1.1933611
H	4.7110334	-3.5518031	-3.4464853
C	4.2114029	2.9643169	0.4114016
C	4.0068961	2.4998960	-0.8868191
C	3.2954320	1.3227518	-1.1089819
C	2.7817091	0.5972783	-0.0273999
C	2.9838616	1.0713660	1.2770829
C	3.6966737	2.2479919	1.4918125
H	4.7729230	3.8858855	0.5820317
H	4.4048000	3.0558455	-1.7386562
H	3.1520217	0.9685012	-2.1312126
H	2.6013823	0.5148662	2.1339094
H	3.8539452	2.6024476	2.5131732
C	-5.2444601	2.4912665	1.1135044
C	-4.4891134	2.9971953	0.0562848
C	-3.1592337	2.6099411	-0.1050719
C	-2.5786966	1.7136340	0.7996304
C	-3.3383603	1.2116166	1.8660511
C	-4.6662853	1.6005568	2.0196476
H	-6.2866946	2.7952488	1.2355944
H	-4.9352095	3.7011693	-0.6499525
H	-2.5765799	3.0105925	-0.9373835
H	-2.8920068	0.5169022	2.5821871
H	-5.2523618	1.2065889	2.8530940
C	0.7946665	0.9540768	4.8536502
C	0.6981026	-0.2139179	4.0989508
C	0.1888679	-0.1600705	2.8027815
C	-0.2193356	1.0591317	2.2505285
C	-0.1706533	2.2214289	3.0334723
C	0.3500871	2.1676994	4.3235616
H	1.2015134	0.9185227	5.8669629
H	1.0210316	-1.1709511	4.5148465
H	0.1112908	-1.0752435	2.2127194
H	-0.5523371	3.1689141	2.6474380
H	0.3999581	3.0786736	4.9242455
C	1.0641899	4.1479261	-2.2652637
C	1.2288936	4.3105174	-0.8905315
C	0.6703630	3.3968173	0.0011243
C	-0.0569175	2.3068051	-0.4843824
C	-0.2104819	2.1393296	-1.8692638
C	0.3444920	3.0586234	-2.7558657
H	1.5063860	4.8678513	-2.9578689
H	1.8062280	5.1528951	-0.5036071
H	0.8296650	3.5303427	1.0708177
H	-0.7773547	1.2864610	-2.2568417
H	0.2161046	2.9205632	-3.8317834
Pd	-0.6022007	-1.0109761	-0.4832828
C	-0.5117823	-2.7686243	-1.2927942

C -4.8766912 -1.9481022 0.0245585
C -3.3548851 -2.0144802 0.1912002
C -2.6861490 -1.0778065 -0.8007936
C -3.0626633 -1.3959200 -2.2400997
C -4.5837385 -1.3063006 -2.3988376
C -5.2936983 -2.2309082 -1.4149753
H -5.2196464 -0.9390417 0.3153243
H -5.3612340 -2.6574731 0.7155128
H -3.0166902 -3.0542719 0.0296919
H -3.0791297 -1.7528763 1.2259265
H -3.0378940 -0.0588154 -0.5949527
H -2.5664486 -0.7038611 -2.9408276
H -2.7406960 -2.4142447 -2.5216318
H -4.9035101 -0.2638776 -2.2202995
H -4.8678534 -1.5500762 -3.4356983
H -5.0469237 -3.2790705 -1.6643228
H -6.3866002 -2.1358581 -1.5215293
O -0.4131600 -3.8090695 -1.7282901

cisL-PdL2chexCO **33**
E (E_h) = -2547.520757
OC (E_h) = 0.003021088
dU_{298} ($kJ*mol^{-1}$) = 1933.01
dS_{298} ($kJ*mol^{-1}*K^{-1}$) = 1.14182

88
P 2.0142990 -1.2439598 -0.1316015
P -0.9251372 0.9297106 0.8554699
C 3.4890525 -4.6592822 2.6019548
C 4.3880394 -3.6646830 2.2127300
C 3.9653065 -2.6185832 1.3953150
C 2.6345052 -2.5645098 0.9593198
C 1.7364969 -3.5628266 1.3587114
C 2.1620780 -4.6089089 2.1751328
H 3.8241247 -5.4754574 3.2464681
H 5.4265256 -3.7024525 2.5499112
H 4.6713438 -1.8376452 1.1026138
H 0.6904521 -3.5241052 1.0332345
H 1.4537962 -5.3812600 2.4831453
C 3.2437189 -2.2899937 -4.4594983
C 2.1822878 -1.4207163 -4.2060902
C 1.8221014 -1.1306958 -2.8917097
C 2.5300943 -1.6962772 -1.8217367
C 3.5929349 -2.5690946 -2.0818058
C 3.9444102 -2.8644249 -3.3979775
H 3.5232559 -2.5252302 -5.4891773
H 1.6283006 -0.9723512 -5.0340172
H 0.9888379 -0.4516096 -2.6921593
H 4.1452732 -3.0260870 -1.2577517

H	4.7721955	-3.5495863	-3.5951848
C	4.5548825	2.4380597	1.0301355
C	4.4550270	2.0665940	-0.3090945
C	3.6964667	0.9557096	-0.6774471
C	3.0322566	0.2083387	0.2996757
C	3.1242472	0.5922387	1.6454238
C	3.8852794	1.6996640	2.0071135
H	5.1535804	3.3066697	1.3144876
H	4.9726185	2.6437189	-1.0787864
H	3.6312248	0.6696008	-1.7292687
H	2.6126757	0.0175474	2.4202871
H	3.9566110	1.9858066	3.0591623
C	-5.2784211	2.4573292	0.6905731
C	-4.3659705	2.8866327	-0.2726780
C	-3.0388108	2.4621555	-0.2212503
C	-2.6215919	1.5941954	0.7950669
C	-3.5407744	1.1668414	1.7651910
C	-4.8617325	1.6022229	1.7125639
H	-6.3169318	2.7936424	0.6485941
H	-4.6842759	3.5642055	-1.0681427
H	-2.3306146	2.8190658	-0.9717460
H	-3.2260765	0.4911580	2.5640248
H	-5.5710592	1.2674993	2.4727474
C	0.1883874	1.0790912	5.3244963
C	0.4176326	-0.0827318	4.5886959
C	0.0756469	-0.1291972	3.2383379
C	-0.4943089	0.9885220	2.6182781
C	-0.7481840	2.1465151	3.3687342
C	-0.3980026	2.1904965	4.7154260
H	0.4565751	1.1163894	6.3829473
H	0.8595927	-0.9595677	5.0671489
H	0.2455478	-1.0428678	2.6611089
H	-1.2272496	3.0125140	2.9050013
H	-0.5908626	3.0960062	5.2950642
C	1.6459027	3.9134635	-1.5463575
C	1.6627600	3.9692010	-0.1539377
C	0.9021496	3.0753360	0.5976858
C	0.1150844	2.1175499	-0.0495393
C	0.1195445	2.0486268	-1.4506099
C	0.8754808	2.9486825	-2.1956831
H	2.2466076	4.6157742	-2.1288672
H	2.2805453	4.7098864	0.3579311
H	0.9373939	3.1216390	1.6868583
H	-0.4745590	1.2876746	-1.9628350
H	0.8676576	2.8905457	-3.2863440
Pd	-0.4152061	-1.1280029	0.0264604
C	-2.3369787	-1.5784336	0.1854208
O	-2.6878965	-2.0788737	1.2108250
C	-3.4241696	-0.7860897	-3.4791812

C -2.5672692 -0.7059234 -2.2201472
C -3.1460024 -1.5687696 -1.1034430
C -4.6125364 -1.2249812 -0.8128515
C -5.4502164 -1.3054536 -2.0826747
C -4.8762218 -0.4252025 -3.1872605
H -3.3755235 -1.8123279 -3.8847827
H -3.0068136 -0.1230928 -4.2532149
H -2.5289948 0.3411079 -1.8765042
H -1.5318258 -1.0157383 -2.4455140
H -3.0979594 -2.6295626 -1.4212730
H -4.9990215 -1.9009486 -0.0359827
H -4.6602381 -0.2026709 -0.4035843
H -5.4874117 -2.3530806 -2.4309695
H -6.4879723 -1.0137457 -1.8549571
H -4.9315714 0.6329429 -2.8731667
H -5.4824171 -0.5135691 -4.1026686

Pd(dppp)chexI **34**
E (E_h) = -2387.073459
OC (E_h) = -0.001102853
dU_{298} (kJ*mol^{-1}) = 1643.21
dS_{298} (kJ*mol^{-1}*K^{-1}) = 0.98822

74
P 1.7781871 1.4165575 -0.9135469
P -1.1234352 0.7789791 0.6436752
C 6.0415986 2.8222095 0.0956778
C 5.0004037 2.9899085 1.0105162
C 3.7174333 2.5550033 0.6919292
C 3.4723196 1.9547316 -0.5501155
C 4.5162804 1.7775186 -1.4629838
C 5.7980834 2.2170712 -1.1369074
H 7.0491951 3.1607609 0.3481055
H 5.1892988 3.4565960 1.9798504
H 2.9124363 2.6713044 1.4234430
H 4.3365525 1.2894705 -2.4228942
H 6.6128279 2.0790554 -1.8513142
C 1.5094077 -0.0142036 -5.2924998
C 1.2773948 -0.9148894 -4.2542661
C 1.3744243 -0.4921496 -2.9300265
C 1.6973336 0.8382060 -2.6353969
C 1.9285186 1.7411548 -3.6850300
C 1.8383382 1.3117172 -5.0062635
H 1.4355331 -0.3458756 -6.3308121
H 1.0229865 -1.9542603 -4.4729064
H 1.2035127 -1.2020632 -2.1182165
H 2.1909781 2.7821146 -3.4829970
H 2.0261385 2.0190478 -5.8170261
C 0.8850621 3.0165727 -0.9335529

C	-4.0116986	-1.8095774	-1.8628635
C	-2.9129805	-1.1741947	-2.4435503
C	-2.0728790	-0.3850526	-1.6638025
C	-2.3301655	-0.2109459	-0.2944398
C	-3.4372304	-0.8450673	0.2807913
C	-4.2706252	-1.6431130	-0.5032968
H	-4.6647562	-2.4390723	-2.4715179
H	-2.6994223	-1.3043833	-3.5068279
H	-1.1945430	0.0714238	-2.1262222
H	-3.6565836	-0.7319357	1.3436200
H	-5.1281718	-2.1384704	-0.0425288
C	-2.3263472	1.4195468	5.0423782
C	-1.0213286	1.0557296	4.7121455
C	-0.6798633	0.8231151	3.3813905
C	-1.6440265	0.9520917	2.3740821
C	-2.9479831	1.3470377	2.7071933
C	-3.2863020	1.5688056	4.0401788
H	-2.5962237	1.5966833	6.0861093
H	-0.2640441	0.9508344	5.4921251
H	0.3454031	0.5391656	3.1238153
H	-3.7046770	1.4917025	1.9324278
H	-4.3053492	1.8675359	4.2960325
Pd	0.9438150	-0.2165999	0.4574023
I	3.3128485	-1.4036920	0.2737993
C	-0.5855795	-4.4800711	0.4068812
C	-0.2664221	-3.0020356	0.0653243
C	0.0593221	-2.2887873	1.3312155
C	-0.9754773	-2.3188531	2.3960619
C	-1.2764211	-3.8030960	2.7222883
C	-1.6695956	-4.5702774	1.4692566
H	0.3334532	-4.9726351	0.7660314
H	-0.8844009	-4.9919417	-0.5212259
H	-1.1579816	-2.5646359	-0.4114694
H	0.5682012	-2.9601255	-0.6493143
H	1.0940228	-2.3897484	1.6893440
H	-0.6471201	-1.7972384	3.3038879
H	-1.9140905	-1.8580164	2.0546992
H	-0.3814966	-4.2617139	3.1757088
H	-2.0732135	-3.8346062	3.4817922
H	-2.6150689	-4.1636024	1.0680142
H	-1.8616550	-5.6261156	1.7205340
C	-1.3832522	2.5015645	0.0397109
C	-0.6091120	2.9346653	-1.1927989
H	-1.1176733	3.1505694	0.8894882
H	-2.4690433	2.6001621	-0.1143159
H	-0.9646945	3.9372469	-1.4752217
H	-0.8253587	2.2907941	-2.0609927
H	1.3920214	3.6156095	-1.7056392
H	1.0929609	3.5167828	0.0258565

Pd(dppp)COchex **35**
E (E_h) = -2202.429152
OC (E_h) = 0.003259335
dU_{298} (kJ*mol^{-1}) = 1667.2
dS_{298} (kJ*mol^{-1}*K^{-1}) = 1.00523

75

P	1.1943165	1.2348165	-1.4034669
P	-1.5396879	0.3117545	0.0711437
C	3.8538505	3.5268364	1.5857829
C	2.8838849	4.2233089	0.8673863
C	2.0814703	3.5572262	-0.0583607
C	2.2429093	2.1825419	-0.2639274
C	3.2231489	1.4875473	0.4591472
C	4.0272703	2.1574083	1.3757885
H	4.4780070	4.0515478	2.3129482
H	2.7465890	5.2952214	1.0267143
H	1.3289458	4.1221554	-0.6107454
H	3.3572819	0.4124613	0.3135116
H	4.7870321	1.6065885	1.9348516
C	3.6309805	0.5900891	-5.2714611
C	2.5851809	-0.2726208	-4.9433691
C	1.8715639	-0.0831669	-3.7610578
C	2.1938066	0.9795965	-2.9065750
C	3.2428517	1.8451579	-3.2411165
C	3.9598668	1.6454891	-4.4192193
H	4.1966635	0.4365009	-6.1934874
H	2.3277454	-1.1025127	-5.6054021
H	1.0624606	-0.7729356	-3.5013722
H	3.5061088	2.6722702	-2.5769917
H	4.7824785	2.3184262	-4.6723548
C	-0.1401837	2.3757966	-1.9690953
C	-4.9257163	-2.0062106	2.1821659
C	-4.7639750	-2.1065912	0.7996714
C	-3.7606929	-1.3833721	0.1596780
C	-2.9167913	-0.5414607	0.8994566
C	-3.0810814	-0.4468823	2.2867671
C	-4.0838308	-1.1774703	2.9226917
H	-5.7109505	-2.5769121	2.6834001
H	-5.4189233	-2.7561661	0.2146794
H	-3.6380960	-1.4874509	-0.9217461
H	-2.4288546	0.2021195	2.8752253
H	-4.2074321	-1.0946006	4.0048559
C	-0.1350766	3.5328754	3.0669296
C	0.5453193	2.3219223	2.9550176
C	0.1109302	1.3639464	2.0421803
C	-1.0120241	1.6068615	1.2393586
C	-1.7036263	2.8181121	1.3711667

C	-1.2596247	3.7786179	2.2771905
H	0.2118570	4.2915930	3.7722685
H	1.4281499	2.1256373	3.5665818
H	0.6579543	0.4220161	1.9438853
H	-2.5920119	3.0240125	0.7699839
H	-1.7976520	4.7249574	2.3679538
Pd	0.3872229	-0.8662267	-0.5289659
C	2.0195029	-1.8310684	-0.8858360
C	-0.9891757	-3.8854964	2.3535314
C	-0.2167594	-2.7090343	1.7478836
C	-0.4758153	-2.6244908	0.2515454
C	-0.0963834	-3.9175066	-0.4569136
C	-0.8925061	-5.0838318	0.1355179
C	-0.6682229	-5.1944486	1.6402049
H	-2.0703512	-3.6733041	2.2694062
H	-0.7677321	-3.9662042	3.4306498
H	0.8640470	-2.8423800	1.9371756
H	-0.5124342	-1.7775606	2.2577463
H	-1.5531996	-2.4747763	0.0926482
H	-0.2811363	-3.8426132	-1.5410723
H	0.9798668	-4.1319145	-0.3300385
H	-1.9675967	-4.9256320	-0.0648947
H	-0.6142406	-6.0243076	-0.3682614
H	0.3887385	-5.4584266	1.8271777
H	-1.2719047	-6.0162197	2.0588954
C	-2.3113258	1.1416087	-1.3780549
C	-1.3519661	1.5950307	-2.4756972
H	-2.9049773	1.9864639	-0.9953493
H	-3.0325588	0.4204930	-1.7892620
H	-1.9198176	2.2279822	-3.1740489
H	-1.0230090	0.7220187	-3.0646514
H	0.2861495	3.0115702	-2.7588270
H	-0.4238654	3.0326522	-1.1331727
O	3.0170579	-2.3294368	-1.0859703

Pd(dppp)chexCO **36**
E (E_h) = -2202.42915
OC (E_h) = 0.003277913
dU_{298} (kJ*mol^{-1}) = 1667.2
dS_{298} (kJ*mol^{-1}*K^{-1}) = 1.00523

75

P	1.5398345	1.6399270	-0.8747326
P	-1.2628750	0.3340680	0.3639296
C	3.9485736	4.6251479	1.6837557
C	2.9613922	5.1133637	0.8287527
C	2.2197419	4.2349710	0.0389529
C	2.4661849	2.8593972	0.1018127
C	3.4617598	2.3737582	0.9616855

C	4.1994517	3.2530544	1.7492746
H	4.5241113	5.3165646	2.3039550
H	2.7612134	6.1861861	0.7758898
H	1.4479132	4.6372669	-0.6201461
H	3.6489835	1.2971321	1.0233135
H	4.9707083	2.8666470	2.4195700
C	4.1618214	0.6714910	-4.5567734
C	3.3346407	-0.3038434	-4.0010395
C	2.5538015	0.0013670	-2.8868585
C	2.5863608	1.2862197	-2.3282172
C	3.4144089	2.2639683	-2.8962491
C	4.1999265	1.9536043	-4.0038600
H	4.7822687	0.4317818	-5.4234902
H	3.3009530	-1.3085322	-4.4286096
H	1.9166130	-0.7734636	-2.4444303
H	3.4608436	3.2652357	-2.4598630
H	4.8502859	2.7165697	-4.4379287
C	0.1021246	2.5604288	-1.5740967
C	-4.3450162	-2.4559456	2.3461158
C	-4.4615820	-2.1906154	0.9822564
C	-3.5767862	-1.3112142	0.3580504
C	-2.5701646	-0.6860363	1.1037886
C	-2.4691211	-0.9415426	2.4802170
C	-3.3527277	-1.8220635	3.0965925
H	-5.0345112	-3.1537332	2.8267607
H	-5.2442139	-2.6754459	0.3945018
H	-3.6786310	-1.1312875	-0.7135488
H	-1.6878887	-0.4566794	3.0725156
H	-3.2636831	-2.0159601	4.1678693
C	-0.7648188	4.0547562	3.0664941
C	0.2562673	3.1107839	2.9883323
C	0.0871720	1.9669746	2.2097734
C	-1.0995342	1.7607369	1.4964672
C	-2.1376933	2.6982395	1.6097869
C	-1.9679518	3.8387306	2.3894077
H	-0.6303373	4.9594556	3.6640373
H	1.1978810	3.2681405	3.5188858
H	0.8957687	1.2333389	2.1478975
H	-3.0812653	2.5488426	1.0780028
H	-2.7770963	4.5687617	2.4651858
Pd	0.8255689	-0.4676493	0.0547806
C	-1.5780919	-5.4818587	0.9972495
C	-0.4149383	-4.5248403	1.2385831
C	-0.5092696	-3.3449453	0.2701792
C	-0.5403109	-3.8102633	-1.2009072
C	-1.6773976	-4.8004308	-1.4254437
C	-1.6116400	-5.9679674	-0.4476732
H	-2.5243248	-4.9622817	1.2325862
H	-1.5073899	-6.3340993	1.6912139

H	0.5450058	-5.0474219	1.0910332
H	-0.4299847	-4.1546025	2.2754804
H	-1.4401328	-2.7996402	0.4700896
H	-0.6421370	-2.9403523	-1.8697147
H	0.4251621	-4.2900386	-1.4379541
H	-2.6429154	-4.2763519	-1.3111292
H	-1.6376006	-5.1614405	-2.4656184
H	-0.7019847	-6.5618252	-0.6494489
H	-2.4671242	-6.6436449	-0.6055242
C	-1.9377584	1.0221629	-1.2014988
C	-0.9124916	1.5896177	-2.1808333
H	-2.6850084	1.7848914	-0.9303131
H	-2.4812886	0.2009291	-1.6903905
H	-1.4725090	2.1019525	-2.9776434
H	-0.3835742	0.7586099	-2.6786149
H	0.4785604	3.2601693	-2.3350368
H	-0.3445354	3.1618333	-0.7658631
C	0.6188256	-2.3750921	0.3775124
O	1.8041764	-2.6059686	0.4109118

Pd(dppp)IchexCO **37**
E (E_h) = -2500.380608
OC (E_h) = -0.000681856
dU_{298} ($kJ*mol^{-1}$) = 1674.05
dS_{298} ($kJ*mol^{-1}*K^{-1}$) = 1.02399

76
Energy =

P	1.5455443	1.4859931	-1.0283071
P	-1.3964333	0.6078586	0.3186858
C	4.6582342	3.8678381	1.4192966
C	3.3296712	4.2904161	1.3941584
C	2.3894497	3.6042185	0.6262579
C	2.7713238	2.4865174	-0.1276133
C	4.1080668	2.0615001	-0.0912742
C	5.0441367	2.7522018	0.6741667
H	5.3942479	4.4072384	2.0204998
H	3.0184875	5.1616930	1.9757340
H	1.3535451	3.9478008	0.6285420
H	4.4144820	1.1811046	-0.6607491
H	6.0827992	2.4131580	0.6914889
C	3.4508536	0.4021834	-5.0953350
C	2.6891439	-0.5358271	-4.4000704
C	2.1434666	-0.2091332	-3.1581925
C	2.3489041	1.0624547	-2.6116898
C	3.1128237	2.0027993	-3.3165529
C	3.6639745	1.6714078	-4.5518150
H	3.8847308	0.1441838	-6.0646747
H	2.5246917	-1.5309927	-4.8201021

H	1.5653196	-0.9485382	-2.5977372
H	3.2850550	2.9969683	-2.8945975
H	4.2634089	2.4066763	-5.0942284
C	0.2484159	2.6773833	-1.5802755
C	-4.7494281	-2.2934956	1.6106022
C	-4.4354654	-2.1513369	0.2586252
C	-3.4702918	-1.2300316	-0.1422356
C	-2.8134736	-0.4345063	0.8065972
C	-3.1389841	-0.5760197	2.1621580
C	-4.1015693	-1.5015298	2.5592620
H	-5.5005067	-3.0219655	1.9250534
H	-4.9386704	-2.7669805	-0.4906384
H	-3.2203763	-1.1559108	-1.2031558
H	-2.6262149	0.0282830	2.9135598
H	-4.3437456	-1.6066252	3.6194498
C	-1.2284436	3.9391697	3.5212082
C	-0.1527434	3.0697134	3.3523210
C	-0.2143812	2.0628672	2.3885334
C	-1.3511362	1.9256354	1.5840005
C	-2.4355343	2.7959182	1.7669994
C	-2.3720047	3.7987585	2.7304038
H	-1.1804360	4.7291971	4.2746992
H	0.7424427	3.1727031	3.9697905
H	0.6248163	1.3735546	2.2557705
H	-3.3403440	2.6887131	1.1623830
H	-3.2196700	4.4745057	2.8670104
Pd	0.6653997	-0.3595074	0.2249958
I	3.0019878	-1.6898037	0.3044016
C	-0.8646221	-5.5495616	1.2208759
C	-0.1244425	-4.2968473	1.6753212
C	-0.6363870	-3.0605014	0.9334335
C	-0.5418468	-3.2376247	-0.5754227
C	-1.2927016	-4.4862050	-1.0242975
C	-0.7918374	-5.7279821	-0.2925489
H	-1.9243112	-5.4717007	1.5259712
H	-0.4561195	-6.4364565	1.7326853
H	0.9562376	-4.4032149	1.4727432
H	-0.2343866	-4.1404554	2.7588890
H	-1.7046814	-2.9311226	1.1982997
H	-0.9318788	-2.3410697	-1.0864137
H	0.5224014	-3.3156273	-0.8578734
H	-2.3707002	-4.3521437	-0.8181955
H	-1.1954720	-4.6170504	-2.1144181
H	0.2574479	-5.9175652	-0.5831859
H	-1.3674282	-6.6156389	-0.6026284
C	-1.9847698	1.4453547	-1.2230407
C	-0.9142948	1.9055181	-2.2080261
H	-2.6016256	2.2971914	-0.8957477
H	-2.6660322	0.7450934	-1.7263437

H	-1.4080841	2.5396635	-2.9601509
H	-0.5277071	1.0344064	-2.7647690
H	0.7010291	3.3813065	-2.2947110
H	-0.0982499	3.2665204	-0.7167474
C	-0.0013587	-1.7893556	1.4809717
O	0.1062512	-1.6033419	2.6679192

PdLImorphchexCO+H+ **38**
E (E_h) = -2097.399114
OC (E_h) = -0.000715971
dU_{298} (kJ*mol^{-1}) = 1564.03
dS_{298} (kJ*mol^{-1}*K^{-1}) = 0.95413
70

P	0.2610665	-0.7378061	0.5241309
C	-3.9436638	-2.2452536	1.7411108
C	-3.0687422	-1.7771306	2.7213292
C	-1.7797814	-1.3723430	2.3792031
C	-1.3525915	-1.4334462	1.0477439
C	-2.2311591	-1.9188476	0.0681159
C	-3.5190925	-2.3208332	0.4143026
H	-4.9551404	-2.5570065	2.0122275
H	-3.3902763	-1.7237116	3.7641368
H	-1.1116823	-1.0038582	3.1591703
H	-1.9131828	-1.9810425	-0.9748816
H	-4.1940388	-2.6945899	-0.3593349
C	1.2154131	-3.4221323	-3.1080380
C	1.1305534	-2.0390657	-3.2549774
C	0.8652093	-1.2364522	-2.1449675
C	0.6881843	-1.8123124	-0.8827013
C	0.7421149	-3.2083887	-0.7474253
C	1.0149511	-4.0062706	-1.8547382
H	1.4248580	-4.0523656	-3.9757630
H	1.2650143	-1.5799659	-4.2370138
H	0.7737685	-0.1533173	-2.2576400
H	0.5504844	-3.6767776	0.2210239
H	1.0614022	-5.0919729	-1.7419862
C	3.2069211	-1.3716448	4.0300729
C	3.2963598	-2.2145257	2.9232914
C	2.4037045	-2.0784304	1.8594853
C	1.4157055	-1.0886727	1.8962833
C	1.3451085	-0.2258575	3.0025528
C	2.2279381	-0.3773069	4.0684993
H	3.9086517	-1.4816519	4.8603456
H	4.0703814	-2.9845041	2.8809243
H	2.5016211	-2.7344452	0.9933788
H	0.6111581	0.5862678	3.0151945
H	2.1615102	0.2973650	4.9252291
Pd	0.3848718	1.5197586	0.1072636
C	-1.4344903	1.6941485	0.8344342

O	-1.5277358	1.9262278	2.0136212
C	-3.6853160	1.0852203	-2.2948618
C	-2.3858331	1.2891799	-1.5244515
C	-2.6630904	1.5512416	-0.0509427
C	-3.6017590	2.7474580	0.1378512
C	-4.8959527	2.5516860	-0.6436005
C	-4.6274226	2.2717564	-2.1186862
H	-4.1817877	0.1674248	-1.9288213
H	-3.4694383	0.9192575	-3.3626912
H	-1.8310025	2.1478093	-1.9407605
H	-1.7280755	0.4121083	-1.6366569
H	-3.1673140	0.6611159	0.3738535
H	-3.8048219	2.8840214	1.2106178
H	-3.0833556	3.6576312	-0.2119658
H	-5.4544295	1.7024494	-0.2091767
H	-5.5395644	3.4394440	-0.5309253
H	-4.1693993	3.1660351	-2.5786956
H	-5.5743477	2.0941313	-2.6542374
C	4.5774486	1.7182033	-1.9205237
C	3.1437706	1.2645119	-1.7096783
N	2.6349513	1.6946395	-0.4042557
C	3.5543226	1.2791540	0.6609235
C	4.9753559	1.7281883	0.3725320
O	5.4127161	1.2621045	-0.8827158
H	4.6131863	2.8259869	-1.9804894
H	4.9743154	1.3156768	-2.8641104
H	3.1002412	0.1654524	-1.7513291
H	2.4912875	1.6636549	-2.5009806
H	3.2037759	1.6843085	1.6214168
H	3.5222978	0.1816439	0.7341210
H	5.0328332	2.8360973	0.4083883
H	5.6659391	1.3297899	1.1304216
H	2.5995497	2.7192486	-0.4118238
I	0.2555450	4.1615303	-0.4613491

trans-PdL2morphchexCO **39**
E (E_h) = -2834.65764
OC (E_h) = -0.000217445
dU_{298} (kJ*mol^{-1}) = 2263.76
dS_{298} (kJ*mol^{-1}*K^{-1}) = 1.25024

102

P	0.0799649	-2.4465385	0.4989699
P	0.3847489	2.1629218	-0.0728507
C	-4.2853252	-3.9757402	0.4795004
C	-3.7071944	-3.5737058	1.6829410
C	-2.3736982	-3.1656966	1.7225325
C	-1.6089330	-3.1529045	0.5525364
C	-2.1924953	-3.5652143	-0.6549850

C	-3.5217203	-3.9772377	-0.6897106
H	-5.3309787	-4.2916158	0.4518187
H	-4.2974915	-3.5758924	2.6024724
H	-1.9349945	-2.8454179	2.6695408
H	-1.6071966	-3.5524823	-1.5782824
H	-3.9661818	-4.2937962	-1.6363913
C	2.3040811	-5.4656881	-2.1903236
C	2.5269628	-4.0965485	-2.3275849
C	1.8517269	-3.1933674	-1.5059330
C	0.9509374	-3.6587345	-0.5456456
C	0.7219117	-5.0368187	-0.4137801
C	1.4017086	-5.9351301	-1.2319233
H	2.8324728	-6.1738340	-2.8336492
H	3.2309609	-3.7260096	-3.0766460
H	2.0144534	-2.1156696	-1.5770706
H	0.0050575	-5.4086165	0.3227661
H	1.2238097	-7.0079575	-1.1249915
C	1.8329723	-2.7557812	4.7680446
C	2.1752228	-3.7232288	3.8236821
C	1.6359028	-3.6733130	2.5383300
C	0.7478082	-2.6503662	2.1907981
C	0.4108312	-1.6723072	3.1403759
C	0.9478968	-1.7325910	4.4246633
H	2.2620758	-2.7954841	5.7723482
H	2.8743742	-4.5214901	4.0846914
H	1.9274866	-4.4243681	1.8017320
H	-0.2673527	-0.8579013	2.8641784
H	0.6810915	-0.9673321	5.1578082
C	-3.5369667	4.6325323	0.2679966
C	-2.9107707	4.5147149	-0.9715168
C	-1.7187393	3.7990793	-1.0919401
C	-1.1451707	3.1843941	0.0268236
C	-1.7827940	3.3055848	1.2704968
C	-2.9662129	4.0282736	1.3895748
H	-4.4683851	5.1961504	0.3618685
H	-3.3456747	4.9888587	-1.8547850
H	-1.2336051	3.7334090	-2.0676057
H	-1.3529276	2.8262720	2.1522311
H	-3.4497258	4.1145709	2.3656787
C	2.6237010	3.6860696	3.6730729
C	2.5022272	2.3185109	3.4298209
C	1.8462189	1.8687200	2.2846940
C	1.3009991	2.7835488	1.3788543
C	1.4037608	4.1575089	1.6404710
C	2.0721789	4.6051862	2.7777337
H	3.1412034	4.0395626	4.5684425
H	2.9189757	1.5942592	4.1340442
H	1.7463756	0.8012662	2.0840136
H	0.9474474	4.8824980	0.9615262

H	2.1544185	5.6774021	2.9715738
C	2.3876129	3.7048361	-3.9473993
C	2.8669322	4.1495286	-2.7163726
C	2.2794269	3.7086537	-1.5299184
C	1.2090661	2.8110405	-1.5675251
C	0.7448844	2.3508799	-2.8090877
C	1.3223118	2.8040299	-3.9922661
H	2.8501820	4.0532373	-4.8740473
H	3.7098472	4.8435111	-2.6743848
H	2.6729827	4.0566794	-0.5734874
H	-0.0600745	1.6124222	-2.8477642
H	0.9493958	2.4389840	-4.9521216
Pd	0.2627358	-0.1705504	0.0139843
C	-1.6529767	0.0090258	0.5733523
O	-1.9185020	0.1751180	1.7491422
C	-3.5128294	-0.1573188	-2.8511172
C	-2.3594577	0.1163440	-1.8922961
C	-2.7795005	-0.1109971	-0.4463532
C	-4.0102630	0.7154202	-0.0800397
C	-5.1604431	0.4398181	-1.0412879
C	-4.7425420	0.6704457	-2.4903736
H	-3.7709493	-1.2312633	-2.8043964
H	-3.2020743	0.0455465	-3.8891803
H	-2.0353825	1.1652076	-2.0072239
H	-1.4877177	-0.5161551	-2.1336535
H	-3.0601075	-1.1779764	-0.3526842
H	-4.2970040	0.4969237	0.9592003
H	-3.7471036	1.7857203	-0.1168576
H	-5.4907163	-0.6086047	-0.9219898
H	-6.0275860	1.0720814	-0.7874725
H	-4.5079731	1.7417954	-2.6297364
H	-5.5757076	0.4399964	-3.1746701
C	4.3608797	0.8338879	-1.3006167
C	3.0601002	0.0784930	-1.5377909
N	2.3663292	-0.1761950	-0.3106032
C	3.2361133	-0.8703222	0.5949228
C	4.5473633	-0.1250275	0.8065681
O	5.2031374	0.1236878	-0.4178562
H	4.1286686	1.8308665	-0.8763527
H	4.9242016	0.9735220	-2.2383568
H	3.3554538	-0.8601752	-2.0821313
H	2.4366331	0.6520642	-2.2381367
H	2.7614109	-1.0126533	1.5801524
H	3.5028626	-1.8999170	0.2360759
H	4.3394362	0.8363434	1.3200591
H	5.2435127	-0.7114694	1.4290621

PdLmorphchexCO **40**
E (E_h) = -1798.97132

OC (E_h) = 2.07988E-05
dU_{298} (kJ*mol^{-1}) = 1521.69
dS_{298} (kJ*mol^{-1}*K^{-1}) = 0.9347

68

P	0.5351798	-1.5137373	0.3895057
C	-4.0478640	-1.5133921	0.9541058
C	-3.2142127	-1.3681364	2.0622040
C	-1.8286703	-1.4308585	1.9112655
C	-1.2685452	-1.6323324	0.6445757
C	-2.1106251	-1.7769409	-0.4671864
C	-3.4931342	-1.7246952	-0.3099597
H	-5.1327245	-1.4639842	1.0741624
H	-3.6425638	-1.2053268	3.0540465
H	-1.1817067	-1.3116987	2.7835154
H	-1.6853570	-1.9282074	-1.4629419
H	-4.1419618	-1.8434762	-1.1809643
C	1.7325725	-5.1847869	-2.1589207
C	2.5069358	-4.0242175	-2.1629025
C	2.1147886	-2.9227851	-1.4044140
C	0.9493847	-2.9757551	-0.6287751
C	0.1748420	-4.1435002	-0.6304237
C	0.5668499	-5.2419090	-1.3940511
H	2.0357365	-6.0466720	-2.7583470
H	3.4173927	-3.9720749	-2.7645370
H	2.7188724	-2.0096129	-1.4175461
H	-0.7429799	-4.1960465	-0.0399324
H	-0.0439201	-6.1479463	-1.3915775
C	2.3166491	-2.3915940	4.5660632
C	1.7554925	-3.4344749	3.8256826
C	1.2210131	-3.1866344	2.5638148
C	1.2453054	-1.8901722	2.0327990
C	1.8118143	-0.8471318	2.7762916
C	2.3429292	-1.1001446	4.0415875
H	2.7372503	-2.5892366	5.5551788
H	1.7367144	-4.4483223	4.2327518
H	0.7901475	-4.0101411	1.9894880
H	1.8216924	0.1667964	2.3677447
H	2.7817183	-0.2817309	4.6173672
Pd	1.3572509	0.4443229	-0.4809941
C	0.0934480	1.5038412	0.5656396
O	0.4266545	1.9623835	1.6284276
C	-2.8841292	1.6887288	-1.9604879
C	-1.4336788	1.5415184	-1.5146570
C	-1.3045272	1.7197403	-0.0063651
C	-1.8912934	3.0546127	0.4547263
C	-3.3393890	3.1981922	0.0011573
C	-3.4770574	3.0185878	-1.5069062
H	-3.4758259	0.8609941	-1.5306704

H -2.9521498 1.5885390 -3.0557654
H -0.8168098 2.3018776 -2.0220632
H -1.0276748 0.5617512 -1.8150583
H -1.8865185 0.9099056 0.4764096
H -1.8078620 3.1342168 1.5484831
H -1.2855981 3.8764974 0.0313948
H -3.9532780 2.4359258 0.5148223
H -3.7347977 4.1791838 0.3112926
H -2.9503230 3.8443217 -2.0192073
H -4.5352440 3.0894630 -1.8071905
C 1.6357119 3.9129407 -2.0812964
C 2.1238786 2.5400515 -2.5270117
N 2.5445282 1.7238983 -1.4105952
C 3.5229446 2.4504327 -0.6275486
C 3.0137942 3.8225216 -0.2050491
O 2.6168674 4.5826296 -1.3257287
H 0.7132320 3.7913740 -1.4759801
H 1.3983519 4.5524964 -2.9460587
H 2.9794496 2.7029444 -3.2182997
H 1.3354455 2.0311914 -3.1039531
H 3.8088863 1.8715096 0.2652044
H 4.4468524 2.6036320 -1.2268775
H 2.1603620 3.6940016 0.4899788
H 3.7994828 4.3966514 0.3105573

cis-Ldiss-PdLIchexCO **41**
E (E_h) = -1809.754664
OC (E_h) = -0.000332527
dU_{298} (kJ*mol^{-1}) = 1200.42
dS_{298} (kJ*mol^{-1}*K^{-1}) = 0.88539

55
I -0.4174807 -2.8339307 -0.2740862
P 1.5371647 0.4990242 -0.0964580
C 4.4674465 -0.4853697 3.3423710
C 4.3738941 0.8297791 2.8891350
C 3.4999105 1.1542197 1.8505135
C 2.7107976 0.1596446 1.2629849
C 2.7992325 -1.1607491 1.7291025
C 3.6794750 -1.4804133 2.7594203
H 5.1534483 -0.7368043 4.1550022
H 4.9861455 1.6121901 3.3442062
H 3.4346262 2.1861423 1.4981750
H 2.1738152 -1.9389807 1.2806859
H 3.7458743 -2.5118354 3.1141496
C 3.5609557 -1.1051191 -3.9245198
C 2.1864940 -0.8641230 -3.8866944
C 1.5926427 -0.3995293 -2.7157055
C 2.3733279 -0.1586517 -1.5780963

C 3.7501333 -0.4058913 -1.6179919
C 4.3403159 -0.8790128 -2.7898155
H 4.0253931 -1.4777018 -4.8409288
H 1.5717081 -1.0502727 -4.7705923
H 0.5124522 -0.2328141 -2.6777993
H 4.3638716 -0.2323640 -0.7305862
H 5.4155238 -1.0723044 -2.8151103
C 1.6478186 5.1026991 -0.5157791
C 2.5899318 4.3359781 -1.2030671
C 2.5717836 2.9457052 -1.1005319
C 1.6058518 2.3131889 -0.3082159
C 0.6550821 3.0875679 0.3698114
C 0.6798939 4.4775223 0.2712577
H 1.6638902 6.1921551 -0.5994767
H 3.3447765 4.8232952 -1.8249569
H 3.3109653 2.3504213 -1.6420521
H -0.1151628 2.5980655 0.9745958
H -0.0649351 5.0731588 0.8043433
Pd -0.6965351 -0.3122071 0.3247698
C -2.5509400 -0.3614142 0.8307447
O -2.5767300 0.8010042 1.1552867
C -5.2585043 -2.5473563 -0.7234506
C -4.1231129 -1.5333073 -0.6519175
C -3.6699840 -1.3455986 0.8084205
C -4.8339377 -0.9352294 1.7106095
C -5.9653620 -1.9533475 1.6201194
C -6.4235144 -2.1581843 0.1800815
H -4.8724146 -3.5373989 -0.4219787
H -5.5942931 -2.6463794 -1.7680261
H -4.4630901 -0.5598920 -1.0466884
H -3.2675953 -1.8568887 -1.2638238
H -3.2361343 -2.3040681 1.1419762
H -4.4850868 -0.8309958 2.7499570
H -5.1953765 0.0580575 1.3935634
H -5.6187090 -2.9172230 2.0339512
H -6.8077989 -1.6280608 2.2512859
H -6.8747118 -1.2212140 -0.1938215
H -7.2132329 -2.9251863 0.1356504

chexCOI **42**
E (E_h) = -646.0889437
OC (E_h) = -1.47277E-05
dU_{298} (kJ*mol^{-1}) = 451.29
dS_{298} (kJ*mol^{-1}*K^{-1}) = 0.41555

20
C 1.6395828 1.5517759 -1.0487820
O 2.7918026 1.4091434 -0.8512513
C -0.2452978 -0.3934740 1.6715473

C 0.9299688 0.2690680 0.9616923
C 0.4751178 0.9099145 -0.3505092
C -0.2103890 -0.1168457 -1.2671927
C -1.3757735 -0.7811097 -0.5422424
C -0.9396969 -1.4121892 0.7751264
H -0.9706562 0.3824823 1.9753392
H 0.1075724 -0.8706596 2.5995922
H 1.7061146 -0.4822459 0.7383029
H 1.3991143 1.0285266 1.6060517
H -0.2475746 1.7188098 -0.1450628
H -0.5570559 0.3717256 -2.1902983
H 0.5321714 -0.8788144 -1.5615129
H -2.1551621 -0.0231565 -0.3464498
H -1.8314995 -1.5360349 -1.2023111
H -0.2449110 -2.2455761 0.5663455
H -1.8075974 -1.8501496 1.2934503
I 1.0041694 2.9488097 -2.6818353

PPh_3
E (E_h) = -1035.6597
OC (E_h) = -0.000148643
dU_{298} (kJ*mol^{-1}) = 733.27
dS_{298} (kJ*mol^{-1}*K^{-1}) = 0.57265

34
C 3.4970645 -2.3442482 0.5159969
C 3.1288388 -1.1127543 1.0576354
C 2.0804674 -0.3828624 0.4961682
C 1.3924679 -0.8756488 -0.6195671
C 1.7791996 -2.1083342 -1.1664083
C 2.8175975 -2.8425989 -0.5971146
H 4.3176697 -2.9147569 0.9581898
H 3.6589196 -0.7164621 1.9275570
H 1.7942392 0.5773813 0.9318712
H 1.2591197 -2.4971982 -2.0472596
H 3.1045282 -3.8041587 -1.0303240
C -3.7776672 -1.8573778 0.5266846
C -2.5263499 -2.1442938 1.0722070
C -1.3718438 -1.6011502 0.5073878
C -1.4570815 -0.7685526 -0.6154384
C -2.7185544 -0.4970464 -1.1658805
C -3.8720180 -1.0295165 -0.5934422
H -4.6806525 -2.2830938 0.9714156
H -2.4463442 -2.7939257 1.9476202
H -0.3965487 -1.8257710 0.9457501
H -2.7970300 0.1399956 -2.0522340
H -4.8487322 -0.8051912 -1.0296625
C 0.2837783 4.2002306 0.5244865
C -0.5966588 3.2641377 1.0668531

C -0.7062933 1.9926540 0.5026043
C 0.0608990 1.6459707 -0.6164628
C 0.9329776 2.5987622 -1.1640480
C 1.0513252 3.8638629 -0.5921358
H 0.3689454 5.1950734 0.9688276
H -1.2020606 3.5228438 1.9393869
H -1.3933723 1.2635218 0.9386023
H 1.5268437 2.3450500 -2.0475281
H 1.7389302 4.5943404 -1.0259410
P -0.0026040 0.0011171 -1.4357977

Iodine radical
E (E_h) = -297.69167
OC (E_h) = -5.83559E-05
dU_{298} (kJ*mol^{-1}) = 0
dS_{298} (kJ*mol^{-1}*K^{-1}) = 0

1
I 0.0000000 0.0000000 0.0000000

Hydrogen iodide
E (E_h) = -298.3292118
OC (E_h) = -0.000120655
dU_{298} (kJ*mol^{-1}) = 19.51
dS_{298} (kJ*mol^{-1}*K^{-1}) = 0.2067

2
I 0.0000000 0.0000000 -0.8094907
H 0.0000000 0.0000000 0.8094907

Carbon monoxide
E (E_h) = -113.2340801
OC (E_h) = -1.16033E-05
dU_{298} (kJ*mol^{-1}) = 19.16
dS_{298} (kJ*mol^{-1}*K^{-1}) = 0.19776

2
C 0.0000000 0.0000000 0.5639015
O 0.0000000 0.0000000 -0.5639015

Iodide
E (E_h) = -297.8915862
OC (E_h) = -0.008836426
dU_{298} (kJ*mol^{-1}) = 0
dS_{298} (kJ*mol^{-1}*K^{-1}) = 0

1
I 0.0000000 0.0000000 0.0000000

Hydroxide ion
E (E_h) = -75.83165501
OC (E_h) = -0.004905795
dU_{298} (kJ*mol^{-1}) = 27.19
dS_{298} (kJ*mol^{-1}*K^{-1}) = 0.1726

2

O	0.00000	0.00000	-0.05770
H	0.00000	0.00000	0.91630

Water
E (E_h) = -76.39821502
OC (E_h) = -0.000223884
dU_{298} (kJ*mol^{-1}) = 60.66
dS_{298} (kJ*mol^{-1}*K^{-1}) = 0.1891

3

O	0.0000000	0.0000000	-0.4019172
H	-0.7529559	0.0000000	0.2009586
H	0.7529559	0.0000000	0.2009586

Cyclohexene
E (E_h) = -234.4612806
OC (E_h) = 2.09601E-05
dU_{298} (kJ*mol^{-1}) = 381.52
dS_{298} (kJ*mol^{-1}*K^{-1}) = 0.31227

16

C	-1.1837321	-0.6993146	0.6036312
C	0.0372985	-0.5239843	1.4589111
C	1.1592392	0.0668907	1.0275506
C	1.3073269	0.6522038	-0.3467268
C	-0.0361379	0.8015117	-1.0550042
C	-0.8915344	-0.4472522	-0.8728295
H	-1.9777655	-0.0145526	0.9587923
H	-1.5941463	-1.7134421	0.7483369
H	-0.0070026	-0.9068044	2.4850733
H	2.0214550	0.1371890	1.7007284
H	1.8191779	1.6276240	-0.2800243
H	1.9853260	0.0120032	-0.9433689
H	-0.5740187	1.6692673	-0.6329895
H	0.1179221	1.0177033	-2.1240696
H	-0.3508339	-1.3161434	-1.2888573
H	-1.8325742	-0.3628996	-1.4391536

Cyclohexane
E (E_h) = -235.6905301
OC (E_h) = 4.95258E-05
dU_{298} (kJ*mol^{-1}) = 442.46

dS_{298} (kJ*mol^{-1}*K^{-1}) = 0.31508

18

C	1.0796885	0.1778660	-0.9880008
C	-0.3879880	0.2262458	-1.4041904
C	-1.2798616	0.6861772	-0.2541522
C	-1.0796782	-0.1778495	0.9880115
C	0.3880024	-0.2262507	1.4041856
C	1.2798584	-0.6861944	0.2541397
H	1.4276680	1.2051014	-0.7727829
H	1.7025235	-0.1933041	-1.8186436
H	-0.7053266	-0.7830690	-1.7253064
H	-0.5178649	0.8854464	-2.2783931
H	-2.3385976	0.6771369	-0.5617870
H	-1.0365148	1.7359485	-0.0064493
H	-1.7025026	0.1933847	1.8186358
H	-1.4276836	-1.2050906	0.7728601
H	0.7053342	0.7830651	1.7253050
H	0.5178916	-0.8854548	2.2783837
H	1.0364636	-1.7359433	0.0063909
H	2.3385879	-0.6772156	0.5617933

Oxidative addition to PdL2 TS 1

E (E_h) = -2732.135598

OC (E_h) = -0.00033046

dU_{298} (kJ*mol^{-1}) = 1903.29

dS_{298} (kJ*mol^{-1}*K^{-1}) = 1.16884

Imaginary frequencies = 1

87

P	1.2531763	-0.9555910	0.9165973
P	-0.9057327	2.5032775	1.4716338
C	-3.7622048	-3.1456545	0.4919097
C	-2.4955670	-2.4056128	0.0831521
C	-2.5268364	-0.9233320	0.3017830
C	-3.8239809	-0.2211212	0.0408361
C	-5.0735259	-0.9889570	0.4452089
C	-5.0158324	-2.4413370	-0.0194361
H	-3.7976969	-3.2131093	1.5912650
H	-3.7157094	-4.1781142	0.1107125
H	-2.3628525	-2.5089998	-1.0158540
H	-1.5916805	-2.8482080	0.5263750
H	-1.6826459	-0.4037744	-0.1975044
H	-3.8072631	0.7989496	0.4486733
H	-3.8251559	-0.0873519	-1.0628204
H	-5.1772365	-0.9542275	1.5420858
H	-5.9582564	-0.4829299	0.0271425
H	-5.0247874	-2.4707268	-1.1247890
H	-5.9160495	-2.9828904	0.3120792

C	1.4709861	-5.5309143	1.6317442
C	0.6361902	-4.7190152	2.4001828
C	0.5841084	-3.3463567	2.1594725
C	1.3759815	-2.7719879	1.1568971
C	2.2118237	-3.5931127	0.3884800
C	2.2566915	-4.9662687	0.6248794
H	1.5062329	-6.6081298	1.8133060
H	0.0132193	-5.1569942	3.1841402
H	-0.0890711	-2.7069256	2.7398370
H	2.8296219	-3.1560249	-0.4002678
H	2.9096079	-5.6003570	0.0195619
C	2.3338941	-0.4387589	-3.5696754
C	1.1341908	-1.0357731	-3.1737588
C	0.8499222	-1.1975981	-1.8210976
C	1.7623702	-0.7777045	-0.8405909
C	2.9654888	-0.1908991	-1.2459782
C	3.2463950	-0.0202515	-2.6030711
H	2.5550347	-0.3021873	-4.6310902
H	0.4138150	-1.3712267	-3.9241071
H	-0.0941438	-1.6611053	-1.5207027
H	3.6894250	0.1425258	-0.4996695
H	4.1888379	0.4445085	-2.9038506
C	4.8477605	0.7979422	3.2672790
C	3.9512611	1.6274633	2.5899121
C	2.8842435	1.0750638	1.8871117
C	2.7091200	-0.3169122	1.8334897
C	3.6149250	-1.1422653	2.5092963
C	4.6750533	-0.5850357	3.2268050
H	5.6785494	1.2311212	3.8298521
H	4.0755026	2.7128362	2.6200037
H	2.1727264	1.7266337	1.3723081
H	3.4925050	-2.2272846	2.4796512
H	5.3714658	-1.2400642	3.7565473
C	-5.2483788	3.7480613	0.4543462
C	-4.2354674	3.9822671	-0.4746503
C	-2.9092088	3.6775124	-0.1630779
C	-2.5838512	3.1260083	1.0816596
C	-3.6099828	2.8889653	2.0113740
C	-4.9296565	3.2049816	1.7018066
H	-6.2858763	3.9878435	0.2087372
H	-4.4760582	4.4093944	-1.4516763
H	-2.1227501	3.8657537	-0.8980599
H	-3.3728045	2.4354128	2.9774407
H	-5.7174822	3.0161649	2.4354566
C	0.2111885	4.5174249	5.4946272
C	0.7723902	3.2975555	5.1154082
C	0.4017038	2.7045768	3.9093960
C	-0.5193520	3.3331069	3.0609018
C	-1.0852274	4.5539321	3.4530701

C -0.7217328 5.1412184 4.6643661
H 0.4941446 4.9797142 6.4437253
H 1.4947800 2.7984409 5.7660865
H 0.8205569 1.7369366 3.6183681
H -1.8221906 5.0454581 2.8127456
H -1.1705093 6.0923380 4.9620010
C 1.7606168 4.6306802 -1.6767583
C 1.3093595 5.3573695 -0.5726851
C 0.5054865 4.7469187 0.3885801
C 0.1488561 3.3985056 0.2581501
C 0.6134066 2.6754127 -0.8473723
C 1.4097250 3.2881553 -1.8144409
H 2.3934869 5.1121563 -2.4266774
H 1.5877998 6.4081443 -0.4581977
H 0.1617291 5.3245875 1.2498158
H 0.3608143 1.6145572 -0.9330899
H 1.7675395 2.7059581 -2.6671284
Pd -0.6975865 0.1718751 1.4927320
I -2.5926653 -0.8764184 3.0437718

Associative CO binding TS 3
E (E_h) = -2845.408616
OC (E_h) = -0.000522225
dU_{298} (kJ*mol^{-1}) = 1935.93
dS_{298} (kJ*mol^{-1}*K^{-1}) = 1.19307
Imaginary frequencies = 1

89
P -2.0038936 2.5783098 0.1185779
C -6.1024566 3.8787813 -1.5850796
C -5.4398138 2.7744723 -2.1155017
C -4.2053841 2.3905021 -1.5973106
C -3.6187255 3.1109874 -0.5534866
C -4.2935021 4.2175780 -0.0194569
C -5.5287330 4.5987886 -0.5355057
H -7.0760848 4.1753091 -1.9826024
H -5.8918950 2.1948782 -2.9229327
H -3.6952940 1.5096571 -1.9896917
H -3.8604899 4.7850011 0.8068583
H -6.0493835 5.4606091 -0.1113242
C -2.7507919 2.7068570 4.7075229
C -2.8237167 1.4972522 4.0188933
C -2.5721394 1.4577857 2.6487387
C -2.2536217 2.6251805 1.9442988
C -2.1709320 3.8360076 2.6481798
C -2.4203849 3.8744552 4.0187443
H -2.9432709 2.7401028 5.7826134
H -3.0718945 0.5721582 4.5450267
H -2.6119515 0.4995904 2.1320377

H	-1.8975136	4.7574503	2.1299596
H	-2.3505688	4.8256513	4.5518059
C	0.9236422	6.1099388	-0.4735951
C	1.2373655	5.0498968	0.3796811
C	0.3345520	4.0060215	0.5558284
C	-0.8991930	4.0154452	-0.1124893
C	-1.2115516	5.0839420	-0.9584385
C	-0.3001422	6.1249916	-1.1398361
H	1.6353700	6.9266102	-0.6163551
H	2.1941273	5.0323245	0.9066535
H	0.5932642	3.1772415	1.2198690
H	-2.1708940	5.1107333	-1.4794040
H	-0.5537216	6.9534820	-1.8053572
Pd	-1.2795507	0.5111394	-0.5925167
P	-3.5665176	-0.8785759	0.0127513
C	-7.7866403	1.0735092	0.0305846
C	-6.7998551	1.4948701	0.9231909
C	-5.5461679	0.8927027	0.9141172
C	-5.2579827	-0.1539241	0.0239134
C	-6.2560563	-0.5726834	-0.8640534
C	-7.5096941	0.0407086	-0.8620331
H	-8.7680275	1.5541607	0.0290710
H	-7.0012833	2.3097622	1.6222964
H	-4.7831710	1.2484166	1.6083828
H	-6.0573324	-1.3840224	-1.5666241
H	-8.2752719	-0.2978739	-1.5647937
C	-4.0268362	-4.0559638	-3.3175473
C	-3.6601245	-2.7435661	-3.6209997
C	-3.5034433	-1.8118600	-2.5989707
C	-3.7369422	-2.1743592	-1.2660898
C	-4.1083505	-3.4884970	-0.9679456
C	-4.2458428	-4.4270111	-1.9910871
H	-4.1360140	-4.7926866	-4.1173859
H	-3.4758007	-2.4495630	-4.6571237
H	-3.1761616	-0.7951559	-2.8353283
H	-4.2876874	-3.7832254	0.0687333
H	-4.5285737	-5.4545271	-1.7486639
C	-3.2502474	-3.1935180	4.0186786
C	-2.1774840	-3.1350021	3.1268578
C	-2.3037593	-2.4458716	1.9234839
C	-3.5142088	-1.8193591	1.5876828
C	-4.5900515	-1.8917069	2.4810511
C	-4.4546660	-2.5713769	3.6924194
H	-3.1468675	-3.7256777	4.9676403
H	-1.2310492	-3.6230422	3.3728299
H	-1.4594956	-2.3961263	1.2294951
H	-5.5403852	-1.4143339	2.2340359
H	-5.3010905	-2.6176804	4.3823358
C	-0.1689730	0.1823416	1.2364367

O 0.8760362 0.4356357 1.6231891
H -0.3486841 0.3770963 -2.9071174
C 1.7405858 2.2133628 -3.3053225
C 1.0042712 1.7888533 -2.0286014
C -0.4011495 1.3164953 -2.3381346
C -1.2108452 2.3042678 -3.1533563
C -0.4459986 2.7134520 -4.4187503
C 0.9398939 3.2486167 -4.0834129
H 1.9053992 1.3261384 -3.9429465
H 2.7384229 2.6036816 -3.0439088
H 0.9783112 2.6502435 -1.3441052
H 1.5624135 0.9872552 -1.5202897
H -2.1773683 1.8658301 -3.4423931
H -1.4315507 3.2179115 -2.5820521
H -0.3448120 1.8357048 -5.0823542
H -1.0338195 3.4624918 -4.9754906
H 0.8388971 4.1646573 -3.4725382
H 1.4727969 3.5394984 -5.0040247
I -0.0740720 -1.8648064 -1.3359375

Beta-hydride elimination TS 4
E (E_h) = -1696.476899
OC (E_h) = -0.000683414
dU_{298} (kJ*mol^{-1}) = 1154.23
dS_{298} (kJ*mol^{-1}*K^{-1}) = 0.79593
Imaginary frequencies = 1

53
I -0.0801109 0.2081688 0.5223042
C -4.0980343 2.6325179 -2.1444820
C -3.9538978 1.8413336 -0.8715323
C -2.8840324 2.1520944 -0.0303347
C -1.9698444 3.2748676 -0.4364802
C -1.6065120 3.2581140 -1.9242102
C -2.7622980 2.8344434 -2.8576810
H -4.5234231 3.6118163 -1.8532569
H -4.8288716 2.1661669 -2.8217601
H -3.7051013 0.1823563 -1.6825986
H -4.8558809 1.3910043 -0.4450202
H -2.9545622 1.9086960 1.0347936
H -2.4995116 4.2166801 -0.1936834
H -1.0568503 3.2640850 0.1760381
H -1.2291286 4.2529826 -2.2060502
H -0.7636267 2.5608450 -2.0563475
H -2.5040437 1.8785229 -3.3441636
H -2.8941420 3.5659963 -3.6693090
Pd -2.3673123 0.2107240 -0.9176664
P -1.9871502 -1.7912945 -1.9817815
C -2.8886102 -5.5022258 0.6245398

C	-2.3505351	-4.3468162	1.1933178
C	-2.0992512	-3.2284898	0.4012645
C	-2.3775735	-3.2634481	-0.9718937
C	-2.9187888	-4.4238907	-1.5391931
C	-3.1731972	-5.5383648	-0.7405542
H	-3.0901977	-6.3765662	1.2483618
H	-2.1285671	-4.3125741	2.2626426
H	-1.6770046	-2.3219997	0.8446909
H	-3.1446385	-4.4599283	-2.6073385
H	-3.5971461	-6.4397847	-1.1896230
C	-4.4466418	-2.5795563	-5.8299732
C	-5.0557396	-2.0436442	-4.6932625
C	-4.2996599	-1.7876142	-3.5528936
C	-2.9292576	-2.0772423	-3.5290600
C	-2.3253766	-2.6205223	-4.6687809
C	-3.0824946	-2.8645180	-5.8157164
H	-5.0376360	-2.7713476	-6.7288723
H	-6.1245189	-1.8166116	-4.6975694
H	-4.7812784	-1.3574719	-2.6699910
H	-1.2587779	-2.8548137	-4.6653279
H	-2.5991454	-3.2819397	-6.7023089
C	2.3860199	-2.2733615	-3.3776470
C	1.6938346	-1.0780222	-3.5854782
C	0.3817033	-0.9480125	-3.1416410
C	-0.2542465	-2.0196482	-2.5011995
C	0.4401415	-3.2141407	-2.2934880
C	1.7602299	-3.3370641	-2.7295508
H	3.4197121	-2.3722763	-3.7180927
H	2.1837599	-0.2392587	-4.0857163
H	-0.1522991	-0.0041995	-3.2841949
H	-0.0464445	-4.0514739	-1.7881232
H	2.3007599	-4.2714931	-2.5604056

Neutral carbonylative insertion TS 5
E (E_h) = -1809.738432
OC (E_h) = -0.000651665
dU_{298} (kJ*mol^{-1}) = 1195.11
dS_{298} (kJ*mol^{-1}*K^{-1}) = 0.85744
Imaginary frequencies = 1

55

P	-2.2893042	2.6901443	0.0772710
C	-2.9809113	-1.7503999	3.2954717
C	-2.5373210	-1.7591383	1.8302212
C	-3.3073550	-0.6920748	1.0678175
C	-4.8157566	-0.8471623	1.1561331
C	-5.2389629	-0.8450045	2.6268509
C	-4.4924379	-1.9101717	3.4232844
H	-2.6742731	-0.7974962	3.7610416

H	-2.4586037	-2.5525551	3.8419094
H	-2.7339777	-2.7564350	1.4007633
H	-1.4532354	-1.5815165	1.7546091
H	-3.0354994	0.2819718	1.5003088
H	-5.3199707	-0.0332648	0.6123910
H	-5.1305365	-1.7950174	0.6852963
H	-5.0304195	0.1505498	3.0572563
H	-6.3279952	-0.9990793	2.6984229
H	-4.7833679	-2.9100774	3.0532024
H	-4.7903093	-1.8714789	4.4836948
C	-4.8495634	5.7007610	-2.3112354
C	-3.7486349	6.1646862	-1.5894007
C	-2.9533450	5.2715238	-0.8739102
C	-3.2550823	3.9047478	-0.8813205
C	-4.3535445	3.4420609	-1.6167107
C	-5.1525011	4.3392154	-2.3233502
H	-5.4698587	6.4037153	-2.8727130
H	-3.5049218	7.2297711	-1.5847018
H	-2.0907902	5.6426850	-0.3158436
H	-4.5770913	2.3718621	-1.6457437
H	-6.0073947	3.9702940	-2.8947663
C	-4.0926608	2.6763744	4.3367054
C	-2.8051144	2.1879154	4.1084924
C	-2.2559907	2.2360218	2.8294394
C	-2.9849127	2.7905975	1.7656750
C	-4.2762597	3.2762913	1.9998230
C	-4.8255091	3.2176639	3.2811145
H	-4.5248392	2.6334297	5.3392060
H	-2.2250355	1.7622214	4.9307201
H	-1.2482897	1.8454532	2.6601705
H	-4.8566933	3.7065328	1.1803635
H	-5.8334541	3.6024570	3.4536484
C	1.9480011	4.5056964	0.3952128
C	0.9570411	4.9170149	1.2879609
C	-0.3218051	4.3686781	1.2147584
C	-0.6174760	3.4014962	0.2431780
C	0.3818117	2.9881057	-0.6468655
C	1.6588525	3.5421727	-0.5708709
H	2.9506606	4.9360038	0.4560844
H	1.1803219	5.6697116	2.0478670
H	-1.0897725	4.6912146	1.9218302
H	0.1546974	2.2343183	-1.4057627
H	2.4322327	3.2141316	-1.2694028
C	-2.9501604	-1.3594201	-0.7982069
Pd	-2.4929122	0.4385872	-0.7575630
I	-1.7644961	0.9146252	-3.2603593
O	-3.1895725	-2.4521110	-1.0732682

Cationic carbonylative insertion TS 6

E (E_h) = -2547.507158
OC (E_h) = 0.003207725
dU_{298} (kJ*mol^{-1}) = 1928.82
dS_{298} (kJ*mol^{-1}*K^{-1}) = 1.11406
Imaginary frequencies = 1

88

P	-0.2776236	-0.3235052	-0.4144418
P	-2.8643301	2.0485689	0.4631744
C	1.7228985	-4.1051895	1.3524197
C	2.1381112	-2.8519799	1.7968529
C	1.5538716	-1.6926243	1.2839362
C	0.5410094	-1.7855685	0.3239715
C	0.1312223	-3.0495746	-0.1261738
C	0.7209727	-4.2014585	0.3840612
H	2.1833462	-5.0102766	1.7554346
H	2.9290251	-2.7686012	2.5458879
H	1.9038213	-0.7190155	1.6302868
H	-0.6421472	-3.1394237	-0.8923454
H	0.3973999	-5.1802611	0.0226953
C	0.6485689	-0.8005526	-4.9096559
C	-0.6568648	-0.5432604	-4.4944113
C	-0.9381477	-0.3995123	-3.1362934
C	0.0853202	-0.5071605	-2.1891750
C	1.3937787	-0.7850918	-2.6108027
C	1.6720574	-0.9254995	-3.9672687
H	0.8704549	-0.9148139	-5.9733726
H	-1.4624378	-0.4597934	-5.2272634
H	-1.9664140	-0.2174929	-2.8106112
H	2.1962022	-0.8974137	-1.8769311
H	2.6930433	-1.1396026	-4.2914007
C	2.0920672	3.3044487	1.1777697
C	2.1436726	3.0002315	-0.1815964
C	1.4402031	1.9087781	-0.6880633
C	0.6810715	1.1084998	0.1721533
C	0.6205578	1.4267734	1.5363107
C	1.3248641	2.5177104	2.0369099
H	2.6436596	4.1630485	1.5678161
H	2.7300849	3.6225570	-0.8610600
H	1.4774224	1.6918952	-1.7565658
H	0.0271554	0.8171207	2.2195460
H	1.2698911	2.7535041	3.1021811
C	-7.2676649	3.2346840	-0.3482162
C	-6.3117112	3.4032520	-1.3501982
C	-4.9753684	3.0886714	-1.1085426
C	-4.5818579	2.6055636	0.1458754
C	-5.5483362	2.4311589	1.1480929
C	-6.8827284	2.7485787	0.9007656
H	-8.3137146	3.4834295	-0.5412260

H	-6.6052044	3.7877171	-2.3297140
H	-4.2398363	3.2378743	-1.9014921
H	-5.2600717	2.0549962	2.1328233
H	-7.6243373	2.6147536	1.6916955
C	-2.3912095	2.8095642	4.9858701
C	-2.2109157	1.5308279	4.4605224
C	-2.3426996	1.3198338	3.0888808
C	-2.6495450	2.3860956	2.2349862
C	-2.8668079	3.6624126	2.7744634
C	-2.7261222	3.8720267	4.1436654
H	-2.2853593	2.9783509	6.0601499
H	-1.9706192	0.6927319	5.1183702
H	-2.2127366	0.3134328	2.6777590
H	-3.1654417	4.4924147	2.1299944
H	-2.8888492	4.8696441	4.5579022
C	-0.2796175	4.7953634	-2.1984403
C	-0.3722004	5.0784162	-0.8366564
C	-1.1334716	4.2680536	0.0043160
C	-1.8125713	3.1644341	-0.5196740
C	-1.6957875	2.8689716	-1.8858383
C	-0.9384034	3.6834288	-2.7228249
H	0.3202424	5.4343421	-2.8506876
H	0.1614160	5.9344801	-0.4176435
H	-1.1745379	4.4919226	1.0706005
H	-2.1947357	1.9899209	-2.3008916
H	-0.8562474	3.4414383	-3.7848827
Pd	-2.5945456	-0.2795831	-0.0737976
C	-3.1500099	-2.0287937	-0.4484858
C	-6.9736325	-1.4338050	1.0612465
C	-5.4841483	-1.7777827	0.9557018
C	-4.8462254	-0.8710886	-0.0806465
C	-5.4891406	-0.9457548	-1.4534470
C	-6.9794725	-0.6176031	-1.3268914
C	-7.6614832	-1.5130642	-0.2978460
H	-7.0825905	-0.4132707	1.4685409
H	-7.4557512	-2.1147228	1.7810774
H	-5.3814655	-2.8371141	0.6658823
H	-4.9914532	-1.6578241	1.9332102
H	-4.9269686	0.1618580	0.2823469
H	-5.0033909	-0.2418973	-2.1482084
H	-5.3738847	-1.9569193	-1.8805888
H	-7.0885318	0.4392662	-1.0278016
H	-7.4624814	-0.7189064	-2.3120960
H	-7.6395430	-2.5588430	-0.6540935
H	-8.7243932	-1.2391726	-0.2016565
O	-3.2879248	-3.1389293	-0.7079267

Cationic carbonylative insertion (dppp) TS 7
E (E_h) = -2202.412772

OC (E_h) = 0.003234333
dU_{298} (kJ*mol^{-1}) = 1661.7
dS_{298} (kJ*mol^{-1}*K^{-1}) = 0.98779
Imaginary frequencies = 1

75

P	-0.7166104	1.4576097	-0.8660508
P	-3.5602967	0.7465071	0.6297555
C	2.2210837	3.5117197	2.0310136
C	1.2826855	4.2876064	1.3527780
C	0.3946346	3.6921117	0.4575219
C	0.4425245	2.3101803	0.2410103
C	1.3906463	1.5336657	0.9229721
C	2.2778175	2.1337066	1.8113003
H	2.9120927	3.9812635	2.7350958
H	1.2368118	5.3659657	1.5208376
H	-0.3336060	4.3169208	-0.0623363
H	1.4297857	0.4522628	0.7647145
H	3.0122604	1.5221097	2.3401933
C	1.4848681	0.6920797	-4.8441818
C	0.5594570	-0.2361748	-4.3684218
C	-0.0866532	-0.0149331	-3.1527056
C	0.1855555	1.1425729	-2.4148131
C	1.1149297	2.0745656	-2.8962536
C	1.7634981	1.8450795	-4.1070781
H	1.9973008	0.5147332	-5.7927162
H	0.3435416	-1.1411521	-4.9407453
H	-0.8012718	-0.7504400	-2.7720049
H	1.3415474	2.9761489	-2.3206247
H	2.4935430	2.5690384	-4.4763594
C	-2.0015508	2.6918628	-1.3350821
C	-6.6444115	-2.1748647	2.4114330
C	-6.4251085	-2.1356259	1.0334591
C	-5.5332869	-1.2118190	0.4939625
C	-4.8652784	-0.3057391	1.3314443
C	-5.0830420	-0.3526639	2.7140856
C	-5.9720747	-1.2847844	3.2484072
H	-7.3397567	-2.9040369	2.8335509
H	-6.9464019	-2.8330646	0.3737904
H	-5.3545629	-1.2085238	-0.5851168
H	-4.5581210	0.3393803	3.3768815
H	-6.1401790	-1.3127051	4.3274769
C	-2.5363332	3.9419205	3.7953099
C	-1.6518531	2.8973409	3.5333672
C	-1.9794699	1.9338000	2.5812024
C	-3.1900457	2.0133846	1.8809211
C	-4.0816840	3.0591217	2.1587030
C	-3.7525484	4.0188063	3.1123188
H	-2.2797017	4.7019155	4.5371705

H -0.6991760 2.8328533 4.0632902
H -1.2811914 1.1181856 2.3735251
H -5.0388337 3.1288800 1.6355225
H -4.4493506 4.8331183 3.3238496
Pd -1.5984218 -0.4571051 0.0777935
C -0.3814123 -1.8834451 -0.0195820
C -2.4664870 -3.8657654 2.9612095
C -1.4825502 -2.9236036 2.2637423
C -2.0238248 -2.5606671 0.8910204
C -2.3076228 -3.7641590 0.0050362
C -3.2835767 -4.6988736 0.7232026
C -2.7674089 -5.0908356 2.1039382
H -3.4052795 -3.3188252 3.1631243
H -2.0593810 -4.1670759 3.9398417
H -0.5029333 -3.4232203 2.1662560
H -1.3237566 -2.0184814 2.8712182
H -2.9817824 -2.0350576 1.0340126
H -2.7238105 -3.4431711 -0.9631035
H -1.3724412 -4.3097913 -0.2082981
H -4.2590455 -4.1914636 0.8275596
H -3.4567965 -5.5948134 0.1054783
H -1.8448739 -5.6882686 1.9899313
H -3.4980454 -5.7402650 2.6122843
C -4.2855562 1.6205985 -0.8199148
C -3.2599303 2.0129385 -1.8851031
H -4.8246278 2.5053221 -0.4468865
H -5.0428121 0.9514668 -1.2542735
H -3.7540915 2.6967414 -2.5910611
H -2.9815351 1.1239449 -2.4770360
H -1.5518313 3.3635620 -2.0816242
H -2.2407353 3.3002479 -0.4484574
O 0.5115864 -2.5985244 -0.1644829

Associative morphline binding TS 8
E (E_h) = -3208.929823
OC (E_h) = -0.004752362
dU_{298} (kJ*mol^{-1}) = 2339.8
dS_{298} (kJ*mol^{-1}*K^{-1}) = 1.31612
Imaginary frequencies = 1

106
P 0.0303127 -0.9600995 -1.7428903
P -1.1002185 3.5386685 -1.5030286
C -3.1950454 -3.9918702 -0.3453041
C -2.2395842 -3.5496425 0.5679435
C -1.2467491 -2.6519193 0.1718559
C -1.2048907 -2.1820939 -1.1440304
C -2.1718862 -2.6291498 -2.0570499
C -3.1557536 -3.5309594 -1.6627969

H	-3.9709905	-4.6947475	-0.0323962
H	-2.2612374	-3.9065153	1.6006586
H	-0.5001363	-2.3219982	0.8961994
H	-2.1447368	-2.2788998	-3.0921173
H	-3.8992027	-3.8714011	-2.3876495
C	1.9315361	-3.1923256	-5.3321003
C	1.9073231	-1.7989078	-5.2974296
C	1.3106866	-1.1347874	-4.2264167
C	0.7320696	-1.8580963	-3.1805682
C	0.7649799	-3.2599187	-3.2155108
C	1.3597322	-3.9218358	-4.2876930
H	2.3951693	-3.7132557	-6.1738477
H	2.3496729	-1.2200063	-6.1121225
H	1.2926299	-0.0441593	-4.1839856
H	0.3261560	-3.8405580	-2.4011272
H	1.3782021	-5.0144604	-4.3059518
C	3.4527230	-1.0485840	1.3856318
C	3.6433019	-1.6429109	0.1390311
C	2.6174395	-1.6425137	-0.8058677
C	1.3878941	-1.0436785	-0.5114406
C	1.2044346	-0.4347976	0.7386002
C	2.2293834	-0.4459864	1.6823013
H	4.2588116	-1.0496653	2.1237533
H	4.6011841	-2.1086321	-0.1063708
H	2.7846754	-2.1009553	-1.7818434
H	0.2567410	0.0608619	0.9629385
H	2.0714500	0.0270319	2.6551474
C	-5.4666616	4.4639799	-0.2038362
C	-5.1580141	4.2610071	-1.5488599
C	-3.8472642	4.0185883	-1.9627235
C	-2.8146138	4.0079937	-1.0109193
C	-3.1286773	4.1959593	0.3442832
C	-4.4452301	4.4203253	0.7443260
H	-6.4997683	4.6406342	0.1073796
H	-5.9548675	4.2636046	-2.2981579
H	-3.6833906	3.7464519	-3.0267448
H	-2.3464437	4.1476818	1.1028969
H	-4.6697240	4.5586779	1.8051992
C	1.3518935	4.8219848	2.2109025
C	1.4207455	3.5123386	1.7393529
C	0.6872618	3.1331664	0.6149368
C	-0.1249379	4.0590408	-0.0427768
C	-0.1918537	5.3760109	0.4368490
C	0.5440367	5.7556082	1.5563007
H	1.9297402	5.1202810	3.0896289
H	2.0557043	2.7781522	2.2417738
H	0.7480715	2.1158662	0.2271810
H	-0.8248175	6.1104071	-0.0681953
H	0.4876308	6.7846658	1.9199796

C	0.1764546	6.5297210	-4.8154748
C	0.9421075	6.4019785	-3.6564713
C	0.5574063	5.5112182	-2.6561256
C	-0.6081616	4.7450373	-2.7985557
C	-1.3582622	4.8476272	-3.9773542
C	-0.9650636	5.7443229	-4.9718024
H	0.4786175	7.2291538	-5.5997600
H	1.8526615	6.9933092	-3.5293122
H	1.1790164	5.4061818	-1.7666615
H	-2.2183234	4.1768611	-4.1632693
H	-1.5559244	5.8144075	-5.8898918
Pd	-0.7078009	1.2669098	-1.9875455
C	-2.0362934	0.9126464	-0.5321398
I	2.0920107	2.0877387	-2.6266183
O	-1.6811476	0.8460323	0.6213792
C	-5.7054040	-0.2779658	-0.2019614
C	-4.2010219	-0.2428908	0.0410108
C	-3.5191164	0.7813642	-0.8704672
C	-3.8489046	0.5535788	-2.3348350
C	-5.3538619	0.5060688	-2.5603562
C	-6.0320000	-0.5291219	-1.6709336
H	-6.1439078	0.6901616	0.1032694
H	-6.1702753	-1.0487116	0.4360806
H	-3.7740327	-1.2399515	-0.1541753
H	-3.9786565	-0.0027794	1.0911976
H	-3.9097778	1.7774988	-0.5907025
H	-3.4249846	1.3414882	-2.9729981
H	-3.4040374	-0.4042363	-2.6486490
H	-5.7724040	1.5051948	-2.3437252
H	-5.5574177	0.3146121	-3.6261011
H	-5.6762533	-1.5383272	-1.9495628
H	-7.1240542	-0.5260034	-1.8277827
C	-2.3535189	0.3481642	-6.5532811
C	-1.7441532	0.0656110	-5.1897053
N	-1.2327337	1.2827936	-4.5740273
C	-0.2979757	1.9230157	-5.4875383
C	-0.9163386	2.1610180	-6.8558111
O	-1.4152309	0.9632323	-7.4149006
H	-3.2266012	1.0127058	-6.4010679
H	-2.6842490	-0.5788518	-7.0491239
H	-0.9292152	-0.6700192	-5.3005391
H	-2.5090100	-0.3800493	-4.5364758
H	0.0484218	2.8721768	-5.0537697
H	0.5970751	1.2882134	-5.6105876
H	-1.7373711	2.8996945	-6.7578888
H	-0.1744552	2.5626728	-7.5646973
H	-2.0765531	1.9263137	-4.4894083
O	-3.4966382	2.8045188	-4.5978361
H	-3.4630380	3.1789080	-5.4860452

Outer sphere aminocarbonylation TS 9
E (E_h) = -3133.043097
OC (E_h) = -0.000439452
dU_{298} (kJ*mol^{-1}) = 2305.79
dS_{298} (kJ*mol^{-1}*K^{-1}) = 1.27449
Imaginary frequencies = 1

104

P	2.0258331	-0.8489288	-0.1287505
P	-0.3662965	2.0469965	0.3254926
C	2.8796775	-4.7531825	-2.5161101
C	3.7837810	-3.6920501	-2.5915826
C	3.5386122	-2.5100290	-1.8961968
C	2.3791449	-2.3689207	-1.1194857
C	1.4863159	-3.4438899	-1.0371522
C	1.7342367	-4.6278248	-1.7321265
H	3.0723774	-5.6781458	-3.0654549
H	4.6898543	-3.7849722	-3.1956983
H	4.2571303	-1.6908832	-1.9678496
H	0.5916352	-3.3561886	-0.4148605
H	1.0238604	-5.4550872	-1.6585071
C	4.1958512	2.4470460	-2.5801500
C	4.3223822	2.4222940	-1.1916486
C	3.7011810	1.4228424	-0.4424295
C	2.9425291	0.4284775	-1.0737713
C	2.8001335	0.4781127	-2.4699603
C	3.4267608	1.4718373	-3.2171232
H	4.6885325	3.2281555	-3.1641389
H	4.9076527	3.1893394	-0.6790059
H	3.8088690	1.4246622	0.6434563
H	2.2048867	-0.2787105	-2.9840433
H	3.3089363	1.4856421	-4.3032846
C	4.6297082	-1.7591131	3.6045373
C	5.2550908	-1.4169854	2.4037715
C	4.4929174	-1.1356660	1.2721542
C	3.0936289	-1.1772566	1.3336285
C	2.4741764	-1.5350788	2.5362376
C	3.2384054	-1.8263674	3.6660854
H	5.2299757	-1.9792682	4.4909374
H	6.3456193	-1.3727790	2.3466872
H	4.9958526	-0.8762597	0.3377581
H	1.3830144	-1.5940084	2.5833115
H	2.7411938	-2.1036315	4.5989206
C	-0.4080805	4.6269804	-3.5103581
C	0.7649633	4.0153299	-3.0678865
C	0.7690398	3.2882944	-1.8806110
C	-0.3988383	3.1648460	-1.1158880
C	-1.5753124	3.7759542	-1.5683784

C	-1.5755761	4.5049567	-2.7563335
H	-0.4135712	5.1958435	-4.4435275
H	1.6854815	4.0958484	-3.6509004
H	1.6895098	2.8035964	-1.5540719
H	-2.5017460	3.6561227	-1.0081070
H	-2.5007781	4.9755325	-3.0983083
C	-3.4135156	3.5336722	3.4783738
C	-3.0649680	2.1857227	3.3949502
C	-2.1814828	1.7548961	2.4068723
C	-1.6421436	2.6628538	1.4913246
C	-1.9695287	4.0199759	1.5992302
C	-2.8588935	4.4497832	2.5829967
H	-4.1106574	3.8738651	4.2481989
H	-3.4826864	1.4644426	4.1019318
H	-1.8906751	0.7020779	2.3392739
H	-1.5255006	4.7514153	0.9203399
H	-3.1151103	5.5098366	2.6541317
C	3.3242424	3.1853636	2.8892502
C	2.6466757	1.9800398	3.0761158
C	1.5485626	1.6710363	2.2762945
C	1.1220394	2.5532134	1.2767607
C	1.7895117	3.7719588	1.1112842
C	2.8876356	4.0821446	1.9123622
H	4.1920871	3.4281185	3.5073393
H	2.9810860	1.2673544	3.8337585
H	1.0174441	0.7264533	2.4189065
H	1.4615020	4.4811866	0.3487975
H	3.4089228	5.0319166	1.7689556
Pd	-0.5060200	-0.2366128	0.0148440
C	-2.3113422	0.1383873	-1.0499170
I	-1.1897424	-2.4931992	1.5585844
O	-2.8937506	1.2072553	-0.9860395
C	-1.0753013	-2.3799121	-3.8201541
C	-1.3389759	-1.8740589	-2.4080192
C	-1.9159850	-0.4613081	-2.4206650
C	-0.9778821	0.4921784	-3.1665604
C	-0.6776001	-0.0101536	-4.5740072
C	-0.1440233	-1.4390861	-4.5747104
H	-2.0351735	-2.4633995	-4.3627183
H	-0.6465020	-3.3940947	-3.7780914
H	-0.3835744	-1.8680540	-1.8546724
H	-1.9896030	-2.5764851	-1.8650142
H	-2.8644125	-0.4743855	-2.9906181
H	-1.4204776	1.4979332	-3.1981209
H	-0.0381417	0.5884178	-2.5969806
H	-1.6025609	0.0283870	-5.1779644
H	0.0394542	0.6706696	-5.0619445
H	0.8484580	-1.4654808	-4.0892015
H	0.0094105	-1.7923963	-5.6072261

C	-5.7349882	-2.2470701	-0.7800578
C	-4.5867235	-1.4596206	-1.3889424
N	-3.5692487	-1.1303554	-0.3888981
C	-4.1760108	-0.5244319	0.7926827
C	-5.3432073	-1.3417514	1.3108422
O	-6.3047334	-1.5680685	0.3081830
H	-5.3727157	-3.2460128	-0.4622987
H	-6.5232921	-2.3961495	-1.5321492
H	-4.9612003	-0.5089164	-1.8014300
H	-4.1332042	-2.0387171	-2.2041722
H	-3.3996370	-0.4436505	1.5616368
H	-4.4901048	0.4913866	0.5121523
H	-4.9745345	-2.3082943	1.7104216
H	-5.8399601	-0.8039044	2.1311753
H	-3.0285398	-1.9551533	-0.0982706

NMR spectra

*cyclohexyl(morpholino)methanone (**3**)*

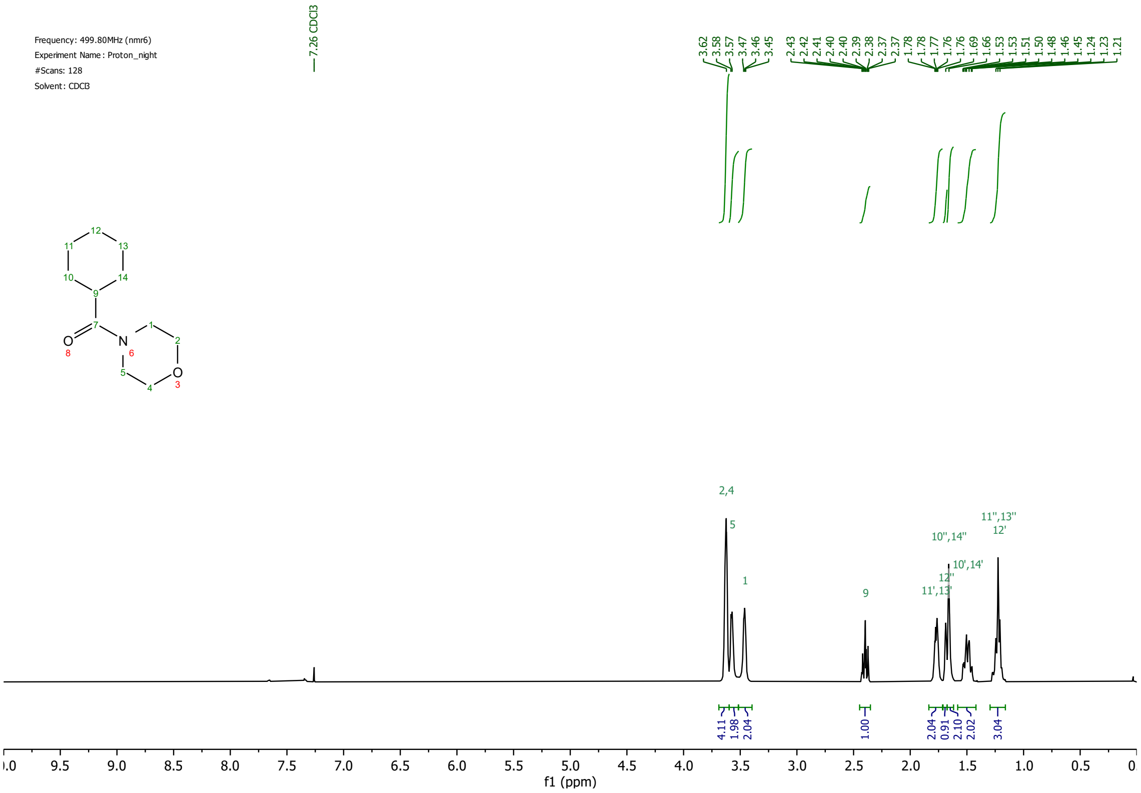

Figure S13: 1H NMR of amide **3**.

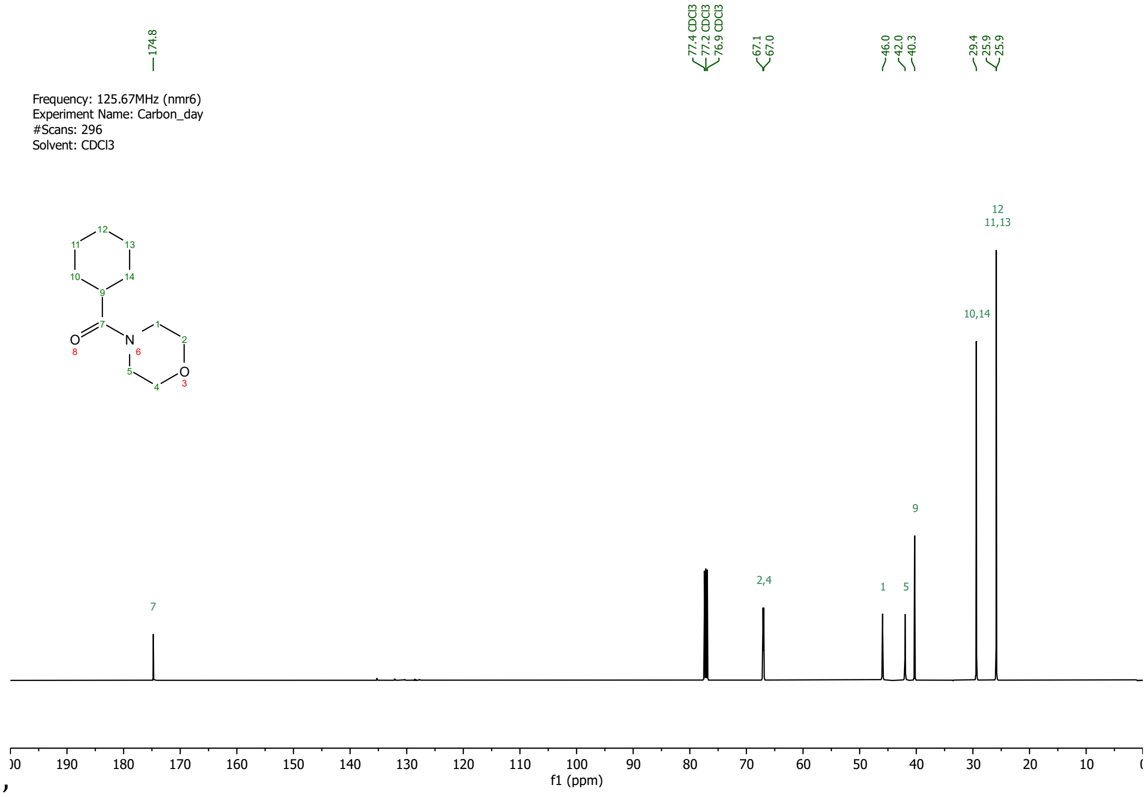

,

Figure S14: 13C{1H} NMR of **3**

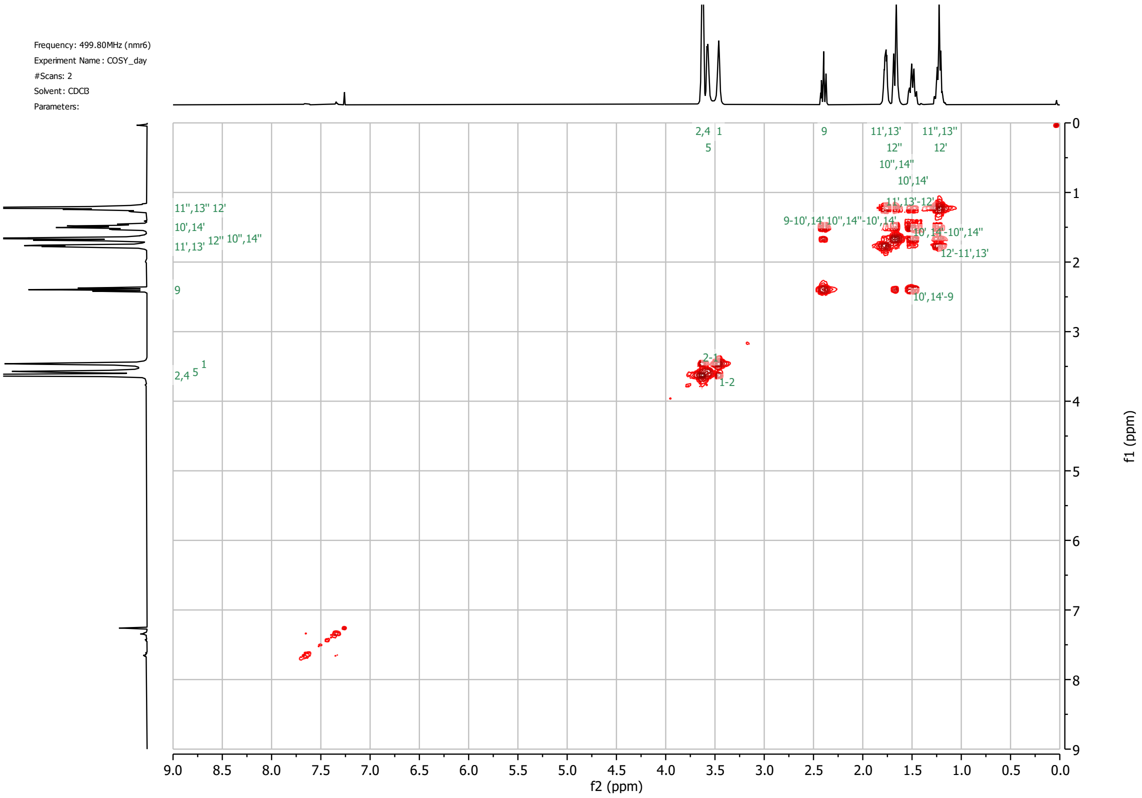

Figure S15: COSY of amide **3**

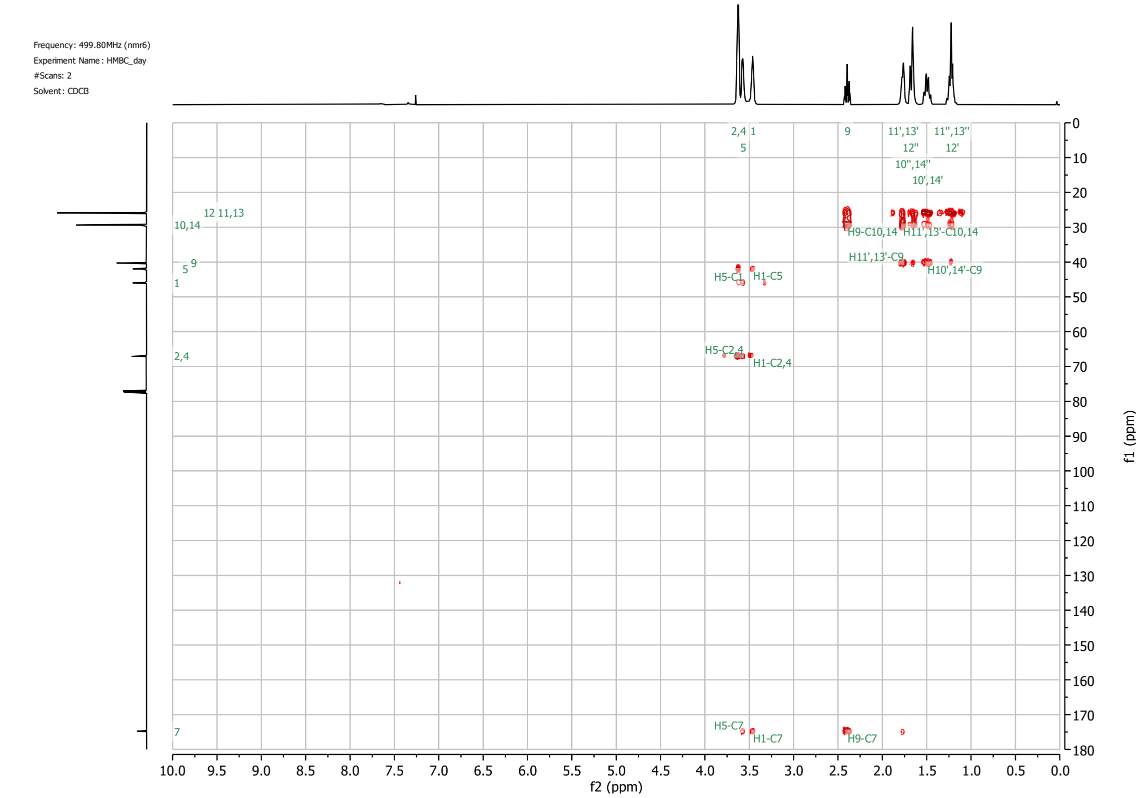

Figure S16: HMBC of amide **3**

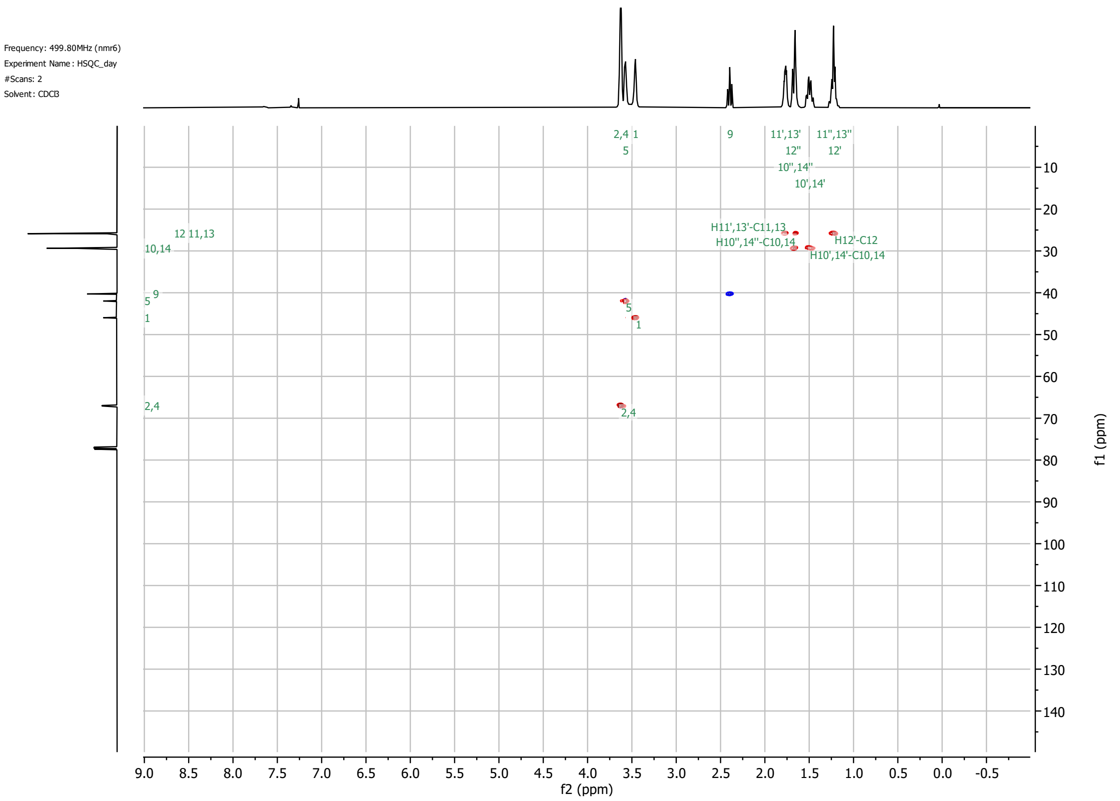

Figure S17: HSQC of amide **3**

cis-(4-iodocyclohexyl)benzene (***cis-20***)

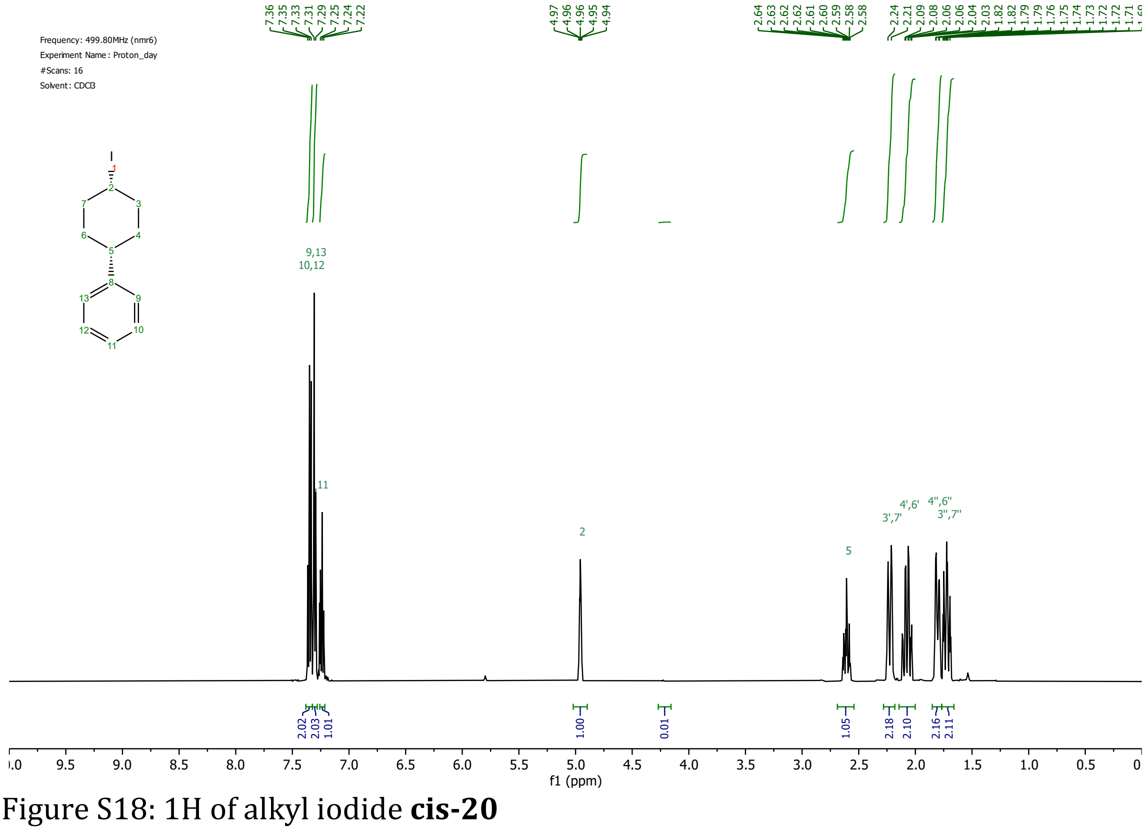

Figure S18: 1H of alkyl iodide **cis-20**

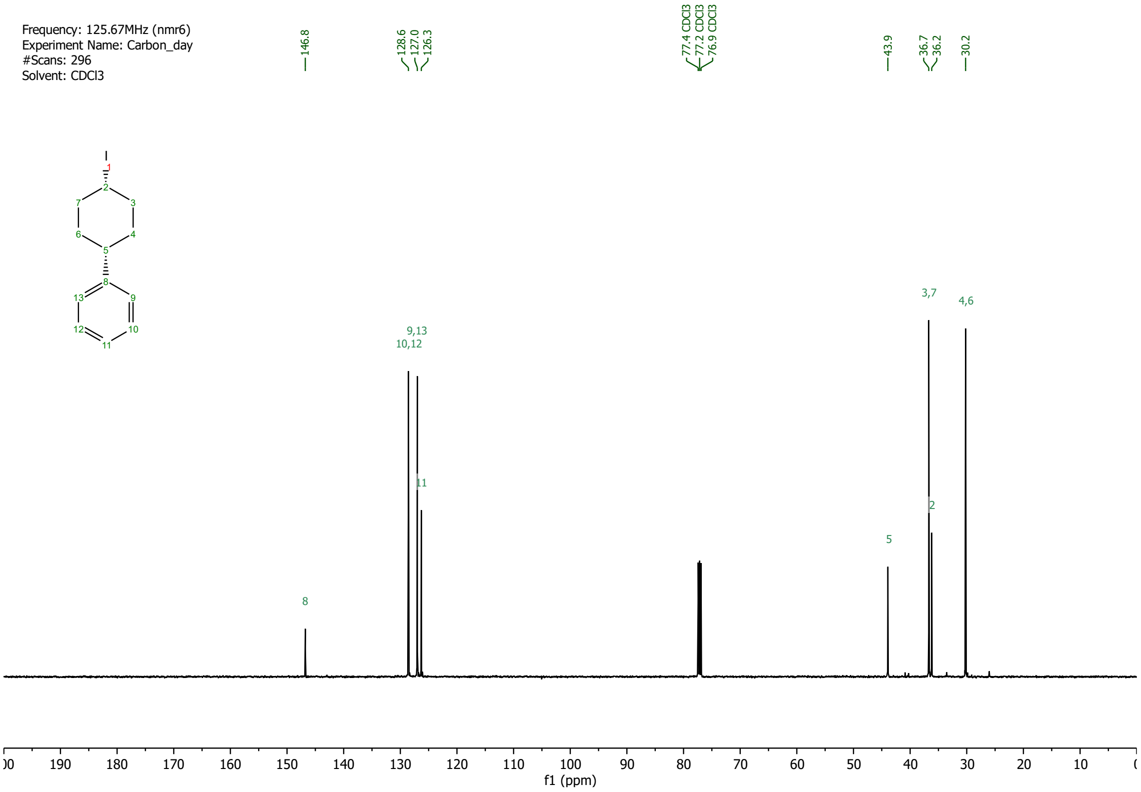

Figure S19: 13C{1H} of alkyl iodide **cis-20**

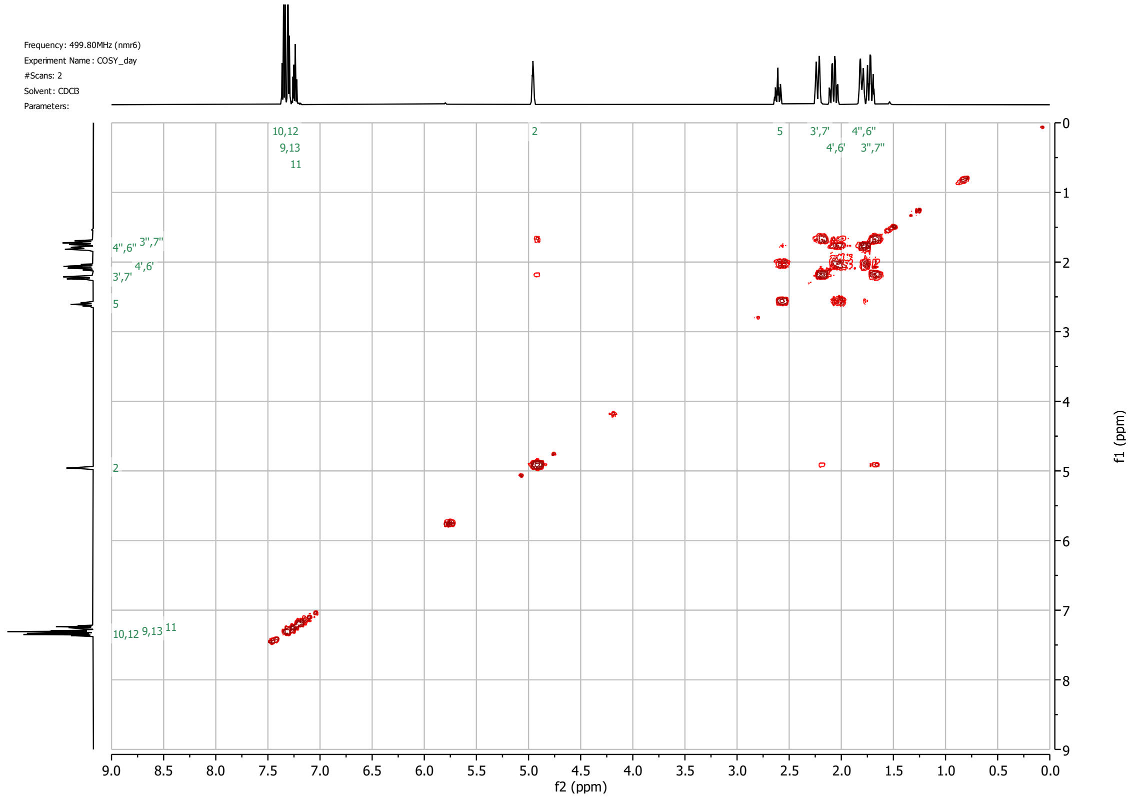

Figure S20: COSY of alkyl iodide **cis-20**

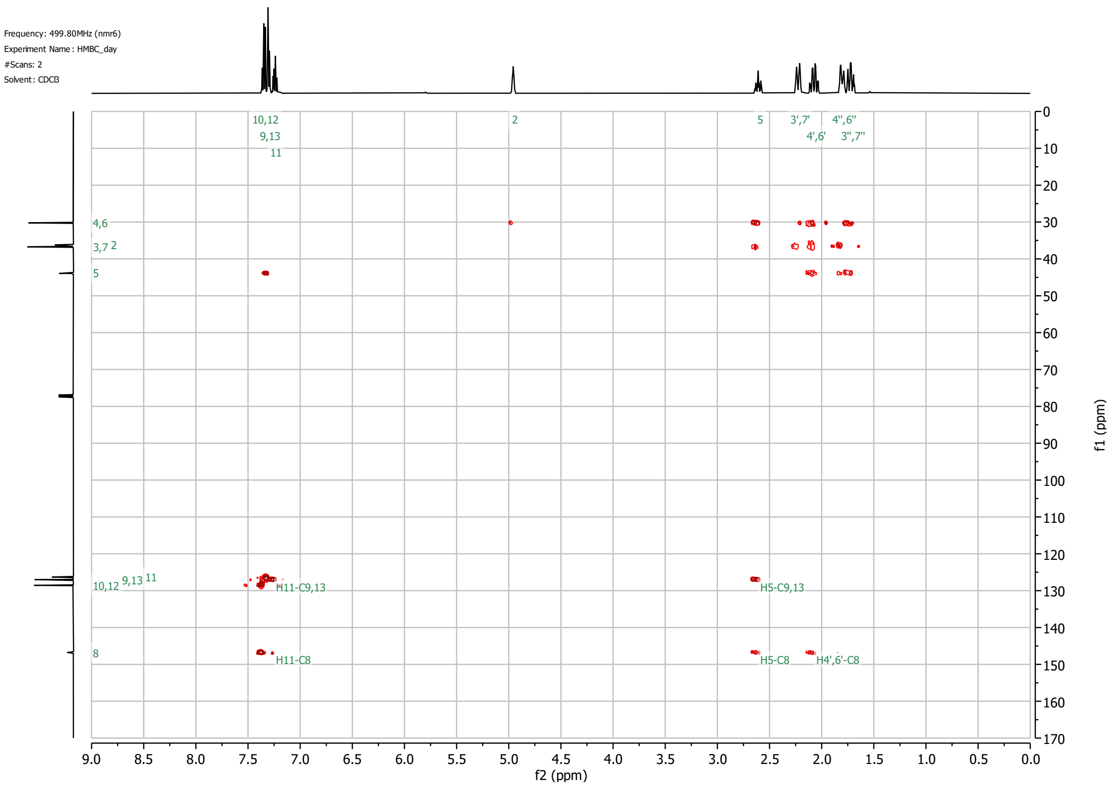

Figure S21: HMBC of alkyl iodide **cis-20**

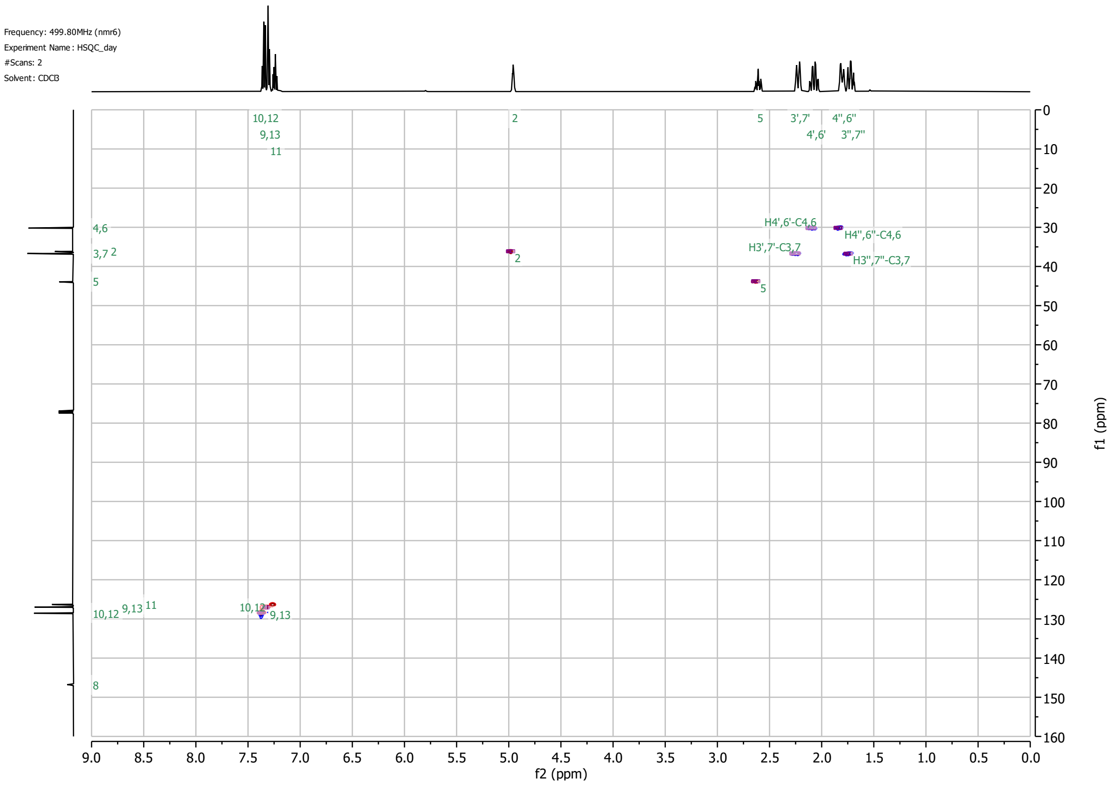

Figure S22: HSQC of alkyl iodide **cis-20**

*trans-(4-iodocyclohexyl)benzene (**trans-20**)*

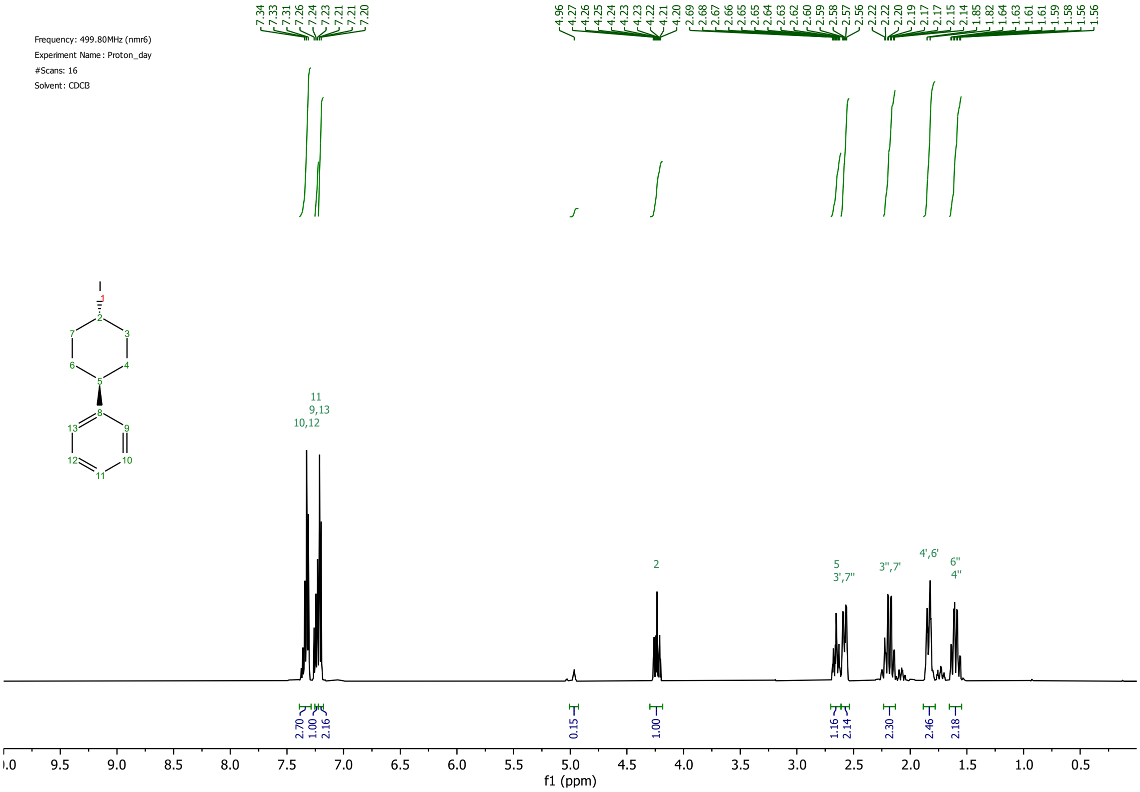

Figure S23: 1H of alkyl iodide **trans-20**

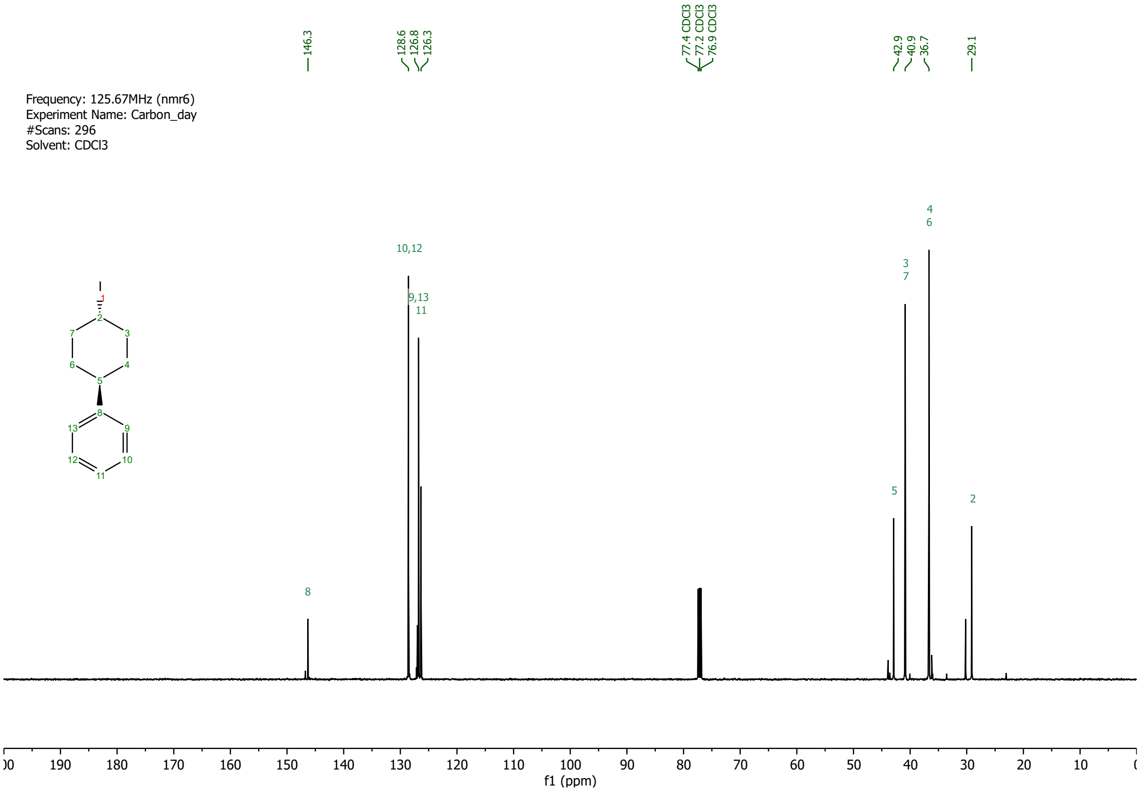

Figure S24: 13C{1H} of alkyl iodide **trans-20**

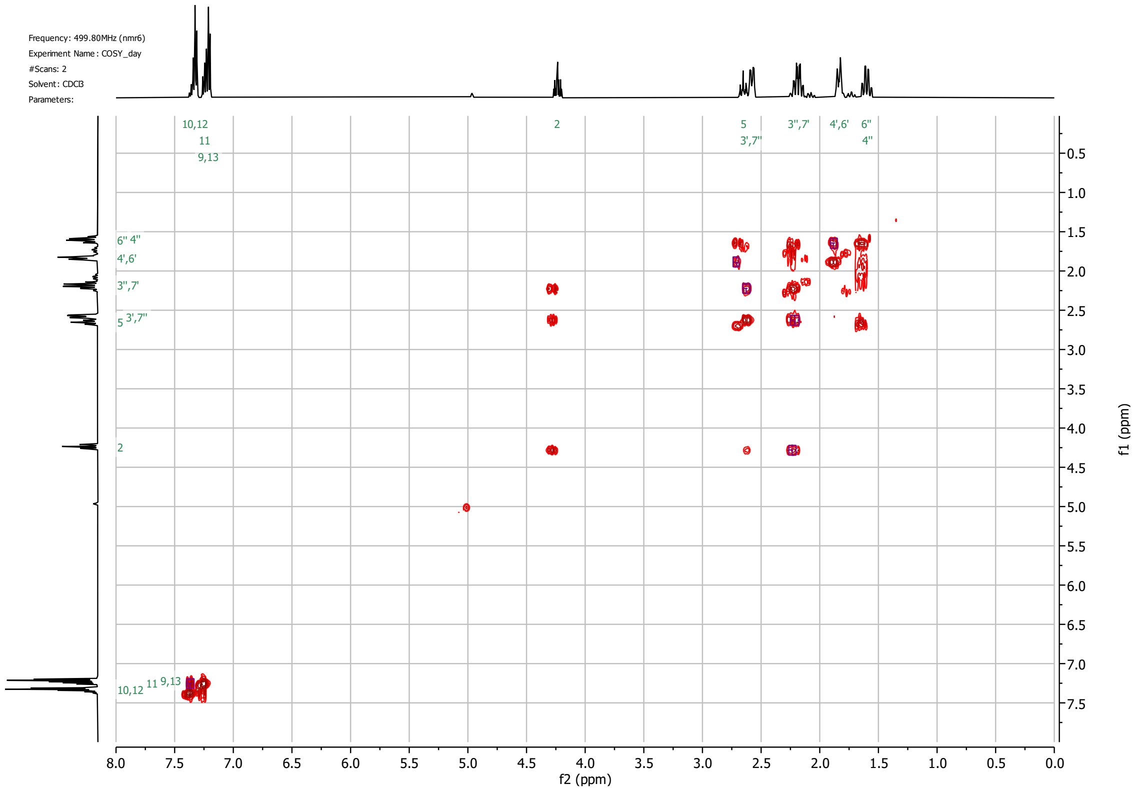

Figure S25: COSY of alkyl iodide **trans-20**

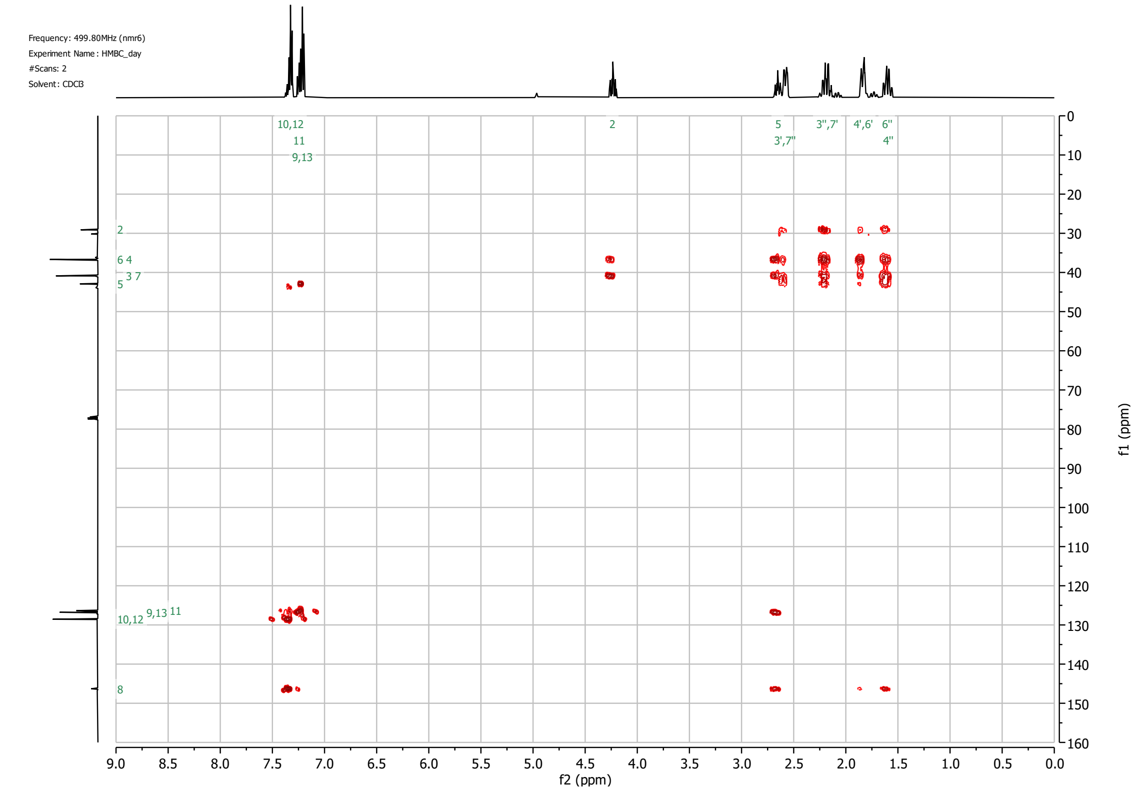

Figure S26: HMBC of alkyl iodide **trans-20**

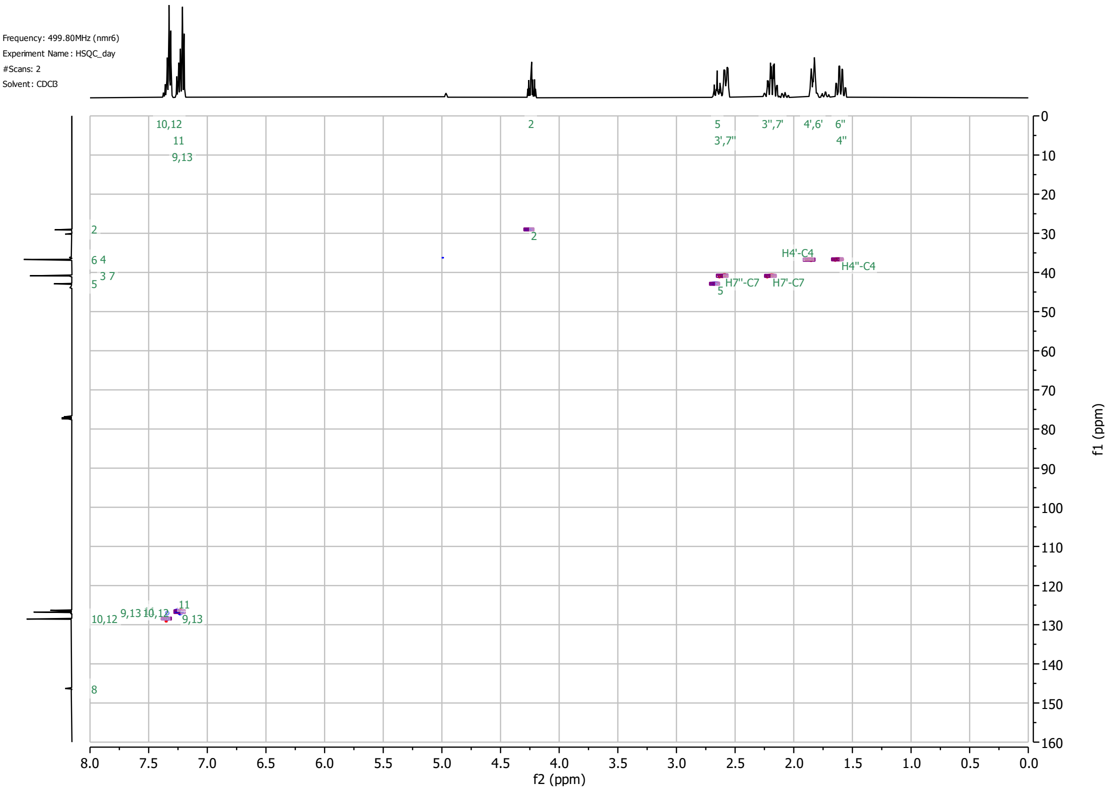

Figure S27: HSQC of alkyl iodide **trans-20**

*Morpholino-cis-(4-phenylcyclohexyl)methanone (**cis-21**)*

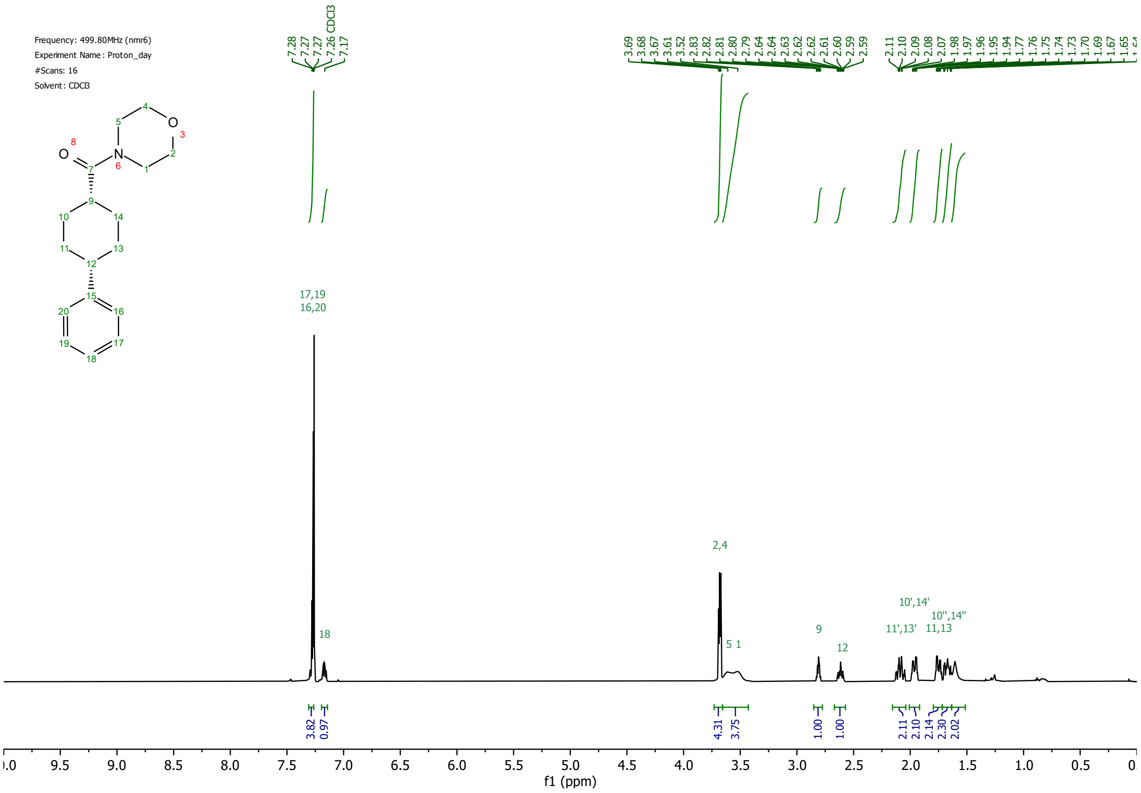

Figure S28: 1H of amide **cis-21**

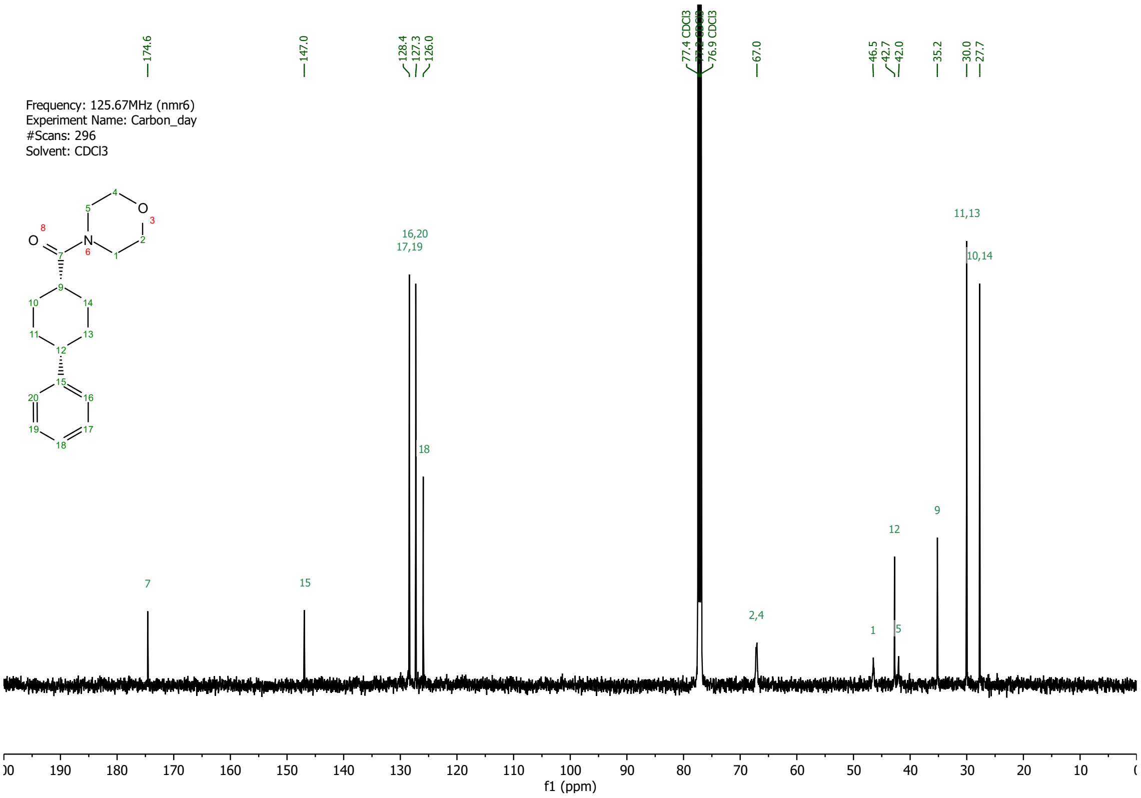

Figure S29: 13C{1H} of amide **cis-21**

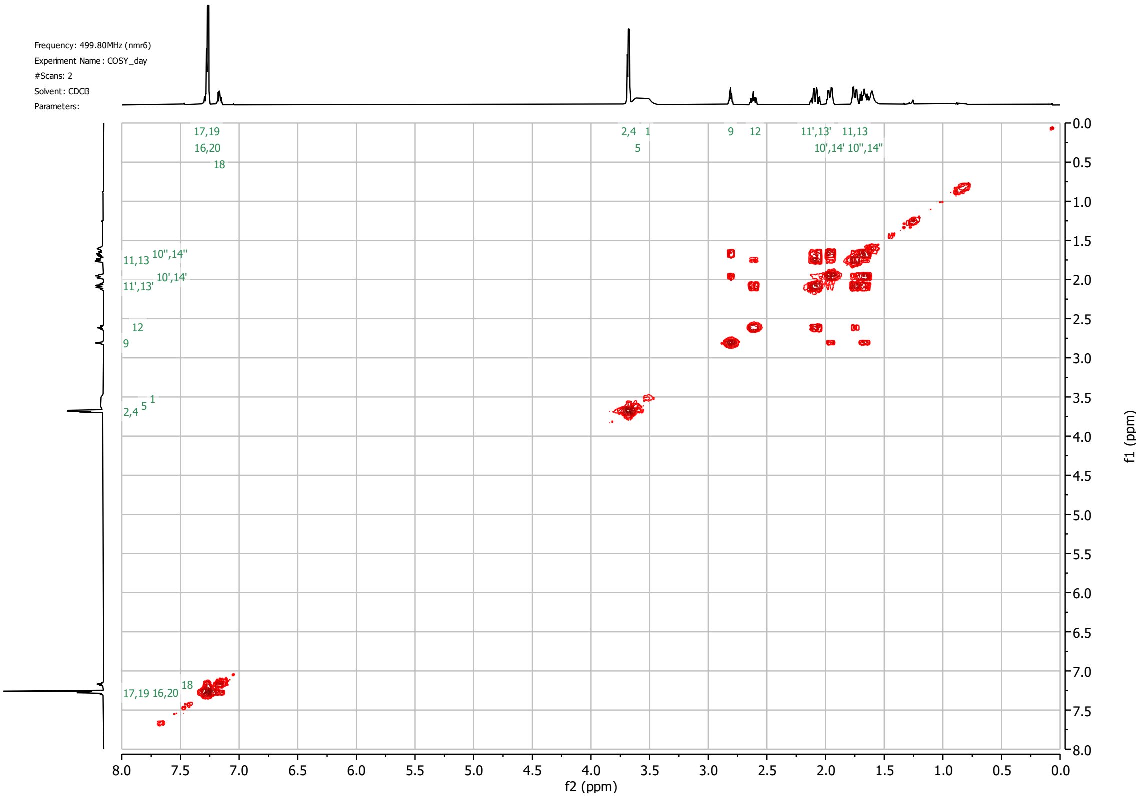

Figure S30: COSY of amide **cis-21**

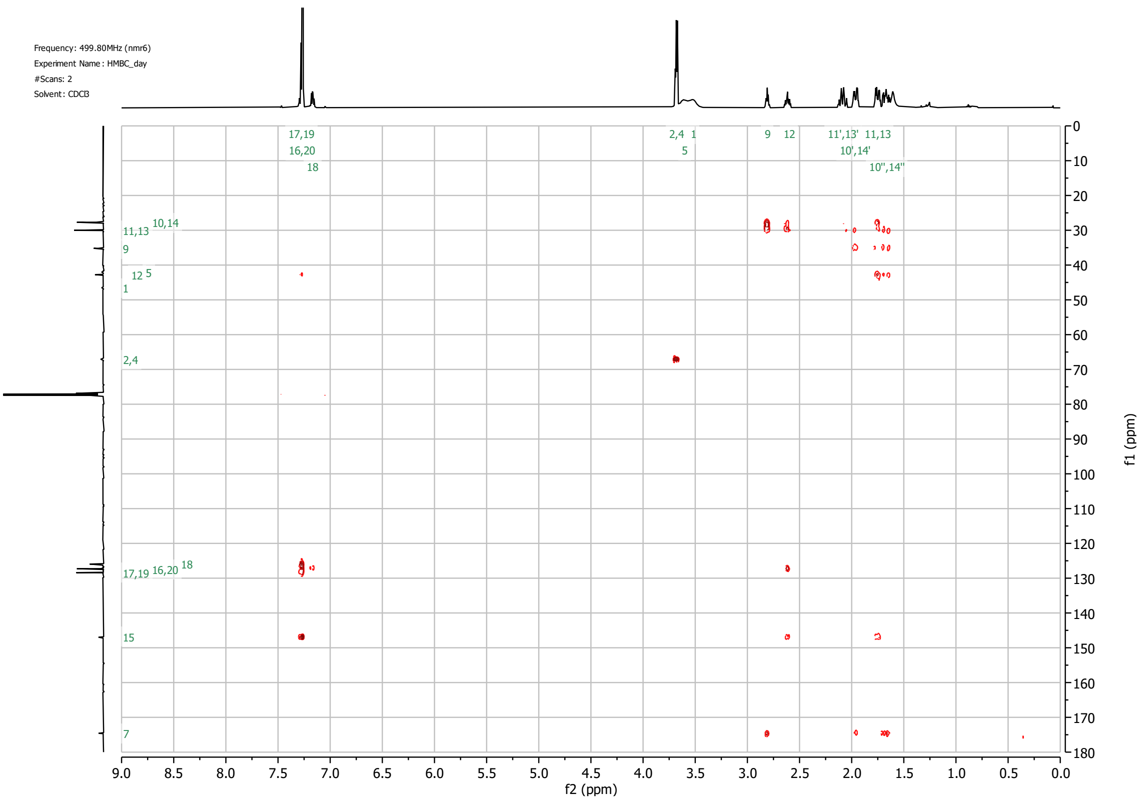

Figure S31: HMBC of amide **cis-21**

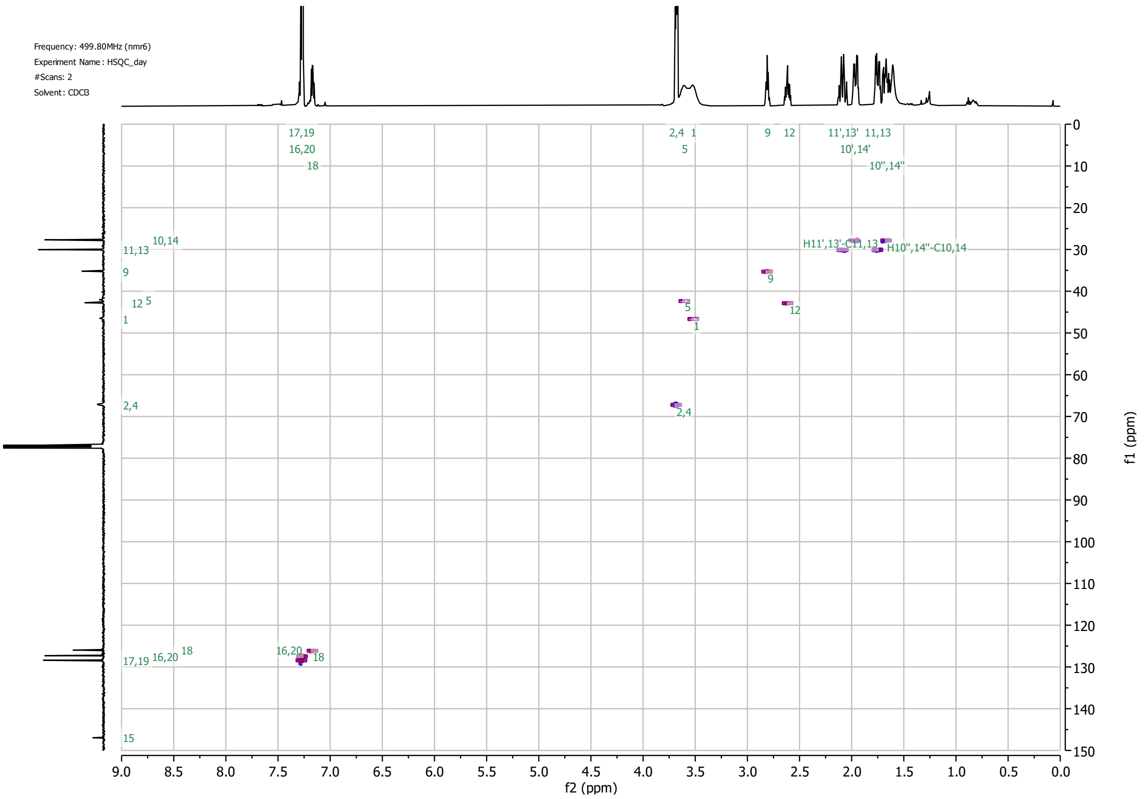

Figure S32: HSQC of amide **cis-21**

Morpholino-trans-(4-phenylcyclohexyl)methanone (***trans-21***)

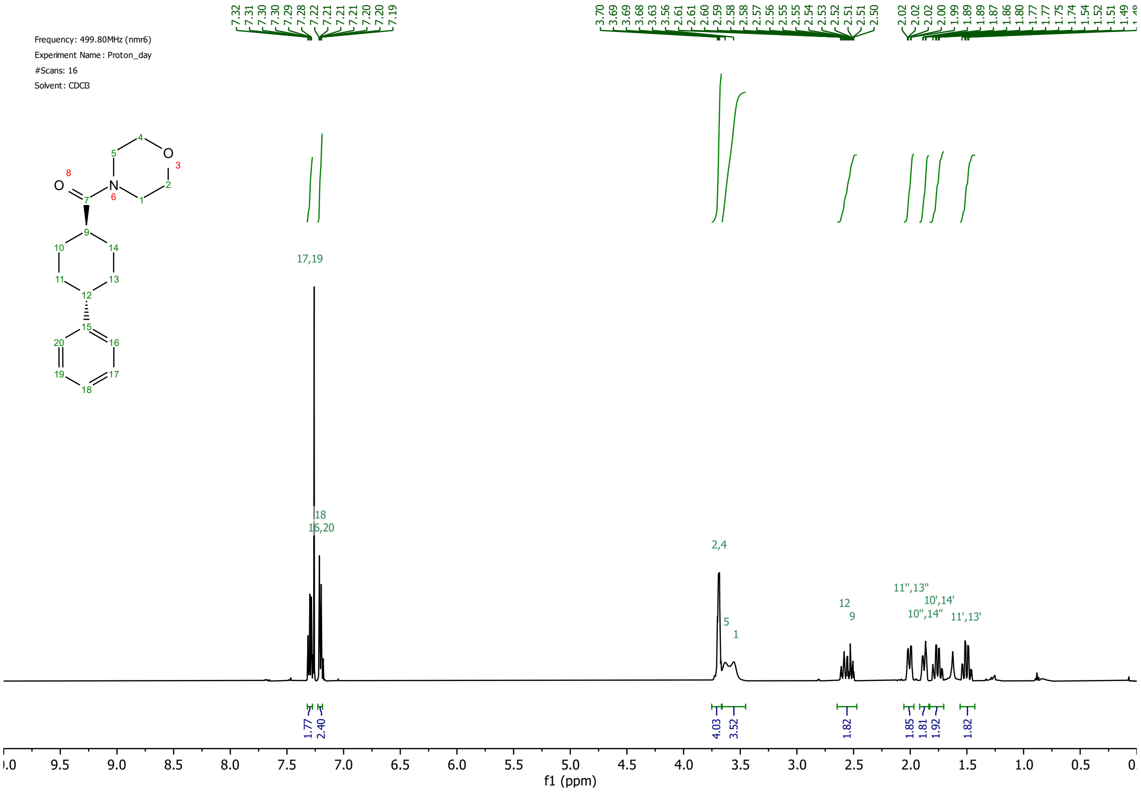

Figure S33: 1H of amide **trans-21**

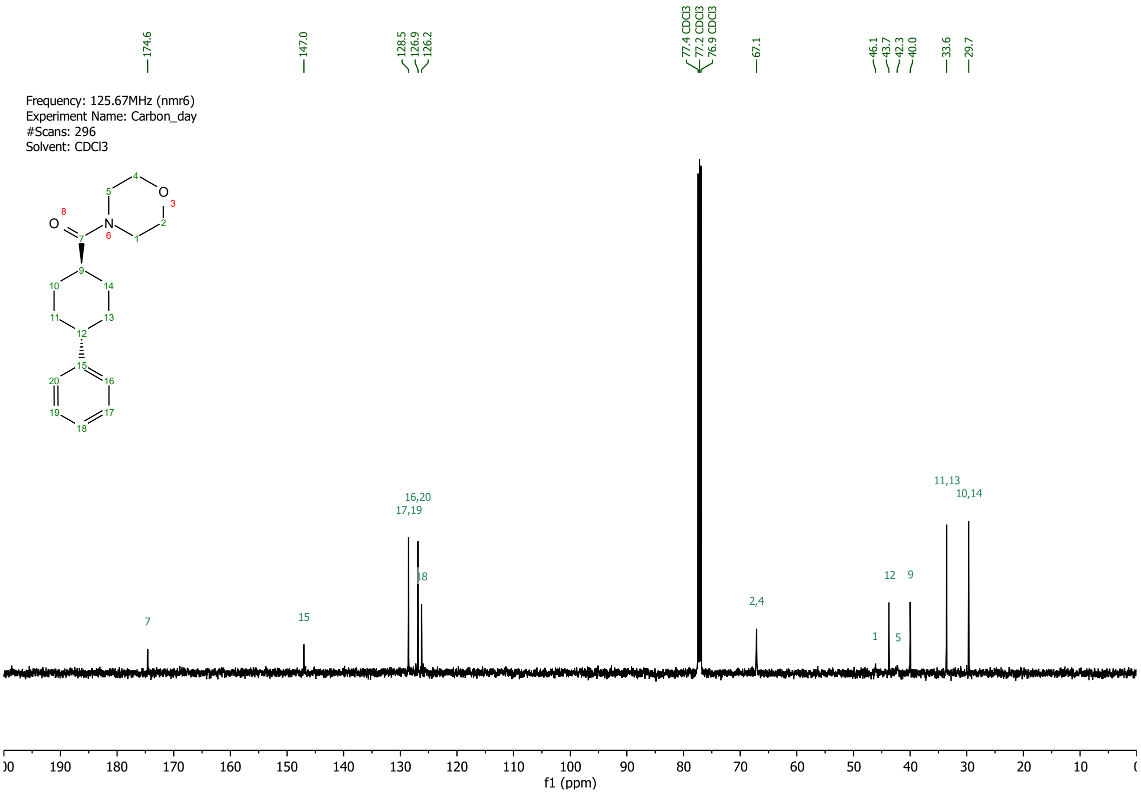

Figure S34: 13C{1H} of amide **trans-21**

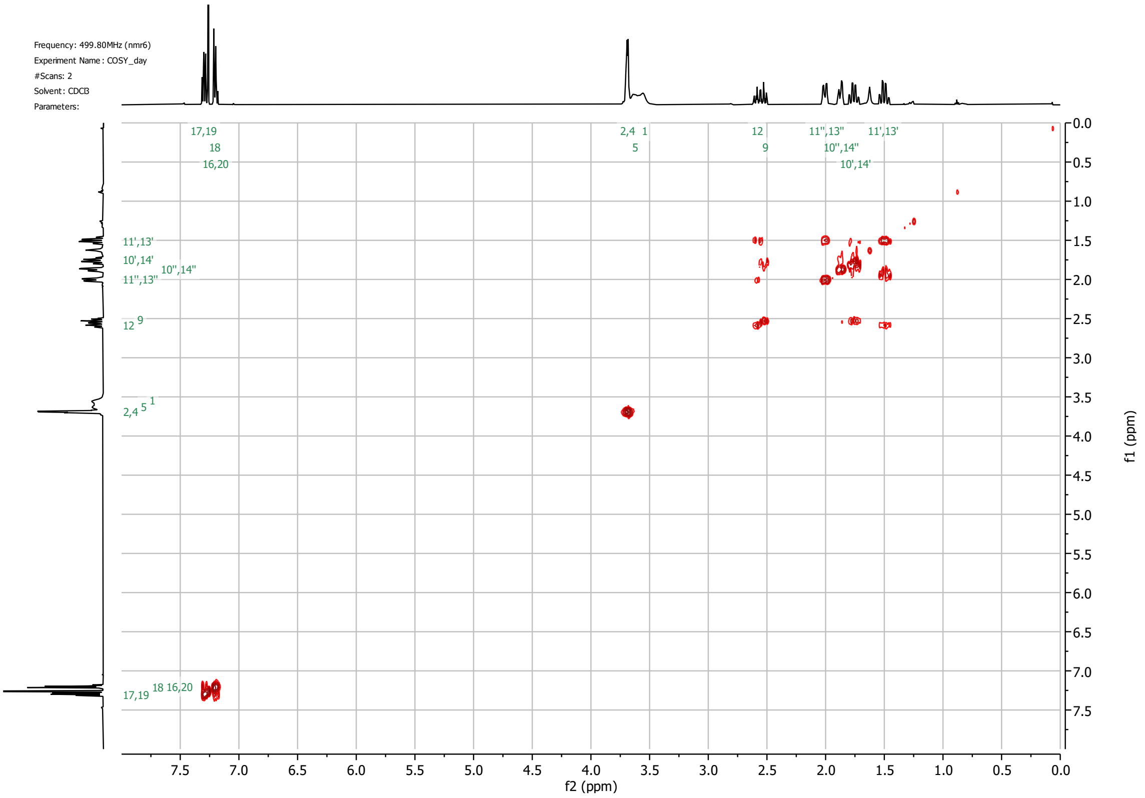

Figure S35: COSY of amide **trans-21**

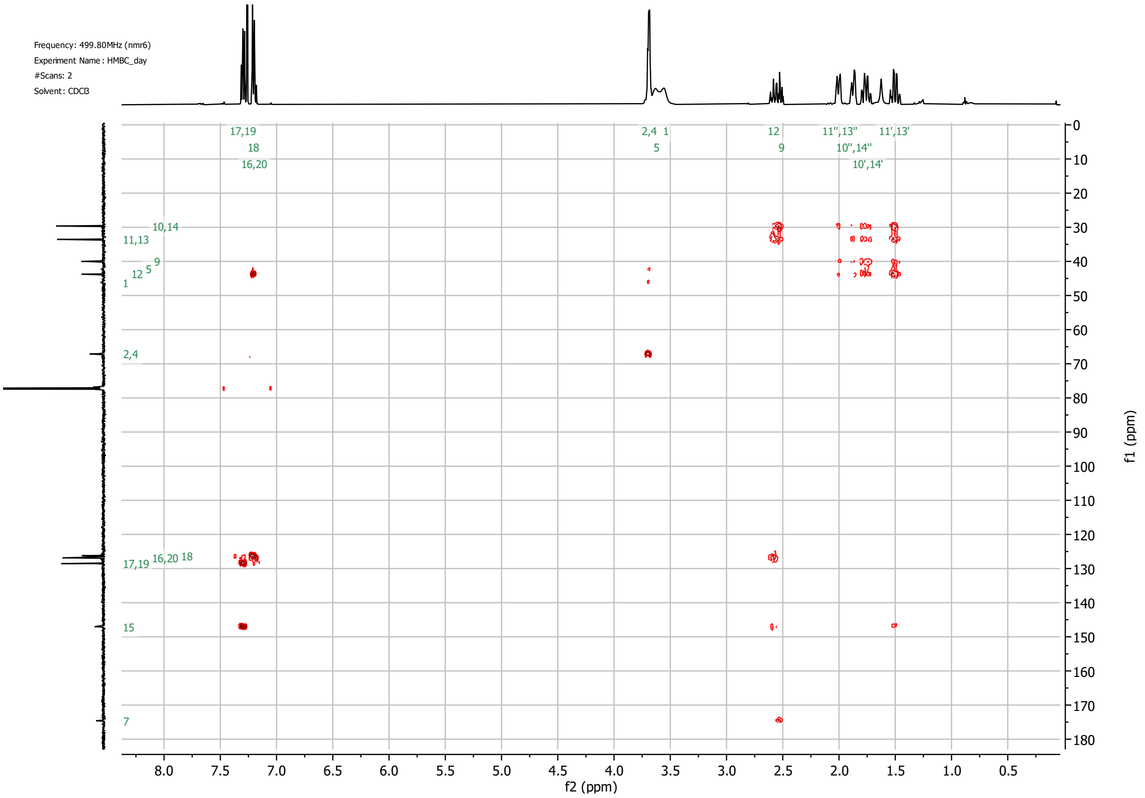

Figure S36: HMBC of amide **trans-21**

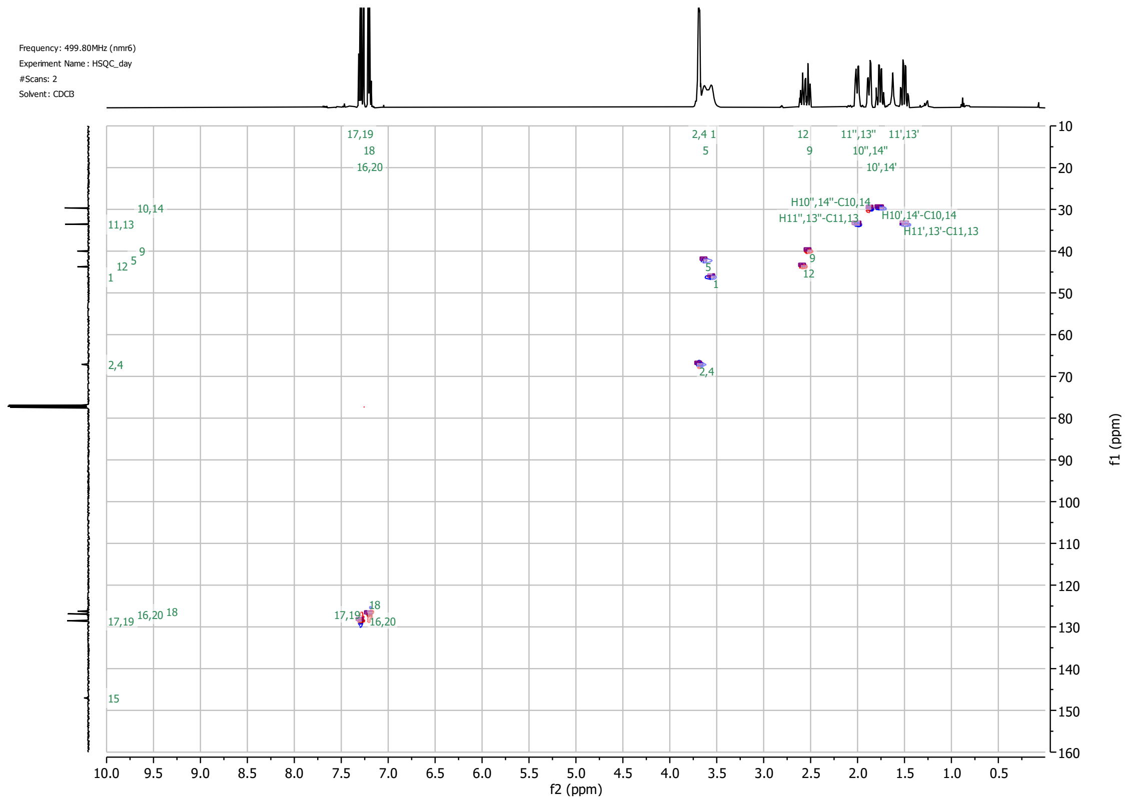

Figure S37: HSQC of amide **trans-21**

*Carbonyl dimorpholine (**43**)*

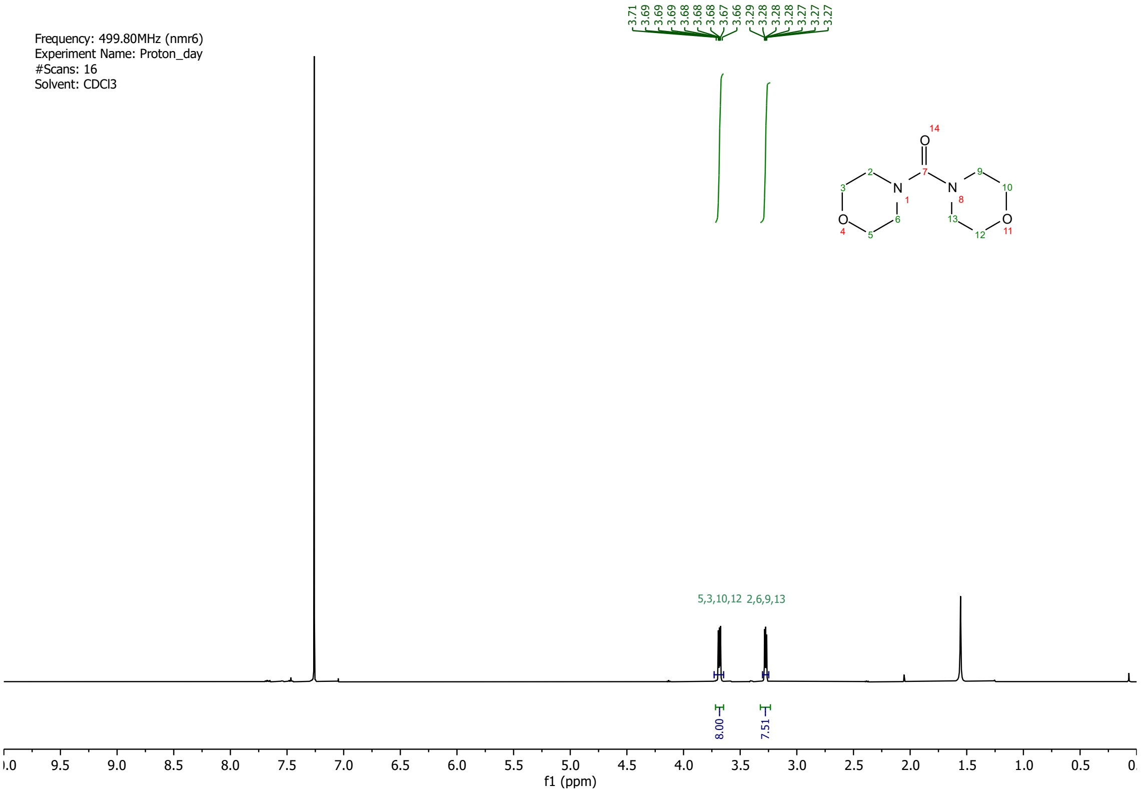

Figure S38: 1H of urea **43**

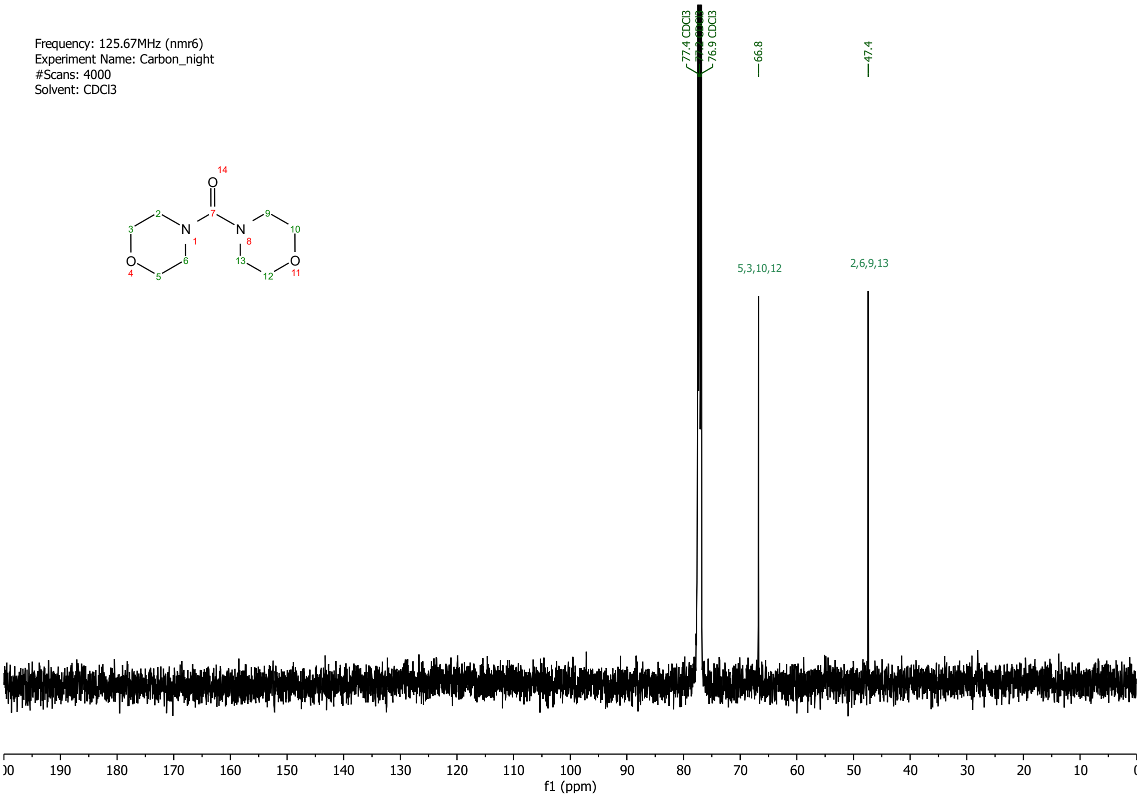

Figure S39: 13C{1H} of urea **43**

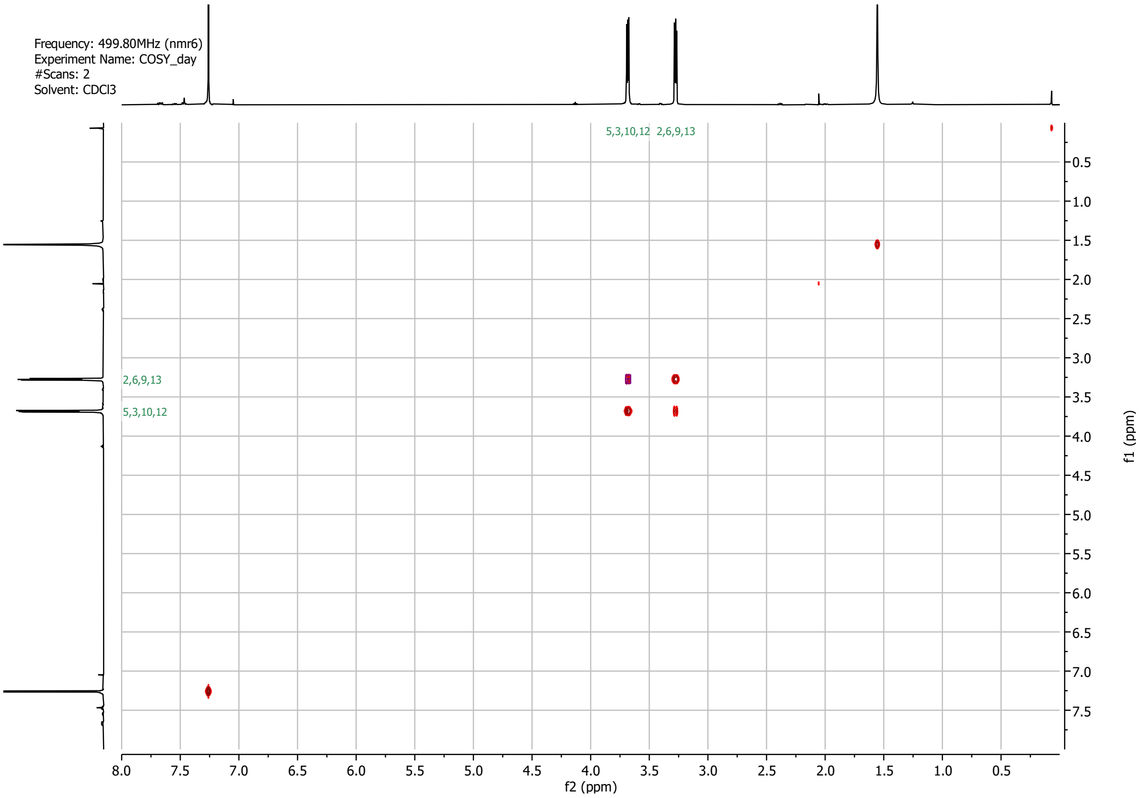

Figure S40: COSY of urea **43**

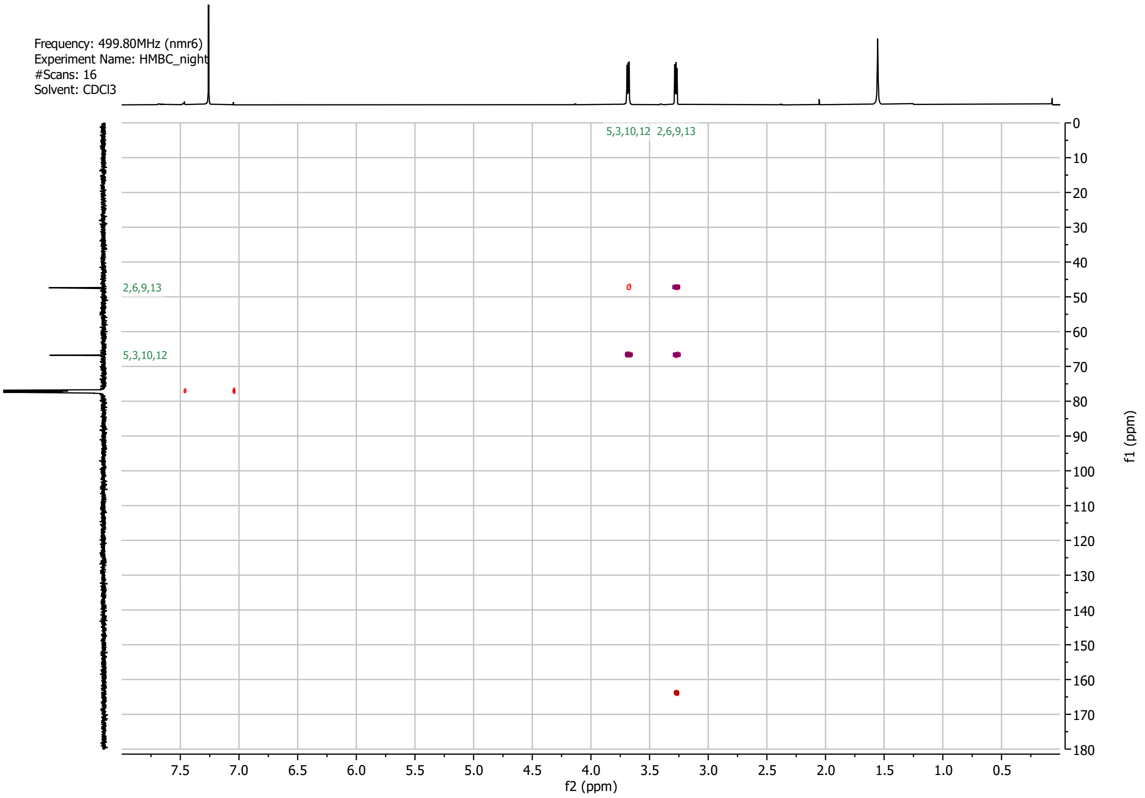

Figure S41: HMBC of urea **43**

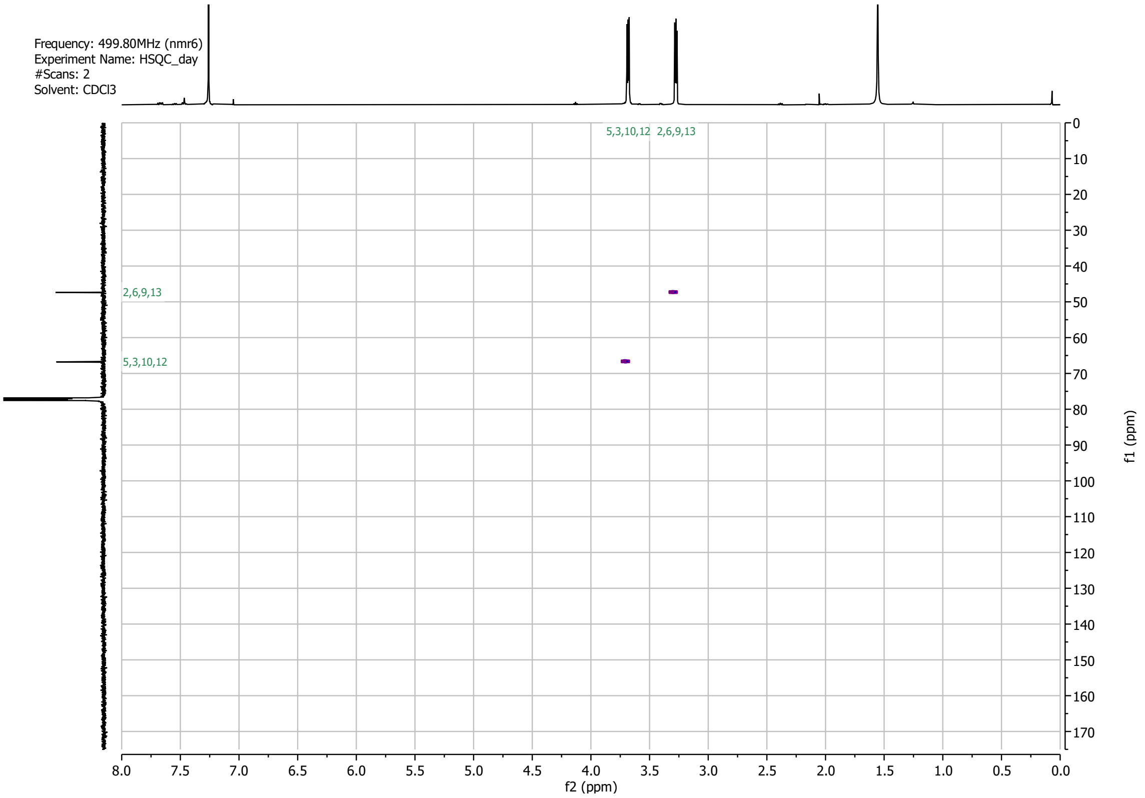

Figure S42: HSQC of urea **43**

References

(1) Sardana, M.; Bergman, J.; Ericsson, C.; Kingston, L. P.; Schou, M.; Dugave, C.; Audisio, D.; Elmore, C. S. Visible-Light-Enabled Aminocarbonylation of Unactivated Alkyl Iodides with Stoichiometric Carbon Monoxide for Application on Late-Stage Carbon Isotope Labeling. *J. Org. Chem.* **2019**, *84* (24), 16076–16085. https://doi.org/10.1021/acs.joc.9b02575.

(2) Fulmer, G. R.; Miller, A. J. M.; Sherden, N. H.; Gottlieb, H. E.; Nudelman, A.; Stoltz, B. M.; Bercaw, J. E.; Goldberg, K. I. NMR Chemical Shifts of Trace Impurities: Common Laboratory Solvents, Organics, and Gases in Deuterated Solvents Relevant to the Organometallic Chemist. *Organometallics* **2010**, *29* (9), 2176–2179. https://doi.org/10.1021/om100106e.

(3) *EvoluChem LED 525PF - HepatoChem*. https://hepatochem.com/product/hck1012-xx-004/ (accessed 2024-11-07).

(4) *PR160L | Kessil LED*. Kessil. https://www.Kessil.com/products/science_PR160L.php (accessed 2024-11-20).

(5) Kessil. Private Correspondence, 2024.

(6) Friis, S. D.; Lindhardt, A. T.; Skrydstrup, T. The Development and Application of Two-Chamber Reactors and Carbon Monoxide Precursors for Safe Carbonylation Reactions. *Acc. Chem. Res.* **2016**, *49* (4), 594–605. https://doi.org/10.1021/acs.accounts.5b00471.

(7) Mu, X.; Shibata, Y.; Makida, Y.; Fu, G. C. Control of Vicinal Stereocenters through Nickel-Catalyzed Alkyl–Alkyl Cross-Coupling. *Angew. Chem. Int. Ed.* **2017**, *56* (21), 5821–5824. https://doi.org/10.1002/anie.201702402.

(8) Soulard, V.; Villa, G.; Vollmar, D. P.; Renaud, P. Radical Deuteration with D2O: Catalysis and Mechanistic Insights. *J. Am. Chem. Soc.* **2018**, *140* (1), 155–158. https://doi.org/10.1021/jacs.7b12105.

(9) Pandey, G.; Koley, S.; Talukdar, R.; Sahani, P. K. Cross-Dehydrogenating Coupling of Aldehydes with Amines/R-OTBS Ethers by Visible-Light Photoredox Catalysis: Synthesis of Amides, Esters, and Ureas. *Org. Lett.* **2018**, *20* (18), 5861–5865. https://doi.org/10.1021/acs.orglett.8b02537.

(10) Donnier-Maréchal, M.; Goyard, D.; Folliard, V.; Docsa, T.; Gergely, P.; Praly, J.-P.; Vidal, S. 3-Glucosylated 5-Amino-1,2,4-Oxadiazoles: Synthesis and Evaluation as Glycogen Phosphorylase Inhibitors. *Beilstein J. Org. Chem.* **2015**, *11* (1), 499–503. https://doi.org/10.3762/bjoc.11.56.

(11) *CrysAlisPro*. https://rigaku.com/products/crystallography/x-ray-diffraction/crysalispro (accessed 2024-05-10).

(12) Sheldrick, G. M. SHELXT – Integrated Space-Group and Crystal-Structure Determination. *Acta Crystallogr. Sect. Found. Adv.* **2015**, *71* (1), 3–8. https://doi.org/10.1107/S2053273314026370.

(13) Sheldrick, G. M. A Short History of SHELX. *Acta Crystallogr. A* **2008**, *64* (1), 112–122. https://doi.org/10.1107/S0108767307043930.

(14) Dolomanov, O. V.; Bourhis, L. J.; Gildea, R. J.; Howard, J. a. K.; Puschmann, H. OLEX2: A Complete Structure Solution, Refinement and Analysis Program. *J. Appl. Crystallogr.* **2009**, *42* (2), 339–341. https://doi.org/10.1107/S0021889808042726.
